# Single-Cell RNA Sequencing Reveals Dynamic Transcriptional Landscape of Testicular Maturation in Dezhou Donkeys

**DOI:** 10.3390/ani16111621

**Published:** 2026-05-26

**Authors:** Zhaofei Wang, Jie Yu, Guiqin Liu, Halima Jafari, Cong Li, Ge Yang, Chuzhao Lei, Ruihua Dang

**Affiliations:** 1 Key Laboratory of Animal Genetics, Breeding and Reproduction of Shaanxi Province, College of Animal Science and Technology, Northwest A&F University, Yangling, Xianyang 712100, China; 2 National Engineering Research Center for Gelatin-Based Traditional Chinese Medicine, Dong-E-E-Jiao Co., Ltd., No. 78, E-Jiao Street, Dong’e Country, Liaocheng 252201, China; 3 College of Agriculture and Biology, Liaocheng University, 1 Hunan Road, Liaocheng 252000, China; 4 College of Animal Science and Technology, Tarim University, Alar 843300, China

**Keywords:** scRNA-seq, donkey, spermatogenesis, testicle, Cellchat

## Abstract

Testicular development and spermatogenesis are critical for male reproduction, but their molecular mechanisms in Dezhou donkeys remain understudied. This study used single-cell RNA sequencing (scRNA-seq) to analyze testicular tissues from Dezhou donkeys at juvenile (2 months), pre-pubertal (12 months), and mature (24 months) stages. A total of 24,606 high-quality cells were profiled, constructing a comprehensive single-cell transcriptional atlas. Unsupervised clustering identified nine major cell types: three germ cell subtypes (spermatogonia, spermatocytes, spermatids) and six somatic cell subtypes (Leydig cells, Sertoli cells, peritubular muscle cells, macrophages, endothelial cells, T cells). Key marker genes (AMH, TNP1, UTF1, ZMYND10) were validated by immunofluorescence. Pseudotemporal trajectory analysis revealed sequential germ cell differentiation (spermatogonia → spermatocytes → spermatids) and Sertoli cell maturation (immature → mature), while Leydig cells and peritubular muscle cells shared common progenitors. CellChat analysis identified critical ligand–receptor pairs in BMP, IGF, WNT, and FSH pathways, which regulate testicular development. This study provides the comprehensive single-cell transcriptional map of Dezhou donkey testicular development, elucidating key molecular mechanisms of germ and somatic cell maturation. The findings offer valuable insights into donkey reproductive biology, supporting breeding improvement and male infertility research.

## Simple Summary

Testicular development and sperm production are essential for male fertility, but the sperm production in equids, including donkeys, remains incompletely understood, particularly at the single-cell level. In this study, we investigated testicular development in Dezhou donkeys at three stages of growth: juvenile (2 months), pre-pubertal (12 months), and mature (24 months), using single-cell RNA sequencing. By analyzing more than 24,000 individual cells, we identified the main types of germ cells and supporting somatic cells in the testis and examined how they develop and interact over time. We also revealed key communication signals between different cell types that regulate testicular development and spermatogenesis. This study provides a detailed cellular map of donkey testicular development and offers new insights into the mechanisms controlling male reproduction, which may support future breeding programs and research on male infertility in donkeys.

## 1. Introduction

Testicular development and spermatogenesis are vital processes in the male reproductive system, playing a crucial role in sperm production and successful reproduction. During infancy, the testes are small and inactive, but with the onset of puberty, they gradually increase in size due to the activation of the hypothalamic-pituitary-gonadal axis. This growth is accompanied by complex hormonal regulation, particularly an increase in testosterone production [1].The enlargement of the testes occurs due to an increase in the number of germ cells required for spermatogenesis, which is essential for reproductive success in males. This process involves the gradual development of male germ cells starting from a juvenile state of spermatogonia, which are specialized stem cells capable of self-renewal and differentiation into mature spermatozoa [2,3]. Through a series of cell division and differentiation processes, these cells progressively develop into spermatozoa [4]. Indeed, meiosis is a specialized form of cell division that plays a key role in ensuring the production of genetically diverse spermatozoa by facilitating chromosome reorganization and double-strand breaks in the genome [5,6].

The mature spermatozoa are the end product of spermatogenesis, possessing the ability to swim towards the egg and fertilize it, leading to the formation of a zygote. It is important to emphasize that testicular development and spermatogenesis are highly complex processes regulated by multiple factors. In addition to hormonal regulation, interactions among different cell types, such as Sertoli, mesenchymal, and endothelial cells, contribute to the regulation of these processes [7]. Furthermore, there are significant differences in the duration and specifics of spermatogenesis among different mammalian species, reflecting their unique reproductive strategies and evolutionary adaptations [8]. Advancements in sequencing technology and the study of testicular development have enabled Single-cell RNA sequencing (scRNA-seq) to detect thousands of individual cells simultaneously, providing a comprehensive representation of entire organ tissue and aiding in the detection of different cellular growths during testicular development. Consequently, several studies have used scRNA-seq on animal testes, uncovering the mysteries of gene expression and cellular heterogeneity associated with spermatogenic cell development and presenting new avenues for studying mammalian spermatogenesis [9,10,11,12].

Single-cell RNA sequencing (scRNA-seq) on human and mouse testis has been a hot topic recently [13]. Donkeys play an important role in agriculture and transportation in many regions worldwide, and their economic value has increased in recent years due to their use in meat, milk, and traditional medicine. Dezhou donkeys are one of the most important native breeds in China, characterized by their large body size, high meat yield, and reproductive potential. Understanding the molecular mechanisms underlying testicular development in this breed is therefore of both biological and economic importance. However, there is a lack of studies using this method to identify key germ cell types in donkey spermatogenesis and their cell subpopulations. Therefore, this study utilized scRNA-seq technology to sequence testicular tissues of Dezhou donkeys at three different ages (2 months, 12 months, and 24 months), to detect the cellular dynamics during testicular development and spermatogenesis, and to identify marker genes specific to different testicular cell types. This research will provide new insights into the development of germ and somatic cells, as well as spermatogenesis in Dezhou donkeys enhancing our understanding of reproduction and breeding potential in this species.

## 2. Materials and Methods

### 2.1. Animals

In this experiment, we used three age groups (juvenile, pre-pubertal, and mature) of donkey from the National Dezhou Donkey Breeding Farm of Shandong Dong’e Black Donkey Ranching Science and Technology Co. Ltd. (Liaocheng, China) to collect testicular tissue samples. A total of three male donkeys’ testicular tissues were collected (one donkey from each age group). All paired testes were incised medially in the scrotum, which was found within the scrotal membrane on both the right and left sides. The testicular tissues were immediately stored in a liquid nitrogen tank and transferred to a −80 °C refrigerator for storage before undergoing total RNA extraction. Testicular tissues used for immunofluorescence staining were collected from the same animals and processed as described below.

### 2.2. Sample Preparation for scRNA-Seq

The testicular tissues were cut into small 2 mm^2^ pieces and placed into a 60 mm^2^ culture dish. To digest the tissue, an enzyme cocktail consisting of 2 mg/mL collagenase type IV (Invitrogen, Carlsbad, CA, USA), 20 μg/mL DNase (Sigma-Aldrich, St. Louis, MO, USA), and 2 mg/mL dispase (Invitrogen) was used for 20 min at 37 °C in a shaking machine (250 rpm). The resulting fragments were centrifuged at 1200 rpm for 5 min at 37 °C. The fragments were further digested in 0.25% trypsin/ethylenediaminetetraacetic acid and DNaseI, followed by vigorous shaking and incubation at 37 °C for 20 min. The single-cell suspensions were filtered using a 40 μm diameter cell strainer and washed twice with DPBS wash buffer at 37 °C. The viability of the cells was determined by trypan blue staining with a TC20 automated cell counter (Bio-Rad, Hercules, CA, USA), indicating that more than 85% of the cells were viable. The concentration of the single-cell suspension was adjusted to 700–1200 cells/μL. Finally, the cells from each sample were processed using Chromium Single Cell 3′ Reagent Kits according to the manufacturer’s instructions (v2 chemistry CG00052).

### 2.3. scRNA-Seq Library Construction and Sequencing

To generate the libraries for sequencing, the input cells were loaded onto the channel of the Single Cell A Chip (v2 chemistry, PN-120236) using the Chromium Single Cell 3′ Reagent Kits (v2 chemistry CG00052). The Chromium Controller was used to construct the 10× libraries. In brief, single-cell suspensions in each channel of the chip were loaded onto a Chromium Controller (10× Genomics, Pleasanton, CA, USA) to generate single-cell GEMs (gel beads in the emulsion). The 10× libraries for each channel were prepared using the Chromium Single Cell 3′ Gel Bead and Library Kit v2 (PN-120236, 120267, 120262) from 10× Genomics. The libraries were then sequenced using the Illumina NovaSeq sequencer (Illumina, San Diego, CA, USA) with 150 bp paired-end reads.

### 2.4. Cell Ranger Analysis

To evaluate the basic statistics on the quality of the raw reads, we utilized Fastp [14]. The raw data was processed through Cell Ranger 2.2.0 with default parameters (10× Genomics). This pipeline (https://support.10xgenomics.com/single-cell-geneexpression/software/pipelines/latest/what-is-cell-ranger, accessed on 1 November 2023) involves aligning the reads to the reference genome and generating gene–cell matrices. The genome and GTF annotation files for donkeys were obtained from the NCBI website (https://www.ncbi.nlm.nih.gov/datasets/genome/GCF_016077325.2, accessed on 1 November 2023). The gene–cell matrices, referred to as ‘filtered_gene_bc_matrices’ by 10× Genomics, were subsequently used as input data for further analyses using Seurat v3 [15].

### 2.5. Seurat Analysis

The filtered gene expression matrices from Cell Ranger were loaded into the Seurat package (v3.0) [16]. To ensure high-quality data, genes expressed in more than three single cells were retained, and the poor-quality cells were removed. Each cell was required to express a minimum of 200 genes. Subsequently, the data was normalized by the ‘logNormalize’ method, and highly variable genes were identified using the ‘FindVariableFeatures’ function, with specific criteria for the mean (0.0125–5) and dispersion cutoffs (1, Inf). These genes were used for clustering analysis, and Principal Component Analysis (PCA) was performed on the scaled data, reducing it to approximately 50 (set npcs = 50) Principal Components (PCs). For nonlinear dimensional reduction analysis, the number of PCs was used to determine the Elbowplot function. Clusters were identified using the ‘FindClusters’ with selected PCs and a resolution parameter of 0.6. The clustering resolution (0.6) and number of principal components (50) were selected based on standard practices and empirical evaluation. Sensitivity analyses using different parameter settings showed consistent cell type identification, indicating the robustness of our results. The enriched genes specific to each cluster were identified using the ‘FindAllMarkers’ function in Seurat, testing for significant differences using the ‘wilcox’ method. The enriched genes were filtered by the criteria of a minimum cell percentage (min. pct = 0.25) and a log2 fold change threshold (logfc. threshold = 0.25). Finally, the single-cell data structures and trajectories were visualized through Uniform Manifold Approximation and Projection (UMAP) techniques.

### 2.6. Differential Expression Analysis of Specific Clusters

The edgeR package was used to perform differential expression analysis between two sample clusters based on a filtered gene expression matrix obtained through Seurat [17]. This facilitated the identification of genes that exhibit differential expression patterns between distinct clusters within two samples.

### 2.7. Cell Type Identification and Pathway/Enrichment Analysis of Differential Expressed Genes

The Loupe Cell Browser was used to accurately determine the cell type of each cluster manually based on significant marker genes and cluster-enriched genes. For pathway enrichment analysis of differentially expressed genes, the cluster Profiler package was utilized to detect statistically significant enrichments within Gene Ontology (GO) terms and Kyoto Encyclopedia of Genes and Genomes (KEGG) pathways, with a significance threshold set at *p* < 0.05. Subsequently, pathway analysis was performed to enrich differentially expressed genes using KOBAS (http://kobas.cbi.pku.edu.cn, accessed on 11 November 2023), a tool utilized for functional annotation and pathway enrichment analysis.

### 2.8. Trajectory Analysis

The R package ‘Monocle2′ (version 2.20.0) was utilized to infer the developmental trajectory of specific cell lineages based on scRNA-seq data [18]. Specifically, the differentially expressed genes between various cell types were selected for the following analyses. Dimensionality reduction was performed using the ‘DDRTree’ method, which helped reduce the complexity of the data and allowed visualization in a lower-dimensional space. Cells were ordered along pseudotime to represent their developmental progression. To designate the starting point of the trajectory, we specified the zygote stage as the ‘root_state’. The ‘orderCells’ method was then reapplied to further refine the ordering of cells along the trajectory.

### 2.9. Cell Communication Analysis

The CellChat software package (version 1.1.3) was used to quantify intercellular communication networks and investigate potential cellular communication [19]. Due to incomplete receptor–ligand pair information in the donkey genome, only homologous human genes were selected for further analysis. Briefly, CellChat objects were first created using the ‘CellChat’ function. Subsequently, communication probabilities were inferred using the ‘computeCommunProb’ and ‘computeCommunProbPathway’ functions. Network centrality scores were calculated using ‘netAnalysis_computeCentrality’, and each ligand–receptor’s contribution to signaling pathways was determined with the ‘netAnalysis_contribution’ function. All graphs illustrating cellular communication were created using the visualization functions provided by the CellChat package.

### 2.10. Immunofluorescence Staining

The tissue sections underwent a series of steps for processing and staining. The sections were placed in an eco-friendly dewaxing solution for 10 min each, followed by sequential immersion in anhydrous ethanol I, anhydrous ethanol II, and anhydrous ethanol III for 5 min each. After washing with distilled water, the sections were placed in a retrieval cassette filled with Ethylenediaminetetraacetic acid (EDTA) antigen retrieval buffer (pH 8.0) and heated in a microwave oven for antigen retrieval. The heating process involved 8 min at medium heat followed by 7 min at medium-low heat to prevent excessive evaporation of the buffer. Once cooled, the sections were washed in PBS (pH 7.4) on a decolorizing shaker three times, each wash lasting 5 min. The repair solution and conditions were determined based on the specific tissues. A histochemical pen was used to draw a circle around the tissue, which was then incubated with BSA for 30 min. The primary antibody was added to the section, and it was incubated overnight at 4 °C. The sections were then placed in phosphate-buffered saline (PBS) (pH 7.4) and incubated overnight. Following incubation, the slides were incubated in PBS (pH 7.4) on a decolorizing shaker three times, for 5 min each, and then the sections were air-dried. The secondary antibody was added and incubated for 50 min at room temperature. Next, 4′,6-diamidino-2-phenylindole (DAPI) stain was applied to the sections for 10 min and incubated at room temperature, protected from light. Afterward, the sections were rinsed under running water, and an autofluorescent quencher was applied for 5 min. After washing in PBS, the sections were sealed and observed under a scanner or fluorescence microscope for imaging. DAPI was excited with UV light at a wavelength of 330–380 nm and emitting light at 420 nm (blue light). FITC was excited at a wavelength of 465–495 nm and emitting light at 515–555 nm (green light). CY3 was excited at 510–560 nm and emitting light at 590 nm (red light). CY5 was excited at 608–648 nm, with emission at 672–712 nm. The nuclei of DAPI-stained cells appeared blue under Ultraviolet ray excitation, while positive expression was indicated by corresponding red and green fluorescence. Antibody sources are detailed in Table S1. Negative controls were performed by omitting the primary antibody to ensure staining specificity.

## 3. Results and Analysis

### 3.1. Identifying Cellular Heterogeneity During Donkey Testis Development Using scRNA-Seq

**Figure 1:**
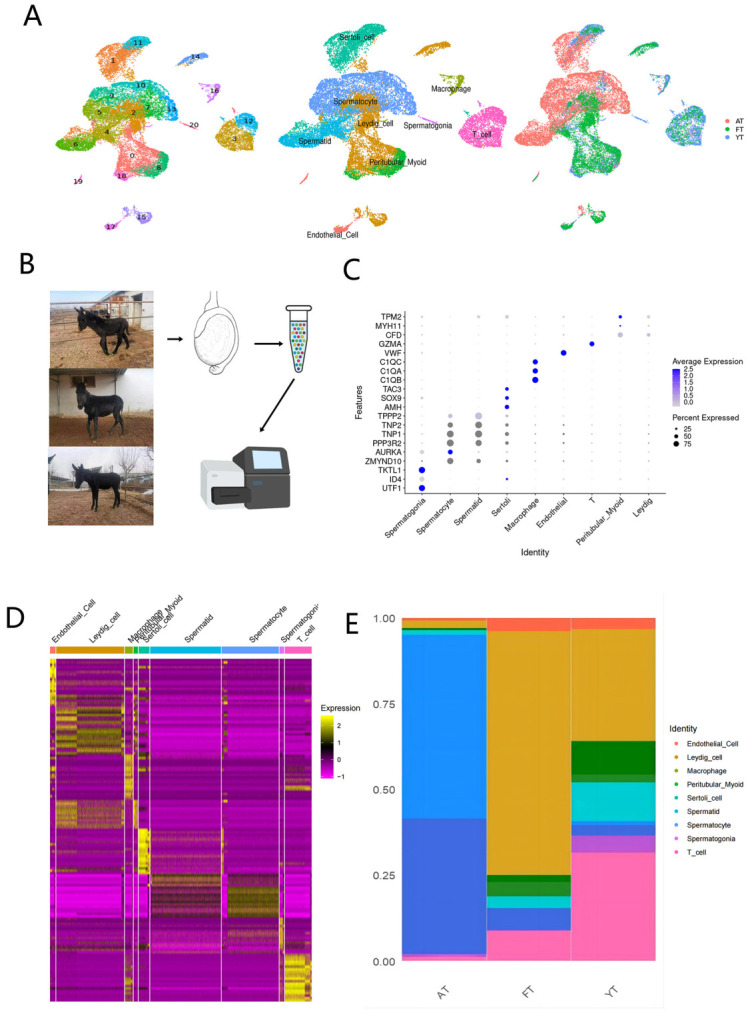
Single-cell RNA sequencing reveals cellular heterogeneity during testicular development in Dezhou donkeys. (**A**) Clusters of 20 transcribed cells were revealed by unsupervised clustering and projected on UMAP maps (left panel), UMAP maps of cells colored by cell type marked with identity (middle panel), and UMAP maps of integrated datasets from three different time points (right panel). Cells are colored according to developmental stage. AT, mature donkeys; FT, pre-pubertal donkeys; YT, juvenile donkeys. (**B**) Schematic of the experimental procedure based on the 10× Genomics platform. (**C**) The dot plot of the scRNA-seq dataset depicts the expression of cell type markers. The diameter of the dots represents the percentage of expressed cells and the color intensity corresponds to the average expression level. UTF1, ELAVL2, and TKTL2 are highly expressed in representative spermatogonia (cluster 20); AURKA, CCNA1, and TMEM225 are highly expressed in representative spermatocyte (clusters 5, 7, 9, 10, and 13); and PRSS37, TNP1, and TNP2 are highly expressed in representative spermatid (clusters 4, 6); AMH, SOX9, and TAC3 were highly expressed in representative Sertoli cells (clusters 1, 11); C1QB, C1QA, and C1QC were highly expressed in representative macrophages (cluster 16); VWF, CLDN5, and EGFL7 were highly expressed in representative endothelial cells (cluster 17); GZMA and CTSW were highly expressed in representative T cells (clusters 3, 12); MYH11 andTPM2 were highly expressed in representative peritubular muscle cells (clusters 8, 18); and DCN, COL1A2, and COL1A1 were highly expressed in representative Leydig cells (clusters 0, 2, 14, 15). (**D**) Heatmap showing the top 20 differentially expressed genes from each cell cluster from (**A**), colored according to gene expression level. (**E**) Histograms show the percentage of transcriptionally defined cell types from the three phases. Colors represent identified cell types. Figure 1B is original and created by the authors.

The present study utilized single-cell RNA sequencing to examine the transcriptional regulation of testicular development and spermatogenesis in Dezhou donkeys of different ages at the single-cell level. We performed transcriptome sequencing of testis samples and filtered the data to obtain high-quality cells for analysis (Figure 1B). Seurat software was used for data filtering. The results showed a total of 24,606 cells from three age stages: YT (2 months), FT (12 months), and AT (24 months). These filtered cells were used for further analysis. The post-QC datasets were merged using Seurat, with gene expression calibration and normalization, followed by dimensionality reduction clustering. All cells were clustered using Seurat’s clustering pipelines, as illustrated in Figure 1A, and 20 distinct cell subpopulations were identified across three developmental stages. Based on conserved known marker genes for germ cells and somatic cells, we categorized these subpopulations into nine major cell types. In germ cells, cluster 20 represents spermatogonia (*UTF1*+, *TKTL2*+), clusters 5, 7, 9, 10, and 13 consisted of spermatocytes (*AURKA*+, *ZMYND10*+), and clusters 4 and 6 were identified as meiotic spermatids (*TNP1*+, *TNP2*+). In somatic cells, clusters 0, 2, 14, and 15 corresponded to Leydig cells (*DCN*+), clusters 1 and 11 were Sertoli cells (*SOX9*+, *AMH*+), clusters 8 and 18 were peritubular muscle cells (*MYH11*+, *ACTA2*+), cluster 16 contained macrophages (*C1QB*+), cell cluster 17 was endothelial cells (*VWF*+), and cell clusters 3 and 12 were identified as T cells (*GZMA*+) (Figure 1C) [20].

After completing the identification of cell subpopulations, the percentage of each cell type was quantified at the different age stages. The analysis revealed very low numbers of germ cells in the YT and FT stages, and sperm cells were absent at these stages. However, a high proportion of spermatids was observed in AT cells, with the number of spermatids and spermatocytes exceeding half of the total cell count (Figure 1D). This result is consistent with previous reports indicating that donkeys reach sexual maturity and begin producing spermatozoa around 18 months of age. Furthermore, Sertoli cells increased in percentage with age, suggesting their significance in testicular development. Finally, the accuracy of cell type identification was confirmed through heat maps of cell type-specific genes (top 100), as shown in Figure 1E. In summary, the scRNA-seq study successfully characterized nine cell types in Dezhou donkey testes across three age stages. This provides a base for understanding the transcriptional regulation of testicular development and spermatogenesis in donkey.

### 3.2. Temporal Trajectory Analysis Reveals Cellular Developmental Trajectories of Testicular Germ Cells in the Dezhou Donkey (Equus asinus)

**Figure 2:**
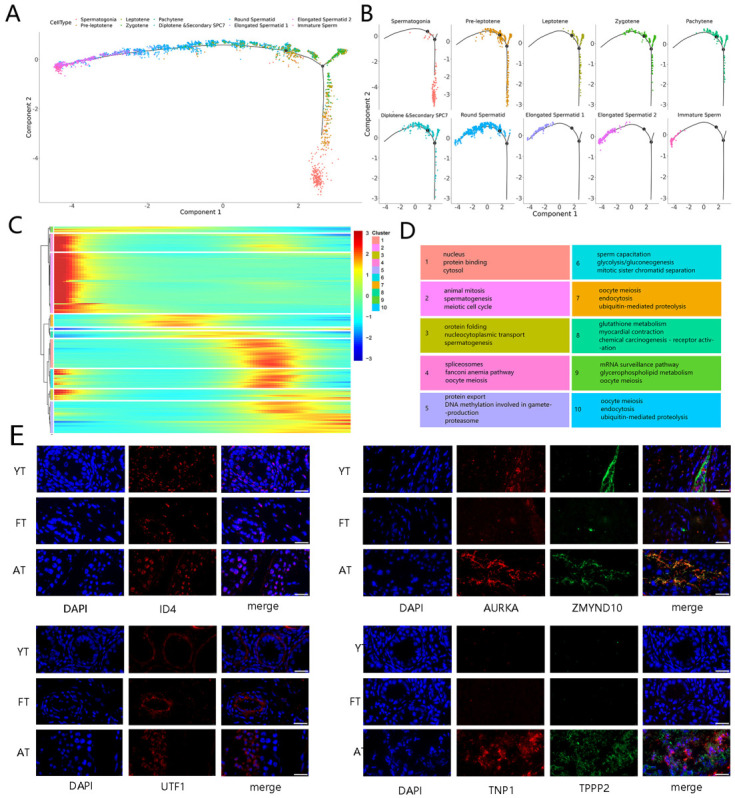
Differential gene expression profile during testicular germ cell development in Dezhou donkey. (**A**) Pseudo-temporal analysis of donkey testicular germ cell subsets by Monocle 2. Pseudo-time coloring based on cell type in the trace diagram. (**B**) The distribution of different cell types in pseudo-time trajectories. (**C**) Clusters of genes that exhibit differential expression along germ cell lineages. Each row represents a gene and each column represents a single cell, where columns/cells are placed in the pseudo-chronological order defined in (**A**). (**D**) Enriched GO-BP terminology for the ten gene clusters. (**E**) Immunofluorescence staining of UTF1 and ID4 markers of spermatogonia cells of different ages (**upper left** and **upper right**); Immunofluorescence co-staining of spermatocyte marker genes *AURKA* and *ZMYND10* at different ages (**lower left**); Immunofluorescence costaining of sperm cell marker genes *TNP1* and *TPPP2* at different ages (**lower right**). The scale bars represent 20 μm.

The composition of Dezhou donkey germ cells was further analyzed during these three periods. We reclassified three types of germ cells (spermatogonia, spermatocytes, and spermatids) from all cells. Spermatogonia, being less abundant and lacking subgroup differentiation, was not categorized further. On the other hand, spermatocytes were categorized into different subtypes, including pre-leptotene, leptotene, zygotene, pachytene, diplotene, and Secondary SPC. Spermatids were further classified as round spermatids, extended spermatid 1, extended spermatid 2, and immature sperm. The differentiation trajectories of these germ cells were analyzed using Monocle 2, a computational algorithm that allows for the ordering of individual cells along a developmental trajectory. The analysis results revealed that the germ cells followed a differentiation trajectory from spermatogonia to spermatocytes and then to spermatids (Figure 2A). This proposed temporal trajectory provides basic insight into the developmental progression of donkey spermatogenesis (Figure 2B).

To explore a greater extent of molecular factors which influence different cellular states, gene expression analysis was conducted along pseudotemporal trajectories, revealing 10 distinct gene clusters with varied expression patterns (Figure 2C). These 10 cellular clusters contained different numbers of genes: 1961, 4135, 725, 1215, 2176, 1287, 818, 429, 393, and 128 genes, respectively (Table S2). The expression patterns of these genes showed that some increased or decreased consistently with activation, while others exhibited non-monotonic behavior. For example, gene cluster 5 showed a consistent increase in pseudotime, with elevated expression of genes such as *KLHL36*, *TLDC1*, *WFDC1*, *ADAD2*, *DNAAF1*, *HSBP1*, and *CENPN*. These genes were associated with GO-BP terms such as ‘protein export’, ‘DNA methylation involved in germ cell production’, and ‘proteasome’, indicating their involvement in germ cell genesis. On the other hand, gene clusters 2 and 4 demonstrated a consistent decrease in pseudotime, featuring high expression levels of genes like *SLC7A5*, *KLHDC4*, *USP10*, *FBXO31*, *ZDHHC7*, and *TAF1C*. These clusters were enriched for terms related to ‘animal mitosis’, ‘spermatogenesis’, ‘spermatogonium’, and ‘meiotic cell cycle’, indicating their involvement in germ cell meiosis. For gene clusters 1, 3, and 6–10, non-monotonic gene expression trends were observed for genes like *BANP*, *MAP1LC3B*, *MTHFSD*, *GINS2*, *HOXC4*, *KARS*, and *GPS1*. These genes were associated with diverse GO-BP terms such as ‘nucleoplasmic transport’, ‘spermatogenesis’, ‘sperm acrosome formation’, ‘oocyte meiosis’, and ‘mitotic sister chromatid segregation’, suggesting a role in the maintenance of normal spermatozoa and the process of spermatogenesis (Figure 2D). Overall, we successfully characterized the transcriptional program during germ cell development, highlighting both monotonic and non-monotonic temporal features that indicate the heterogeneous nature of spermatogenesis at different developmental stages.

Additionally, we utilized immunofluorescence to confirm the expression of specific marker genes in different types of germ cells (Figure 2E). The *UTF1* and *ID4* genes are specifically observed in spermatogonia, expressed at the edges of the tubular lumen, consistent with the known localization of spermatogonia in all three stages. Fluorescence co-localization of *AURKA* and *ZMYND10* in spermatocytes was also performed, and it was observed that these genes were expressed in YT and FT and more prominently in AT and localized inside the lumen as expected for spermatocytes. Lastly, we analyzed the co-localization of *TNP2* and *TPPP2* in spermatids and found that these genes were expressed only in AT. Through immunofluorescence, we successfully confirmed the specific labeling of these genes in testicular germ cells in donkeys.

### 3.3. Temporal Trajectory Analysis Reveals Cellular Developmental Trajectories of Testicular Somatic Cells in Dezhou Donkeys

**Figure 3:**
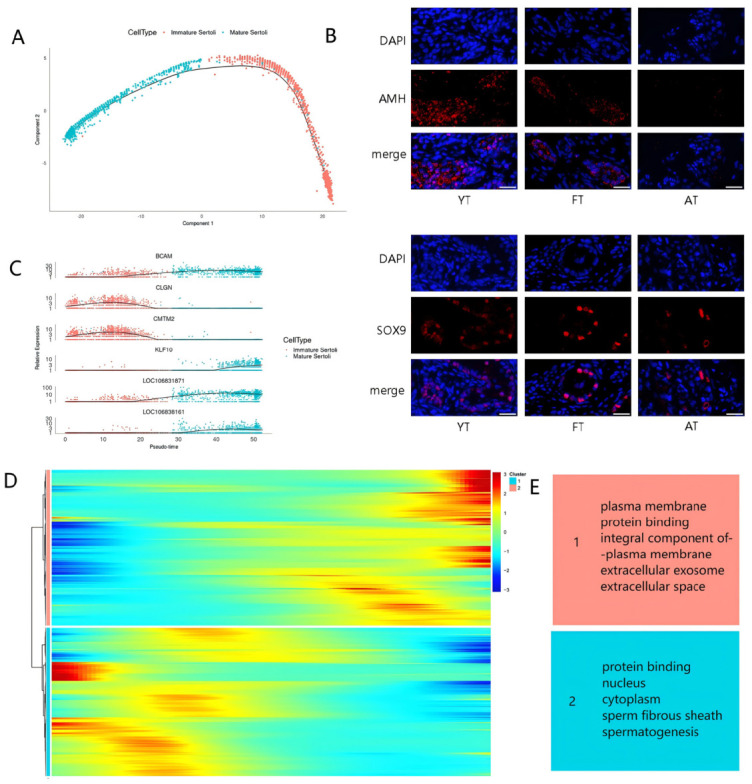
Differential gene expression profile during testicular Sertoli cell development in Dezhou donkey. (**A**) Pseudo-temporal analysis of donkey testicular Sertoli cell subsets by Monocle 2. Pseudo-time coloring based on cell type in the trace diagram. (**B**) Expression levels of the six marker genes at the pseudo time (scatter plot). The *X*-axis represents pseudo time; The *Y*-axis represents standardized gene expression levels; the color refers to two subpopulations of Sertoli cells. (**C**) Clustering of genes that exhibit differential expression along Sertoli cell lineages. Each row represents a gene and each column represents a single cell, where columns/cells are placed in the pseudo-chronological order defined in (**A**). (**D**) Enriched GO-BP terminology for both gene clusters. (**E**) Immunofluorescence staining of Sertoli cell marker genes AMH and SOX9 at different ages. The scale bars represent 20 μm.

Comparable approaches were applied to analyze changes in the populations of Sertoli cells, Leydig cells, and peritubular muscle cells during three periods for a deeper clarity of testicular somatic cell compositions in Dezhou donkeys. Sertoli cells were isolated from the cell population, and a differentiation path was constructed using Monocle 2 software. Through this analysis, we observed a clear trajectory of differentiation for Sertoli cells, progressing from an immature state to a mature state (Figure 3A). In addition, trajectory inference was strengthened by assessing the trends of Sertoli gene expression across pseudotime with known Sertoli biology (Figure 3B). Further gene expression analysis along the pseudo temporal trajectory detected two distinct gene clusters (Figure 3C) containing 4072 and 4280 genes, respectively (Table S3). These clusters displayed opposite expression patterns. Genes in cluster 1 demonstrated a gradual increase in expression over pseudotime, including elevated expression of genes such as *BANP*, *SLC7A5*, *MAP1LC3B*, *FBXO31*, and *MTHFSD*. These genes were enriched with ‘plasma membrane’, ‘protein binding’, ‘integral component of plasma membrane’, ‘protein binding’, and ‘integral component of plasma membrane’ GO-BP terms. In contrast, genes in cluster 2 exhibited a gradual decrease in expression over pseudotime, with high expression levels of *GSE1*, *USP10*, *COTL1*, *TAF1C*, and *MBTPS1*. These genes were enriched in GO-BP terms related to ‘protein binding’, ‘sperm’, ‘fibrous sheath’, ‘spermatogenesis’, and ‘spliceosome’, suggesting their potential involvement in spermatogenesis also plays a role in the heterogeneous cellular process at multiple time points during development (Figure 3D).

Immunofluorescence was once again utilized to validate the presence of two marker genes, *SOX9* and *AMH*, in Sertoli cells as depicted in Figure 3E. *SOX9* is a widely recognized marker gene for support cells in various species (Chen et al., 2019 [21]), whereas *AMH* is typically associated with immature Sertoli cells. Our findings indicated the specific expression of *AMH* in Sertoli cells. The immunofluorescence analysis revealed a notable expression of *AMH* in YT cells, which gradually diminished with age, suggesting a reduction in the population of immature Sertoli cells in AT cells.

**Figure 4:**
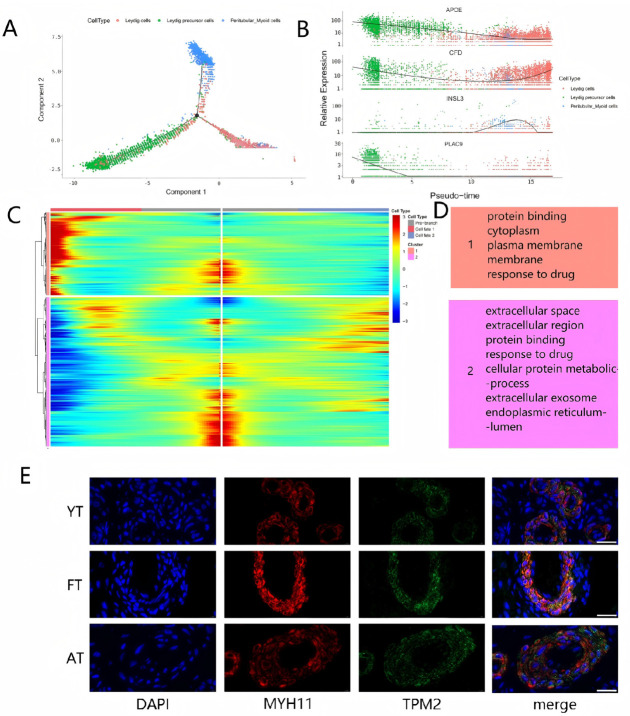
Differential gene expression profiles of testicular Leydig cells and peritubular muscle cells during development in Dezhou donkey. (**A**) Pseudo-temporal analysis of donkey testicular Leydig cells and peritubular muscle cells subsets by Monocle 2. Pseudo-time coloring based on cell type in the trace diagram. (**B**) The expression levels of the four marker genes at spurious times (scatter plot). The *X*-axis represents pseudo time; The *Y*-axis represents standardized gene expression levels; And color refers to three subgroups of cells. (**C**) Clusters of differentially expressed genes along Leydig and peritubular muscle lineages. Each row represents a gene, and each column represents a single cell, where columns/cells are placed in the pseudo-chronological order defined in (**A**). (**D**) Enriched GO-BP terminology for both gene clusters. (**E**) Immunofluorescence co-staining of peritubular muscle marker genes MYH11 and TPM2 at different ages. The scale bars represent 20 μm.

Identifying Leydig cells and peritubular muscle cells posed difficulties due to the presence of overlapping marker genes. Therefore, we combine these cells into a single dataset for further analysis using reclustering techniques. The results showed that both cell types are derived from the same progenitor cells and differentiate into different cell types at a certain point (Figure 4A). To enhance the accuracy of trajectory inference, gene expression trends in Leydig cells and peritubular muscle cells were analyzed across pseudotime, while considering the known biology of these cell types (Figure 4B). To investigate their gene expression, we used Monocle to analyze the time-sorted cell data as well as the specified nodes using the BEAM (Branched Expression Analysis Modeling) method (Figure 4C). This allowed us to identify the differential genes associated with branching. We observed two distinct cell clusters with different gene expression patterns. In cluster 1, genes were significantly upregulated in cell fate1, with elevated expression of *INSL3, GSTO1, LHCGR, GRB14*, and *VGLL3*,while in cluster 2, most genes exhibited decreased expression in cell fate1, showing high levels of expression of *APOE, HSPA6, DCN, SPARCL1, YBX3*, and *CFD* in cell fate2. This differential gene expression demonstrated the difference in cell fate between the two clusters.

Additionally, we performed immunofluorescence to validate the expression of specific marker genes (Figure 4D). As for peritubular myocytes, we chose *MYH11* and *TPM2*, which are well-known marker genes for peritubular muscle cells [22], and their expression was highly consistent in testicular tissue.

### 3.4. Prediction of Ligand–Receptor Interactions During Testicular Development in Dezhou Donkeys

**Figure 5:**
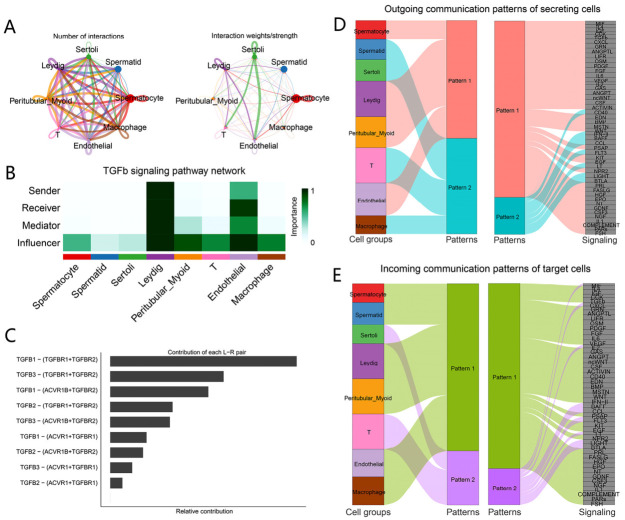
Mature testicular (AT) germ cells and somatic communication. (**A**) ligand–receptor pairs in the AT stage. The number (**left**) and intensity (**right**) of cell interactions (ligand–receptor pairs) are shown in the figure, where the colored dots represent different cell groups and the lines with arrows point to groups of cells expressing homologous receptors. The thickness of the lines is proportional to the number (**left**) or strength (**right**) of the ligand–receptor pairs, while the ring refers to the autocrine circuit. (**B**) The heat map shows the role of each cell population (sender, receiver, mediator, or influencer) in TGF-β signaling during the AT phase. (**C**) Contribution diagram of AT-phase ligand–receptor to TGF-β signaling pathway. (**D**,**E**) Outgoing (**D**) and incoming (**E**) communication patterns of secretory cells in the AT phase. Flow thickness represents the contribution of the signal path to each mode of communication.

To identify the interactions between ligands and receptors involved in intercellular communication, we performed CellChat analysis and constructed dense networks of 1539, 1219, and 2191 significant cellular interactions (*p* ≤ 0.05) in the YT, FT, and AT phases, respectively (Figure 5A, Figures S1A and S2A). The analysis delineated many ligand–receptor pairs across nine cell clusters, which were categorized into important signaling pathways, including MIF, IGF, WNT, CXCL, ANGPTL, TGFβ, FSH, BMP, and FGF, among others.

Previous studies have suggested that TGFβ ligand-activated signaling pathways play a key role in regulating testicular development [23,24]. In this study, we specifically focused on examining how TGFβ-based communication is altered during testicular development in Dezhou donkeys. Our results revealed that Leydig cells were the main source of TGFβ ligands at all three stages of development (Figure 5B, Figures S1B and S2B). However, in the AT stage, there was a significant increase in the contribution of TGFβ receptors from endothelial cells. Interestingly, the TGFβ ligand–receptor pair TGFβ1-(TGFβR1 + TGFβR2) emerged as the major contributor to this communication network (Figure 5E, Figures S1E and S2E), which is consistent with previous findings [25]. These findings support the existing evidence suggesting the involvement of the TGFβ signaling pathway in testis development. In this context, we hypothesized that there are specific cell–cell interaction mechanisms driving testis development during the developmental stages.

Additionally, we observed the involvement of several signaling pathways at different time points during testicular development in Dezhou donkeys. For example, the BMP signaling pathway changed from being primarily associated with Sertoli cells during the YT stage to being predominantly expressed in peritubular muscle cells during the FT and AT stages (Figure S3). Similarly, the FSH signaling pathway shifted from Sertoli cells during the YT and FT stages to being more prominent in endothelial cells during the AT stage (Figure S4). Furthermore, the WNT pathway showed a change in the source of ligand, moving from Sertoli cells to spermatid cells after sexual maturation (Figure S5). These findings indicate the presence of a complex regulatory network involving various signaling pathways that contribute to testicular development, including skeletal muscle formation.

To explore how different cell populations and signaling pathways work together during myogenesis, we utilized the CellChat pattern recognition module, which showed that certain cell types activate multiple signaling pathways simultaneously and depend on overlapping afferent and efferent signaling networks at different developmental stages. Through this analysis, we identified two, three, and two patterns of outgoing and incoming signaling in the YT, FT, and AT stages (Figure 5D, Figures S1D and S2D). These patterns represent a variety of signaling pathways over time. Notably, Leydig cells and peritubular myoid cells exhibited similar patterns of signaling afferents and efferents during different phases. In addition, macrophages and T cells showed the same efferent pattern during the AY and YT stages, whereas macrophages and Sertoli cells had an independent efferent and afferent pattern during the FT stage. These findings may contribute to a deeper understanding of the regulatory mechanisms driving skeletal muscle lineage differentiation and the complex interplay between different cell populations and signaling pathways during development.

## 4. Discussion

The RNA-seq technique is tissue-based, and it captures signals from a mixture of cells, averaging out the heterogeneity of individual cell types. Therefore, single-cell sequencing is now increasingly favored by researchers. Remarkable progress has been made in single-cell mapping of testicular development in humans and mice [11,26]. However, the application of this technique in donkeys has not been well explored, and there have been no reports of single-cell level studies of testicular development and spermatogenesis in donkeys. In this study, single-cell transcriptome sequencing technology was used to comprehensively analyze the expression rules of key genes in germ cells and somatic cells during the spermatogenesis process in Dezhou donkeys. Based on UMAP analysis [27], we analyzed the complex process of donkey spermatogenesis. A total of 24,606 cells were divided into 20 clusters, which were in turn regrouped into nine different cell types. Although the total number of cells (24,606) may appear modest, it is comparable to or within the range of previously published single-cell studies in reproductive biology. Importantly, the dataset captures all major cell types in the testis, supporting the robustness of downstream analyses. Our findings are generally consistent with previous scRNA-seq studies in human, mouse, and bovine testes, particularly in the identification of major germ and somatic cell populations and the dynamic transcriptional changes during spermatogenesis. However, we also observed donkey-specific transcriptional features, including distinct stage-specific expression patterns of several genes during pseudotime analysis. In addition, differences in cell population proportions and intercellular communication patterns suggest potential species-specific regulatory mechanisms during donkey spermatogenesis.

The germ cells were regrouped and the results were found to be similar to those in human and mouse studies. For example, similar to the study in mice [28], this study found that *UTF1* was exclusively expressed in spermatogonium cells in donkey testis, serving as a crucial marker for germ cells. In addition, immunofluorescence staining of testicular tissue at different developmental stages indicated a gradual down-regulation of UTF1 expression with age, aligning with expected outcomes. Monocle2 was used to conduct a detailed analysis of spermatocytes and sperm cells, infer the developmental trajectory of testicular germ cells of Dezhou donkey, and identify distinct functional subgroups at different stages. Although the root state was set as the earliest developmental stage for trajectory inference, we acknowledge that this choice may not fully reflect the biological origin of germ cell development. Similarly, this study highlighted upregulated genes associated with meiosis in spermatocytes, such as *ZMYND10* and *AURKA* [29]. In addition, several genes highly expressed in sperm, including *TNP1*, *TNP2*, and *TPPP2*, were found to be enriched in the ‘spermatogenic’ and ‘sperm capacitation’ pathways [30,31,32]. The variations in gene expression across different cells reflect the dynamic nature of gene expression during spermatogenesis in Dezhou donkeys. Through comprehensive cell subpopulation division and quasi-temporal analysis, this study provides insights into the dynamic process of testicular germ cell development in Dezhou donkeys, which is helpful for revealing the key regulatory mechanisms of germ cell development.

The testicular cells were also further clustered. Sertoli cells play a crucial role in supporting and regulating spermatogenesis [33,34]. In donkey testicular tissue, we found classical Sertoli cell marker genes, such as *AMH* and *SOX9*, which are closely related to Sertoli cell maturation [35]. We also performed cell subpopulation division and simulation time analysis of immature Sertoli cells at different developmental stages and Sertoli cells with different gene expressions. The testis of the Dezhou donkey was stained by immunofluorescence with *AMH* and *SOX9* antibodies. AMH was mainly expressed in immature testis, which may be related to the decrease of somatic cell numbers after testis maturation. The expression level of *SOX9* was higher in the testis of sexually mature donkeys. It is suggested that these genes act as marker genes in donkey testes, and the number of mature Sertoli cells is higher in sexually mature testes. Leydig cells and peritubular myoid cells also play a vital role in testicular physiology and development. The two cell types share a common progenitor cell and have many shared marker genes when defining cell types. We extracted the two cells together for reclustering and simulation analysis and plotted the differentiation trajectories of their cells with different fates [36]. After cell differentiation, Leydig cells highly express genes such as *STAR*, which is essential for cholesterol transport to mitochondrial hormone-induced steroid production [37]. *ACTA2*, *MYH11* and *TPM2* genes were highly expressed in peritubular myoid cells. For example, *ACTA2* can act as a molecular marker for testicular cells in macaque monkeys [38]. *MYH11* encodes the myosin heavy chain, which play a key role in smooth muscle cells, and finally, *TPM2* encodes the actin light chain, a component of the actin molecule whose role in the major cytoskeleton protein in muscle is involved in many cellular motor processes, including muscle contraction, cell oscillation, and cytoplasmic flow [39,40]. These three genes are involved in the contraction and relaxation of smooth muscle cells and affect cell differentiation and proliferation [22,41,42]. These findings reveal the important role of Leydig cells and peritubular muscle cells in testicular physiology and development. These findings provide valuable insights into the molecular regulation of spermatogenesis in donkeys, which may contribute to improving reproductive efficiency in breeding programs. In addition, the identification of key regulatory genes and signaling pathways may offer potential targets for the diagnosis and treatment of infertility in donkeys. A major limitation of this study is that only one animal was included per age group, which may limit the generalizability of the findings. Inter-individual variation could potentially influence the observed transcriptional patterns. Future studies with larger sample sizes are needed to validate these results.

In multicellular organisms, the formation and regulation of cell networks are key factors in maintaining tissue homeostasis and function. These networks involve complex intercellular interactions that can be achieved through cellular autocrine or paracrine signaling [43]. The use of homologous human ligand–receptor pairs may introduce bias, as species-specific interactions in donkeys are not fully characterized. This limitation should be considered when interpreting signaling pathway analyses. In addition to local interactions, long-distance communication between cells via extracellular signals plays a crucial role in regulating cell fate decisions [44]. Studies have shown that dense ligand–receptor networks between different cell types facilitate extensive intercellular communication. This specificity can help identify dimorphism at the level of gene expression and regulatory programs unique to each cell population [45]. In the testis, the BMP signaling pathway shows that at different stages of development (FT and AT), the primary receptor source shifts from the Sertoli cells (YT) to the peritubule muscle cells. Similarly, the FSH and WNT signaling pathways change in different cell types during testicular development and maturation, and their major receptor and ligand sources change. These ligand–receptor interactions at the protein level could further improve CellChat’s modeling accuracy and enhance understanding of cellular communication mechanisms.

## 5. Conclusions

In conclusion, we have successfully generated a single-cell transcriptional map of the Dezhou donkey testis and performed mimicry analysis on different cell types to determine the differentiation nodes and stages of transcriptional regulation in these cells. Next, the immunofluorescence assay validated certain marker genes and mapped the cellular ligand–receptor regulatory network at various time points. This analysis elucidated the regulation of testicular cellular signaling pathways in Dezhou donkeys during different development stages. This research establishes a framework for further studies on donkey reproduction, and molecular mechanisms underlying testicular development and maintenance.

## Data Availability

The single-cell sequencing data used in this research is deposited in CNCB GSA databases under accession number: CRA015500. The authors did not use any artificial intelligence-assisted technologies in the writing process.

## Supplementary Materials

The following supporting information can be downloaded at: https://www.mdpi.com/article/10.3390/ani16111621/s1. Table S1: List of antibody sources used in immunofluorescence staining. Table S2: List of 10 gene clusters in germ cells. Table S3: List of two gene clusters that Sertoli cells. Figure S1: Pre-mature testicular(FT) germ cells and somatic communication. Figure S2: Juvenile testicular(YT) germ cells and somatic communication. Figure S3: Communication modes of BMP signal paths in different periods. Figure S4: Communication modes of FSH signal paths in different periods. Figure S5: Communication modes of WNT signal paths in different periods.
