# Supplementary material for: Single-Cell RNA Sequencing Reveals Dynamic Transcriptional Landscape of Testicular Maturation in Dezhou Donkeys
Source: Animals (Basel). 2026 May 26;16(11):1621. doi: 10.3390/ani16111621 (PMC13255784; doi:10.3390/ani16111621)
Supplement: Supplementary file 1 [file animals-16-01621-s001.zip › File S1. figures and table captions.pdf]

Table S1: List of antibody sources used in immunofluorescence staining

Table S2: List of 10 gene clusters in germ cells

Table S3: List of two gene clusters that Sertoli cells

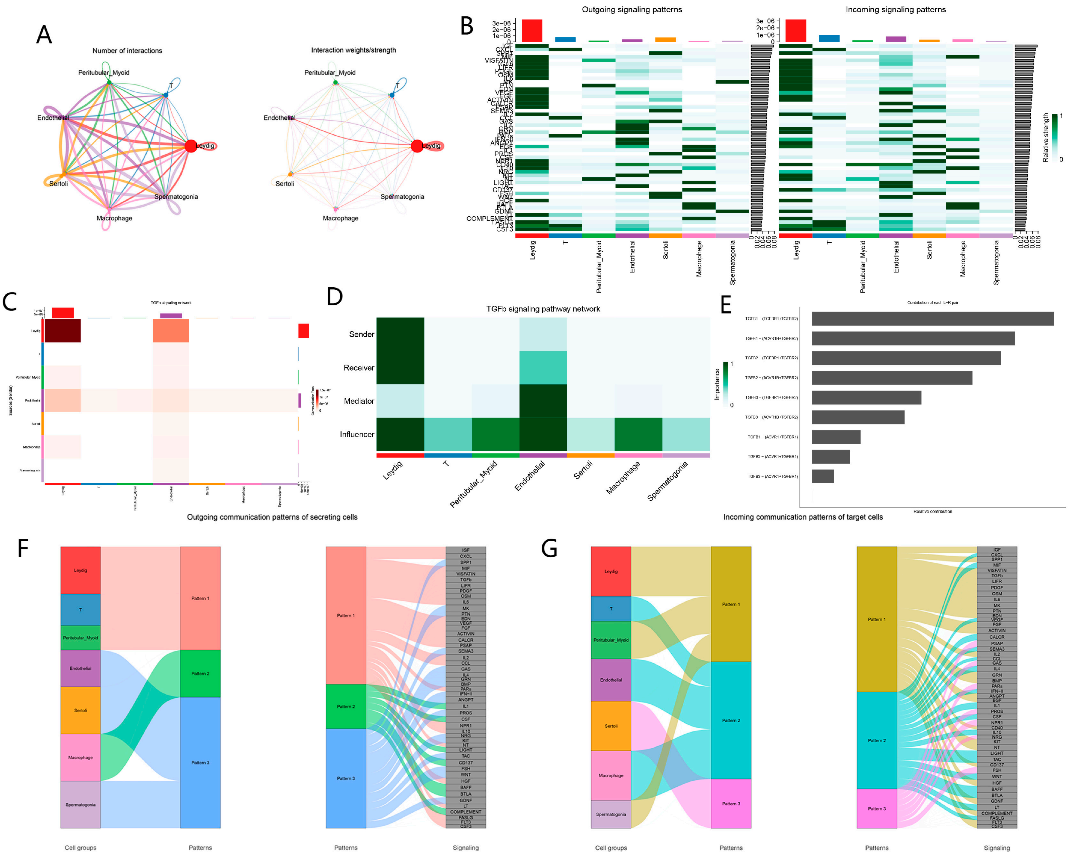

Figure S1: Pre-mature testicular(FT) germ cells and somatic communication.(A) ligand-receptor pairs in the FT stage. The number (left) and intensity (right) of cell interactions (ligand-receptor pairs) are shown in the figure, where the colored dots represent different cell groups and the lines with arrows point to groups of cells expressing homologous receptors. The thickness of the lines is proportional to the number (left) or strength (right) of the ligand-receptor pairs, while the ring refers to the autocrine circuit.(B) The contribution of multiple signaling pathways to outgoing(left) and incoming(right) signals from different cell clusters.(C) The interaction of different cell types in the TGF-β signaling pathway at the FT stage, with the vertical and horizontal coordinates representing the source and target, respectively, and the color representing the signal strength.(D) The

heat map shows the role of each cell population (sender, receiver, mediator, or influencer) in TGF-β signaling during the FT phase.(E) Contribution diagram of FT-phase ligand-receptor to TGF-β signaling pathway.(F) Outgoing (left) and incoming (right) communication patterns of secretory cells in the FT phase. Flow thickness represents the contribution of the signal path to each mode of communication.

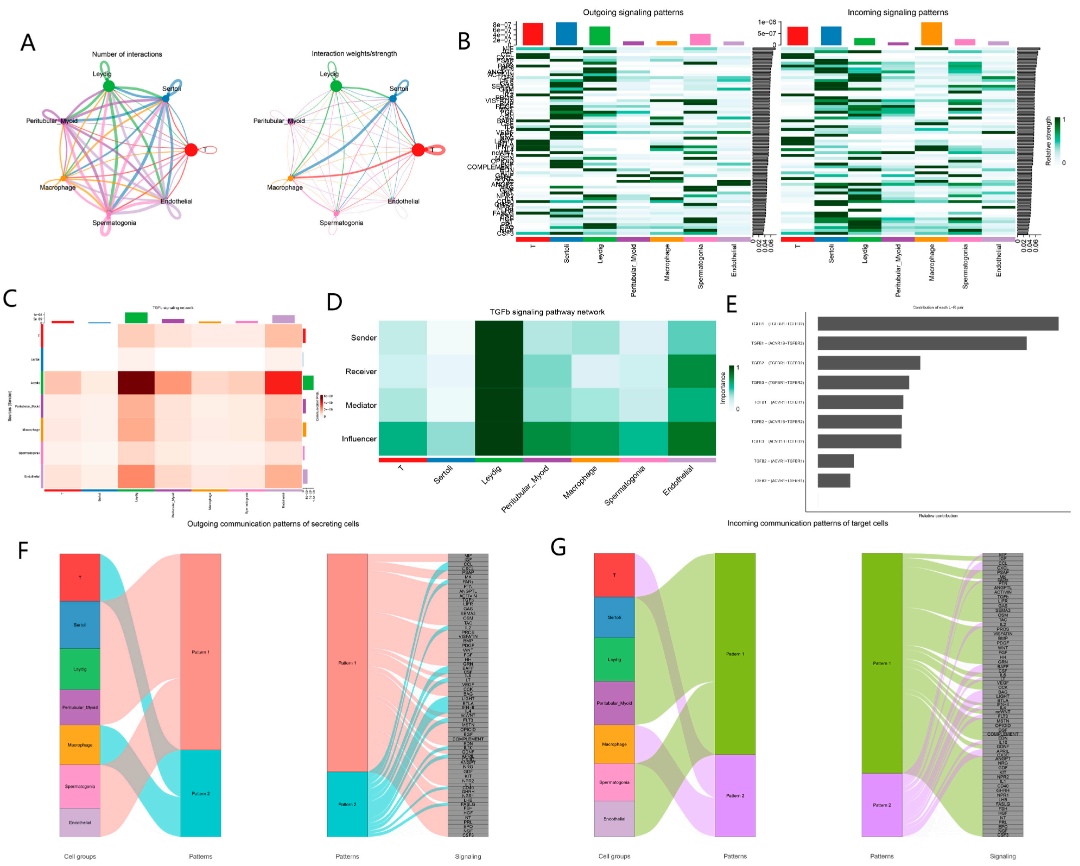

Figure S2: Juvenile testicular(YT) germ cells and somatic communication.(A) ligand-receptor pairs in the YT stage. The number (left) and intensity (right) of cell interactions (ligand-receptor pairs) are shown in the figure, where the colored dots represent different cell groups and the lines with arrows point to groups of cells expressing homologous receptors. The thickness of the lines is proportional to the number (left) or strength (right) of the ligand-receptor pairs, while the ring refers to the autocrine circuit.(B) The contribution of multiple signaling pathways to outgoing(left) and incoming(right) signals from different cell clusters.(C) The interaction of different cell types in the TGF-β signaling pathway at the YT stage, with the vertical and horizontal coordinates representing the source and target, respectively, and the color representing the signal

strength.(D) The heat map shows the role of each cell population (sender, receiver, mediator, or influencer) in TGF-β signaling during the YT phase.(E) Contribution diagram of YT-phase ligand-receptor to TGF-β signaling pathway.(F) Outgoing (left) and incoming (right) communication patterns of secretory cells in the YT phase. Flow thickness represents the contribution of the signal path to each mode of communication.

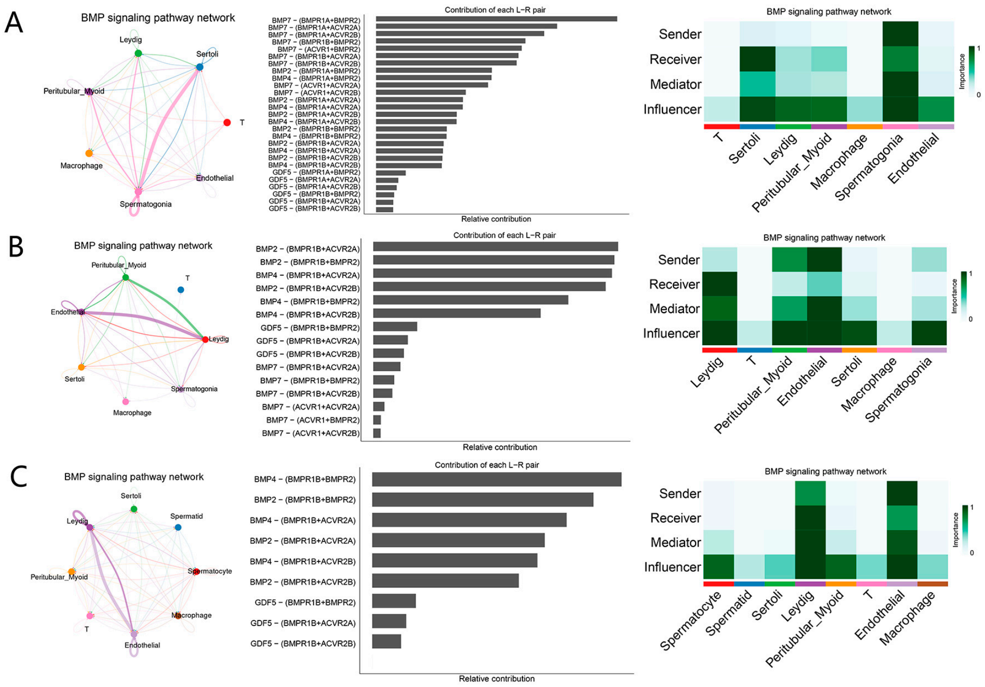

Figure S3:Communication modes of BMP signal paths in different periods.(A) The communication mode of BMP signaling pathway in YT stage. From left to right, (1)The BMP signal channel communication network diagram. (2)Contribution diagram of ligand-receptor to BMP signaling pathway. (3)The heat map shows the role of each cell population (sender, receiver, mediator, or influencer) in BMP signaling pathway. (B) The communication mode of BMP signaling pathway in FT stage.(C) The communication mode of BMP signaling pathway in AT stage.

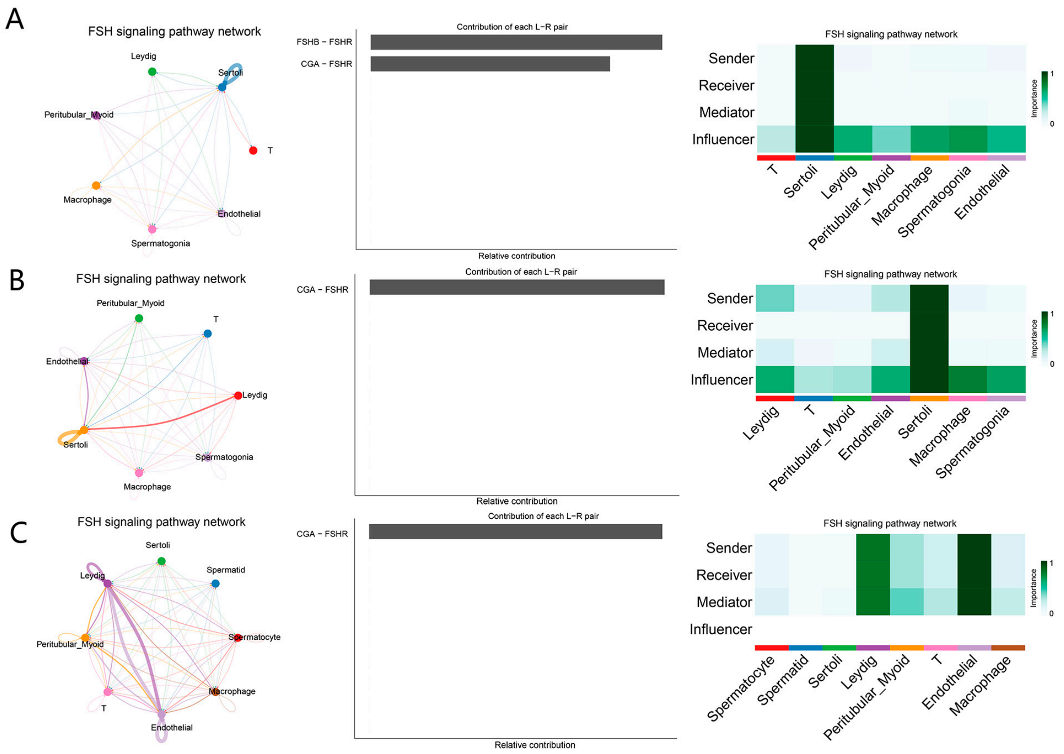

Figure S4:Communication modes of FSH signal paths in different periods.(A) The communication mode of FSH signaling pathway in YT stage. From left to right, (1)The FSH signal channel communication network diagram. (2)Contribution diagram of ligand-receptor to FSH signaling pathway. (3)The heat map shows the role of each cell population (sender, receiver, mediator, or influencer) in FSH signaling pathway. (B) The communication mode of FSH signaling pathway in FT stage.(C) The communication mode of FSH signaling pathway in AT stage.

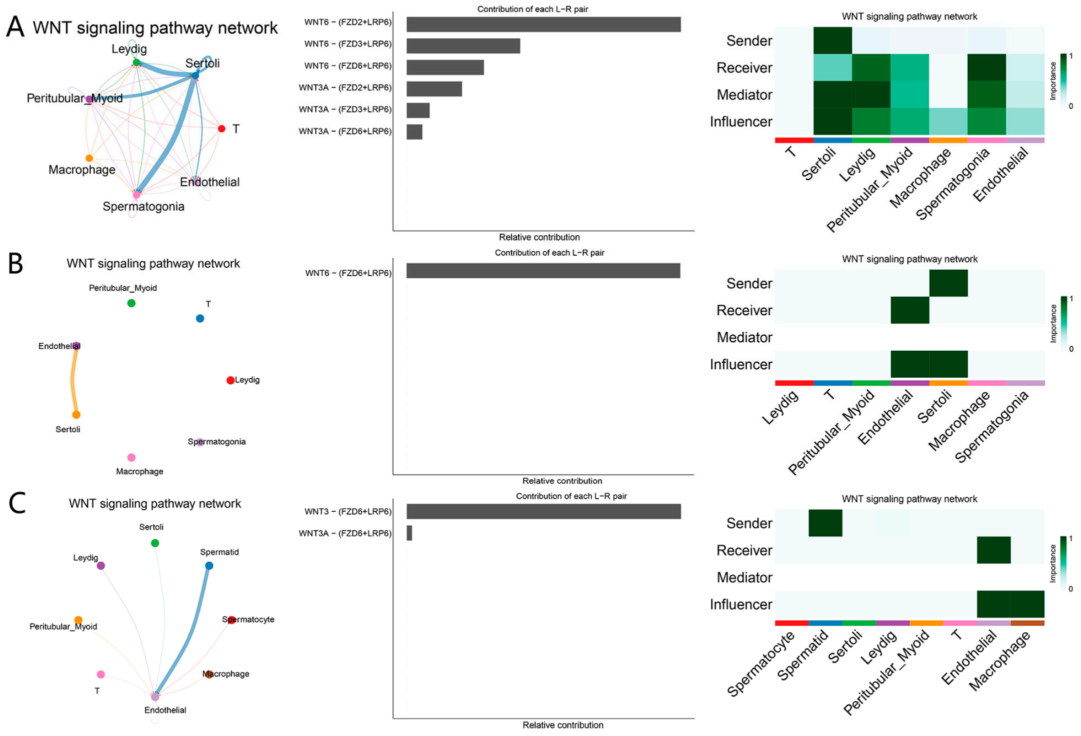

Figure S5:Communication modes of WNT signal paths in different periods.(A) The communication mode of BMP signaling pathway in YT stage. From left to right, (1)The WNT signal channel communication network diagram. (2)Contribution diagram of ligand-receptor to WNT signaling pathway. (3)The heat map shows the role of each cell population (sender, receiver, mediator, or influencer) in WNT signaling pathway. (B) The communication mode of WNT signaling pathway in FT stage.(C) The communication mode of WNT signaling pathway in AT stage.
