# Supplementary material for: Single-Cell RNA Sequencing Reveals Dynamic Transcriptional Landscape of Testicular Maturation in Dezhou Donkeys
Source: Animals (Basel). 2026 May 26;16(11):1621. doi: 10.3390/ani16111621 (PMC13255784; doi:10.3390/ani16111621)
Supplement: Supplementary file 1 [file animals-16-01621-s001.zip › Table S1.pdf]

Antibody sources

REAGENT or RESOURCE	SOURCE	IDENTIFIER
Rabbit anti-UTF1	Abcam	Cat #AB105090;
Rabbit anti-ID4	Abcam	Cat #AB20881;
Rabbit anti-TNP1	Abcam	Cat #AB73135;
Rabbit anti-SOX9	Abcam	Cat #AB5535;
Rabbit anti-ACTA2	Abcam	Cat #AB5694;
Rabbit anti-AMH	Proteintech	Cat No.23479-1-AP
Rabbit anti-ZMYND10	Proteintech	Cat No.14431-1-AP
Rabbit anti-AURKA	Orangene	Cat＃: TA329186
Rabbit anti-TPPP2	Proteintech	Cat No.13633-1-AP
Rabbit anti-STAR	Orangene	Cat＃: TA382081
Rabbit anti-MYH11	Abcam	Cat #: AB82541
Rabbit anti-TPM2	Orangene	Cat＃: TA382756
