# Supplementary material for: Single-Cell RNA Sequencing Reveals Dynamic Transcriptional Landscape of Testicular Maturation in Dezhou Donkeys
Source: Animals (Basel). 2026 May 26;16(11):1621. doi: 10.3390/ani16111621 (PMC13255784; doi:10.3390/ani16111621)
Supplement: Supplementary file 1 [file animals-16-01621-s001.zip › Table s2.pdf]

	Gene_Clusters
BANP	1
SLC7A5	2
KLHDC4	2
JPH3	2
ZCCHC14	2
MAP1LC3B	3
FBXO31	4
LOC106827906	5
MTHFSD	6
FOXF1	2
LOC106829176	5
EMC8	3
LOC106829243	5
LOC106829317	3
GINS2	7
GSE1	2
FAM92B	1
KIAA0513	1
ZDHHC7	4
USP10	2
KLHL36	5
COTL1	2
TLDC1	5
WFDC1	5
LOC106831119	1
ADAD2	5
TAF1C	4
DNAAF1	5
HSDL1	4
MBTPS1	2
OSGIN1	7
MLYCD	2
HSBP1	5
MPHOSPH6	2
SDR42E1	3
PLCG2	2
GAN	6
BCO1	1
GCSH	2
LOC106833328	8
ATMIN	4
CENPN	5
CMC2	5

CDYL2	6
DYNLRB2	5
WWOX	5
NUDT7	2
LOC106834642	1
SYCE1L	5
LOC106834828	5
CNTNAP4	1
LOC106828173	1
TERF2IP	4
KARS	9
GABARAPL2	5
TMEM231	6
TMEM170A	3
LOC106835903	6
CFDP1	2
LOC106836080	4
BCAR1	2
LOC106833713	7
ZNRF1	6
WDR59	6
FA2H	5
RFWD3	2
LOC106828792	1
NOCT	4
ELF2	5
MGARP	2
NDUFC1	5
NAA15	4
LOC106838485	1
MAML3	2
SCOC	4
CLGN	5
MGAT4D	5
ELMOD2	5
TBC1D9	2
RNF150	2
ZNF330	5
PDE5A	6
LOC106841444	6
LOC106841137	4
USP53	2
SEC24D	5
METTL14	2

LOC106842767	2
LOC106842379	5
UGT8	4
CAMK2D	4
ANK2	4
LARP7	3
ZC3H7A	2
RSL1D1	3
GSPT1	3
SNX29	7
CPPED1	5
LOC106840798	5
HDGFL1	5
SOX4	4
CDKAL1	4
E2F3	6
ID4	2
LRP12	1
DPYS	5
RIMS2	6
DCAF13	3
SLC25A32	5
CTHRC1	2
FZD6	2
ATP6V1C1	3
AZIN1	4
KLF10	2
ODF1	7
LOC106822938	7
UBR5	3
LOC106823358	2
NCALD	2
PLCZ1	7
CAPZA3	7
PLCH1	6
LOC106824265	2
SLC33A1	6
GMPS	2
LOC106824975	5
LOC106826008	2
SIGIRR	2
PTDSS2	5
RNH1	2
LOC106826502	1

ZKSCAN1	6
ZSCAN21	4
ZNF3	2
COPS6	3
MCM7	2
AP4M1	5
TAF6	2
STAG3	2
LOC106826829	8
LAMTOR4	2
VWA5A	5
ATP5G2	2
CALCOCO1	2
HOXC12	1
HOXC9	9
HOXC4	8
CBX5	6
HNRNPA1	2
COPZ1	4
ITGA5	5
GTSF1	5
LOC106828013	1
NCKAP1L	1
PDE1B	1
PPP1R1A	2
LOC106828356	7
CTNNB1	4
ULK4	1
LOC106828022	1
KCNMA1	1
LOC106828484	8
DLG5	2
POLR3A	4
RPS24	2
ZMIZ1	9
ZCCHC24	4
ANXA11	2
PLAC9	9
TMEM254	5
LOC106828660	9
TBC1D30	2
LOC106828693	6
SKIL	6
CLDN11	2

MYH15	1
KIAA1524	4
LOC106828744	1
RRBP1	9
DSTN	2
ARHGEF4	2
LOC106828832	2
PLEKHB2	4
LOC106828861	6
KLHL8	2
WDR33	3
SFT2D3	2
LIMS2	2
DPCR1	8
CCHCR1	5
TCF19	4
FOXP2	2
MDFIC	2
TFEC	1
TES	1
LOC106829424	5
CAV1	9
CAPZA2	2
ST7	6
ASZ1	2
CTTNBP2	2
LSM8	3
MORF4L2	3
MEMO1	8
DPY30	5
LOC106829528	5
FAM188A	2
LOC106829580	6
SNX5	2
BANF2	7
LOC106829746	2
LOC106829728	9
LOC106829729	9
FAM3C	2
ING3	6
TSPAN12	1
TM9SF2	5
CLYBL	7
ZIC5	2

PCCA	5
GGACT	2
TMTC4	2
ITGBL1	2
LOC106830030	5
TPP2	5
CCDC168	1
TEX30	5
LOC106830082	2
PRPF4B	6
FAM217A	5
LOC106830266	1
ECI2	5
LOC106830278	7
LOC106830318	2
LOC106830298	2
MAGED1	2
GSPT2	1
LOC106830356	2
LOC106830394	9
CT55	6
ZNF75D	2
LOC106830448	1
TMEM37	2
DBI	2
TFB2M	2
CNST	4
LOC106830541	1
IRX1	2
LOC106830714	5
PRCP	2
DDIAS	2
LOC106830607	3
PCF11	6
LOC106830624	6
ANKRD42	5
CCDC90B	5
TXNDC16	1
ERO1A	2
LOC106830797	1
MAP1S	2
FCHO1	2
RPL18A	2
SLC5A5	9

COLGALT1	2
LOC106830935	1
LOC106830945	1
LOC106830955	1
THSD1	9
LOC106830988	2
LOC106830977	6
LOC106831082	4
LOC106831104	4
LOC106831048	2
ATXN7L3B	2
CAPS2	1
GLIPR1L1	5
GLIPR1L2	5
GLIPR1	1
KRR1	3
PHLDA1	2
NAP1L1	5
BBS10	6
OSBPL8	2
ZDHHC17	6
CSRP2	2
E2F7	4
PAWR	3
LOC106831515	1
PPP1R12A	4
PTPRQ	5
LOC106831656	2
LOC106831673	1
LOC106831711	8
ANXA3	9
SLC9C1	5
LOC106831806	8
CD200	2
LOC106831880	2
LOC106831871	2
LIPA	2
CCDC112	4
PGGT1B	2
TRIM36	8
LOC106832184	7
YTHDC2	4
MCC	2
DCP2	4

REEP5	2
SRP19	3
APC	2
EPB41L4A	2
LOC106832316	5
NREP	2
STARD4	4
CAMK4	5
WDR36	4
SLC25A46	1
TMEM232	5
MAN2A1	6
PJA2	4
FER	6
LOC106832491	7
LOC106832498	5
LOC106832509	5
ASB12	4
LOC106832752	5
RB1CC1	5
FAM150A	8
LOC106832768	1
PCMTD1	2
ERGIC2	5
PLRG1	2
FGB	4
LRAT	1
RBM46	4
LOC106832973	1
MAP9	5
GUCY1A3	1
GUCY1B3	1
PDGFC	6
GRIA2	1
UBAC2	3
GPR18	1
LOC106833172	2
DEGS1	2
FBXO28	1
LOC106833202	2
ARL6IP1	2
SMG1	2
ADTRP	1
TMEM170B	1

SMIM13	2
ELOVL2	5
MAK	5
LOC106833388	2
PAK1IP1	2
LOC106833242	2
GCNT2	2
TFAP2A	1
LOC106833265	1
GSTO1	2
LOC106833484	7
ITPRIP	1
CFAP58	5
SORCS3	9
SORCS1	6
MCM4	2
UBE2V2	5
LOC106833608	5
LOC106833636	7
LOC106833622	5
LOC106833647	1
LOC106833684	7
LOC106833764	1
LOC106834188	7
FOCAD	6
MLLT3	2
ACER2	7
RPS6	2
DENND4C	4
PLIN2	2
HAUS6	2
RRAGA	3
SAXO1	8
SH3GL2	2
DIAPH3	1
LOC106834153	8
APEX2	2
NSDHL	9
PNMA5	6
LOC106834339	2
LOC106834348	4
USP51	9
PIN1	3
UBL5	3

FBXL12	2
LOC106834430	5
LOC106834435	6
NDUFA4	2
LOC106834443	2
DYM	5
LOC106834576	5
LOC106834569	2
ACAA2	9
MYO5B	1
CFAP53	5
MBD1	5
CXXC1	4
SKA1	5
MRO	2
ME2	9
ELAC1	2
SMAD4	2
MEX3C	1
LOC106834803	1
MBD2	2
POLI	5
STARD6	8
LOC106834825	1
LOC106834835	1
CCDC68	4
TCF4	6
TXNL1	5
WDR7	1
ONECUT2	8
FECH	5
NARS	2
LOC106835049	8
PURA	6
CXXC5	6
UBE2D2	5
LOC106835083	5
OTUD1	2
KIAA1217	2
LOC106835206	8
ARHGAP21	2
ENKUR	5
THNSL1	1
ANKRD26	6

SPAG17	5
WDR3	2
GDAP2	2
GTF2A1L	5
STON1	6
PPP1R21	6
FOXN2	2
TEX26	1
HSPH1	2
FRY	5
ZAR1L	6
BRCA2	2
N4BP2L2	5
PDS5B	4
STARD13	5
RFC3	3
CDK17	2
ELK3	2
LOC106835915	2
LOC106835966	4
LOC106835990	4
LUZP2	9
LOC106836067	1
LOC106836077	1
LOC106836085	2
TRPM6	7
ANXA1	2
ALDH1A1	2
ZFAND5	2
LOC106836044	5
LOC106836140	8
LOC106836148	5
ABHD17B	3
LOC106836214	1
LOC106836190	7
LOC106836223	1
LOC106836272	2
LOC106836340	2
LOC106836356	2
LOC106836370	2
LOC106836824	1
SLC44A5	1
ACADM	4
RABGGTB	2

MSH4	5
ASB17	8
PIGK	5
ZZZ3	6
USP33	2
FAM73A	1
NEXN	6
FUBP1	4
DNAJB4	7
PTGFR	1
LOC106836898	4
LOC106836908	5
MSN	2
LOC106836966	7
LOC106836973	1
LOC106836982	1
LOC106836989	3
LOC106837012	6
LOC106836995	3
LOC106837024	6
DDX26B	5
LOC106837097	1
TRIP4	6
KIAA0101	2
CSNK1G1	8
PPIB	2
SNX1	5
FAM96A	2
LOC106837231	7
HERC1	2
USP3	2
LOC106837232	1
CA12	1
APH1B	5
RAB8B	7
RPS27L	2
LACTB	5
TPM1	2
TLN2	5
LOC106837432	1
VPS13C	6
LOC106837466	2
RORA	2
ICE2	2

ANXA2	2
BNIP2	2
GTF2A2	5
GCNT3	1
LOC106837536	2
LOC106837710	4
LOC106837899	1
GBF1	5
NFKB2	7
PSD	4
FBXL15	5
CUEDC2	2
ACTR1A	5
SUFU	6
ARL3	5
SFXN2	1
WBP1L	2
BORCS7	3
LOC106838192	6
CNNM2	1
NT5C2	2
INA	6
PCGF6	2
LOC106838263	5
LYSMD2	2
DMXL2	2
LOC106838329	4
LOC106838351	1
AP4E1	4
LOC106838383	5
SPPL2A	4
TRPM7	2
USP50	8
USP8	3
GABPB1	2
SLC27A2	2
DTWD1	2
FAM227B	5
GALK2	5
COPS2	3
SECISBP2L	2
EID1	2
CEP152	1
FBN1	6

DUT	5
CTXN2	1
MYEF2	6
ISY1	2
CNBP	2
LOC106838840	1
APOE	9
LOC106838862	1
BCAM	9
EXPH5	1
ATE1	5
RAD21	2
UTP23	5
EIF3H	9
TRPS1	5
CNTNAP5	1
CHRDL1	1
PAK3	2
MAGED2	2
LOC106839298	1
LOC106839271	5
LOC106839265	5
LOC106839334	7
ZFP37	4
LOC106839352	6
CBFB	6
FAM96B	2
RRAD	2
NAE1	2
CCDC79	4
DYNC1LI2	5
CMTM4	7
CMTM3	2
CMTM2	5
LOC106839547	5
CKLF	5
TK2	6
BEAN1	2
FSCN3	7
ARF5	2
GCC1	7
ZNF800	3
LOC106839444	5
LOC106839695	1

LOC106839717	5
FKBP1B	2
SF3B6	2
PFN4	5
FAM228A	5
LOC106839802	9
ARL13A	5
TRMT2B	2
LOC106839894	2
LOC106839904	1
TMEM185A	2
LOC106839995	6
PPFIA4	9
TMEM183A	2
LOC106840148	7
LOC106840761	8
LOC106840164	1
ADIPOR1	2
KLHL12	4
RABIF	7
LOC106840779	8
KDM5B	3
SYT2	7
PPP1R12B	1
XRN2	4
KIZ	5
RALGAPA2	2
INSM1	2
CFAP61	1
CRNKL1	2
NAA20	5
RIN2	2
SLC24A3	8
LOC106840799	7
SCP2D1	7
DTD1	2
LOC106840414	2
SEC23B	4
POLR3F	5
DZANK1	5
ZNF133	4
CSRP2BP	2
LOC106840817	7
OVOL2	4

MGME1	2
TOM1L2	9
SREBF1	2
PEMT	8
MED9	5
NT5M	2
COPS3	5
FLCN	4
PLD6	3
MPRIP	2
LOC106840842	7
LOC106840872	2
LOC106840878	1
SCML2	4
HADHA	5
RAB10	2
KIF3C	2
ASXL2	6
DTNB	4
DNMT3A	2
POMC	8
EFR3B	8
DNAJC27	5
ADCY3	2
CENPO	6
PTRHD1	5
NCOA1	4
ITSN2	2
EGLN3	2
NPAS3	6
ARHGAP5	2
NUBPL	5
DTD2	2
HEATR5A	2
HECTD1	2
AP4S1	5
STRN3	4
COCH	2
SCFD1	3
G2E3	1
LOC106841507	2
LOC106841651	7
LOC106841661	2
LOC106841657	2

LOC106841685	2
EPHA1	2
TCAF1	2
LOC106842077	2
TPK1	2
CNTNAP2	2
CUL1	7
EZH2	4
LOC106842183	5
LOC106842173	5
PDIA4	4
ZNF786	4
ZNF425	2
MFSD14B	6
LAS1L	5
GTPBP6	2
SLC25A6	2
IDS	2
SLC25A40	5
CCDC148	5
PKP4	6
TANC1	2
WDSUB1	2
BAZ2B	5
LOC106843009	1
7-Mar	3
LOC106842977	2
RBMS1	5
TANK	4
PSMD14	3
TBR1	7
LOC106843171	5
IFIH1	2
GCA	2
FIGN	6
GRB14	2
COBLL1	2
SCN3A	5
GALNT3	5
LOC106843356	1
TTC21B	2
STK39	5
CERS6	6
NOSTRIN	9

SPC25	4
BBS5	5
KLHL41	1
FASTKD1	6
PPIG	2
CCDC173	5
PHOSPHO2	5
LOC106843606	1
KLHL23	2
SSB	2
METTL5	5
UBR3	4
LOC106842666	7
PCDH7	2
TMEM5	3
PPM1H	1
LOC106843789	6
ODF3	1
BET1L	5
RIC8A	2
SIRT3	6
PSMD13	2
IFITM5	2
CROT	2
LOC106844060	2
RERE	2
SLC45A1	2
ERRFI1	2
PARK7	2
TNFRSF9	6
PER3	1
VAMP3	4
CAMTA1	6
LOC106844433	7
LOC106844420	3
DNAJC11	1
THAP3	7
PHF13	4
KLHL21	4
ZBTB48	5
NOL9	2
PLEKHG5	4
TNFRSF25	6
ESPN	4

HES2	5
ACOT7	5
ICMT	2
RPL22	2
CHD5	1
NPHP4	6
LOC106844671	9
DFFB	2
CEP104	1
LRRC47	2
SMIM1	6
CCDC27	1
WRAP73	2
TPRG1L	5
ARHGEF16	2
ACTRT2	8
TTC34	1
LOC106844785	1
FAM213B	2
CDC40	2
LOC106844854	4
LOC106844873	5
LOC106844904	6
DDX53	8
LOC106844921	5
LOC106844946	4
LOC106844959	3
TNRC6C	4
TMC6	4
TMC8	4
SYNGR2	2
TK1	2
BIRC5	5
PGS1	4
DNAH17	5
CYTH1	1
USP36	2
TIMP2	9
CANT1	4
ENGASE	4
CBX2	2
CBX4	2
CCDC40	1
GAA	2

EIF4A3	2
SLC26A11	2
RNF213	4
ENDOV	6
RPTOR	4
CHMP6	3
BAIAP2	4
AATK	2
CEP131	2
ENTHD2	6
LOC106845759	2
SLC38A10	2
ACTG1	2
NPLOC4	4
OXLD1	5
CCDC137	7
ARL16	7
HGS	7
MRPL12	9
FAM195B	1
P4HB	2
ARHGDIA	2
ALYREF	2
ANAPC11	2
PCYT2	5
SIRT7	4
MAFG	9
PYCR1	2
ASPSCR1	5
STRA13	5
LRRC45	6
RAC3	2
LOC106846098	10
RFNG	2
GPS1	10
DUS1L	2
LOC106846150	6
SLC16A3	2
CSNK1D	7
TEX19	2
OGFOD3	9
HEXDC	2
LOC106846259	2
NARF	9

FOXK2	1
WDR45B	4
RAB40B	9
FN3KRP	2
FN3K	2
TBCD	5
B3GNTL1	2
METRNL	2
LOC106846413	2
LOC106846417	4
LOC106846435	7
LOC106846442	8
MIER2	4
PLPP2	2
LOC106846481	2
LOC106846506	1
LOC106846529	1
LOC106846562	2
CCNG2	2
CCNI	9
SEPT11	4
SHROOM3	4
CCDC158	5
STBD1	2
SCARB2	2
NUP54	6
ART3	3
CXCL9	2
SDAD1	3
NAAA	2
PPEF2	7
USO1	2
LOC106846955	2
LOC106847010	7
ZNF558	6
ADAMTS5	6
N6AMT1	2
LTN1	1
RWDD2B	1
USP16	3
CCT8	3
MAP3K7CL	5
BACH1	2
TIAM1	2

LOC106847297	1
LOC106847304	5
SOD1	2
SCAF4	2
LOC106847492	5
KLK12	1
LOC106847610	5
LOC106847602	7
LOC106847618	1
LOC106847667	6
LOC106847653	2
LOC106847686	5
LOC106847678	2
LOC106847698	1
CHD1	4
RGMB	2
RIOK2	2
LNPEP	4
ERAP1	2
CAST	7
ELL2	2
RHOBTB3	2
SPATA9	1
RFESD	5
ARSK	1
TTC37	6
FAM81B	8
SLF1	4
KIAA0825	1
LOC106848287	1
LOC106848292	5
LOC106848366	5
LOC106848552	7
PRPS2	2
MSL3	2
LOC106848674	4
LOC106822196	8
GDPD1	7
SMG8	2
PRR11	1
SKA2	5
TRIM37	5
PPM1E	1
RAD51C	5

TEX14	5
LOC106821906	6
MTMR4	6
HSF5	5
RNF43	1
SUPT4H1	2
BZRAP1	1
MKS1	5
DYNLL2	7
SRSF1	1
VEZF1	9
LOC106822125	1
MRPS23	2
CCDC182	8
MSI2	6
LOC106822238	2
LOC106822285	2
LOC106822294	2
NEU1	2
EHMT2	5
LOC106822359	1
LOC106822373	2
LOC106822420	2
LOC106822768	7
LPCAT2	1
MMP2	2
IRX5	4
LOC106822498	2
IRX3	2
FTO	9
RPGRIP1L	6
AKTIP	9
RBL2	5
CHD9	2
TOX3	2
SALL1	2
CYLD	2
LOC106822826	1
LOC106822898	5
LOC106823026	5
LOC106823033	2
STK35	4
SNRPB	3
LOC106823097	6

NOP56	2
IDH3B	3
MAEL	3
LOC106823148	8
TADA1	2
POGK	2
C4BPB	3
LOC106822945	5
LOC106823195	1
LOC106823255	7
FCAMR	7
FCMR	1
LOC106823339	9
DYRK3	5
EIF2D	6
RASSF5	4
SRGAP2	9
FAM72A	2
LOC106823454	2
SLC41A1	4
RAB29	6
NUCKS1	4
MFSD4	2
CDK18	2
LEMD1	7
KLHDC8A	1
NUAK2	1
TMCC2	2
DSTYK	4
RBBP5	2
TMEM81	1
LRRN2	1
LOC106823778	1
MDM4	5
PIK3C2B	6
LOC106823833	6
PLEKHA6	1
REN	3
ETNK2	2
SOX13	6
ZC3H11A	6
LOC106824067	1
ATP2B4	2
LOC106824071	7

BTG2	9
LOC106824167	2
LOC106824201	4
LOC106824206	5
LOC106824225	1
LOC106824217	1
LOC106824251	9
LOC106824266	1
NRG1	2
LOC106824642	1
WRN	6
PURG	1
TEX15	3
PPP2CB	5
LOC106824446	1
UBXN8	9
GSR	2
GTF2E2	3
RBPMS	3
DCTN6	4
MBOAT4	1
LEPROTL1	2
SARAF	2
LOC106824577	7
DUSP4	7
TNKS	8
PPP1R3B	1
ERI1	3
MFHAS1	2
WNT5B	1
FBXL14	1
LOC106824344	1
LOC106824724	2
LOC106824728	4
LOC106824745	2
LOC106824771	2
LOC106824761	2
LOC106824780	5
MRPS30	5
PAIP1	2
LOC106824967	2
LOC106824976	1
HMGCS1	3
NIM1K	1

ZNF131	4
CTSZ	2
NELFCD	2
LOC106825087	9
LOC106825122	6
NPEPL1	2
STX16	1
VAPB	2
RAB22A	4
LOC106825186	5
ANKRD60	8
LOC106825225	5
LOC106825884	2
PMEPA1	2
LOC106825251	1
RAE1	5
SPO11	1
BMP7	2
TFAP2C	2
FAM209B	1
RTFDC1	3
CSTF1	3
AURKA	5
FAM210B	2
DOK5	2
PFDN4	5
LOC106825434	2
BCAS1	2
ZNF217	4
TSHZ2	2
LOC106825925	1
ZFP64	9
SALL4	2
ATP9A	9
NFATC2	7
MOCS3	6
DPM1	4
ADNP	6
BCAS4	2
PARD6B	2
PTPN1	6
LOC106825701	1
LOC106825706	7
UBE2V1	5

SNAI1	2
RNF114	4
SPATA2	1
SLC9A8	1
B4GALT5	4
PTGIS	2
LOC106825788	2
ZNFX1	6
DDX27	2
STAU1	2
CSE1L	6
ARFGEF2	2
PREX1	2
FBXO11	4
MSH6	5
KCNK12	1
MSH2	3
EPCAM	4
LOC106826398	1
LOC106826409	1
CALM2	2
LOC106826027	5
TTC7A	7
MCFD2	4
SOCS5	6
CRIPT	4
PIGF	5
RHOQ	2
ATP6V1E2	5
TMEM247	1
EPAS1	6
PRKCE	7
SRBD1	2
LOC106826423	8
SIX2	2
SIX3	2
CAMKMT	1
PREPL	6
SLC3A1	6
PPM1B	5
LRPPRC	2
DYNC2LI1	5
PLEKHH2	2
THADA	1

ZFP36L2	2
MTA3	2
LOC106826494	2
YWHAH	2
DEPDC5	8
PRR14L	6
PISD	3
EIF4ENIF1	2
DRG1	2
PATZ1	9
PIK3IP1	6
RNF185	6
INPP5J	1
LOC106826967	2
SMTN	7
LOC106826625	7
MORC2	5
OSBP2	7
LOC106826998	10
DUSP18	1
SLC35E4	2
LOC106827052	5
LOC106827070	8
PES1	3
GAL3ST1	5
MTFP1	9
SEC14L2	8
CCDC157	10
SF3A1	5
TBC1D10A	5
GATSL3	4
HORMAD2	5
LOC106827226	5
MTMR3	2
ASCC2	5
LOC106827325	3
ZMAT5	5
NF2	6
THOC5	2
NEFH	2
AP1B1	5
GAS2L1	9
EWSR1	3
RHBDD3	1

EMID1	2
LOC106827545	1
ZNRF3	7
XBP1	4
CCDC117	2
HSCB	5
CHEK2	2
TTC28	1
PITPNB	6
LOC106827769	1
LOC106827803	6
LOC106827813	5
LOC106827815	1
LOC106827816	5
LOC106827818	1
TLE1	2
TLE4	6
LOC106827823	2
CEP78	3
LOC106827825	7
PCSK5	4
LOC106827827	2
LOC106827847	1
LOC106827850	6
IRS1	4
LOC106827855	8
RHBDD1	6
COL4A4	1
MFF	10
LOC106827863	8
AGFG1	5
CCL20	1
DAW1	5
PID1	2
DNER	6
TRIP12	5
FBXO36	8
SLC16A14	2
SP110	2
LOC106827877	2
LOC106827879	9
CAB39	3
ITM2C	5
SPATA3	7

PSMD1	3
HTR2B	1
ARMC9	1
LOC106827898	2
LOC106827899	1
LOC106827903	7
LOC106827904	1
EOGT	4
TMF1	5
UBA3	3
ARL6IP5	2
FRMD4B	2
MITF	2
FOXP1	2
LOC106827932	1
EIF4E3	2
GPR27	2
PROK2	4
LOC106827941	10
RYBP	5
SHQ1	6
PPP4R2	4
LOC106827948	9
LOC106827946	6
ZNF32	2
LOC106827958	5
UBR4	3
IFFO2	1
ALDH4A1	2
ACTL8	4
ARHGEF10L	2
RCC2	2
PADI6	1
PADI2	1
SDHB	5
ATP13A2	2
CROCC	2
NECAP2	7
SPATA21	8
SZRD1	4
FBXO42	2
LOC106827995	7
ZBTB17	5
SPEN	3

FBLIM1	1
PLEKHM2	1
DNAJC16	6
CASP9	6
EFHD2	7
FHAD1	1
TMEM51	2
KAZN	2
LOC106828021	1
PRDM2	6
LOC106827967	6
PDPN	2
LOC106828024	5
LOC106828030	1
LOC106828032	3
LOC106828036	1
LOC106828045	5
TMEM117	4
TWF1	4
PUS7L	2
BMP2K	9
PAQR3	7
NAA11	2
GK2	5
ANTXR2	2
LOC106828066	5
RASGEF1B	4
LOC106828074	6
LOC106828078	3
LOC106828083	5
LOC106828084	2
DR1	5
FNBP1L	2
BCAR3	2
DNTTIP2	2
GCLM	6
ABCA4	1
ARHGAP29	6
ABCD3	2
LOC106828107	8
SLC44A3	6
CNN3	6
ALG14	2
TMEM56	7

RWDD3	2
PTBP2	4
DPYD	2
LOC106828123	4
LOC106828128	5
LOC106828129	1
LOC106828132	5
LOC106828131	1
LOC106828133	6
LOC106828134	2
LOC106828145	5
NRP2	4
INO80D	2
NDUFS1	4
EEF1B2	2
DYTN	1
MDH1B	5
FASTKD2	5
KLF7	9
CREB1	3
LOC106828161	1
METTL21A	5
CCNYL1	7
LOC106828168	1
PLEKHM3	1
IDH1	2
PIKFYVE	4
LOC106828184	8
LOC106828183	7
TRIML2	2
TRIML1	1
FRG1	6
ASAH1	2
PCM1	3
LOC106828206	1
MTUS1	5
SLC7A2	6
MTMR7	1
VPS37A	6
CNOT7	3
ZDHHC2	1
MICU3	6
LOC106828218	2
GAS8	5

TUBB3	9
TCF25	3
LOC106828227	1
SPIRE2	5
FANCA	4
ZNF276	1
VPS9D1	4
CDK10	5
CHMP1A	9
RPL13	2
SPG7	4
LOC106828239	5
ANKRD11	5
ACSF3	3
TRAPPC2L	5
GALNS	5
APRT	2
CDT1	2
CTU2	4
RNF166	4
MVD	2
LOC106828257	2
ZC3H18	6
DUSP11	5
TPRKB	3
ALMS1	1
EGR4	2
CCT7	5
PRADC1	5
SMYD5	9
RAB11FIP5	7
SPR	2
EXOC6B	4
LOC106828299	2
DYSF	2
ZNF638	2
PAIP2B	1
NAGK	3
TEX261	3
ANKRD53	8
VAX2	2
ADD2	1
TGFA	4
FAM136A	2

SNRPG	2
PCYOX1	2
TIA1	4
LOC106828317	2
PCBP1	2
MXD1	1
SNRNP27	3
GMCL1	5
ANXA4	2
AAK1	4
NFU1	5
GFPT1	5
ARHGAP25	6
APLF	5
FBXO48	5
PPP3R1	4
PNO1	4
WDR92	4
C1D	5
ETAA1	4
SPRED2	2
ACTR2	2
RAB1A	9
CEP68	6
SLC1A4	1
SERTAD2	6
AFTPH	8
LGALSL	9
PELI1	6
LOC106828366	1
VPS54	2
UGP2	2
MDH1	2
WDPCP	1
EHBP1	6
TMEM17	1
B3GNT2	2
VBP1	3
CMC4	5
FUNDC2	7
SMIM9	1
MPP1	2
DKC1	2
LOC106828439	7

LOC106828441	1
ETV3	4
ARHGEF11	2
LRRC71	5
NTRK1	2
PRCC	6
HDGF	5
MRPL24	5
RRNAD1	4
ISG20L2	7
CRABP2	2
NES	2
BCAN	2
HAPLN2	7
GPATCH4	5
APOA1BP	2
IQGAP3	1
MEF2D	4
LOC106828405	8
TSACC	5
CCT3	5
SMG5	1
LOC106828470	5
SLC25A44	2
LMNA	2
MEX3A	6
LAMTOR2	3
UBQLN4	2
LOC106828407	5
SSR2	2
ARHGEF2	5
KIAA0907	1
RIT1	2
LOC106828483	4
MSTO1	5
DAP3	4
ASH1L	6
FDPS	2
CLK2	6
SCAMP3	2
GBA	2
MTX1	7
THBS3	8
MUC1	1

TRIM46	8
KRTCAP2	2
DPM3	2
SLC50A1	6
EFNA1	2
EFNA3	2
EFNA4	2
ADAM15	6
DCST1	1
DCST2	1
FLAD1	1
CKS1B	2
SHC1	2
PYGO2	2
PBXIP1	2
LOC106828520	7
PMVK	4
KCNN3	8
ADAR	2
UBE2Q1	3
TDRD10	2
LOC106828536	2
FGF9	3
MICU2	3
ZDHHC20	4
TCEAL8	2
MRPL57	2
SKA3	5
SAP18	5
LATS2	6
XPO4	4
N6AMT2	2
LOC106828567	5
IFT88	5
CRYL1	2
LOC106828544	2
TRIM33	5
OLFML3	8
HIPK1	6
DCLRE1B	1
AP4B1	1
LOC106828547	1
RSBN1	6
PHTF1	5

MAGI3	4
LRIG2	4
SLC16A1	1
LOC106828584	5
FAM19A3	1
PPM1J	1
RHOC	2
MOV10	4
CAPZA1	3
ST7L	1
WNT2B	6
CTTNBP2NL	6
KCND3	7
DDX20	5
RAP1A	4
ADORA3	5
ATP5F1	3
WDR77	6
PIFO	5
DENND2D	6
CEPT1	2
LOC106828619	1
LOC106828620	5
RBBP8	2
CABLES1	1
RIOK3	5
LOC106828637	2
NPC1	2
ANKRD29	2
PSMG2	1
PTPN2	2
SEH1L	2
CEP192	4
LDLRAD4	9
FAM210A	4
RNMT	5
NAPG	2
APCDD1	4
VAPA	3
TXNDC2	7
LOC106828653	1
PPP4R1	7
RALBP1	2
TWSG1	9

ANKRD12	2
NDUFV2	2
LOC106828662	6
LOC106828665	6
BASP1	2
MYO10	2
FAM134B	7
ZNF622	2
11-Mar	5
FBXL7	6
LOC106828680	7
COL22A1	8
FAM135B	1
KLK15	2
NUFIP2	4
LOC106828692	2
UBR2	6
TBCC	6
GLTSCR1L	1
RPL7L1	2
LOC106828712	6
CNPY3	6
PEX6	6
PPP2R5D	1
MEA1	10
KLHDC3	5
RRP36	2
CUL7	7
MRPL2	10
KLC4	5
PTK7	6
SRF	7
CUL9	2
CRIP3	5
ZNF318	6
TJAP1	4
LRRC73	5
YIPF3	5
POLR1C	3
XPO5	6
POLH	1
GTPBP2	8
MAD2L1BP	10
RSPH9	5

MRPS18A	5
VEGFA	2
MRPL14	5
CAPN11	5
SLC29A1	2
HSP90AB1	2
SLC35B2	2
TCTE1	5
AARS2	6
SPATS1	5
CDC5L	3
LOC106828778	1
SUPT3H	2
RUNX2	8
CLIC5	8
LOC106828702	2
ENPP4	4
ENPP5	1
RCAN2	6
LOC106828769	1
SLC25A27	1
TDRD6	5
PLA2G7	9
MEP1A	7
ADGRF5	2
TNFRSF21	6
LOC106828811	5
NABP1	1
LOC106828791	4
MYO1B	2
STAT4	5
STAT1	2
GLS	2
LOC106828798	7
NAB1	2
MFSD6	6
INPP1	1
HIBCH	2
LOC106828809	7
LOC106828787	5
IKBKG	2
LOC106828819	7
LOC106828829	7
KIAA0430	4

NDE1	2
MYH11	2
FOPNL	3
ABCC1	2
LOC106828821	2
MFSD1	2
LOC106828839	5
SLF2	2
MRPL43	3
LZTS2	2
KAZALD1	6
TLX1	2
BTRC	5
POLL	5
DPCD	5
FBXW4	7
LOC106828856	1
NPM3	2
MGEA5	2
KCNIP2	7
LOC106828860	7
LDB1	2
PPRC1	2
NOLC1	2
LOC106828867	5
LOC106828825	2
ACRC	2
OGT	2
TAF1	6
LOC106828872	2
NONO	2
ZMYM3	6
MED12	2
LOC106828881	7
SLC7A3	9
ADAM2	5
ADAM18	5
IDO2	7
LOC106828976	7
ZMAT4	1
SFRP1	2
GOLGA7	2
GINS4	2
GPAT4	6

ANK1	1
KAT6A	5
LOC106828953	1
AP3M2	7
IKBKB	2
POLB	7
DKK4	5
VDAC3	5
SLC20A2	4
SMIM19	2
THAP1	5
RNF170	2
HOOK3	2
FNTA	3
HGSNAT	9
INTS10	2
PRKCDBP	2
CNGA4	1
FAM160A2	5
LOC106828974	7
LOC106829009	5
ZSCAN20	1
PHC2	10
ZNF362	7
AZIN2	7
LOC106829032	5
AK2	5
RNF19B	5
TMEM54	1
FNDC5	2
S100PBP	5
YARS	6
KIAA1522	2
RBBP4	3
ZBTB8OS	2
ZBTB8A	2
BSDC1	7
TSSK3	7
FAM229A	8
MARCKSL1	9
HDAC1	3
LOC106829053	2
EIF3I	2
IQCC	5

CCDC28B	8
TXLNA	2
KPNA6	6
TMEM39B	2
KHDRBS1	3
PTP4A2	2
ADGRB2	6
COL16A1	6
PEF1	2
SERINC2	2
LOC106829072	1
FABP3	5
ZCCHC17	4
SNRNP40	2
PUM1	4
LAPTM5	2
LOC106829021	7
KDM8	6
NSMCE1	5
LOC106829082	1
IL4R	2
GTF3C1	6
KIAA0556	1
GSG1L	2
XPO6	4
SPNS1	4
NFATC2IP	3
CD19	8
RABEP2	4
LOC106829104	1
SH2B1	2
TUFM	2
ATXN2L	5
LOC106829108	5
LOC106829110	5
ZHX1	8
LOC106829114	2
LOC106829115	1
TBC1D31	2
DERL1	3
PHF14	4
LOC106829135	9
THSD7A	2
TMEM106B	2

LOC106829124	1
LOC106829126	6
MRAP2	1
CEP162	6
LOC106829136	7
NT5E	2
SNX14	4
SYNCRIP	2
LOC106829134	2
LOC106829137	5
LOC106829139	2
USP38	4
SMARCA5	2
LOC106829160	2
HHIP	9
ANAPC10	5
ABCE1	3
OTUD4	6
SMAD1	2
MMAA	4
LOC106829167	5
ZNF827	9
LSM6	2
SLC10A7	5
TTC29	5
LOC106829174	1
TMEM184C	4
PRMT9	6
ARHGAP10	2
NR3C2	2
LOC106829184	6
LOC106829186	3
MAATS1	5
GSK3B	4
KCTD15	2
LSM14A	4
KIAA0355	4
GPI	9
PDCD2L	5
UBA2	5
WTIP	2
LOC106829201	8
LOC106829208	4
ZNF181	1

LOC106829286	2
LOC106829215	1
LOC106829214	7
LOC106829212	7
LOC106829216	1
ZNF30	2
GRAMD1A	2
FXYD5	9
LOC106829228	7
LSR	2
USF2	2
KRTDAP	1
GAPDHS	7
TMEM147	9
LOC106829241	5
LOC106829289	5
LOC106829290	8
LOC106829291	7
LOC106829293	7
LOC106829294	1
HAUS5	6
RBM42	3
LOC106829247	2
ZBTB32	5
KMT2B	4
IGFLR1	4
U2AF1L4	5
PSENEN	5
LIN37	5
HSPB6	5
ARHGAP33	6
LOC106829257	1
APLP1	1
HCST	2
SDHAF1	1
LOC106829265	6
CLIP3	2
LOC106829269	5
WDR62	5
POLR2I	2
TBCB	5
CAPNS1	5
LOC106829276	8
ZNF565	6

ZNF146	1
LOC106829323	1
DNAJC19	2
FXR1	5
CCDC39	5
TTC14	2
LOC106829303	6
USP13	2
NDUFB5	2
MRPL47	3
ACTL6A	2
GNB4	2
MFN1	4
ZNF639	2
PIK3CA	4
ZMAT3	2
TBL1XR1	2
LOC106829327	4
VGLL4	2
TAMM41	3
TIMP4	7
PPARG	4
LOC106829342	5
TSEN2	2
MKRN2	7
RAF1	4
CAND2	2
RPL32	2
EFCAB12	1
MBD4	4
IFT122	5
LOC106829384	5
PLXND1	2
TMCC1	4
TRH	2
RBSN	1
MRPS25	2
NR2C2	6
LOC106829360	3
FGD5	2
LOC106829362	1
CCDC174	5
LSM3	3
XPC	2

TMEM43	4
CHCHD4	2
HDAC11	7
LOC106829337	2
SYCE1	5
UBAP2	3
LOC106829395	7
ST6GAL2	6
LOC106829398	2
PREX2	4
CPA6	7
ARFGEF1	4
CSPP1	1
COPS5	3
PPP1R42	5
LOC106829407	5
MCMDC2	1
SGK3	2
VCPIP1	3
MYBL1	5
RRS1	2
LOC106829419	5
LOC106829430	2
ZIC4	7
AGTR1	8
GYG1	5
HLTF	3
HPS3	2
TM4SF1	9
WWTR1	2
LOC106829449	2
RNF13	5
TSC22D2	2
SERP1	10
EIF2A	5
LOC106829426	2
ERICH6	5
SIAH2	2
CLRN1	8
LOC106829457	6
LEMD3	4
MSRB3	8
LOC106829480	1
LLPH	2

TMBIM4	5
GAP43	2
ZBTB20	4
TIGIT	1
QTRTD1	7
CCDC191	5
ZDHHC23	4
GRAMD1C	5
ATP6V1A	4
NAA50	2
USF3	2
SIDT1	8
SPICE1	5
CFAP44	5
BOC	6
LOC106829506	8
LOC106829507	3
GTPBP8	2
CCDC80	2
SLC35A5	7
ATG3	3
KIAA1210	2
LOC106829521	6
SNCA	2
MMRN1	1
CCSER1	2
GRID2	1
SMARCAD1	4
MARK1	3
LOC106829574	6
RAB3GAP2	6
IARS2	2
BPNT1	4
MIA3	6
AIDA	3
BROX	5
SUSD4	2
CCDC185	1
TP53BP2	2
LOC106829557	2
NT5C3A	2
RP9	4
BBS9	1
NPSR1	7

DPY19L1	6
DPY19L2	5
HERPUD2	7
LOC106829579	1
OPRK1	2
ATP6V1H	2
TCEA1	3
LYPLA1	7
LOC106829594	4
LOC106829589	6
NCAPG	5
DCAF16	5
FAM184B	5
MED28	5
LAP3	2
QDPR	2
LDB2	2
TAPT1	10
PROM1	1
LOC106829608	7
FAM174A	6
ST8SIA4	1
KBTBD11	1
ARHGEF10	4
PACRGL	3
ADGRA3	2
LOC106829643	5
DNAJC15	5
ENOX1	1
CCDC122	5
LACC1	2
LOC106829654	2
TSC22D1	5
NUFIP1	2
GPALPP1	2
GTF2F2	4
TPT1	2
SLC25A30	7
ERICH6B	1
SPERT	7
ZC3H13	2
LCP1	6
LRRC63	1
KIAA0226L	6

LRCH1	2
ESD	2
SUCLA2	5
NUDT15	6
MED4	3
ITM2B	5
RB1	6
RCBTB2	2
FNDC3A	5
CDADC1	1
CAB39L	5
SETDB2	4
PHF11	3
RCBTB1	4
SEC61G	3
LOC106829700	1
TRIO	7
FAM105A	2
OTULIN	2
ANKH	6
LOC106829707	1
ZNF322	6
LOC106829722	1
LOC106829710	1
LOC106829711	4
LOC106829712	1
LOC106829716	4
FAXC	2
COQ3	1
LOC106829738	1
PNISR	6
LOC106829743	5
LOC106829742	7
LOC106829744	3
OSBPL3	2
MPP6	10
STK31	4
WASF1	2
FIG4	2
AK9	5
ZBTB24	6
SMPD2	8
PPIL6	5
LOC106829760	2

CEP57L1	5
SESN1	4
ARMC2	5
LOC106829765	4
FOXO3	2
LACE1	5
SNX3	3
OSTM1	5
PITX2	2
GAR1	2
CFI	2
PLA2G12A	2
CASP6	2
CCDC109B	5
SEC24B	5
OSTC	3
RPL34	2
LEF1	2
HADH	2
LOC106829787	2
SGMS2	1
LOC106829818	6
PAPSS1	2
AIMP1	2
TBCK	4
NPNT	2
CNGB3	1
CPNE3	2
RMDN1	3
WWP1	3
CA2	7
LOC106829855	2
LRRCC1	5
PTPRZ1	4
AASS	1
FEZF1	5
LOC106829859	5
CADPS2	1
RNF133	7
RNF148	1
IQUB	5
NDUFA5	3
ASB15	1
WASL	2

LOC106829838	5
SPAM1	5
LOC106829873	5
POT1	6
SCLT1	5
JADE1	5
PGRMC2	9
LARP1B	10
MFSD8	6
PLK4	10
HSPA4L	10
SLC25A31	3
INTU	6
THOC2	4
XIAP	2
STAG2	2
U2AF1	3
PKNOX1	5
NDUFV3	10
PDE9A	2
SLC37A1	5
RSPH1	5
LOC106829934	1
ZBTB21	2
C2CD2	1
TMPRSS2	5
FAM3B	9
BACE2	2
LOC106829947	5
LOC106829961	1
SH3BGR	6
LCA5L	5
WRB	2
HMGN1	4
LOC106829922	6
VRK2	3
FANCL	2
BCL11A	2
PAPOLG	4
REL	2
PUS10	6
LOC106829971	5
PSPC1	2
LOC106829976	6

MPHOSPH8	3
CENPJ	6
RNF17	5
MTPN	2
LOC106829994	2
NUP205	6
CNOT4	5
STRA8	2
LOC106829988	5
LOC106830005	1
AGBL3	1
LOC106830006	1
CALD1	2
BPGM	10
LOC106830009	1
AKR1B1	2
SLC35B4	1
LRGUK	1
LOC106830013	1
PDLIM5	4
IFIT5	2
LOC106830028	5
KIF20B	3
HTR7	5
RPP30	2
PCGF5	5
LOC106830034	1
HECTD2	6
PPP1R3C	2
TNKS2	4
BTAF1	5
CPEB3	9
ADAM10	4
ALDH1A2	5
POLR2M	2
MYZAP	2
CGNL1	2
TCF12	4
FBXO3	6
CD59	7
LOC106830059	5
HIPK3	2
TENM3	2
DCTD	2

WWC2	4
LOC106830070	7
CDKN2AIP	8
ING2	5
RWDD4	2
TRAPPC11	6
STOX2	9
IRF2	2
CASP3	2
PRIMPOL	2
CENPU	3
ACSL1	2
SLC25A4	2
CFAP97	10
LOC106830094	5
SNX25	2
LRP2BP	5
ANKRD37	2
UFSP2	5
LOC106830092	5
RTTN	1
CD226	1
UBQLN3	1
LOC106830213	8
UBQLNL	7
LOC106830220	4
ABCA5	3
SOX9	2
SLC39A11	5
COG1	4
FAM104A	5
LOC106830246	5
SDK2	1
RPL38	2
DNAI2	5
KIF19	6
GPRC5C	4
SLC9A3R1	2
NAT9	4
GRIN2C	6
FDXR	3
FADS6	4
CDR2L	5
ICT1	2

ATP5H	2
KCTD2	2
LOC106830285	7
NT5C	2
HN1	5
SUMO2	2
NUP85	2
GGA3	2
MRPS7	9
MIF4GD	5
SLC25A19	5
GRB2	2
TMEM94	5
TSEN54	2
RECQL5	4
SMIM6	1
SAP30BP	3
ITGB4	2
GALK1	9
H3F3B	3
UNK	6
WBP2	5
TRIM47	2
TRIM65	2
MRPL38	3
FBF1	1
ACOX1	6
TEN1	1
SRP68	3
EXOC7	3
FOXJ1	1
UBALD2	7
QRICH2	1
PRPSAP1	3
UBE2O	4
CYGB	6
LOC106830338	6
LOC106830337	2
ST6GALNAC2	7
LOC106830339	7
JMJD6	4
METTL23	4
SRSF2	3
MFSD11	5

LOC106830346	6
SEC14L1	7
SEPT9	2
STXBP6	6
LOC106830421	5
LOC106830429	5
LOC106830430	5
TMEM74	2
NUDCD1	2
ENY2	2
EBAG9	5
SYBU	4
LOC106830444	5
EPRS	4
LOC106830427	4
HHIPL2	6
LOC106830453	2
LOC106830457	7
ZGRF1	6
TIFA	2
AP1AR	2
LOC106830487	1
LOC106830498	7
LOC106830461	5
LOC106830490	2
SMC2	4
LOC106830492	5
LOC106830494	6
ABCA1	9
ZMYM1	6
SFPQ	3
ZMYM4	2
KIAA0319L	6
LOC106830526	1
NCDN	4
PSMB2	5
CLSPN	2
LOC106830530	2
LOC106830533	7
AGO3	2
TEKT2	5
ADPRHL2	2
TRAPPC3	2
MAP7D1	2

THRAP3	2
SH3D21	7
STK40	8
LSM10	5
OSCP1	5
MRPS15	2
MEAF6	5
SNIP1	2
DNALI1	5
GNL2	3
CDCA8	3
LOC106830564	2
YRDC	2
MTF1	1
INPP5B	6
SF3A3	5
FHL3	2
UTP11L	2
POU3F1	2
RRAGC	4
MYCBP	3
LOC106830576	1
AKIRIN1	9
NDUFS5	2
MACF1	2
LOC106830582	7
OXCT2	7
PPIE	2
HPCAL4	1
HEYL	1
LOC106830588	2
LOC106830589	1
ATP11B	4
CEP89	1
FAAP24	9
RHPN2	2
LOC106830594	6
LETM1	5
FGFR3	2
TACC3	5
TMEM129	4
SLBP	8
UVSSA	5
MAEA	2

CTBP1	9
MICU1	1
DNAJB12	5
ANAPC16	3
ASCC1	3
PSAP	2
LOC106830630	2
UNC5B	9
PCBD1	2
SGPL1	2
TBATA	1
PALD1	2
EIF4EBP2	2
PPA1	2
LOC106830662	3
TMEM89	5
SLC26A6	9
NCKIPSD	1
IP6K2	3
PRKAR2A	7
SLC25A20	2
ARIH2OS	5
ARIH2	5
P4HTM	1
WDR6	6
DALRD3	5
NDUFAF3	10
IMPDH2	6
QRICH1	3
QARS	7
USP19	6
LAMB2	2
CCDC71	1
LOC106830758	1
CCDC36	5
LOC106830685	5
USP4	5
GPX1	2
RHOA	2
TCTA	5
AMT	1
NICN1	5
DAG1	2
BSN	1

APEH	2
RNF123	6
GMPPB	10
IP6K1	6
FAM212A	10
TRAIP	2
CAMKV	1
LOC106830712	7
MON1A	8
RBM6	4
RBM5	6
GNAI2	2
IFRD2	4
NAT6	7
LOC106830729	2
TUSC2	2
RASSF1	6
ZMYND10	5
NPRL2	9
LOC106830736	1
TMEM115	4
CACNA2D2	4
DOCK3	2
MANF	5
RBM15B	2
VPRBP	6
RAD54L2	6
TEX264	2
IQCF6	7
LOC106830750	7
IQCF3	7
LOC106830658	7
LOC106830763	7
LOC106830764	7
LOC106830751	7
RRP9	2
PARP3	2
PCBP4	2
LOC106830781	1
RC3H2	6
ZBTB6	2
ZBTB26	6
RABGAP1	4
STRBP	5

LHX2	5
LOC106830851	7
LOC106830811	1
NEK6	2
PSMB7	5
NR6A1	1
OLFML2A	2
WDR38	8
RPL35	2
ARPC5L	2
GOLGA1	5
SCAI	6
PPP6C	5
RABEPK	5
HSPA5	5
GAPVD1	2
MAPKAP1	3
PBX3	2
LOC106830854	7
DNAJB9	5
THAP5	4
ELMO1	1
ANLN	6
KIAA0895	5
EEPD1	2
SEPT7	4
LOC106830856	7
DNAJC17	2
GCHFR	5
LOC106830878	7
RMDN3	5
RAD51	2
CASC5	6
RPUSD2	6
LOC106830883	3
BAHD1	2
DDX3X	9
CASK	2
PMFBP1	1
DHX38	4
TXNL4B	4
IST1	2
ZNF821	5
ATXN1L	7

LOC106830902	5
AP1G1	6
PHLPP2	6
ZNF19	4
ZNF23	2
LOC106830925	5
CMTR2	6
LOC106830890	1
MKLN1	6
PODXL	2
CHCHD3	3
LOC106830923	1
FLVCR2	7
LOC106830937	1
TTLL5	5
IFT43	5
GPATCH2L	6
ANGEL1	1
LRRC74A	8
IRF2BPL	2
LOC106830951	1
CIPC	5
TMEM63C	1
NGB	6
POMT2	5
GSTZ1	7
TMED8	1
SAMD15	5
NOXRED1	1
VIPAS39	4
NEK10	1
SLC4A7	6
CMC1	2
AZI2	5
ZCWPW2	5
RBMS3	4
TGFBR2	2
STT3B	5
NELFE	5
SKIV2L	2
DXO	2
LOC106831006	7
TNXB	4
ATF6B	1

FKBPL	1
PPT2	2
AGPAT1	6
RNF5	2
PBX2	7
NOTCH4	1
LOC106831012	1
LOC106830971	1
HOXA3	2
HOXA7	2
HOXA9	2
HOXA13	5
LOC106831040	5
HIBADH	2
TAX1BP1	9
JAZF1	2
CREB5	2
TRIL	2
CPVL	2
CHN2	7
WIPF3	2
FKBP14	6
PLEKHA8	2
MTURN	2
LOC106831057	8
ZNRF2	5
GGCT	4
GARS	2
FAM188B	1
ADCYAP1R1	2
PDE1C	1
LSM5	4
AVL9	1
KBTBD2	5
TSTD3	6
CCNC	5
ASCC3	6
LOC106831099	8
LOC106831113	8
HSPA6	2
FCGR2B	1
DUSP12	2
ATF6	3
OLFML2B	5

UCK2	4
TMCO1	2
ALDH9A1	2
MGST3	2
LRRC52	1
LOC106831131	8
PBX1	2
LOC106831145	1
NUF2	2
RGS5	9
LOC106831142	5
LOC106831143	1
LOC106831147	7
LOC106831144	5
LOC106831155	4
PPIP5K2	6
GIN1	6
PAM	4
SLCO6A1	5
SLCO4C1	2
VEZT	4
FGD6	2
NR2C1	3
NDUFA12	3
TMCC3	8
CEP83	5
PLXNC1	2
LOC106831176	1
SOCS2	3
MRPL42	5
UBE2N	7
NUDT4	5
EEA1	5
LOC106831185	1
BTG1	8
DCN	2
CCER1	5
LOC106831199	1
ATP6V1B2	2
LOC106831209	5
SMAD7	9
LOC106831213	1
ZBTB7C	7
SMAD2	2

SKOR2	6
IER3IP1	2
HDHD2	2
KATNAL2	5
GATA3	2
LOC106831229	7
TAF3	1
ATP5C1	5
KIN	2
ITIH2	1
ITIH5	2
SFMBT2	3
PRKCQ	8
PFKFB3	7
LOC106831248	1
MRPL32	4
PSMA2	3
LOC106831256	5
TMEM144	4
CSK	2
ULK3	6
SCAMP2	2
MPI	5
FAM219B	1
LOC106831275	3
PPCDC	4
LOC106831280	7
COMMD4	2
MAN2C1	5
SIN3A	6
PTPN9	2
SNUPN	5
IMP3	2
ODF3L1	5
LINGO1	1
LOC106831307	8
HMG20A	2
PEAK1	1
TSPAN3	3
RCN2	3
SCAPER	4
ISL2	4
LOC106831320	7
LOC106831323	8

CCDC179	8
SVIP	5
FANCF	6
LOC106831350	7
SLC17A6	1
ANO5	1
LOC106831328	5
LOC106831314	4
ZNF25	1
LOC106831332	4
ZNF248	6
LOC106831337	6
LOC106831343	5
BMS1	3
RET	2
CSGALNACT2	6
RASGEF1A	2
HNRNPF	5
LOC106831346	1
LOC106831315	1
ZNF239	1
LOC106831353	7
WAPL	3
BMPR1A	2
ADIRF	7
GLUD1	2
FAM35A	2
ANTXRL	6
LOC106831357	9
LOC106831379	1
ZNF488	1
GDF10	2
LOC106831398	7
PTPN20	5
FRMPD2	7
MAPK8	9
ARHGAP22	6
WDFY4	1
LRRC18	7
VSTM4	4
FAM170B	1
ERCC6	2
DYRK1A	2
DSCR3	2

TTC3	2
PIGP	5
RIPPLY3	2
CHAF1B	4
MORC3	5
DOPEY2	2
CBR3	2
LOC106831423	4
SETD4	4
LOC106831432	8
PXDN	2
TMEM18	5
FAM150B	2
ACP1	5
SH3YL1	5
FAM110C	1
FAM92A1	5
RBM12B	6
TMEM67	1
PDP1	6
LOC106831437	8
RAD54B	2
KIAA1429	3
DPY19L4	6
INTS8	6
CCNE2	9
TP53INP1	2
NDUFAF6	2
PLEKHF2	4
LOC106831465	1
SEMA6A	2
COMMD10	2
AP3S1	3
ATG12	3
CDO1	2
TMED7	5
FEM1C	8
LOC106831439	1
RSU1	2
PLEKHG6	2
CD9	2
VWF	2
NTF3	1
NDUFA9	5

AKAP3	8
DYRK4	8
RAD51AP1	4
LOC106831497	3
FGF6	1
TIGAR	4
CCND2	2
PARP11	5
CRACR2A	1
LOC106831505	5
TEAD4	2
LOC106831509	4
TULP3	6
RHNO1	5
FOXM1	5
LOC106831512	7
FKBP4	5
LOC106831517	7
LOC106831519	2
IQSEC3	1
SLC6A13	1
KDM5A	4
CCDC77	6
WNK1	6
RAD52	6
LOC106831444	2
ARGLU1	5
LOC106831556	5
FAM155A	1
LOC106831541	10
LOC106831549	6
PTPN18	2
IMP4	2
CCDC115	3
TGFBR3	6
CDC7	2
HFM1	4
ZNF644	4
LOC106831586	10
ZNF326	4
LRRC8D	2
LRRC8B	7
LOC106831580	1
CCBL2	2

GTF2B	2
PKN2	4
TRIM21	2
RRM1	2
STIM1	6
RHOG	2
PGAP2	7
NUP98	8
LOC106831646	7
ART5	10
RNF121	5
NUMA1	4
LOC106831652	8
LAMTOR1	2
ANAPC15	3
INPPL1	2
PHOX2A	2
CLPB	7
ARAP1	2
STARD10	8
ATG16L2	2
FCHSD2	2
RELT	2
FAM168A	4
RAB6A	4
MRPL48	2
LOC106831678	9
PAAF1	5
DNAJB13	5
UCP2	5
UCP3	1
C2CD3	1
PPME1	5
P4HA3	2
PGM2L1	2
KCNE3	1
LIPT2	5
LOC106831692	6
LOC106831745	5
LOC106831728	1
ADAM29	1
CEP44	5
FBXO8	5
SAP30	2

LOC106831739	2
HMGB2	2
GALNT7	6
SGOL1	2
KAT2B	6
PP2D1	1
RAB5A	2
EFHB	5
SATB1	2
TBC1D5	6
PLCL2	5
LOC106831761	1
DAZL	2
RFTN1	4
OXNAD1	1
DPH3	5
LOC106831768	6
LOC106831784	7
LOC106831785	7
LOC106831783	7
LOC106831787	8
CYLC2	8
PPP3R2	5
LOC106831780	6
RNF20	5
ZNF189	4
MRPL50	4
PLPPR1	1
MURC	1
LOC106831797	4
TEX10	3
INVS	6
ERP44	5
STX17	2
LOC106831805	2
CDH2	2
DSC3	2
LOC106831823	6
ADGRB3	1
LOC106831826	1
LMBRD1	6
LOC106831827	1
LOC106831822	2
LOC106831846	5

SBDS	5
TMEM248	8
RABGEF1	6
KCTD7	1
TPST1	4
CRCP	2
ASL	4
GUSB	2
VKORC1L1	5
NUPR2	7
CHCHD2	2
SUMF2	6
CCT6A	2
PSPH	2
GBAS	4
MRPS17	5
ZNF713	6
SEPT14	2
AHSP	4
LOC106831868	2
TGFB1I1	5
ARMC5	1
LOC106831875	2
PYCARD	6
FUS	3
KAT8	3
BCKDK	5
VKORC1	2
ZNF646	5
ZNF668	2
STX4	4
SETD1A	2
LOC106831899	5
LOC106831839	1
BCL7C	2
ZNF629	1
RNF40	5
CCDC189	1
PHKG2	10
SRCAP	2
FBRS	5
PRR14	2
LOC106831911	7
ZNF689	1

LOC106831915	6
ZNF768	1
SEPHS2	2
DCTPP1	2
ZNF771	2
SEPT1	9
MYLPF	8
TBC1D10B	4
CD2BP2	4
HSD17B11	7
NUDT9	5
SPARCL1	9
PKD2	6
ABCG2	6
PPM1K	2
LOC106831957	1
HERC5	2
PYURF	6
HERC3	1
FAM13A	2
TIGD2	2
GPRIN3	8
ARHGAP42	2
LOC106831968	2
SPATA8	6
NR2F2	6
MCTP2	1
LOC106832003	1
RGMA	2
CHD2	6
FAM174B	2
AKAP13	4
MRPL46	4
MRPS11	2
DET1	6
AEN	9
MFGE8	2
ABHD2	7
FANCI	4
POLG	2
RHCG	5
KIF7	2
PEX11A	2
WDR93	5

MESP1	2
LOC106831984	2
LOC106832031	5
ARPIN	9
ZNF710	2
IDH2	2
LOC106832036	2
SEMA4B	2
CIB1	10
GDPGP1	5
LOC106832040	1
VPS33B	4
PRC1	5
RCCD1	8
UNC45A	6
HDDC3	1
MAN2A2	1
FES	9
FURIN	2
BLM	5
CRTC3	2
LOC106832054	6
ZSCAN2	4
WDR73	6
NMB	2
SEC11A	2
ZNF592	4
PDE8A	5
RPS17	2
CPEB1	2
AP3B2	6
LOC106832067	5
WHAMM	2
HOMER2	2
FAM103A1	2
LOC106831990	2
BTBD1	5
TM6SF1	5
LOC106831991	5
BNC1	4
SH3GL3	7
ADAMTSL3	1
SAXO2	1
ABHD17C	2

MESDC2	3
MESDC1	2
CFAP161	5
EEF1E1	2
BLOC1S5	5
LOC106832087	1
TXNDC5	2
BMP6	4
SNRNP48	6
DSP	2
RIOK1	4
CAGE1	1
SSR1	4
RREB1	4
LOC106832094	1
LOC106832097	1
LOC106832121	1
MED7	5
FAM71B	7
CYFIP2	2
SOX30	1
THG1L	5
LSM11	2
CLINT1	2
EBF1	2
RNF145	9
UBLCP1	3
ADRA1B	7
TTC1	2
PWWP2A	2
CCNJL	2
ZBED8	6
SLU7	4
PTTG1	3
CCNG1	2
NUDCD2	5
HMMR	5
MAT2B	2
FOXA2	2
COPS8	2
COL6A3	2
LRRFIP1	2
RBM44	4
UBE2F	2

SCLY	5
KLHL30	7
ILKAP	2
LOC106832195	5
PER2	5
TRAF3IP1	6
ASB1	5
NDUFA10	2
MYEOV2	2
GPC1	2
ANKMY1	1
CAPN10	8
LOC106832228	7
KIF1A	9
MTERF4	3
PASK	5
PPP1R7	10
HDLBP	5
SEPT2	9
STEAP1	2
CFAP69	5
GTPBP10	2
CLDN12	4
LOC106832231	1
CDK14	1
AKAP9	6
LOC106832249	5
LRRD1	1
KRIT1	4
ANKIB1	4
LOC106832253	5
GATAD1	2
PEX1	2
RBM48	3
LOC106832256	5
FAM133B	6
CDK6	6
LOC106832267	2
LOC106832235	2
CTNNA2	1
LOC106832272	1
ASB3	2
CHAC2	6
ERLEC1	5

GPR75	5
PSME4	1
LOC106832287	1
ACYP2	3
LOC106832289	1
SPTBN1	3
LOC106832291	7
EML6	4
RTN4	6
LOC106832292	7
CLHC1	5
RPS27A	2
MTIF2	6
LOC106832297	6
CCDC88A	5
CFAP36	5
PPP4R3B	3
PNPT1	6
EFEMP1	2
TLE2	2
S1PR4	1
NCLN	1
NFIC	6
FZR1	5
LOC106832357	7
HMG20B	2
TJP3	2
APBA3	2
MRPL54	2
MATK	2
ZFR2	1
ATCAY	8
DAPK3	5
EEF2	2
PIAS4	5
MAP2K2	4
SIRT6	2
CCDC94	5
SHD	7
FSD1	9
STAP2	2
SH3GL1	4
CHAF1A	5
UBXN6	7

LOC106832330	5
MYDGF	5
FEM1A	6
PLIN3	3
LOC106832385	8
ARRDC5	1
UHRF1	6
KDM4B	6
ZNRF4	7
SAFB2	4
SAFB	2
LOC106832396	7
HSD11B1L	1
RPL36	9
LONP1	9
CATSPERD	1
LOC106832339	7
LOC106832420	1
LOC106832398	2
GSC	2
URI1	4
CCNE1	5
TNNI1	7
NAV1	1
IPO9	2
SHISA4	2
TIMM17A	3
RNPEP	2
UBE2T	2
PCLO	2
SMOC2	2
CDK8	1
RNF6	10
SHISA2	2
ATP8A2	6
NUP58	5
MTMR6	5
SPATA13	2
MIPEP	7
TNFRSF19	2
SACS	2
LOC106832454	1
MMS22L	6
KLHL32	7

NDUFAF4	2
GPR63	2
FHL5	7
UFL1	3
MANEA	5
LOC106832467	1
LOC106832469	5
ACSL3	3
MOGAT1	2
FARSB	2
LOC106832483	1
JAKMIP1	6
EVC2	6
MSX1	2
LOC106832496	7
LOC106832497	1
EPHX2	2
CLU	2
CCDC25	3
ESCO2	6
PBK	5
SCARA5	7
NUGGC	1
ELP3	5
ZNF395	2
FZD3	1
EXTL3	2
INTS9	5
TBCCD1	1
DNAJB11	5
EIF4A2	2
RFC4	5
RTP1	1
BCL6	2
LPP	7
TPRG1	1
CCDC50	2
FGF12	1
MB21D2	6
HRASLS	5
ATP13A4	6
OPA1	6
HES1	2
LOC106832593	8

ATP13A3	4
LSG1	2
FAM43A	2
VRK1	2
PAPOLA	3
AK7	5
GSKIP	7
ATG2B	6
LOC106832630	6
SYNE3	1
CLMN	7
DICER1	2
CCDC171	5
PSIP1	3
SNAPC3	4
TTC39B	1
FREM1	1
ZDHHC21	4
NFIB	2
LOC106832669	2
MPDZ	2
LURAP1L	2
CIB4	5
LOC106832678	8
DRC1	5
EPT1	6
HADHB	2
MRPS9	5
TGFBRAP1	2
LOC106832692	1
FHL2	5
NCK2	2
LOC106832695	1
LOC106832696	2
UXS1	2
LOC106832721	2
GK	6
TAB3	2
XRCC5	3
PECR	2
MREG	2
FN1	2
ATIC	2
BARD1	4

LOC106832746	5
LOC106832724	5
NDUFA11	9
VMAC	8
RANBP3	5
RFX2	5
ACSBG2	7
MLLT1	1
CLPP	5
ALKBH7	5
GTF2F1	3
LOC106832756	7
KHSRP	2
SLC25A41	7
GPR108	4
TRIP10	6
SORL1	2
LOC106832788	7
LOC106832769	1
LOC106832773	5
HSPA8	2
GRAMD1B	5
ZNF202	2
TMEM225	5
LOC106832804	8
LOC106832849	1
RUNX1	5
RCAN1	4
LOC106832850	1
SMIM11A	2
MRPS6	2
SLC5A3	2
ATP5O	5
ITSN1	4
CRYZL1	5
DONSON	3
SON	3
DNAJC28	1
TMEM50B	5
IFNGR2	2
LOC106832839	1
IFNAR1	2
IL10RB	9
IFNAR2	6

KCNT2	5
LOC106832852	4
LOC106832862	2
LOC106832863	1
LOC106832860	6
LOC106832864	2
LOC106832854	4
LOC106832865	2
TIMM21	6
FBXO15	1
NETO1	7
VPS4B	2
KDSR	2
BCL2	2
PHLPP1	1
ZCCHC2	5
KIAA1468	4
WASF3	4
USP12	2
RPL21	2
RASL11A	2
GTF3A	2
MTIF3	5
LOC106832901	2
POLR1D	2
LOC106832904	5
CDX2	2
PAN3	6
FLT1	2
POMP	5
SLC46A3	5
MTUS2	2
SLC7A1	6
UBL3	6
KATNAL1	6
ZNF697	2
HSD3B2	2
IKZF4	1
RPS26	2
PA2G4	3
ZC3H10	9
ESYT1	5
MYL6B	7
MYL6	2

SMARCC2	2
LOC106832949	1
RNF41	1
NABP2	4
CS	2
CNPY2	2
PAN2	4
IL23A	1
STAT2	2
LOC106832962	1
TIMELESS	4
GLS2	6
RBMS2	6
BAZ2A	6
ATP5B	3
PTGES3	2
NACA	3
PRIM1	5
HSD17B6	2
LOC106832929	1
GPR182	8
NEMP1	1
NAB2	2
LRP1	2
SHMT2	6
R3HDM2	6
MARS	4
DDIT3	3
MBD6	6
DCTN2	3
PIP4K2C	2
DTX3	1
B4GALNT1	5
OS9	5
TSPAN31	6
CDK4	2
9-Mar	6
METTL1	6
TSFM	2
CTDSP2	7
LOC106833027	5
LOC106833028	1
XRCC6BP1	4
SLC16A7	6

DTWD2	1
DMXL1	1
TNFAIP8	2
HSD17B4	5
FAM170A	5
FBXO33	4
CTAGE5	3
PNN	2
TRAPPC6B	3
GEMIN2	2
SEC23A	3
TTC6	5
FOXA1	7
LOC106833074	7
PDE3B	1
PSMA1	3
COPB1	2
RRAS2	2
FAR1	7
LOC106833081	7
BTBD10	5
ARNTL	6
TEAD1	6
PARVA	4
MICAL2	1
DKK3	2
USP47	3
ZBED5	5
EIF4G2	2
CTR9	3
LOC106833100	5
RNF141	5
ADM	2
SBF2	6
SWAP70	2
WEE1	2
ZNF143	4
LOC106833108	5
IPO7	6
TMEM41B	6
DENND5A	2
NRIP3	2
LOC106833113	5
LOC106833115	6

AKIP1	3
ST5	4
RPL27A	2
TRIM66	8
STK33	5
LMO1	2
LOC106833124	1
RIC3	6
TUB	6
EIF3F	3
MBP	2
ZNF236	2
LOC106833146	5
LOC106833140	1
ZNF516	6
ZADH2	2
LOC106833153	2
KTI12	2
LOC106833165	5
BTF3L4	5
ZFYVE9	2
CC2D1B	4
ORC1	2
PRPF38A	4
ZCCHC11	6
GPX7	2
FAM159A	9
LOC106833177	2
ZYG11B	6
ZYG11A	4
SCP2	4
CPT2	5
LOC106833187	2
MAGOH	2
LRP8	1
LOC106833194	1
LOC106833192	5
DMRTB1	2
NDC1	3
YIPF1	3
HSPB11	3
LRRC42	2
TMEM59	3
TCEANC2	1

MRPL37	2
ACOT11	7
FAM151A	1
MROH7	8
TTC4	2
TTC22	1
LEXM	7
DHCR24	2
BSND	1
USP24	4
ARHGAP20	6
FDX1	3
RDX	2
ZC3H12C	6
DDX10	5
ATP10A	1
UBE3A	2
LOC106833243	1
SNRPN	9
SNURF	8
LOC106833253	8
METTL3	4
TOX4	3
RAB2B	7
CHD8	2
SUPT16H	2
RPGRIP1	5
HNRNPC	2
ZNF219	2
TPPP2	7
NDRG2	2
METTL17	4
RNASE1	7
RNASE6	7
LOC106833279	6
RNASE11	1
RNASE9	1
LOC106833281	4
LOC106833283	9
LOC106833287	2
TMEM55B	2
APEX1	4
OSGEP	3
PARP2	2

LOC106833297	1
CCNB1IP1	4
TTC5	2
LOC106833444	6
GNAI1	4
SEMA3C	2
LOC106833456	8
HOOK1	7
FGGY	4
LOC106833461	7
LOC106833464	2
JUN	2
MYSM1	2
TACSTD2	2
LOC106833467	2
OMA1	2
LOC106833469	7
DAB1	1
PRKAA2	6
PLPP3	2
NUDT8	9
NDUFV1	2
LOC106833486	2
CDK2AP2	2
PITPNM1	8
AIP	2
TMEM134	9
LOC106833494	1
CORO1B	2
RPS6KB2	7
CARNS1	5
TBC1D10C	2
RAD9A	1
POLD4	8
SSH3	2
ANKRD13D	4
ADRBK1	2
KDM2A	6
RHOD	2
PC	2
LRFN4	1
RCE1	6
LOC106833659	1
SPTBN2	2

RBM4B	10
LOC106833517	3
LOC106833516	5
CCS	2
CCDC87	5
CTSF	5
ACTN3	7
ZDHHC24	6
BBS1	1
DPP3	2
PELI3	2
MRPL11	5
BRMS1	5
TMEM151A	8
YIF1A	1
CNIH2	6
RAB1B	6
KLC2	2
PACS1	1
SF3B2	3
CATSPER1	1
CST6	2
BANF1	2
EIF1AD	5
SART1	4
TSGA10IP	5
DRAP1	5
LOC106833551	2
CCDC85B	9
FIBP	9
CTSW	2
EFEMP2	2
MUS81	7
CFL1	2
SNX32	1
LOC106833561	5
OVOL1	6
AP5B1	6
RNASEH2C	8
KAT5	3
RELA	2
SIPA1	2
PCNXL3	2
MAP3K11	4

KCNK7	2
EHBP1L1	1
FAM89B	2
SSSCA1	5
LTBP3	2
SCYL1	2
LOC106833578	1
LOC106833576	1
LOC106833580	2
TIGD3	5
DPF2	2
CDC42EP2	2
POLA2	4
LOC106833596	5
SYVN1	2
MRPL49	4
FAU	2
ZNHIT2	5
VPS51	5
TMEM262	5
ZFPL1	5
CDCA5	5
SAC3D1	2
SNX15	2
ARL2	10
LOC106833609	5
ATG2A	4
EHD1	5
MAP4K2	2
SF1	1
PYGM	7
RASGRP2	6
CCDC88B	5
PRDX5	10
TRMT112	2
ESRRA	6
TEX40	5
GPR137	7
BAD	5
PPP1R14B	5
FKBP2	2
VEGFB	2
DNAJC4	10
NUDT22	3

TRPT1	1
STIP1	2
LOC106833642	2
OTUB1	2
LOC106833641	2
NAA40	4
MARK2	6
LOC106833648	2
RTN3	3
ATL3	9
NSFL1C	3
SDCBP2	4
SNPH	1
RAD21L1	4
TMEM74B	2
PSMF1	10
ANGPT4	1
LOC106833752	9
CSNK2A1	2
TBC1D20	8
RBCK1	2
TRIB3	4
ZCCHC3	2
LOC106833703	6
DEFB119	9
LOC106833705	1
LOC106833706	9
DEFB123	2
REM1	2
HM13	5
ID1	2
LOC106833711	2
BCL2L1	5
TPX2	3
FOXS1	2
DUSP15	5
TTLL9	5
PDRG1	2
HCK	1
TM9SF4	1
PLAGL2	4
KIF3B	1
ASXL1	4
NOL4L	5

LOC106833736	10
COMMD7	3
MAPRE1	2
EFCAB8	1
SUN5	1
BPIFB2	1
BPIFB3	6
BPIFB4	2
BPIFA2	8
NET1	4
ASB13	7
FAM208B	5
GDI2	2
FBXO18	2
RBM17	3
NME7	1
BLZF1	6
CCDC181	5
METTL18	5
LOC106833806	2
SCYL3	6
KIFAP3	1
METTL11B	7
GORAB	4
PRRX1	2
MROH9	1
PRRC2C	2
VAMP4	3
METTL13	2
DNM3	1
LOC106833887	1
LOC106833843	2
LOC106833840	8
LOC106833889	2
TRPV6	1
TRPV5	1
LOC106833850	8
PIP	8
GSTK1	2
LOC106833857	1
BEND5	2
SPATA6	8
LOC106833860	2
CMPK1	9

STIL	4
TAL1	1
LOC106833866	2
LOC106833869	1
GLUL	7
CACNA1E	6
IER5	1
STX6	2
XPR1	4
LOC106833914	7
ACBD6	2
QSOX1	6
CEP350	5
TOR1AIP1	7
LOC106833921	4
LOC106833923	4
TDRD5	4
NPHS2	5
AXDND1	5
SOAT1	2
ABL2	8
TOR3A	6
FAM20B	9
RALGPS2	2
LOC106833935	10
LOC106833991	1
TEX35	7
LINC00083	6
RASAL2	6
LOC106833942	2
LOC106833994	7
PAPPA2	2
LOC106833949	4
RFWD2	6
TNN	4
LOC106833956	1
MRPS14	3
CACYBP	3
LOC106833958	4
B4GALT6	2
TRAPPC8	2
RNF125	8
RNF138	7
GAREM1	6

CCDC178	5
ASXL3	6
NOL4	5
AGA	2
DPYSL2	2
PNMA2	2
BNIP3L	2
PPP2R2A	4
EBF2	2
ST3GAL4	2
DCPS	2
TIRAP	5
FOXRED1	2
SRPRA	4
FAM118B	3
RPUSD4	3
CDON	2
DDX25	5
PUS3	6
HYLS1	10
LOC106834032	1
LOC106834033	5
TJP1	2
FAM189A1	2
NSMCE3	6
APBA2	2
MCEE	3
MPHOSPH10	3
FAN1	2
MTMR10	4
LOC106834065	1
CHRNA7	2
NDN	2
ANXA5	2
EXOSC9	3
CCNA2	3
BBS7	5
KIAA1109	2
ADAD1	5
LOC106834080	5
BBS12	1
NUDT6	5
SPATA5	5
ANKRD50	3

IWS1	4
PROC	8
MAP3K2	2
ERCC3	2
BIN1	2
LOC106834141	5
LOC106834143	7
LOC106834109	2
YBX3	5
SMIM10L1	2
LOC106834114	1
LOC106834115	5
ETV6	2
BCL2L14	7
LRP6	6
LOC106834151	1
LOC106834152	8
THOP1	5
SGTA	5
SLC39A3	5
GNG7	8
GADD45B	2
LMNB2	1
TIMM13	2
TMPRSS9	2
SPPL2B	1
LSM7	2
AMH	9
SF3A2	1
PLEKHJ1	6
DOT1L	6
AP3D1	9
IZUMO4	5
MOB3A	5
MKNK2	2
BTBD2	2
LOC106834170	7
SCAMP4	2
ABHD17A	5
REXO1	8
ATP8B3	8
TCF3	2
LOC106834176	9
LOC106834177	2

MBD3	4
PLK5	2
REEP6	7
PCSK4	1
LOC106834182	3
RPS15	2
DAZAP1	5
GAMT	9
NDUFS7	5
LOC106834187	5
LOC106834225	9
LOC106834190	6
MIDN	7
ATP5D	2
STK11	10
GPX4	10
POLR2E	3
HMHA1	2
ABCA7	2
WDR18	4
LOC106834259	1
R3HDM4	1
MED16	5
CFD	2
PTBP1	2
MISP	1
FSTL3	2
RNF126	5
POLRMT	8
BSG	10
GZMM	4
CDC34	10
TPGS1	5
ODF3L2	7
SHC2	9
LOC106834255	2
THEG	1
SLC22A14	1
SLC22A13	1
OXSR1	6
LOC106834264	1
ACAA1	2
DLEC1	1
VILL	6

CTDSPL	2
GOLGA4	3
LRRFIP2	5
MLH1	2
TRANK1	6
LOC106834281	2
DCLK3	2
FAM135A	6
SDHAF4	9
SMAP1	9
B3GAT2	1
GALC	1
GPR65	1
SPATA7	10
PTPN21	1
ZC3H14	3
EML5	2
TTC8	1
FOXN3	2
GGH	2
LOC106834314	1
YTHDF3	2
LOC106834330	1
ARMC1	4
MTFR1	10
PDE7A	5
DNAJC5B	7
LOC106834325	8
LOC106834331	7
ADA	2
PKIG	5
SERINC3	4
TTPAL	6
LOC106834340	2
FITM2	2
GDAP1L1	9
OSER1	7
JPH2	2
TOX2	2
GTSF1L	7
MYBL2	2
IFT52	5
LOC106834349	1
SGK2	9

L3MBTL1	1
SRSF6	2
CHD6	2
ZHX3	1
PLCG1	4
TOP1	4
MAFB	4
CCDC150	5
GTF3C3	6
ANKRD44	1
SF3B1	3
COQ10B	9
HSPD1	2
LOC106834369	2
RFTN2	1
MARS2	1
BOLL	5
SATB2	1
LOC106834375	6
LOC106834376	5
TYW5	6
LOC106834378	6
FXN	2
TJP2	2
FAM189A2	4
APBA1	9
PTAR1	6
LOC106834382	5
SMC5	5
KLF9	1
PLXDC2	4
ARL5B	6
NSUN6	6
CACNB2	1
STAM	6
HACD1	1
VIM	2
TRDMT1	1
BANK1	1
PPP3CA	3
LOC106834432	5
LOC106834439	5
DNAJB14	6
LAMTOR3	3

DAPP1	9
LOC106834444	1
TRMT10A	6
LOC106834448	5
ALDH1L1	2
CFAP100	1
ZXDC	2
TXNRD3	3
CHCHD6	5
LOC106834465	1
PLXNA1	2
TPRA1	2
MCM2	2
PODXL2	2
ABTB1	2
MGLL	2
KBTBD12	5
SEC61A1	9
RUVBL1	3
EEFSEC	2
GATA2	2
RPN1	3
RAB7A	2
ETV5	4
LOC106834548	1
TRA2B	3
IGF2BP2	2
SENP2	5
TMEM41A	2
MAP3K13	6
LOC106834500	1
LOC106834503	4
VPS8	1
MAGEF1	2
EPHB3	2
LOC106834509	1
CHRD	9
POLR2H	2
FAM131A	1
EIF4G1	4
PSMD2	5
ALG3	6
ABCF3	5
AP2M1	3

DVL3	7
EIF2B5	2
ABCC5	1
PARL	3
YEATS2	2
KLHL24	2
MCF2L2	9
LAMP3	1
MCCC1	2
DCUN1D1	7
TULP1	5
RPL10A	2
FANCE	2
PPARD	6
ZNF76	2
TCP11	7
SCUBE3	1
ANKS1A	6
TAF11	5
UHRF1BP1	6
SNRPC	5
LOC106834573	7
RPS10	2
NUDT3	3
LOC106834578	2
HMGA1	2
LOC106834583	5
ITPR3	2
LOC106834585	5
LOC106834587	6
ZBTB9	2
CUTA	2
PHF1	8
KIFC1	5
DAXX	5
PFDN6	5
WDR46	3
RPS18	2
RING1	6
HSD17B8	6
SLC39A7	6
RXRB	1
BRD2	3
LOC106834610	6

LOC106834611	2
PSMB9	2
PSMB8	2
TAP2	2
LOC106834617	6
LOC106834619	3
LOC106834624	8
MGMT	2
LOC106834644	2
GLRX3	5
SAR1B	3
JADE2	2
CDKN2AIPNL	2
UBE2B	3
CDKL3	8
PPP2CA	3
SKP1	3
TCF7	2
LOC106834659	5
PDCD10	2
SERPINI1	2
LOC106834684	6
GOLIM4	2
MECOM	2
ACTRT3	8
MYNN	6
LRRC34	5
LRRIQ4	7
SEC62	5
GPR160	7
PHC3	5
RYR2	2
MTR	2
HEATR1	2
LGALS8	10
ERO1B	5
GPR137B	4
LYST	6
GNG4	7
B3GALNT2	8
TBCE	5
GGPS1	6
ARID4B	6
RBM34	3

TOMM20	5
IRF2BP2	2
TARBP1	2
LOC106834708	5
PDK1	10
ITGA6	2
METAP1D	5
HAT1	3
SLC25A12	6
DYNC1I2	3
LOC106834745	6
DCAF17	5
METTL8	1
TLK1	6
GORASP2	5
LOC106834731	1
LOC106834732	1
LOC106834744	7
OLA1	3
SP3	2
CDCA7	2
LOC106834739	4
FAM20C	2
PDGFA	2
PRKAR1B	9
DNAAF5	1
SUN1	6
GET4	9
ADAP1	10
LOC106834758	4
LOC106834763	2
ZFAND2A	2
MICALL2	6
INTS1	4
MAFK	6
PSMG3	2
ELFN1	2
MAD1L1	1
FTSJ2	7
NUDT1	5
SNX8	2
EIF3B	2
CHST12	2
LFNG	2

IQCE	5
BRAT1	2
GNA12	2
LOC106834823	1
SEL1L	2
STON2	6
LOC106834798	6
GTF2A1	4
CEP128	1
DIO2	5
SNW1	3
SLIRP	4
ALKBH1	6
SPTLC2	4
AHSA1	3
LOC106834833	1
PUM3	2
VLDLR	6
SMARCA2	3
DMRT2	2
DMRT1	2
LOC106834845	2
LOC106834859	1
PIP5K1B	1
FAM122A	6
ZSCAN25	6
ZNF655	1
FAM200A	1
ZKSCAN5	6
LOC106834867	2
ZNF789	4
TRIM4	4
NRXN1	6
LMNTD1	1
LOC106834885	1
RASSF8	8
ITPR2	2
ASUN	3
FGFR1OP2	4
TM7SF3	2
MED21	5
STK38L	5
ARNTL2	1
SMCO2	5

PPFIBP1	1
LOC106834899	5
MRPS35	2
KLHL42	4
CCDC91	7
ATOX1	10
G3BP1	2
GLRA1	7
FAM114A2	2
MFAP3	2
GALNT10	6
SAP30L	3
HAND1	2
CTDP1	3
NFATC1	1
ATP9B	4
DYNLT1	5
TMEM181	2
TULP4	6
GTF2H5	5
SERAC1	5
SYNJ2	7
SNX9	2
ZDHHC14	7
TMEM242	2
LOC106834939	3
LOC106834947	5
ARID1B	6
LOC106834949	2
LOC106834950	1
FATE1	9
VMA21	2
HMGB3	4
MTM1	2
LOC106834965	5
POP4	2
PLEKHF1	2
PCDH10	1
LOC106835039	7
BLOC1S6	7
SLC30A4	7
LOC106834987	2
SPATA5L1	2
GATM	2

DUOXA2	5
SORD	2
LOC106835000	5
TRIM69	5
B2M	2
SPG11	6
EIF3J	2
CTDSPL2	9
CASC4	1
WDR76	5
MFAP1	3
HYPK	3
SERINC4	1
SERF2	5
ELL3	7
PDIA3	3
CATSPER2	5
MAP1A	6
TP53BP1	6
ADAL	4
CCNDBP1	2
TMEM62	1
LOC106835086	7
MYL9	2
TGIF2	3
LOC106835046	5
NDRG3	1
SOGA1	2
SAMHD1	2
RBL1	1
LOC106835058	1
MROH8	6
RPN2	3
GHRH	2
MANBAL	2
BLCAP	9
NNAT	2
CTNNBL1	3
RPRD1B	6
BPI	1
LOC106835073	5
RALGAPB	6
ADIG	7
SLC32A1	7

ACTR5	5
FAM83D	2
DHX35	2
PRR27	1
CABS1	7
JCHAIN	2
UTP3	3
RUFY3	1
GRSF1	2
MOB1B	1
DCK	7
ADAMTS3	1
LOC106835105	2
ANKRD17	2
ALB	1
RASSF6	1
MRPL19	5
EVA1A	7
POLE4	2
LOC106835133	1
LOC106835134	1
LPCAT4	2
NUTM1	1
NOP10	2
SLC12A6	6
EMC4	5
KATNBL1	2
EMC7	5
AVEN	8
FMN1	5
SCG5	2
ARHGAP11A	6
ACTC1	2
AQR	4
ZNF770	2
DPH6	6
TMCO5A	8
SPRED1	5
FAM98B	6
LOC106835161	7
THBS1	4
FSIP1	1
LOC106835164	1
GPR176	1

EIF2AK4	2
SRP14	2
BUB1B	3
PAK6	2
ANKRD63	8
PLCB2	1
LOC106835174	7
DISP2	1
KNSTRN	10
LOC106835306	5
SLC11A2	4
LETMD1	2
CSRNP2	6
TFCP2	2
DAZAP2	3
SMAGP	6
BIN2	5
SLC4A8	1
SCN8A	6
ACVR1B	2
NR4A1	2
ATG101	2
KRT80	7
KRT7	2
LOC106835314	1
LOC106835264	8
LOC106835252	1
KRT72	2
KRT8	2
LOC106835271	6
KRT18	2
EIF4B	2
TNS2	4
SPRYD3	2
IGFBP6	2
MFSD5	7
ESPL1	1
PFDN5	10
LOC106835285	7
AAAS	5
SP1	1
PRR13	4
PCBP2	3
TARBP2	2

ATF7	2
CITED1	2
RPS4X	2
ERCC6L	6
PIN4	2
RARRES1	2
GFM1	3
LXN	2
LOC106835338	2
LOC106835340	5
SSR3	3
LOC106835343	1
TIPARP	2
CEP112	1
AXIN2	1
RGS9	2
LOC106835352	2
GNA13	2
AMZ2	10
ARSG	1
SLC16A6	2
WIPI1	6
PRKAR1A	3
LOC106835370	1
ABCA8	9
LOC106835366	6
LAMA2	6
L3MBTL3	2
EPB41L2	6
MED23	2
FUT10	1
MAK16	1
TTI2	8
LOC106835419	5
MTERF3	5
PTDSS1	9
SDC2	2
CPQ	4
LOC106835425	8
TSPYL5	4
LOC106835429	1
MTDH	5
LAPTM4B	5
RPL30	2

ERICH5	1
HRSP12	5
POP1	6
NIPAL2	2
STK3	2
LOC106835441	1
LOC106835444	1
TRIM27	2
ZNF311	1
LOC106835447	1
LOC106835457	4
SDCCAG8	1
CEP170	5
INPP4B	1
LOC106835461	7
AUTS2	4
LOC106835463	1
TUSC3	5
INSC	1
SOX6	1
LOC106835474	3
RPS13	2
PIK3C2A	6
NUCB2	2
USH1C	6
KCNC1	6
SAAL1	3
LOC106835497	8
PDHA1	2
LOC106835503	5
MAP7D2	2
EIF1AX	9
RPS6KA3	2
CNKSR2	2
MBTPS2	4
SMS	2
TANC2	2
LOC106835519	1
ACE	7
LOC106835568	1
DCAF7	5
LOC106835524	2
MAP3K3	4
STRADA	10

CCDC47	5
DDX42	2
FTSJ3	6
PSMC5	5
SMARCD2	2
LOC106835535	2
PRR29	1
ERN1	4
LOC106835542	2
TEX2	5
PECAM1	7
MILR1	7
POLG2	5
DDX5	3
CEP95	1
SMURF2	3
KPNA2	5
LOC106835556	2
BPTF	4
NOL11	2
PITPNC1	4
LOC106835559	6
PSMD12	2
HELZ	4
PRKCA	6
LOC106835635	2
RHOB	2
PUM2	4
SDC1	2
LAPTM4A	3
MATN3	2
WDR35	5
TTC32	2
LOC106835586	8
RDH14	5
GEN1	6
SMC6	5
RAD51AP2	5
LOC106835647	8
FAM49A	2
MYCN	2
DDX1	2
NBAS	6
TRIB2	2

LOC106835604	7
MTMR14	5
BRPF1	5
OGG1	5
TADA3	5
LOC106835611	8
RPUSD3	2
JAGN1	5
CRELD1	3
PRRT3	1
EMC3	3
FANCD2	5
FANCD2OS	5
BRK1	3
VHL	2
TATDN2	4
GHRL	8
SEC13	5
SLC6A11	2
FEM1B	5
ANP32A	2
SPESP1	5
GLCE	2
PAQR5	7
KIF23	5
RPLP1	9
TLE3	1
UACA	2
LARP6	2
LRRC49	5
DENND2C	2
NRAS	4
CSDE1	3
SIKE1	2
SYCP1	5
VANGL1	2
ATP1A1	2
CD58	5
IGSF3	2
CD2	2
PTGFRN	2
TTF2	2
TRIM45	5
LOC106835701	5

MAN1A2	3
FAM46C	8
LOC106835713	2
LOC106835803	2
LOC106835804	1
AIM2	1
DUSP23	2
CFAP45	5
LOC106835833	1
TAGLN2	2
IGSF9	1
SLAMF9	1
PIGM	1
KCNJ9	1
ATP1A4	5
CASQ1	1
PEA15	7
DCAF8	4
PEX19	2
COPA	4
NCSTN	4
SLAMF1	1
LY9	1
CD244	1
CD55	1
LOC106835778	5
LOC106835838	1
AKIRIN2	10
RARS2	1
SLC35A1	1
CFAP206	5
LOC106835845	8
SMIM8	2
GJB7	5
ZNF292	6
CCDC127	5
SDHA	6
PDCD6	2
AHRR	1
EXOC3	5
SLC9A3	7
CEP72	5
TPPP	8
ZDHHC11	1

BRD9	2
TRIP13	2
SLC12A7	1
CLPTM1L	3
LOC106835882	8
MRPL36	6
NDUFS6	2
IRX4	4
LOC106835889	1
LOC106835890	2
LOC106835902	2
TRIM23	2
PPWD1	4
CENPK	6
ADAMTS6	1
CWC27	2
SREK1IP1	5
FAM159B	7
RGS7BP	5
RNF180	2
IPO11	4
LOC106835932	8
LOC106835933	1
LOC106835926	6
LOC106835928	1
LOC106835935	5
LOC106835936	1
LOC106835938	1
TBRG1	3
SIAE	1
SPA17	5
NRGN	2
VSIG2	2
ESAM	1
MSANTD2	6
ROBO3	2
CCDC15	5
SLC37A2	1
TMEM218	4
FEZ1	5
EI24	2
STT3A	5
CHEK1	2
ACRV1	1

LOC106835964	7
LOC106835965	9
LOC106836138	1
LOC106836129	1
ZNF567	6
LOC106836088	1
ZNF461	6
ZNF382	1
LOC106836124	6
ZNF566	4
LOC106836089	4
LOC106836123	1
LOC106836137	4
LOC106836144	5
LOC106836135	4
LOC106836139	1
ZNF568	1
ZNF829	6
LOC106836145	1
LOC106836126	2
LOC106836118	4
LOC106836130	6
LOC106836112	6
LOC106836110	1
ZNF570	1
ZNF569	3
LOC106836142	8
LOC106836109	4
LOC106836141	4
LOC106836052	2
LOC106836094	2
LOC106836095	1
LOC106836054	5
DPF1	1
PPP1R14A	2
SPINT2	5
LOC106836060	2
YIF1B	5
CATSPERG	5
PSMD8	5
GGN	5
SPRED3	1
FAM98C	2
RYR1	7

LOC106836066	5
MAP4K1	2
EIF3K	2
LGALS4	8
ECH1	2
HNRNPL	2
SIRT2	8
NFKBIB	4
SARS2	8
MRPS12	5
FBXO17	7
FBXO27	5
PAK4	6
POLR3K	6
SNRNP25	2
MPG	2
HBA1	2
LUC7L	5
FAM234A	1
ARHGDIG	2
AXIN1	4
MRPL28	5
TMEM8A	7
NME4	2
DECR2	2
PIGQ	5
RAB40C	2
FAM195A	2
WDR90	6
RHOT2	2
LOC106836178	1
STUB1	3
WDR24	8
METRN	2
FAM173A	2
HAGHL	5
NARFL	9
MSLN	1
RPUSD1	2
CHTF18	3
LMF1	5
LOC106836194	5
CACNA1H	2
UBE2I	2

TSR3	2
GNPTG	10
UNKL	6
LOC106836202	2
CCDC154	1
CLCN7	2
TELO2	6
IFT140	5
CRAMP1	6
HN1L	7
MAPK8IP3	6
SPSB3	7
NUBP2	7
IGFALS	8
HAGH	5
FAHD1	5
MEIOB	6
HS3ST6	1
NDUFB10	5
RPS2	2
RNF151	7
TBL3	5
NOXO1	1
GFER	8
SYNGR3	2
ZNF598	2
NPW	2
SLC9A3R2	9
NTHL1	2
TSC2	4
PKD1	2
LOC106836284	6
TRAF7	2
MLST8	4
BRICD5	8
PGP	10
E4F1	6
DNASE1L2	8
ECI1	10
RNPS1	2
ABCA3	2
LOC106836248	1
CCNF	1
LOC106836250	5

LOC106836254	3
LOC106836255	2
LOC106836256	2
PCMTD2	2
OPRL1	1
LKAAEAR1	1
TCEA2	5
PRPF6	3
SAMD10	7
ZNF512B	6
UCKL1	5
DNAJC5	9
TPD52L2	7
ABHD16B	7
ZBTB46	2
SLC2A4RG	2
ZGPAT	4
ARFRP1	5
TNFRSF6B	2
RTEL1	1
GMEB2	4
LOC106836313	5
SRMS	8
PPDPF	7
EEF1A2	8
ARFGAP1	5
YTHDF1	4
GID8	3
DIDO1	1
LOC106836417	1
TCFL5	5
OGFR	2
MRGBP	5
LOC106836332	9
GATA5	2
CABLES2	2
RPS21	2
LAMA5	2
ADRM1	9
MTG2	2
SS18L1	2
PSMA7	2
LOC106836345	9
PHACTR3	2

SYCP2	5
FAM217B	1
AKNA	1
COL27A1	1
KIF12	2
ZNF618	2
KIF16B	2
SEC23IP	2
MCMBP	2
INPP5F	2
BAG3	2
TIAL1	3
RGS10	2
GRK5	5
SFXN4	2
FAM45A	9
EIF3A	2
NANOS1	2
CACUL1	5
FAM204A	5
RAB11FIP2	4
EMX2	7
PDZD8	4
SLC18A2	2
SHTN1	6
ENO4	5
HSPA12A	1
LOC106836384	7
PNLIPRP2	2
CCDC172	2
GFRA1	2
TRUB1	2
FAM160B1	1
LOC106836426	8
LOC106836429	5
LOC106836430	1
ARL4D	7
DHX8	3
DUSP3	8
LOC106836437	8
TMEM101	5
LSM12	7
G6PC3	3
HDAC5	2

LOC106836448	2
ASB16	2
TMUB2	4
ATXN7L3	7
UBTF	2
RUNDC3A	7
SLC25A39	10
GRN	2
ITGA2B	5
GPATCH8	2
FZD2	2
MEIOC	8
CCDC43	6
GJC1	2
EFTUD2	2
CCDC103	5
LOC106836468	1
KIF18B	5
C1QL1	9
DCAKD	2
NMT1	5
PLCD3	5
HEXIM1	6
LOC106836479	1
SPATA32	7
MAP3K14	4
PLEKHM1	1
LOC106836484	5
LYZL6	5
LOC106836488	1
GOSR2	9
WNT3	7
NSF	6
LOC106836492	6
SPPL2C	7
MAPT	8
KANSL1	2
CDC27	5
MYL4	5
TMTC3	6
CEP290	6
LOC106836518	7
LOC106836529	8
ECHDC3	5

PROSER2	4
UPF2	6
DHTKD1	1
SEC61A2	2
LOC106836526	2
MGAT5	2
CCNT2	4
RAB3GAP1	4
ZRANB3	4
R3HDM1	2
UBXN4	5
LCT	5
MCM6	2
DARS	2
LOC106836548	5
CXCR4	2
MYLIP	2
DTNBP1	6
JARID2	4
CD83	2
LOC106836558	5
RNF182	6
MCUR1	2
RANBP9	5
NOL7	3
SIRT5	2
TBC1D7	2
LOC106836565	1
PHACTR1	7
MME	2
DHX36	8
ARHGEF26	9
RAP2B	4
P2RY1	2
MBNL1	2
LOC106836601	5
TTC23	5
SYNM	2
IGF1R	2
PGPEP1L	5
ARRDC4	9
LOC106836611	1
LOC106836613	7
EPC1	5

LOC106836615	5
LOC106836630	1
LOC106836631	8
LOC106836616	8
ITGB1	2
NRP1	2
PARD3	4
CUL2	2
LOC106836626	1
CREM	5
MYO9A	5
SENP8	5
PKM	9
PARP6	7
CELF6	5
HEXA	6
LOC106836642	5
LOC106836643	5
LOC106836644	6
NEO1	4
HCN4	1
REC114	2
NPTN	6
CD276	2
TBC1D21	7
STOML1	2
DHRS3	2
VPS13D	1
TNFRSF8	1
MIIP	8
MFN2	8
PLOD1	4
KIAA2013	1
LOC106836706	7
NPPA	5
CLCN6	4
MTHFR	1
LOC106836701	1
AGTRAP	5
DRAXIN	1
MAD2L2	9
LOC106836671	6
MTOR	4
EXOSC10	3

SRM	2
TARDBP	5
LOC106836703	1
PEX14	6
DFFA	2
LOC106836685	10
PGD	2
KIF1B	6
UBE4B	2
NMNAT1	4
LZIC	5
CTNNBIP1	2
CLSTN1	2
PIK3CD	2
TMEM201	2
SLC25A33	3
LYPLAL1	2
TGFB2	2
RRP15	2
SPATA17	5
GPATCH2	5
KCTD3	2
RGS2	2
UCHL5	7
TROVE2	2
GLRX2	9
TXNDC11	6
SNN	2
LITAF	2
LOC106836782	7
LOC106836783	7
PRM3	8
TNP2	7
SOCS1	2
CLEC16A	1
DEXI	5
NUBP1	2
TEKT5	1
LOC106836762	4
ATF7IP2	5
LOC106836781	8
USP7	3
LOC106836785	1
CARHSP1	8

PMM2	4
TMEM186	4
ABAT	1
METTL22	10
IMMP2L	6
DOCK4	2
ZNF277	2
IFRD1	6
LOC106836795	1
LOC106836802	7
TMEM168	6
LOC106836798	2
GPR85	1
LOC106836800	5
DNAJC22	1
C1QL4	7
TROAP	1
PRPH	5
TUBA1C	2
LOC106836806	2
LOC106836809	2
LOC106836811	2
LMBR1L	8
RHEBL1	2
PRKAG1	3
LOC106836817	1
ARF3	5
FKBP11	5
CCDC65	5
DDX23	2
LOC106836827	5
CCNT1	1
KANSL2	4
LOC106836907	7
ZNF641	1
LOC106836831	8
CCDC184	5
ASB8	4
PFKM	6
SENP1	5
COL2A1	9
TMEM106C	2
HDAC7	2
SLC48A1	2

RPAP3	2
AMIGO2	2
CBX7	1
RPL3	2
LOC106836910	7
SYNGR1	2
LOC106836914	1
RALYL	2
EXOC1	3
PDCL2	5
CLOCK	1
TMEM165	4
SRD5A3	5
LOC106836926	7
KDR	9
PIK3C3	2
CDC123	2
CAMK1D	4
LOC106836936	7
CCDC3	8
OPTN	2
MCM10	5
PCDHB4	5
PCDHB7	1
LOC106836949	1
LOC106836946	6
LOC106836947	1
PCDHB14	1
LOC106836948	1
LOC106836944	5
LOC106836951	5
TAF7	2
LOC106836953	1
LOC106836954	1
LOC106836957	6
DIAPH1	2
HDAC3	2
FCHSD1	2
KIAA0141	4
RNF14	8
GNPDA1	2
NDFIP1	5
LOC106836990	1
ARHGAP26	6

LOC106836983	6
NR3C1	2
LAMC1	2
LAMC2	3
SMG7	4
ARPC5	2
RGL1	6
APOBEC4	5
COLGALT2	1
TSEN15	2
LOC106837001	2
EDEM3	4
FAM129A	7
RNF2	2
TRMT1L	6
SWT1	1
IVNS1ABP	4
LOC106837010	1
LOC106837011	1
SLC35F6	4
CENPA	2
DPYSL5	7
MAPRE3	7
TMEM214	5
AGBL5	5
LOC106837026	1
OST4	2
KHK	2
CGREF1	7
ABHD1	7
PREB	5
PRR30	8
LOC106837036	2
ATRAID	2
CAD	2
SLC30A3	6
DNAJC5G	5
GTF3C2	6
EIF2B4	4
SNX17	5
ZNF513	8
PPM1G	5
LOC106837052	6
NRBP1	2

KRTCAP3	2
IFT172	6
FNDC4	2
LOC106837056	1
ZNF512	4
CCDC121	1
GPN1	2
SUPT7L	3
SLC4A1AP	3
MRPL33	2
RBKS	5
LOC106837068	1
BRE	5
FOSL2	2
PPP1CB	2
SPDYA	5
WDR43	2
CLIP4	1
ALK	1
RHBDD2	6
HIP1	7
LOC106837126	4
LOC106837092	8
NSUN5	2
TRIM50	1
FKBP6	5
BAZ1B	4
BCL7B	2
TBL2	5
MLXIPL	6
DNAJC30	1
WBSCR22	2
STX1A	9
ABHD11	2
WBSCR27	7
WBSCR28	1
LIMK1	2
DNAJC2	3
PMPCB	3
NAPEPLD	9
ARMC10	9
FBXL13	1
FAM185A	1
CCDC146	5

GSAP	1
PTPN12	2
RSBN1L	4
TMEM60	2
PHTF2	2
TMOD3	2
LEO1	2
MAPK6	7
GNB5	2
MYO5C	6
MYO5A	8
ARPP19	7
FAM214A	6
ONECUT1	2
WDR72	1
CHCHD10	2
MMP11	2
SMARCB1	2
LOC106837167	4
TCF21	2
TBPL1	5
SGK1	2
LOC106837336	7
HBS1L	5
AHI1	4
MTFR2	5
BCLAF1	2
MAP7	6
MAP3K5	2
PEX7	5
IFNGR1	2
AKAP12	7
MTHFD1L	2
LOC106837205	2
PCMT1	9
NUP43	2
LATS1	6
KATNA1	10
GINM1	5
PPIL4	3
TAB2	4
UST	1
SASH1	2
STXBP5	2

ADGB	1
RAB32	9
SHPRH	4
UTRN	5
STX11	2
SF3B5	5
PLAGL1	2
ZC2HC1B	1
LTV1	2
PHACTR2	2
FUCA2	2
PEX3	8
ADAT2	2
AIG1	2
HIVEP2	2
LOC106837251	1
LOC106837250	5
LTA4H	2
AMDHD1	2
CCDC38	5
SNRPF	4
NTN4	1
USP44	1
METAP2	5
PELI2	2
TMEM260	1
EXOC5	5
AP5M1	4
SLC35F4	1
LOC106837333	2
GGT7	4
ACSS2	5
GSS	4
TRPC4AP	6
EDEM2	5
EIF6	3
LOC106837276	8
CEP250	6
LOC106837343	7
LOC106837344	7
ERGIC3	2
SPAG4	1
LOC106837283	4
ROMO1	4

RBM39	2
PHF20	2
SCAND1	5
CNBD2	1
EPB41L1	2
AAR2	4
BOD1	4
BNIP1	5
CREBRF	2
ATP6V0E1	4
RPL26L1	2
ERGIC1	2
DUSP1	9
NEURL1B	6
SH3PXD2B	2
UBTD2	9
EFCAB9	1
STK10	2
FBXW11	2
SMIM23	7
NPM1	3
RANBP17	5
LOC106837367	5
DOCK2	6
FAM196B	1
SPDL1	5
SLIT3	1
LOC106837374	7
PANK3	2
LOC106837388	2
RARS	2
WWC1	4
EPHA7	6
GABARAPL1	6
CLEC12A	1
LOC106837414	5
LOC106837424	6
M6PR	5
PHC1	6
RIMKLB	3
APOBEC1	1
GDF3	1
DPPA3	5
LOC106837435	5

SLC2A3	5
FOXJ2	2
NECAP1	4
CLEC4A	1
LOC106837443	8
CLEC4E	1
ZBTB10	6
FABP5	2
LOC106837465	1
FABP12	7
NCKAP5	6
LOC106837477	3
TRAF6	2
COMMD9	2
LDLRAD3	2
TRIM44	2
PAMR1	2
SLC1A2	5
CD44	1
PDHX	6
APIP	2
CAT	2
ABTB2	2
NAT10	4
CAPRIN1	3
LOC106837502	1
LMO2	2
DEPDC7	6
TCP11L1	5
CSTF3	6
PTPRD	2
TMEM261	5
GLDC	5
UHRF2	1
TPD52L3	5
HPS5	4
GTF2H1	4
LDHA	5
LDHC	5
TSG101	3
LOC106837538	1
VTI1A	4
ZDHHC6	3
ACSL5	5

GPAM	2
ADRA2A	1
SHOC2	1
BBIP1	5
PDCD4	2
LOC106837553	5
RBM20	6
LOC106837555	5
SMC3	4
DUSP5	2
MYH9	2
TXN2	3
EIF3D	2
CACNG2	5
IFT27	10
PVALB	4
LOC106837636	1
TEX33	8
TST	2
MPST	2
KCTD17	6
C1QTNF6	1
CYTH4	2
LOC106837588	8
MFNG	1
CARD10	2
CDC42EP1	2
GGA1	6
LGALS1	9
NOL12	6
LOC106837598	2
LOC106837599	1
LOC106837601	4
GCAT	8
ANKRD54	5
EIF3L	3
MICALL1	9
LOC106837606	5
POLR2F	2
SOX10	8
PICK1	1
SLC16A8	1
BAIAP2L2	2
PLA2G6	5

LOC106837614	2
LOC106837615	2
KDELR3	1
DDX17	6
DMC1	1
FAM227A	1
CBY1	5
TOMM22	2
JOSD1	2
GTPBP1	4
SUN2	7
DNAL4	5
NPTXR	1
GRIA4	1
MSANTD4	1
KBTBD3	2
AASDHPPT	2
GUCY1A2	1
LOC106837671	5
IKZF2	2
KCNU1	1
ZNF703	2
ERLIN2	5
PROSC	2
BRF2	4
RAB11FIP1	6
GOT1L1	1
EIF4EBP1	2
GABRB3	4
LOC106837695	2
CKAP2	5
LOC106837696	5
ALG11	4
ATP7B	1
CCDC70	7
WDFY2	6
LOC106837705	1
INTS6	5
RNASEH2B	2
LOC106837715	2
LOC106837716	1
TRIM13	5
SPRYD7	5
KPNA3	2

EBPL	5
CBR4	2
PALLD	5
LOC106837730	1
DDX60	4
CPE	2
MSMO1	2
KLHL2	2
TMEM192	2
LOC106837764	2
1-Mar	1
TMA16	2
TKTL2	5
NPY1R	1
NAF1	4
DPH5	2
SLC30A7	2
VCAM1	2
CDC14A	1
RTCA	5
TRMT13	6
SASS6	4
MFSD14A	7
SLC35A3	1
AGL	2
PLPPR5	6
SNX7	2
ABT1	2
HMGN4	2
LOC106837808	1
LOC106837883	1
LOC106837811	1
LOC106837809	1
LOC106837812	2
LOC106837815	1
LOC106837843	5
LOC106837821	9
LOC106837835	5
LOC106837833	2
LOC106837817	2
LOC106837827	6
LOC106837834	1
LOC106837838	9
LOC106837820	2

LOC106837819	4
LOC106837826	1
LOC106837818	2
TRIM38	2
LOC106837852	4
LOC106837888	4
LOC106837854	1
LRRC16A	6
LOC106837860	1
GMNN	2
LOC106837863	3
ACOT13	2
TDP2	3
NDUFA8	3
MORN5	5
LHX6	7
RBM18	2
CIR1	5
SCRN3	4
GPR155	2
WIPF1	2
CHN1	2
ATF2	2
ATP5G3	2
KIAA1715	1
LOC106837909	2
LOC106837932	6
MAPK7	3
B9D1	5
EPN2	2
SLC5A10	5
PRPSAP2	2
SHMT1	9
SMCR8	1
TOP3A	4
MIEF2	2
FLII	2
ALKBH5	6
DRG2	5
GID4	8
ATPAF2	8
DRC3	5
RAB23	6
BAG2	2

ZNF451	4
KIAA1586	2
DST	2
KIF5B	5
ARHGAP12	2
ZEB1	2
LOC106837960	8
LOC106837961	1
LOC106837972	5
ZNF438	4
SVIL	5
KIAA1462	2
MTPAP	5
MAP3K8	2
LOC106837978	1
LOC106837979	2
COIL	5
SCPEP1	2
LOC106837981	5
AKAP1	7
CPEB2	1
BOD1L1	6
RAB28	8
LOC106838000	1
CRTAP	2
GLB1	2
CCR4	5
TRIM71	2
LOC106838006	7
CNOT10	5
ARHGAP28	1
LAMA1	2
PTPRM	2
MTCL1	6
RAB12	2
LOC106838029	6
TMEM171	2
BTF3	5
ANKRA2	2
UTP15	2
FCHO2	2
TNPO1	4
LOC106838062	7
PTCD2	4

MRPS27	4
MAP1B	2
MCCC2	2
BDP1	6
LOC106838049	3
NAIP	6
LOC106838053	4
OCLN	2
RAD17	4
AK6	5
TAF9	5
GCSAML	2
LOC106838082	1
PDZRN3	2
LOC106838137	6
NCK1	3
IL20RB	1
DBR1	4
ARMC8	1
NME9	7
MRAS	5
ESYT3	4
CEP70	5
FAIM	4
PIK3CB	2
MRPS22	5
COPB2	2
RBP1	2
NMNAT3	6
LOC106838105	2
TRIM42	7
SLC25A36	2
LOC106838113	8
PXYLP1	6
LOC106838115	1
ZBTB38	2
RASA2	2
RNF7	3
ATP1B3	7
TFDP2	3
GK5	6
XRN1	2
ATR	6
TRPC1	4

PCOLCE2	2
PAQR9	1
U2SURP	3
CHST2	6
LOC106838134	1
LOC106838133	2
FAM133A	2
NAP1L3	2
HMBOX1	1
KIF13B	6
MSRA	8
LOC106838193	7
LOC106838190	7
LOC106838194	1
LOC106838169	8
SOX7	7
PINX1	3
MTMR9	6
LOC106838174	2
MRPL17	3
DCHS1	6
TAF10	10
ILK	3
RRP8	1
DNHD1	1
TIMM10B	5
ARFIP2	4
TRIM3	2
APBB1	2
LIG4	6
ABHD13	6
TNFSF13B	2
MYO16	2
IRS2	2
COL4A1	2
COL4A2	2
RAB20	2
CARKD	2
CARS2	2
ING1	5
ANKRD10	6
SPACA7	1
LOC106838208	6
KYNU	2

LOC106838217	5
GTDC1	4
LOC106838220	5
ELAVL4	2
EDAR	7
LOC106838225	1
LIG1	4
LOC106838234	1
ZNF114	7
CCDC114	5
EMP3	2
TMEM143	1
SYNGR4	5
KDELR1	4
GRWD1	2
CYTH2	1
SPACA4	5
RPL18	2
SPHK2	5
DBP	4
CA11	1
RASIP1	1
IZUMO1	1
BCAT2	1
PLEKHA4	2
PPP1R15A	4
TULP2	5
NUCB1	4
DHDH	2
FTL	2
GYS1	1
RUVBL2	5
LOC106838274	1
LOC106838272	1
SNRNP70	4
LIN7B	9
PPFIA3	7
TRPM4	6
LOC106838313	1
LOC106838281	1
LOC106838283	5
CD37	8
TEAD2	1
DKKL1	5

CCDC155	5
SLC17A7	1
PIH1D1	4
ALDH16A1	2
FLT3LG	6
RPL13A	2
RPS11	2
RNF219	4
LOC106838298	6
DUSP21	9
FUNDC1	2
EFHC2	5
MAOA	7
LOC106838332	7
LOC106838357	8
MGAT4C	7
TMEM263	5
MTERF2	2
CRY1	6
PWP1	4
PRDM4	4
RTCB	2
BPIFC	7
FBXO7	9
LOC106838345	1
TIMP3	9
LOC106838356	4
MTHFS	4
ZFAND6	9
FAH	2
ITM2A	2
LOC106838359	4
FAM46D	1
LOC106838369	2
BRWD3	1
SH3BGRL	2
LOC106838364	1
PRSS45	5
PRSS46	8
PRSS50	1
RTP3	2
LTF	9
FYCO1	5
LZTFL1	5

SACM1L	2
LOC106838399	9
LIMD1	6
EXOSC7	3
ZDHHC3	10
TMEM42	3
KIF15	5
KIAA1143	8
LOC106838414	4
LOC106838413	1
LOC106838422	2
ZNF445	2
TCAIM	5
TOPAZ1	2
LOC106838427	1
LOC106838428	3
SMARCAL1	6
RPL37A	2
IGFBP2	2
TNP1	7
TNS1	9
LOC106838440	1
ARPC2	3
AAMP	5
LOC106838446	3
TMBIM1	2
CATIP	5
USP37	6
RQCD1	4
PLCD4	1
ZNF142	6
LOC106838454	2
RNF25	5
STK36	6
TTLL4	1
LOC106838458	2
WNT6	1
LOC106838513	1
FBXO38	6
ADRB2	5
ABLIM3	2
AFAP1L1	9
GRPEL2	4
PCYOX1L	4

CSNK1A1	3
PPARGC1B	1
PDE6A	7
SLC26A2	1
HMGXB3	4
PDGFRB	1
TCOF1	2
CD74	2
RPS14	2
NDST1	4
RBM22	2
DCTN4	2
SMIM3	6
ZNF300	4
LOC106838525	2
MRPS5	3
ZNF514	6
ZNF2	1
LOC106838523	5
LOC106838524	2
NPHP1	1
BUB1	5
LOC106838540	8
BCL2L11	4
LOC106838644	5
ANAPC1	2
TMEM87B	2
ZC3H8	2
ZC3H6	6
TTL	2
POLR1B	2
CHCHD5	10
SLC20A1	2
CKAP2L	5
IL1A	7
PAX8	6
LOC106838649	7
RPIA	2
EIF2AK3	2
TEX37	7
FOXI3	1
SMYD1	1
KRCC1	5
CD8B	2

RMND5A	4
RNF103	6
CHMP3	5
KDM3A	1
MRPL35	4
IMMT	3
PTCD3	9
POLR1A	4
ST3GAL5	2
SFTPB	7
USP39	2
LOC106838599	5
TMEM150A	2
RNF181	5
VAMP5	2
VAMP8	9
MAT2A	2
CAPG	2
ELMOD3	5
RETSAT	1
TGOLN2	3
TCF7L1	2
KCMF1	5
TRABD2A	5
DNAH6	5
SUCLG1	2
LOC106838665	5
PIGC	5
SUCO	5
PRDX6	9
LOC106838673	5
SLC9C2	5
ANKRD45	5
LOC106838675	5
KLHL20	2
CENPL	1
ZBTB37	1
SERPINC1	1
RC3H1	6
LOC106838686	1
COL3A1	1
COL5A2	6
WDR75	2
SLC40A1	2

ASNSD1	2
ANKAR	1
OSGEPL1	6
ORMDL1	4
LOC106838709	2
PKIB	7
TRDN	4
RNF217	6
TPD52L1	7
HDDC2	5
LOC106838738	1
RBMX2	2
SLC25A14	4
ZNF280C	2
TFAM	8
UBE2D1	2
CISD1	5
IPMK	4
FBXL17	1
LOC106838744	7
EFNA5	2
LOC106838750	1
JAK1	2
LOC106838756	2
AK4	2
DNAJC6	2
LOC106838760	6
LEPROT	2
SGIP1	7
WDR78	5
MIER1	2
SLC35D1	5
MAP7D3	2
FHL1	7
SLC9A6	2
LOC106838791	2
RPGR	3
LOC106838792	7
CFAP47	1
NEU3	6
SPCS2	3
XRRA1	5
RNF169	1
CHRDL2	6

LOC106838812	1
FAM177A1	5
PPP2R3C	5
KIAA0391	6
PSMA6	2
NFKBIA	2
RALGAPA1	5
BRMS1L	1
MBIP	2
NKX2-1	2
LOC106838809	4
FAM46A	2
IBTK	4
TPBG	2
UBE3D	4
DOPEY1	6
PGM3	2
RWDD2A	5
ME1	6
PRSS35	1
SNAP91	6
LOC106838849	2
LOC106838851	5
ULK2	1
AKAP10	1
LOC106838855	6
ZSWIM7	3
LOC106838860	5
NCOR1	3
PIGL	1
CENPV	6
UBB	5
ZNF287	5
IFT57	5
CD47	5
BBX	6
CCDC54	8
LOC106838871	9
CBLB	9
ALCAM	2
NFKBIZ	4
NXPE3	4
CEP97	1
LOC106838908	1

RPL24	3
ZBTB11	2
PCNP	4
TRMT10C	4
LOC106838885	5
SENP7	6
ABI3BP	7
TFG	5
TMEM45A	2
TOMM70A	3
NIT2	1
TBC1D23	2
LOC106838896	5
CMSS1	2
FILIP1L	1
RSPO3	5
RNF146	5
ECHDC1	5
PDK4	2
PON2	2
PPP1R9A	5
PEG10	2
SGCE	2
COL1A2	2
BET1	5
GNG11	2
TFPI2	2
TMEM243	2
DMTF1	4
LOC106838951	8
SEMA3D	1
LOC106838952	3
EQTN	1
LOC106838969	8
LOC106838957	1
IFT74	5
PLAA	4
CAAP1	3
LOC106838965	6
TUSC1	2
RIPK2	2
OSGIN2	4
NBN	3
DECR1	2

CALB1	7
TMEM64	8
LOC106838978	5
TMEM55A	2
OTUD6B	1
LRRC69	1
LOC106838997	2
LOC106838990	2
LOC106838991	1
LOC106838992	3
ZNF654	6
CGGBP1	9
ACSM3	1
ERI2	1
LOC106839006	1
UMOD	2
IQCK	5
KNOP1	7
LOC106839013	5
CCP110	3
GDE1	9
TMC5	1
TMC7	1
COQ7	2
SYT17	2
CLEC19A	2
LOC106839027	6
SPATA18	7
USP46	2
FIP1L1	2
CHIC2	2
LOC106839057	1
KIT	2
PGLYRP2	2
RASAL3	2
WIZ	6
AKAP8L	6
LOC106839066	1
AKAP8	2
BRD4	2
EPHX3	5
ILVBL	2
SYDE1	1
CCDC105	5

GATB	6
FAM160A1	4
SH3D19	4
LOC106839079	1
LOC106839078	2
RPS3A	3
LOC106839088	4
LRBA	4
MAB21L2	5
SLC9A1	2
WDTC1	8
TMEM222	7
SYTL1	2
CD164L2	7
GPR3	8
WASF2	2
AHDC1	2
IFI6	9
FAM76A	1
STX12	4
PPP1R8	2
RPA2	5
EYA3	6
DNAJC8	2
ATPIF1	5
SESN2	6
MED18	2
PHACTR4	2
RCC1	7
TRNAU1AP	1
RAB42	9
TAF12	3
GMEB1	2
YTHDF2	4
EPB41	6
SRSF4	4
MECR	2
PTPRU	4
LOC106839142	1
PPP2R5E	5
LOC106839146	6
WDR89	1
SGPP1	1
SYNE2	5

ESR2	4
LOC106839162	5
STK17A	4
LOC106839157	2
BLVRA	2
STMN2	2
ZC2HC1A	1
LOC106839247	1
PKIA	2
PEX2	2
ZFHX4	2
UBE2W	1
TCEB1	5
TMEM70	2
JPH1	6
LOC106839187	8
STAU2	6
RDH10	9
RPL7	2
LOC106839193	5
SBSPON	2
TERF1	5
LOC106839254	1
PKD2L2	6
FAM13B	6
NME5	5
BRD8	5
KIF20A	5
CDC23	6
GFRA3	4
CDC25C	1
LOC106839214	7
FAM53C	1
KDM3B	4
REEP2	2
EGR1	2
ETF1	3
HSPA9	2
LOC106839222	1
CTNNA1	4
SIL1	5
MATR3	2
LOC106839228	7
PAIP2	9

SPATA24	10
DNAJC18	5
ECSCR	9
MAST4	4
MRPL44	2
SERPINE2	2
CUL3	10
DOCK10	2
POR	2
STYXL1	5
MDH2	2
HSPB1	2
YWHAG	2
SSC4D	1
ZP3	1
DTX2	4
LOC106839282	2
POLR2J	3
LRWD1	5
ALKBH4	5
ORAI2	8
PRKRIP1	6
LOC106839288	8
CUX1	4
LOC106839293	4
IFT22	5
LOC106839295	1
FIS1	2
ZNHIT1	2
AP1S1	6
TRIM56	2
LOC106839302	8
UFSP1	1
SRRT	5
SLC12A9	2
ZAN	6
POP7	2
GIGYF1	2
GNB2	2
TFR2	1
MOSPD3	2
PCOLCE	1
FBXO24	1
LRCH4	5

SAP25	2
AGFG2	6
TSC22D4	1
PPP1R35	2
MEPCE	5
ZCWPW1	2
TRMT12	1
RNF139	5
TATDN1	2
NDUFB9	5
MTSS1	2
SQLE	6
KIAA0196	2
NSMCE2	3
FAM84B	2
LOC106839381	8
PDCL3	2
RPL31	2
TBC1D8	2
CNOT11	3
RNF149	2
RFX8	5
MAP4K4	4
MFSD9	7
TMEM182	7
CDS1	5
WDFY3	4
ARHGAP24	1
LOC106839427	1
LOC106839423	1
ADIPOR2	2
DCP1B	5
CECR5	1
CECR2	9
SLC25A18	1
ATP6V1E1	2
BCL2L13	8
BID	2
MICAL3	4
TUBA8	7
LOC106839448	7
LOC106839447	1
USP18	2
LOC106839449	5

LOC106839451	1
LOC106839450	1
TARSL2	2
TM2D3	3
SNRPA1	2
VIMP	5
CHSY1	2
ASB7	6
LINS1	5
CERS3	6
LYSMD4	6
MEF2A	6
LRRC28	10
LOC106839482	6
PTGER2	6
PTGDR	2
NID2	2
LOC106839488	3
LOC106839491	1
FRMD6	2
TMX1	2
PYGL	2
SAV1	2
ATL1	5
MAP4K5	2
ATP5S	4
L2HGDH	2
SOS2	5
VCPKMT	2
ARF6	2
NEMF	2
KLHDC2	4
KLHDC1	2
POLE2	6
DNAAF2	6
SND1	1
LRRC4	6
RBM28	2
HILPDA	2
FAM71F2	7
FAM71F1	8
CALU	2
CCDC136	10
FLNC	2

ATP6V1F	5
LOC106839534	7
KCP	1
TNPO3	3
TSPAN33	2
SMO	5
AHCYL2	1
STRIP2	6
LOC106839544	1
NRF1	1
UBE2H	5
ZC3HC1	5
KLHDC10	3
TMEM209	6
SSMEM1	5
CPA5	1
CEP41	5
MEST	2
COPG2	1
C1GALT1	5
MIOS	2
RPA3	2
UMAD1	1
GLCCI1	5
ICA1	1
LOC106839578	1
BRF1	1
BTBD6	5
NUDT14	2
JAG2	2
CDCA4	4
LOC106839592	5
CEP170B	2
ZBTB42	5
AKT1	2
SIVA1	2
ADSSL1	5
KIF26A	2
TDRD9	6
LOC106839606	9
LOC106839608	5
PPP1R13B	1
ZFYVE21	2
XRCC3	2

KLC1	4
APOPT1	8
BAG5	8
MARK3	2
EIF5	2
LOC106839624	2
TNFAIP2	7
EXOC3L4	5
CDC42BPB	4
TRAF3	2
RCOR1	6
TECPR2	1
CINP	5
ZNF839	6
MOK	5
WDR20	8
HSP90AA1	3
LOC106839641	8
DYNC1H1	4
PPP2R5C	1
LOC106839697	7
LOC106839652	2
DLK1	2
WDR25	6
WARS	3
SLC25A29	9
YY1	4
EVL	9
CCNK	5
SETD3	2
BCL11B	2
LOC106839702	1
SIPA1L2	4
MAP10	5
NTPCR	2
PCNXL2	6
LOC106839676	5
KCNK1	2
LOC106839706	2
ACSL4	2
NXT2	2
IRS4	2
PSMD10	2
VSIG1	7

LOC106839724	5
TSC22D3	2
PRPS1	2
MORC4	2
TBC1D8B	2
LOC106839736	1
MED30	2
DIRAS2	1
AUH	4
NFIL3	2
ROR2	2
SPTLC1	6
LOC106839745	5
NFIA	6
TM2D1	3
INADL	2
KANK4	1
USP1	2
DOCK7	2
LOC106839777	3
RNFT2	9
FBXW8	1
TESC	2
FBXO21	2
RFC5	2
WSB2	2
PEBP1	2
LOC106839775	5
TAOK3	5
SUDS3	3
LOC106839815	8
LOC106839782	2
LOC106839784	5
MGAT2	3
EMC1	2
MRTO4	2
LOC106839787	5
LOC106839786	2
CAPZB	7
MINOS1	3
TMCO4	6
OTUD3	2
PLA2G2A	1
PLA2G5	1

PLA2G2C	6
UBXN10	5
LOC106839810	1
LARS	5
RBM27	5
TCERG1	2
PPP2R2B	7
STK32A	4
DPYSL3	2
SPINK5	7
RGCC	3
NAA16	4
MTRF1	2
KBTBD7	4
LOC106839857	6
WBP4	2
ELF1	2
SUGT1	5
LECT1	2
PCDH8	7
STIM2	1
ADGRG6	2
VTA1	2
LOC106839871	2
TEX29	1
TUBGCP3	2
MCF2L	2
F10	1
PCID2	2
FAM98A	2
LTBP1	1
TTC27	5
BIRC6	2
YIPF4	3
NLRC4	1
SLC30A6	6
SPAST	5
ISL1	4
PARP8	5
FAM46B	5
TRNP1	4
NUDC	3
NR0B2	1
GPATCH3	6

GPN2	1
ZDHHC18	2
PIGV	1
ARID1A	6
DHDDS	5
ZNF683	1
AIM1L	2
CD52	6
UBXN11	8
SH3BGRL3	5
CEP85	2
CATSPER4	1
LOC106839926	1
ZNF593	2
PDIK1L	2
STMN1	5
PAQR7	9
AUNIP	1
MTFR1L	5
MAN1C1	2
LDLRAP1	2
TMEM57	3
RHCE	6
TMEM50A	2
RSRP1	1
LOC106839952	3
SYF2	3
RUNX3	2
CLIC4	5
SRRM1	4
NCMAP	2
RCAN3	2
NIPAL3	2
STPG1	1
SRSF10	1
PNRC2	8
FUCA1	6
HMGCL	2
GALE	2
LYPLA2	2
PITHD1	2
TCEB3	2
RPL11	2
ID3	2

E2F2	7
ASAP3	9
LOC106839985	7
TCEA3	2
ZNF436	4
LOC106839988	8
HNRNPR	4
HTR1D	5
LUZP1	1
KDM1A	2
LOC106840059	7
ZBTB40	6
CDC42	2
LOC106840006	2
LOC106840061	8
USP48	2
RAP1GAP	2
ECE1	2
EIF4G3	5
HP1BP3	2
KIF17	7
LOC106840021	1
DDOST	5
PINK1	5
CDA	2
MUL1	6
CAMK2N1	2
LOC106840029	7
VWA5B1	1
LOC106840031	1
CCDC152	1
SEPP1	2
LOC106840035	1
HMX1	2
LOC106840087	1
CPZ	2
TRMT44	6
ACOX3	5
SH3TC1	8
ABLIM2	1
AFAP1	7
GRPEL1	2
TADA2B	6
CCDC96	5

TBC1D14	2
KIAA0232	4
BLOC1S4	3
LOC106840080	6
LOC106840082	6
LOC106840083	2
OXR1	8
LOC106840091	1
ANGPT1	1
EIF3E	2
EMC2	3
FGF13	2
ATP11C	2
LOC106840107	1
LOC106840108	2
LOC106840109	7
LOC106840123	2
ZBTB25	7
ZBTB1	6
HSPA2	5
LOC106840114	5
COL14A1	4
MRPL13	3
LOC106840121	1
SH3BP5L	2
ZNF672	4
ZNF692	3
LOC106840130	1
PGBD2	6
FAM71C	7
CCDC125	9
CDK7	5
MRPS36	5
CENPH	3
CCNB1	5
SLC30A5	4
PIK3R1	2
ZSWIM2	5
FAM171B	4
CACHD1	2
UBE2U	5
PGM1	9
EFCAB7	5
ITGB3BP	5

ALG6	2
ATG4C	5
KHDRBS3	5
LOC106840173	1
ZFAT	8
CLDN34	1
WWC3	2
CLCN4	2
MID1	9
LOC106840188	2
LOC106840202	5
LAYN	1
SIK2	6
PPP2R1B	5
ALG9	2
LOC106840208	7
FDXACB1	5
CRYAB	4
DIXDC1	1
DLAT	2
PIH1D2	5
LOC106840216	3
TIMM8B	5
SDHD	2
IL18	2
TEX12	2
BCO2	6
PTS	5
NCAM1	2
TTC12	5
LOC106840339	5
ZW10	2
USP28	2
ZBTB16	6
LOC106840238	7
RBM7	6
REXO2	2
CADM1	5
LOC106840342	5
LOC106840247	1
BUD13	6
ZPR1	5
APOA4	9
APOC3	7

APOA1	2
SIK3	9
PAFAH1B2	2
SIDT2	6
RNF214	2
CEP164	1
FXYD6	2
LOC106840270	1
TMPRSS4	8
JAML	4
MPZL3	5
CD3G	2
UBE4A	4
ATP5L	3
KMT2A	4
LOC106840284	1
IFT46	3
ARCN1	5
PHLDB1	4
TREH	10
DDX6	2
BCL9L	7
UPK2	1
FOXR1	2
CCDC84	4
RPS25	3
TRAPPC4	3
SLC37A4	5
HYOU1	3
VPS11	6
HMBS	4
LOC106840300	5
DPAGT1	4
C2CD2L	6
HINFP	2
CBL	6
MCAM	2
LOC106840310	1
RNF26	5
USP2	5
THY1	2
OAF	2
FAM213A	2
DYDC2	7

DYDC1	5
FAT1	2
SORBS2	1
CCDC110	5
GDPD4	1
CAPN5	1
LOC106840386	6
ACER3	5
TSKU	2
EMSY	6
LOC106840393	8
PRKRIR	2
WNT11	2
UVRAG	4
DGAT2	9
MAP6	1
SERPINH1	2
GDPD5	2
LOC106840408	2
RPS3	2
ARRB1	1
TPBGL	9
DHX15	4
CCDC149	5
SEPSECS	2
LOC106840429	1
PI4K2B	8
ZCCHC4	4
ANAPC4	3
SEL1L3	4
LOC106840446	1
SMIM20	3
RBPJ	2
TBC1D19	1
RPP38	5
MEIG1	5
DCLRE1C	1
SUV39H2	1
HSPA14	2
LOC106840454	2
FAM107B	2
FRMD4A	2
PRPF18	4
BEND7	5

SEPHS1	5
LOC106840464	6
LOC106840463	5
LOC106840476	7
LOC106840478	3
LOC106840489	5
LOC106840479	2
ESX1	2
FAM199X	2
TMSB15B	2
ATP5J2	3
CPSF4	4
BUD31	3
PDAP1	2
ARPC1B	2
ARPC1A	2
SMURF1	1
TRRAP	2
NPTX2	2
BAIAP2L1	4
LOC106840503	3
LOC106840504	8
TECPR1	6
LMTK2	6
CCZ1	1
LOC106840508	5
PMS2	2
AIMP2	3
EIF2AK1	2
ANKRD61	5
USP42	5
FAM220A	7
LOC106840515	7
RAC1	2
DAGLB	2
KDELR2	5
ZDHHC4	5
LOC106840531	4
ZNF12	8
SLC4A3	7
STK11IP	4
INHA	2
OBSL1	2
TMEM198	8

CHPF	1
GMPPA	5
DES	2
DNPEP	4
PTPRN	2
LOC106840558	9
STK16	9
GLB1L	10
ANKZF1	2
ATG9A	5
ABCB6	3
ZFAND2B	3
FAM134A	3
CNPPD1	5
NHEJ1	2
LOC106840568	2
CFAP65	1
LOC106840587	1
EYA4	4
RPS12	2
LOC106840577	6
HACD3	2
VWA9	2
DENND4A	2
RAB11A	3
MEGF11	6
DIS3L	6
TIPIN	2
MAP2K1	2
SNAPC5	9
RPL4	2
ZWILCH	2
AAGAB	6
IQCH	5
LOC106840613	5
MAP2K5	1
SKOR1	1
ZMYND11	4
DIP2C	2
LARP4B	4
GTPBP4	6
LOC106840621	5
WDR37	6
PFKP	5

PITRM1	2
KLF6	2
AP1G2	2
THTPA	8
ZFHX2	1
NGDN	2
LOC106840636	4
SLC22A17	2
LOC106840641	4
LOC106840642	1
LOC106840645	7
HOMEZ	1
RNF212B	2
ACIN1	3
LOC106840648	2
PSMB5	2
AJUBA	6
HAUS4	2
PRMT5	6
RBM23	6
REM2	6
LRP10	2
MRPL52	3
SLC7A7	4
LOC106840664	3
ABHD4	9
DAD1	5
LOC106840669	6
LOC106840680	1
TRAPPC13	6
SGTB	3
NLN	4
ERBB2IP	4
SREK1	4
TMED3	2
LOC106840812	5
CTSH	2
LOC106840816	5
LOC106840815	5
ADAMTS7	2
TBC1D2B	2
CIB2	2
IDH3A	2
DNAJA4	7

WDR61	5
CRABP1	5
IREB2	4
HYKK	1
PSMA4	9
CHRNA5	1
CHRNA3	6
LOC106840837	5
UBE2Q2	5
FBXO22	2
LOC106840843	6
LOC106840854	1
TMEM132B	7
LOC106840850	2
KLK5	2
NDUFA3	9
TFPT	4
PRPF31	4
CNOT3	4
LENG1	3
MBOAT7	2
TSEN34	2
RPS9	2
LOC106840877	7
LOC106840953	1
CDC42EP5	2
LENG9	1
LENG8	1
LOC106840889	5
TTYH1	1
GP6	2
PPP1R12C	2
TNNT1	2
SYT5	2
TMEM86B	3
PPP6R1	1
HSPBP1	3
KMT5C	9
FAM71E2	1
IL11	1
TMEM190	1
RPL28	2
UBE2S	3
ISOC2	3

ZNF628	7
NAT14	1
ZNF865	2
LOC106840950	2
CCDC106	6
EPN1	4
GATSL2	2
LOC106840980	6
NCF1	7
GTF2I	4
GTF2IRD1	2
CLIP2	2
RFC2	9
EIF4H	7
LOC106841001	2
LOC106841000	3
HSPA4	2
ZCCHC10	4
LOC106841006	7
AFF4	7
LOC106841008	10
SHROOM1	5
SOWAHA	6
LOC106841013	1
KIF3A	3
IL13	9
RAD50	2
IL5	2
IRF1	2
LOC106841020	7
PDLIM4	2
P4HA2	2
ACSL6	5
MEIKIN	5
OTOL1	8
NMD3	4
B3GALNT1	2
PPM1L	9
POU6F2	1
YAE1D1	5
RALA	2
LOC106841048	4
CDK13	4
MPLKIP	6

PPP2R2D	2
BNIP3	2
STK32C	2
LRRC27	1
PWWP2B	1
INPP5A	2
NKX6-2	2
CFAP46	5
UTF1	2
LOC106841076	7
TUBGCP2	2
ZNF511	3
CALY	2
FUOM	10
ECHS1	2
PAOX	5
MTG1	2
LOC106841090	7
CDC14B	9
HABP4	7
ZNF367	4
SLC35D2	1
ERCC6L2	6
PTCH1	2
LOC106841118	2
LOC106841111	1
LOC106841124	5
APPBP2	3
PPM1D	5
BCAS3	8
LOC106841129	7
BRIP1	4
MED13	6
RNFT1	5
RPS6KB1	2
TUBD1	2
VMP1	2
PTRH2	4
CLTC	4
LOC106841142	1
SCAMP1	4
BHMT	5
JMY	4
HOMER1	4

PAPD4	4
CMYA5	6
MTX3	2
THBS4	2
SERINC5	6
SPZ1	7
ZFYVE16	4
LOC106841153	1
FAM151B	1
DHFR	2
MSH3	6
RASGRF2	1
ZCCHC9	6
ACOT12	1
SSBP2	4
ATG10	5
RPS23	2
ATP6AP1L	4
LOC106841166	1
LOC106841167	1
TMEM167A	2
XRCC4	2
VCAN	4
EDIL3	1
LOC106841203	5
RASA1	2
CCNH	3
TMEM161B	4
MEF2C	5
CETN3	2
MBLAC2	2
POLR3G	2
LYSMD3	4
ADGRV1	1
ARRDC3	2
LOC106841223	6
NR2F1	6
FAM172A	6
POU5F2	5
FRYL	6
ZAR1	2
SLC10A4	2
SLAIN2	5
TEC	2

NFXL1	4
ATP10D	2
COMMD8	4
LOC106841260	1
LOC106841238	5
GABRA2	2
GNPDA2	6
GUF1	2
BEND4	2
SLC30A9	4
TMEM33	4
LIMCH1	2
UCHL1	9
NSUN7	5
LOC106841282	6
RBM47	3
N4BP2	7
PDS5A	2
UBE2K	5
LOC106841242	1
SMIM14	10
LIAS	9
RPL9	2
KLB	1
LOC106841231	6
SUOX	1
LOC106841299	1
RAB5B	7
CDK2	1
PMEL	1
DGKA	5
PYM1	5
LOC106841306	1
DNAJC14	5
ORMDL2	5
SARNP	2
CD63	2
BLOC1S1	2
ITGA7	2
SIGLEC15	10
UBR1	1
TTBK2	6
CDAN1	4
STARD9	1

HAUS2	5
LRRC57	7
SNAP23	1
ZNF106	6
GANC	5
TMEM87A	2
VPS39	2
PLA2G4D	1
EHD4	2
MAPKBP1	6
MGA	4
RPAP1	2
ITPKA	6
RTF1	4
NDUFAF1	2
NUSAP1	5
OIP5	6
LOC106841361	5
CHP1	9
EXD1	3
INO80	6
VPS18	6
RHOV	1
SPINT1	2
ZFYVE19	2
UBAP1	5
KIF24	1
NUDT2	2
KIAA1161	1
LOC106841397	7
FAM219A	1
DNAI1	5
ENHO	2
CNTFR	2
RPP25L	5
DCTN3	5
SIGMAR1	2
GALT	7
LOC106841407	1
CCL21	6
FAM205A	1
MCPH1	6
AGPAT5	5
LOC106841386	8

LOC106841426	7
LOC106841429	2
COMMD1	5
CCT4	3
FAM161A	5
LOC106841438	5
XPO1	2
USP34	6
AHSA2	3
LOC106841431	1
KIAA1841	1
ZMYM5	2
LOC106841427	10
LOC106841446	5
ZMYM2	6
GJB6	6
NOL6	7
AQP3	2
AQP7	1
NFX1	2
CHMP5	9
BAG1	7
LOC106841462	5
B4GALT1	6
SMU1	2
DNAJA1	5
APTX	2
NDUFB6	4
LOC106841470	5
TOPORS	4
DDX58	2
ACO1	6
LOC106841455	1
LOC106841479	6
ABCC12	5
LONP2	3
SIAH1	4
LOC106841477	8
N4BP1	4
LOC106841478	7
ZNF423	9
PGRMC1	2
LOC106841501	5
SLC25A5	2

LOC106841504	2
UBE2A	5
NKRF	2
SEPT6	2
RPL39	2
UPF3B	3
RNF113A	5
TYK2	7
ICAM3	7
RAVER1	6
FDX1L	5
ZGLP1	2
ICAM5	1
ICAM4	6
MRPL4	2
DNMT1	5
EIF3G	2
ANGPTL6	4
LOC106841527	2
COL5A3	1
LOC106841498	1
BFSP1	2
POLR2D	2
AMMECR1L	2
SAP130	5
UGGT1	4
HS6ST1	2
POU2F1	2
RCSD1	2
MPZL1	2
ADCY10	5
MPC2	5
DCAF6	1
GPR161	6
TIPRL	5
SFT2D2	2
ATP1B1	2
LOC106841567	5
AP1S2	2
ZRSR2	2
PIR	2
PIGA	4
MOSPD2	2
GEMIN8	3

RNF144B	9
DEK	2
KDM1B	2
TPMT	2
NHLRC1	1
KIF13A	2
LOC106841600	6
FAM175B	4
METTL10	6
LHPP	1
OAT	2
ZNF169	6
LOC106841608	8
LOC106841615	5
LOC106841626	4
CDK20	6
SPIN1	2
CKS2	2
SECISBP2	9
SEMA4D	2
LOC106841639	5
MRPL15	2
LOC106841640	6
LOC106841638	1
LOC106841644	6
LOC106841643	7
SOX17	2
RP1	2
TGS1	2
RPS20	9
PLAG1	4
CHCHD7	5
LOC106841659	2
PENK	2
IMPAD1	5
FAM110B	2
UBXN2B	6
LOC106841669	1
SDCBP	2
NSMAF	4
TOX	9
CA8	2
KIAA1211	2
AASDH	2

PPAT	2
PAICS	2
SRP72	4
ARL9	7
THEGL	1
HOPX	2
SPINK2	5
REST	2
NOA1	4
POLR2B	2
IGFBP7	9
CRYBA4	6
TPST2	6
TFIP11	6
SRRD	3
HPS4	6
ASPHD2	7
SEZ6L	1
MYO18B	1
ADRBK2	3
KIAA1671	4
LOC106841714	1
LOC106841732	4
LOC106841731	2
LOC106841735	1
LOC106841736	1
LOC106841727	5
LOC106841715	3
LOC106841716	1
CDR2	3
POLR3E	4
EEF2K	2
VWA3A	1
PDZD9	5
LOC106841746	5
LOC106841718	5
LOC106841750	5
CRYM	2
TMEM159	2
LOC106841719	1
LMCD1	1
RAD18	5
SRGAP3	5
LOC106841769	1

THUMPD3	3
SETD5	2
ACSS1	2
APMAP	6
LOC106841778	8
LOC106841791	2
LOC106841779	9
LOC106841792	7
CST8	7
LOC106841793	9
LOC106841794	9
CSTL1	7
NAPB	2
GZF1	4
NXT1	3
BFSP2	1
TMEM108	4
NPHP3	1
UBA5	4
ACAD11	1
DNAJC13	4
MRPL3	2
NUDT16	8
DDX18	2
LOC106841826	7
RALB	4
TMEM185B	5
EPB41L5	2
PTPN4	6
SLC15A4	2
GLT1D1	8
LOC106841839	2
FZD10	2
PIWIL1	5
STX2	1
RAN	3
LY6E	2
LOC106841942	2
ZFP41	6
GLI4	5
ZNF696	5
TOP1MT	3
ZC3H3	5
MROH6	2

NAPRT	1
EEF1D	7
TIGD5	1
TSTA3	5
LOC106841911	6
ZNF623	4
LOC106841932	1
CCDC166	1
MAPK15	1
SCRIB	6
PUF60	2
PLEC	2
PARP10	1
GRINA	5
SPATC1	7
LOC106841886	1
OPLAH	9
EXOSC4	4
LOC106841927	6
GPAA1	2
LOC106841912	2
SHARPIN	5
MAF1	2
LOC106841922	1
LOC106841855	1
LOC106841933	1
MROH1	1
BOP1	2
HSF1	3
LOC106841935	1
DGAT1	5
TMEM249	1
FBXL6	2
SLC52A2	2
ADCK5	4
CPSF1	2
VPS28	2
TONSL	2
CYHR1	5
KIFC2	4
PPP1R16A	1
GPT	4
MFSD3	9
RECQL4	4

LRRC14	5
LRRC24	8
ARHGAP39	1
ZNF251	6
COMMD5	5
ZNF7	2
RPL8	2
ZNF34	2
ZNF16	2
LOC106841951	1
LOC106841959	1
LOC106841956	1
LOC106841957	6
CYTIP	2
GPD2	8
NR4A2	9
CENPW	3
TRMT11	1
HINT3	2
NCOA7	4
HEY2	2
LOC106842001	6
LOC106841973	3
LOC106841976	7
LOC106842003	1
MSANTD1	6
LOC106842004	6
RGS12	2
HGFAC	8
LRPAP1	5
EZR	2
RSPH3	5
LOC106842013	7
SOD2	2
WTAP	4
ACAT2	2
TCP1	5
MRPL18	3
PNLDC1	5
IGF2R	6
LOC106842044	1
PLG	1
MAP3K4	4
PARK2	6

PACRG	5
LOC106842054	6
QKI	3
LOC106842056	5
PDE10A	1
LOC106842058	2
SFT2D1	5
MPC1	3
RPS6KA2	6
RNASET2	2
FGFR1OP	3
LOC106842011	5
MLLT4	2
KIF25	5
FRMD1	1
LOC106842075	5
DACT2	2
TACR3	2
CXXC4	2
PPA2	5
INTS12	9
GSTCD	1
PIK3AP1	2
TM9SF3	5
ZNF518A	4
LOC106842106	1
CCNJ	4
CC2D2B	1
ENTPD1	1
TCTN3	6
ALDH18A1	2
SORBS1	6
LOC106842113	1
PDLIM1	2
LOC106842117	2
GPC3	2
GPC4	2
USP26	1
MBNL3	2
STK26	2
NFAT5	6
NQO1	2
NOB1	2
WWP2	7

PSMD7	5
LOC106842140	1
LOC106842141	6
ZFHX3	4
LOC106842143	5
NSUN3	6
ARL13B	5
PROS1	2
MACROD2	5
FLRT3	4
TMEM45B	5
NFRKB	6
APLP2	5
ST14	2
ZBTB44	2
LOC106842160	1
SNX19	4
LOC106842172	7
MORC1	2
GUCA1C	6
LOC106842174	4
DZIP3	3
RABL2A	5
TMEM19	4
LOC106842185	1
THAP2	5
ZFC3H1	6
LOC106842181	1
TSPAN8	1
PTPRR	1
CNOT2	3
RAB3IP	2
CCT2	3
FRS2	4
YEATS4	2
MED13L	4
LOC106842233	1
TBX5	2
LOC106842230	7
RBM19	1
PLBD2	5
TPCN1	4
LOC106842242	4
IQCD	5

RITA1	2
DDX54	2
DTX1	1
PTPN11	2
RPL6	2
HECTD4	5
TRAFD1	3
NAA25	1
ERP29	2
TMEM116	5
LOC106842264	5
MAPKAPK5	6
ALDH2	2
ACAD10	4
BRAP	4
ATXN2	4
SH2B3	1
LOC106842231	9
CCDC63	1
PPP1CC	5
TCTN1	5
PPTC7	1
RAD9B	1
VPS29	2
FAM216A	5
GPN3	5
ARPC3	2
ANAPC7	2
ATP2A2	5
IFT81	5
P2RX7	1
P2RX4	6
ANAPC5	5
RNF34	4
KDM2B	2
LOC106842295	1
ORAI1	5
MORN3	7
TMEM120B	2
PSMD9	2
WDR66	1
BCL7A	2
MLXIP	2
LRRC43	5

DIABLO	3
VPS33A	2
CLIP1	6
ZCCHC8	6
RSRC2	5
KNTC1	6
DENR	3
CCDC62	5
HIP1R	4
VPS37B	2
OGFOD2	6
ARL6IP4	5
PITPNM2	1
MPHOSPH9	5
LOC106842327	5
CDK2AP1	3
SBNO1	3
KMT5A	2
RILPL2	2
RILPL1	9
TMED2	2
DDX55	2
EIF2B1	4
GTF2H3	7
TCTN2	5
ATP6V0A2	4
DNAH10	1
CCDC92	5
ZNF664	1
NCOR2	6
LOC106842350	1
LOC106842364	3
LOC106842351	8
PHF3	3
PTP4A1	7
SC5D	4
LOC106842375	1
TECTA	6
TBCEL	5
ARHGEF12	2
SYNPO2L	1
USP54	1
PPP3CB	6
MSS51	1

ANXA7	2
CFAP70	5
MRPS16	5
DNAJC9	2
FAM149B1	1
ECD	2
P4HA1	6
LOC106842371	4
EXO1	6
WDR64	7
CHML	2
OPN3	7
KMO	7
LOC106842423	7
FH	2
FMN2	2
MIEF1	7
ATF4	2
RPS19BP1	2
ENTHD1	4
GRAP2	1
FAM83F	5
TNRC6B	2
ADSL	2
SGSM3	2
MKL1	4
SLC25A17	4
ST13	2
XPNPEP3	1
DNAJB7	7
RBX1	2
EP300	4
L3MBTL2	5
RANGAP1	7
SLC35F5	2
ACTR3	3
JAM2	2
ATP5J	5
GABPA	6
APP	6
HIVEP1	4
NEDD4L	4
ALPK2	1
LOC106842479	5

MALT1	6
ZNF532	2
SEC11C	2
GRP	5
LMAN1	2
PMAIP1	2
BAMBI	2
WAC	5
LOC106842501	1
LOC106842506	5
MPP7	2
ARMC4	5
MKX	2
RAB18	2
MAP4	6
DHX30	3
LOC106842521	1
TMEM127	5
CIAO1	2
SNRNP200	4
ITPRIPL1	7
NCAPH	5
NEURL3	2
ARID5A	2
KANSL3	4
FER1L5	1
LMAN2L	6
CNNM4	6
CNNM3	2
FAM178B	3
LOC106842542	3
ACTR1B	4
LOC106842544	5
TMEM131	2
VWA3B	8
INPP4A	2
LOC106842553	2
UNC50	5
LOC106842552	9
LOC106842554	2
KCNG3	5
LOC106842589	1
LOC106842593	7
LOC106842594	7

LOC106842607	7
PRSS37	7
SSBP1	2
WEE2	1
KIAA1147	2
AGK	6
LOC106842615	5
TMEM178B	8
MRPS33	3
BRAF	6
NDUFB2	3
DENND2A	5
MKRN1	10
KDM7A	2
PARP12	2
TBXAS1	7
HIPK2	1
CLEC2L	2
LOC106842633	2
LOC106842634	5
UBN2	6
TTC26	1
ZC3HAV1	6
KIAA1549	2
ATP6V0A4	2
SVOPL	1
TRIM24	5
THOC7	4
PSMD6	2
PRICKLE2	1
LOC106842599	8
ADAMTS9	2
LOC106842654	6
MAGI1	2
SLC25A26	5
LRIG1	2
KBTBD8	4
LOC106842661	4
ZBBX	8
LOC106842667	7
USP15	5
LOC106842668	5
LOC106842669	1
PGM2	2

RELL1	2
LOC106842678	2
NWD2	1
ARAP2	2
CNTLN	5
BNC2	2
CCDC33	8
UBL7	5
ARID3B	2
LOC106842697	8
CLK3	5
EDC3	9
ASNS	2
LOC106842704	7
SDHAF3	6
DLX5	1
DLX6	1
LOC106842713	3
SLC25A13	2
DYNC1I1	4
LOC106842722	6
LOC106842723	1
LOC106842724	6
LOC106842728	1
LOC106842727	1
PIGN	1
RNF152	2
SNX30	6
INIP	2
KIAA1958	2
PIK3R4	4
CAPN7	4
SH3BP5	9
METTL6	1
EAF1	7
LOC106842761	8
HACL1	4
BTD	3
ANKRD28	6
TBC1D32	5
HSF2	4
SERINC1	4
LOC106842773	1
POPDC3	2

LIN28B	1
HACE1	1
CDC42SE2	2
LYRM7	5
HINT1	2
CHSY3	1
ISOC1	2
FBN2	4
SLC12A2	2
LOC106842805	1
LOC106842806	1
PRRC1	2
LOC106842813	6
3-Mar	2
LMNB1	6
TEX43	1
PHAX	2
ALDH7A1	2
GRAMD3	2
ZNF608	6
LOC106842824	1
CSNK1G3	6
CEP120	6
PPIC	2
SNX24	2
SNX2	2
SNCAIP	1
ZNF474	7
SRFBP1	4
LOC106842793	5
FTMT	5
FOXF2	2
GMDS	1
WRNIP1	2
SERPINB1	3
XYLB	2
ACVR2B	4
EXOG	1
SCN11A	1
WDR48	2
GORASP1	7
TTC21A	1
CSRNP1	9
LOC106842866	1

SLC25A38	5
RPSA	2
LOC106842856	7
EIF1B	2
ENTPD3	2
LOC106842882	1
NARS2	2
USP35	2
ALG8	4
NDUFC2	5
KCTD14	2
INTS4	3
AAMDC	3
RSF1	2
CLNS1A	2
AQP11	4
PAK1	2
LOC106842881	3
CSTF2	4
ADGRL2	4
CXCL12	2
RASSF4	2
ZNF22	2
LOC106842916	2
RHOU	2
SEC61B	5
ALG2	6
COL15A1	2
GALNT12	2
GABBR2	2
TBC1D2	2
CORO2A	7
TRIM14	1
NANS	2
ANP32B	2
HEMGN	1
TRMO	2
LOC106842937	2
XPA	2
NCBP1	3
TSTD2	5
TDRD7	5
BCKDHB	5
TTK	5

ELOVL4	3
UBE2G2	9
SUMO3	2
PTTG1IP	2
FAM207A	5
ADARB1	6
POFUT2	6
COL18A1	4
SLC19A1	2
PCBP3	10
COL6A1	2
COL6A2	2
SPATC1L	5
LSS	9
MCM3AP	4
LOC106842955	4
YBEY	5
LOC106842990	1
YWHAB	2
TOMM34	2
STK4	6
LOC106842997	5
LOC106843015	9
MATN4	2
SDC4	9
SYS1	9
TP53TG5	7
DBNDD2	4
PIGT	6
WFDC2	2
WFDC3	1
DNTTIP1	5
UBE2C	5
TNNC2	7
SNX21	2
ACOT8	3
ZSWIM1	1
SPATA25	1
NEURL2	7
CTSA	2
PLTP	1
PCIF1	3
ZNF335	4
MMP9	6

NCOA5	1
CD40	6
TMSB4X	2
XKR3	1
GALNT2	6
PGBD5	2
SLAIN1	2
MYCBP2	3
FBXL3	3
CLN5	5
KCTD12	2
LOC106843090	1
LMO7	3
UCHL3	5
COMMD6	5
LOC106843075	1
KLF12	2
LOC106843078	1
KLF5	3
PIBF1	1
DIS3	5
BORA	5
MZT1	4
DACH1	1
LOC106843110	2
TMEM123	2
DCUN1D5	2
LOC106843121	1
DYNC2H1	1
PDGFD	2
DDI1	8
DYNLT3	5
THOC3	2
CPLX2	1
LOC106843125	1
SFXN1	6
DRD1	1
LOC106843143	5
TMEM237	4
ALS2	2
ABHD5	4
ANO10	5
SNRK	6
HIGD1A	5

CCDC13	1
HHATL	2
NKTR	3
SS18L2	5
SEC22C	1
LYZL4	5
CCK	9
LOC106843188	8
MRPL39	5
BDH1	2
APOD	9
PPP1R2	10
ACAP2	3
NEBL	2
CENPC	5
UBA6	4
YTHDC1	6
CDYL	2
RPP40	2
LYRM4	4
FARS2	1
LOC106843241	4
GMFG	7
SAMD4B	4
PAF1	5
MED29	5
ZFP36	2
RPS16	2
SUPT5H	2
TIMM50	2
DLL3	4
LOC106843267	8
LOC106843282	1
DYRK1B	10
FBL	2
FCGBP	2
PSMC4	3
LOC106843288	2
CNTD2	2
AKT2	2
LOC106843290	5
PLD3	2
HIPK4	5
PRX	6

SERTAD1	2
BLVRB	2
SPTBN4	2
SHKBP1	2
ADCK4	1
SNRPA	5
RAB4B	5
EGLN2	5
LOC106843306	1
LOC106843319	7
WFS1	4
DCAF5	5
ACTN1	4
LOC106843327	1
MOCS2	7
FST	2
NDUFS4	5
ARL15	2
SNX18	3
ESM1	5
CDC20B	1
CCNO	5
DHX29	2
SKIV2L2	3
PLPP1	4
LOC106843363	5
SLC38A9	1
DDX4	5
IL31RA	1
IL6ST	2
ANKRD55	5
MAP3K1	2
SETD9	5
MIER3	2
GPBP1	6
PLK2	2
PDE4D	2
LOC106843383	7
TDRD12	1
NUDT19	2
LOC106843386	4
RGS9BP	6
ANKRD27	2
PDCD5	6

LOC106843343	5
DPY19L3	2
ZNF507	2
TSHZ3	2
MIB1	4
SNRPD1	2
ESCO1	6
GREB1L	4
LOC106843402	1
LOC106843403	5
ROCK1	4
LOC106843411	7
RSL24D1	2
RAB27A	2
LOC106843422	3
PIGB	6
CCPG1	8
LOC106843421	5
DYX1C1	5
PYGO1	2
NEDD4	4
RFX7	2
TEX9	5
MNS1	5
ZNF280D	2
LOC106843434	8
LOC106843440	6
ARID2	1
SCAF11	3
SLC38A1	2
SLC38A2	2
PPP1R11	8
ZNRD1	2
ZFP57	1
GABBR1	1
LOC106843519	5
SEC24C	6
CHCHD1	3
ZSWIM8	4
NDST2	5
CAMK2G	2
PLAU	8
VCL	4
AP3M1	2

ADK	6
LOC106843536	1
KAT6B	4
DUPD1	5
DUSP13	5
SAMD8	6
VDAC2	5
COMTD1	2
ZNF503	2
NEIL3	2
SPCS3	5
ASB5	7
SPATA4	5
WDR17	6
CPSF6	2
MDM2	2
SLC35E3	2
NUP107	2
RAP1B	2
MDM1	6
DYRK2	9
LOC106843576	8
YIPF5	3
SH3RF2	7
PLAC8L1	1
SLC1A1	2
SPATA6L	8
CDC37L1	2
AK3	2
RCL1	2
JAK2	3
INSL6	5
LOC106843622	5
RIC1	4
ERMP1	2
KIAA2026	3
RANBP6	1
LOC106843636	7
LOC106843613	1
LOC106843614	5
LOC106843651	5
ATM	4
NPAT	6
ACAT1	2

CUL5	3
LOC106843657	6
RAB39A	6
ALKBH8	4
CWF19L2	5
RAB2A	3
CHD7	6
ASPH	9
STC1	2
SLC25A37	8
PRDM1	2
ATG5	2
AIM1	4
RTN4IP1	2
QRSL1	5
RAB4A	3
CCSAP	8
LOC106843697	7
ACTA1	7
NUP133	1
URB2	4
LOC106843714	2
ZFP2	2
LOC106843711	1
ZNF879	2
RUFY1	5
HNRNPH1	4
CBY3	7
CANX	2
MAML1	6
LTC4S	7
MGAT4B	2
SQSTM1	2
LOC106843725	2
TBC1D9B	2
RNF130	5
LOC106843733	5
MAPK9	2
GFPT2	2
CNOT6	2
LOC106843749	8
GPHN	6
FAM71D	7
KLC3	7

ERCC2	9
CD3EAP	2
ERCC1	2
RTN2	8
VASP	6
EML2	2
SNRPD2	3
QPCTL	1
DMWD	9
RSPH6A	5
SYMPK	1
NOVA2	1
10-Mar	10
MRC2	2
TLK2	5
METTL2A	6
EFCAB3	1
EFCAB13	1
ITGB3	2
LOC106843794	1
IL13RA1	2
DOCK11	4
WDR44	2
NTM	2
HNRNPUL1	3
TGFB1	2
B9D2	2
TMEM91	4
EXOSC5	2
BCKDHA	2
LOC106843824	2
LOC106843826	1
LOC106843828	1
LOC106843833	1
LOC106843832	8
LOC106843838	1
SH3BGRL2	2
LCA5	5
LOC106843847	2
DGKE	2
LOC106843846	6
MMD	2
LOC106843843	1
SFSWAP	6

MMP17	4
ULK1	2
PUS1	2
EP400	4
DDX51	2
NOC4L	2
POLE	8
PXMP2	7
PGAM5	2
ANKLE2	6
GOLGA3	6
CHFR	2
ZNF605	6
ZNF84	10
ZNF140	1
ZNF891	4
ZNF10	4
ZNF268	6
ANHX	2
LOC106843887	7
ZWINT	2
ZCCHC7	2
GRHPR	2
ZBTB5	6
POLR1E	5
FBXO10	7
TOMM5	2
TRMT10B	2
EXOSC3	7
DCAF10	5
SLC25A51	9
SHB	2
IGFBPL1	2
ZNF214	5
NLRP14	1
RBMXL2	5
PPFIBP2	6
LOC106843960	5
API5	2
TTC17	4
HSD17B12	2
ALKBH3	2
ACCS	1
EXT2	2

LOC106843974	1
LOC106843951	5
LOC106843982	1
LOC106843983	5
CRY2	6
MAPK8IP1	8
LOC106843985	5
PEX16	4
PHF21A	1
DGKZ	2
MDK	2
AMBRA1	4
HARBI1	5
ATG13	4
ARHGAP1	6
ZNF408	6
CKAP5	6
LOC106844002	1
LOC106844009	1
ARFGAP2	2
PACSIN3	2
DDB2	6
ACP2	5
MADD	2
SPI1	7
SLC39A13	7
PSMC3	5
CELF1	2
PTPMT1	10
KBTBD4	5
NDUFS3	2
MTCH2	5
AGBL2	1
FNBP4	4
NUP160	1
PSMB6	5
TM4SF5	1
ZMYND15	5
CXCL16	6
MED11	4
PELP1	3
ARRB2	9
RNASEK	2
LOC106844162	2

ACADVL	2
DVL2	6
PHF23	4
GABARAP	5
CTDNEP1	5
ELP5	3
CLDN7	2
SLC2A4	1
YBX2	5
LOC106844185	8
EIF5A	4
GPS2	5
NEURL4	6
LOC106844188	7
ACAP1	8
TMEM95	1
PLSCR3	2
TMEM256	2
SPEM1	7
LOC106844195	7
TMEM102	7
CHRNB1	4
LOC106844202	6
SLC35G6	1
POLR2A	2
LOC106844213	2
SENP3	3
EIF4A1	2
MPDU1	5
FXR2	2
SAT2	2
SHBG	7
ATP1B2	2
TP53	2
WRAP53	2
EFNB3	1
DNAH2	1
KDM6B	1
TMEM88	6
NAA38	7
LOC106844222	1
LOC106844225	8
CHD3	4
TRAPPC1	5

CNTROB	5
ALOX12B	8
PER1	6
VAMP2	4
TMEM107	5
BORCS6	5
LOC106844241	1
AURKB	5
LOC106844243	5
CTC1	4
PFAS	2
ORAOV1	5
CCND1	2
TPCN2	2
IGHMBP2	6
MRPL21	2
CPT1A	4
MTL5	5
GAL	2
PPP6R3	4
LOC106844274	4
LOC106844275	8
KMT5B	6
CHKA	7
TCIRG1	2
NDUFS8	9
ALDH3B1	1
ZNF333	6
ADGRE3	5
NDUFB7	10
TECR	2
GIPC1	2
DNAJB1	5
PKN1	6
DDX39A	5
ADGRE5	2
ADGRL1	2
ASF1B	5
PRKACA	1
SAMD1	2
LOC106844318	5
LOC106844282	10
IL27RA	3
RFX1	1

DCAF15	5
CC2D1A	7
LOC106844322	2
NANOS3	2
LOC106844328	2
MRI1	2
CCDC130	5
LOC106844289	4
IER2	2
STX10	2
LOC106844334	6
NACC1	6
TRMT1	5
GADD45GIP1	2
RAD23A	5
CALR	3
FARSA	3
GCDH	9
KLF1	1
RNASEH2A	10
JUNB	2
VPS37C	6
CD6	3
SLC15A3	5
TMEM132A	2
TMEM109	1
PRPF19	2
CCDC86	2
LOC106844359	10
LOC106844360	5
MS4A13	10
MS4A5	1
MS4A14	1
MS4A7	1
LOC106844394	1
MRPL16	2
LOC106844400	2
PATL1	6
OSBP	2
PBLD	1
MYPN	1
HERC4	6
SIRT1	6
LOC106844401	6

HEY1	4
MRPS28	2
TPD52	2
AAED1	2
LOC106844422	4
ZNF510	5
ZNF782	3
LOC106844428	1
CTSV	2
LOC106844430	8
SLC4A11	5
ITPA	2
DDRGK1	2
UBOX5	2
FASTKD5	6
MRPS26	5
PTPRA	2
VPS16	5
PCED1A	2
LOC106844435	7
TMEM239	7
LOC106844437	7
CPXM1	2
LOC106844458	1
CCNL1	4
SHOX2	2
LOC106844467	2
LOC106844459	2
MLF1	8
ACTL9	1
HNRNPM	3
2-Mar	9
RAB11B	5
ANGPTL4	2
KANK3	7
RPS28	2
NDUFA7	5
CD320	9
CERS4	2
ELAVL1	5
TIMM44	5
CTXN1	8
MAP2K7	2
EVI5L	1

LOC106844506	1
TRAPPC5	2
STXBP2	3
PCP2	7
PET100	5
XAB2	2
CAMSAP3	9
PNPLA6	1
MCOLN1	2
LOC106844521	1
PEX11G	5
ARHGEF18	2
LOC106844528	4
PSMG1	5
ETS2	2
FAM222B	4
TRAF4	6
RPL23A	2
RAB34	4
PROCA1	7
SUPT6H	2
SDF2	2
KIAA0100	6
LOC106844578	6
SPAG5	5
ALDOC	2
PIGS	2
UNC119	2
SLC46A1	2
TMEM199	3
POLDIP2	2
TNFAIP1	4
IFT20	5
TMEM97	2
NLK	6
LYRM9	2
NOS2	6
WSB1	2
NF1	6
RAB11FIP4	5
LOC106844611	2
COPRS	3
UTP6	2
SUZ12	6

CRLF3	2
ATAD5	2
TEFM	4
ADAP2	5
RHOT1	6
LOC106844621	6
ZNF207	4
PSMD11	3
CDK5R1	7
TMEM98	4
SPACA3	5
LOC106844629	1
LOC106844630	9
LOC106844559	7
TMEM132E	1
CCT6B	5
ZNF830	4
LIG3	5
RFFL	1
LOC106844639	7
RAD51D	9
FNDC8	7
NLE1	2
PEX12	1
AP2B1	5
LOC106844656	8
TAF15	4
HEATR9	1
CCL5	2
LOC106844668	2
LOC106844669	2
LOC106844670	2
HEATR6	4
HNF1B	5
DDX52	2
SYNRG	2
DUSP14	2
TADA2A	2
LOC106844683	4
ACACA	6
AATF	6
LHX1	6
MRM1	7
GGNBP2	5

PIGW	2
MYO19	6
ZNHIT3	5
USP32	1
EFCAB1	1
PVRL3	5
PLCXD2	1
PHLDB2	2
ABHD10	2
TAGLN3	2
OCIAD1	3
OCIAD2	2
DCUN1D4	1
SGCB	6
LOC106844566	7
TMEM69	6
IPP	4
NVL	6
CNIH4	2
WDR26	3
LOC106844721	1
CNIH3	9
DNAH14	5
LBR	3
ENAH	2
LOC106844727	8
SRP9	5
PYCR2	2
SDE2	3
H3F3A	3
ACBD3	5
LIN9	6
RUNDC3B	1
RAD51B	6
ZFYVE26	1
RDH12	1
LOC106844775	5
VTI1B	9
ARG2	6
CCDC60	5
PRKAB1	2
CIT	4
CCDC64	3
RAB35	2

LOC106844742	1
GCN1	4
RPLP0	2
PXN	2
SIRT4	1
LOC106844790	2
TRIAP1	2
GATC	2
SRSF9	2
DYNLL1	10
COQ5	2
RNF10	5
POP5	2
CABP1	1
MLEC	3
UNC119B	1
ACADS	2
SPPL3	3
LOC106844805	2
LOC106844808	5
LOC106844743	5
LOC106844744	2
LOC106844815	6
FICD	2
SART3	2
ISCU	7
LOC106844826	4
NCL	2
LOC106844828	1
LOC106844831	7
PTMA	2
COPS7B	4
NPPC	2
DIS3L2	1
ECEL1	8
EIF4E2	2
EFHD1	2
GIGYF2	6
ATG16L1	2
DGKD	9
USP40	6
LOC106844856	7
LOC106844857	1
DNAJB3	8

MROH2A	1
HJURP	3
ARL4C	9
SH3BP4	2
IQCA1	5
LOC106844871	1
BRI3BP	7
DHX37	2
LOC106844875	1
UBC	5
SCARB1	2
LOC106844881	5
PLEKHG3	2
CHURC1	5
FNTB	4
MAX	2
FUT8	5
STK25	5
BOK	2
THAP4	3
ATG4B	4
DTYMK	2
LOC106844911	7
ING5	7
LOC106844900	1
COG2	3
EYA1	7
XKR9	2
LACTB2	2
TRAM1	5
NCOA2	4
SLCO5A1	1
SUPT20H	5
EXOSC8	2
ALG5	5
SMAD9	2
RFXAP	6
SOCS6	2
LOC106844950	1
RRP12	2
PGAM1	2
EXOSC1	4
ZDHHC16	6
MMS19	2

UBTD1	6
ANKRD2	2
LOC106844961	7
MORN4	6
PI4K2A	1
AVPI1	1
ZFYVE27	4
R3HCC1L	1
TRIQK	1
KPNA4	3
TRIM59	5
SMC4	5
IFT80	5
SPATA19	8
IGSF9B	1
JAM3	6
NCAPD3	4
VPS26B	6
ACAD8	2
THYN1	2
LOC106845000	7
TET1	2
CCAR1	6
STOX1	6
DDX50	3
DDX21	2
KIF1BP	2
SRGN	9
VPS26A	5
SUPV3L1	5
HK1	7
TSPAN15	1
COL13A1	2
LOC106845024	2
AIFM2	5
TYSND1	1
LOC106845028	5
SAR1A	3
ABLIM1	7
TMPRSS12	1
ATF1	5
DIP2B	2
LARP4	4
FAM186A	1

LIMA1	5
CERS5	2
LOC106845049	3
SMARCD1	4
RACGAP1	1
AQP5	5
FAIM2	5
BCDIN3D	6
NCKAP5L	2
TMBIM6	10
LOC106845064	8
PRPF40B	6
FAM186B	1
MCRS1	2
KCNH3	6
SPATS2	3
NDFIP2	3
RBM26	3
5-Mar	2
IDE	4
KIF11	3
LOC106845087	1
EXOC6	2
MYOF	6
CEP55	5
RBP4	2
FRA10AC1	3
SLC35G1	2
USP14	2
THOC1	4
COLEC12	2
CETN1	5
TYMS	5
ENOSF1	5
YES1	6
ADCYAP1	1
LOC106845117	1
ALG13	2
AKAP14	5
NDUFA1	2
LOC106845150	1
SEPW1	6
GLTSCR2	2
ZNF541	5

NAPA	3
KPTN	2
DHX34	6
C5AR2	1
BBC3	7
SAE1	4
ZC3H4	1
TMEM160	2
NPAS1	7
ARHGAP35	4
AP2S1	2
STRN4	6
PRKD2	5
DACT3	1
CALM3	5
LOC106845169	4
LOC106845170	1
PPP5C	2
LOC106845176	7
ETV1	2
ARL4A	5
SCIN	4
PANK4	1
PEX10	6
RER1	5
MORN1	5
SKI	2
FAAP20	2
PRKCZ	1
LOC106845215	8
CFAP74	1
LOC106845219	2
GNB1	2
NADK	2
LOC106845222	4
CDK11B	5
MIB2	2
LOC106845191	2
SSU72	9
LOC106845231	2
ACAP3	2
CCNL2	3
AURKAIP1	10
MXRA8	2

CPTP	5
CPSF3L	2
PUSL1	5
UBE2J2	5
FAM132A	2
B3GALT6	2
SDF4	5
TTLL10	7
LOC106845245	2
AGRN	2
ISG15	2
NOC2L	2
SAMD11	8
QSER1	4
PRRG4	6
CCDC73	4
WT1	2
RCN1	2
PAX6	2
ELP4	3
IMMP1L	3
DCDC1	1
PRMT3	6
HTATIP2	2
NAV2	6
LOC106845278	1
E2F8	5
CSRP3	1
ZDHHC13	2
PTPN5	2
TMEM86A	1
SPTY2D1	3
LOC106845288	7
UEVLD	1
MRPS2	2
LOC106845330	5
PPP1R26	2
LOC106845332	2
COL5A1	2
WDR5	3
BRD3	6
LOC106845347	8
VAV2	6
CACFD1	8

ADAMTS13	7
REXO4	7
STKLD1	10
SURF4	2
SURF2	5
SURF1	2
RPL7A	2
MED22	2
SURF6	2
LOC106845362	1
GBGT1	2
RALGDS	9
CEL	1
GTF3C5	1
TSC1	6
LOC106845370	7
AK8	5
GTF3C4	6
CFAP77	1
TTF1	6
SETX	5
MED27	3
RAPGEF1	6
UCK1	2
POMT1	5
PRRC2B	4
FAM78A	7
NUP214	4
AIF1L	2
ABL1	2
EXOSC2	4
FUBP3	2
ASS1	2
NCS1	2
GPR107	6
FNBP1	2
USP20	1
LOC106845404	4
TOR1A	2
PTGES	2
PRRX2	2
ASB6	2
NTMT1	7
LOC106845407	7

IER5L	2
PPP2R4	3
CRAT	5
SH3GLB2	2
NUP188	6
DOLK	1
PHYHD1	1
LRRC8A	2
CCBL1	3
ENDOG	5
TBC1D13	4
ZER1	5
ZDHHC12	5
SET	2
WDR34	5
SPTAN1	2
GLE1	3
ODF2	10
URM1	6
SLC27A4	6
COQ4	1
TRUB2	4
SWI5	5
GOLGA2	5
DNM1	1
CIZ1	5
LOC106845449	2
PTGES2	2
SLC25A25	6
FAM102A	2
DPM2	5
ST6GALNAC4	2
AK1	8
CDK9	2
TOR2A	1
TTC16	1
CFAP157	5
STXBP1	2
RPL12	9
ZNF79	6
SLC2A8	5
GARNL3	7
RALGPS1	1
ZBTB34	4

ZBTB43	4
LOC106845313	2
FAM71E1	7
EMC10	7
ASPDH	8
LOC106845327	5
CLEC11A	2
LOC106845491	2
LOC106845493	5
KLK4	7
DPP7	8
MAN1B1	2
LOC106845496	1
LOC106845525	2
PTGDS	9
C8G	10
FBXW5	5
TRAF2	4
EDF1	10
PHPT1	2
RABL6	2
CCDC183	1
TMEM141	8
GLT6D1	7
SOHLH1	2
CAMSAP1	7
UBAC1	3
NACC2	2
LOC106845548	2
CCDC187	8
GPSM1	4
DNLZ	2
SNAPC4	6
SDCCAG3	7
PMPCA	6
INPP5E	1
SEC16A	5
NOTCH1	2
EGFL7	2
AGPAT2	7
FAM69B	5
LRRC1	10
LOC106845570	6
GCLC	2

ELOVL5	1
FBXO9	2
GSTA4	2
LOC106845591	2
ACTRT1	7
LOC106845594	5
LOC106845595	1
HNMT	4
UBE2J1	7
PM20D2	2
SRSF12	5
PNRC1	2
KIF22	2
MAZ	2
PAGR1	2
MVP	2
LOC106845617	8
CDIPT	6
KCTD13	6
TMEM219	2
TAOK2	4
HIRIP3	5
INO80E	1
DOC2A	1
LOC106845626	1
FAM57B	5
ALDOA	9
PPP4C	3
YPEL3	10
GDPD3	2
MAPK3	7
CORO1A	2
BOLA2B	9
SLX1A	5
LOC106845634	6
SGF29	5
NUPR1	2
CLN3	2
LOC106845643	2
CAND1	2
ATP2B1	2
LOC106845650	1
POC1B	8
DUSP6	2

LOC106845659	1
HMGB4	8
LOC106845662	1
LOC106845660	5
GJB3	1
SMIM12	2
DLGAP3	2
ZMYM6NB	5
ZMYM6	4
CCDC93	3
LOC106845679	1
LOC106845680	1
LOC106845681	1
INSIG2	3
LOC106845675	7
STEAP3	1
LOC106845687	2
ETS1	2
ARHGAP32	2
MINA	2
CRYBG3	1
ARL6	5
LOC106845709	1
LOC106845724	1
RANGRF	5
SLC25A35	6
ODF4	5
KRBA2	4
RPL26	2
NDEL1	1
MYH10	4
CCDC42	5
MFSD6L	5
STX8	5
CFAP52	5
GAS7	4
LOC106845760	1
MYH3	2
LOC106845768	4
LOC106845770	7
DNAH9	1
ZNF18	2
MAP2K4	2
MYOCD	1

ARHGAP44	2
ELAC2	4
LOC106845780	5
LOC106845779	5
PMP22	2
TEKT3	5
CDRT4	7
LOC106845785	6
CDRT1	5
ZNF286A	4
ZNF624	1
LOC106845732	1
STARD7	2
GPAT2	2
FAHD2A	5
LOC106845805	2
LOC106845810	8
LOC106845808	8
LOC106845822	4
CHP2	2
PLK1	5
DCTN5	3
RBBP7	2
TXLNG	2
SYAP1	6
LOC106845814	9
PRICKLE1	2
PPHLN1	2
ZCRB1	5
YAF2	4
GXYLT1	2
DAAM1	4
LOC106845851	1
DACT1	2
KIAA0586	5
TIMM9	3
TOMM20L	1
ARID4A	4
LOC106845860	1
PSMA3	5
ACTR10	3
SLC35B3	1
CEP126	1
LOC106845873	5

YAP1	4
LOC106845877	2
WDR1	2
SLC2A9	2
DRD5	2
TMEM128	5
LYAR	3
ZBTB49	2
LOC106845883	2
STX18	5
PRKX	2
SGMS1	7
MINPP1	4
ATAD1	2
PTEN	5
LOC106845891	1
ZC3H7B	2
TEF	8
TOB2	4
PHF5A	3
ACO2	2
POLR3H	10
CSDC2	1
PMM1	4
ARFGAP3	3
PACSIN2	7
LOC106845939	1
TTLL1	5
BIK	2
MCAT	4
TSPO	2
TTLL12	2
SCUBE1	4
EFCAB6	5
SULT4A1	2
PNPLA3	2
SAMM50	2
KIAA1644	1
LDOC1L	6
PRR5	2
LOC106845961	2
NUP50	5
KIAA0930	7
LOC106845920	1

UPK3A	2
FAM118A	3
SMC1B	3
RIBC2	5
FBLN1	9
LOC106845971	5
ATXN10	2
WNT7B	1
CDPF1	2
PKDREJ	1
TTC38	2
GTSE1	5
TRMU	6
CERK	2
TBC1D22A	6
LOC106845922	1
FAM19A5	2
BRD1	3
ZBED4	2
ALG12	4
CRELD2	5
LOC106845923	8
PIM3	2
TTLL8	7
MLC1	1
MOV10L1	4
PANX2	2
TRABD	2
LOC106845912	2
TUBGCP6	2
MAPK12	5
MAPK11	5
PLXNB2	1
DENND6B	1
PPP6R2	6
SBF1	4
LMF2	4
NCAPH2	7
LOC106846016	6
TYMP	5
ODF3B	7
CPT1B	5
CHKB	2
LOC106846023	6

MAPK8IP2	6
ARSA	5
SHANK3	2
ACR	5
GLI3	2
LOC106846033	7
GREB1	1
E2F6	4
ROCK2	2
PQLC3	2
PDIA6	4
ATP6V1C2	2
NOL10	4
ODC1	2
PERP	9
ARFGEF3	6
NHSL1	1
CCDC28A	9
ECT2L	1
REPS1	6
MNAT1	5
SIX1	2
LOC106846063	2
PPM1A	6
DHRS7	2
PCNXL4	6
LOC106846077	1
LOC106846066	1
CCDC175	5
JKAMP	5
GPR135	1
LOC106846089	1
NOL8	4
IARS	2
ZNF484	6
HELLS	2
TBC1D12	2
NOC3L	6
LOC106846085	1
DLGAP5	3
LGALS3	2
MAPK1IP1L	2
SOCS4	1
WDHD1	6

GCH1	2
SAMD4A	7
CGRRF1	5
GMFB	2
CNIH1	2
CDKN3	5
LOC106846103	1
BMP4	2
PALB2	2
NDUFAB1	3
UBFD1	3
EARS2	2
GGA2	4
COG7	3
USP31	2
OTOA	2
METTL9	2
THUMPD1	6
LOC106846162	6
LOC106846161	5
DCUN1D3	7
LYRM1	5
DNAH3	1
FMR1NB	2
FMR1	2
PTPDC1	5
BARX1	2
PHF2	4
LOC106846175	4
LOC106846176	2
WNK2	2
NINJ1	2
CARD19	5
SUSD3	2
FGD3	1
BICD2	2
LOC106846184	6
IPPK	4
CENPP	5
OGN	9
TGDS	6
GPR180	5
SOX21	2
ABCC4	2

CLDN10	2
DZIP1	5
DNAJC3	5
UGGT2	6
MBNL2	4
RAP2A	6
LOC106846203	8
IPO5	7
FARP1	8
STK24	2
DOCK9	4
HAX1	5
UBAP2L	2
LOC106846241	5
LOC106846248	8
TPM3	9
LOC106846245	1
NUP210L	1
RPS27	2
RAB13	1
JTB	2
SLC39A1	2
DENND4B	6
GATAD2B	9
SLC27A3	4
INTS3	6
NPR1	7
ILF2	2
SNAPIN	6
CHTOP	2
S100A1	9
S100A13	2
S100A4	9
S100A6	2
S100A9	7
LOR	2
PRR9	7
LELP1	7
LOC106846215	8
KLHL4	1
CHRAC1	2
AGO2	4
PTK2	2
SLC45A4	5

PTP4A3	9
LOC106846302	8
MSRB2	2
ARMC3	5
LOC106846326	1
PIP4K2A	2
LOC106846327	8
SPAG6	5
BMI1	2
COMMD3	9
DNAJC1	3
MLLT10	3
DSEL	2
LOC106846330	2
FRAS1	1
MRPL1	6
CNOT6L	2
TNRC18	1
SLC29A4	2
WIPI2	5
RADIL	5
PAPOLB	1
AP5Z1	2
FOXK1	3
LOC106846349	7
RAP1GDS1	1
TSPAN5	7
EIF4E	9
LOC106846354	7
LOC106846355	7
METAP1	4
ADH5	2
SCCPDH	5
KIF28P	1
AHCTF1	6
LOC106846377	8
CDC42BPA	2
ADCK3	2
PSEN2	2
PARP1	2
MTHFD2L	1
AREG	5
BTC	1
RCHY1	5

THAP6	7
CDKL2	3
G3BP2	2
LOC106846391	8
TMX3	4
LOC106846406	4
PPFIA1	1
CRTC1	2
COMP	5
UPF1	5
COPE	7
DDX49	2
HOMER3	2
SUGP2	3
ARMC6	2
SLC25A42	2
GPAT3	7
FAM175A	6
MRPS18C	5
HELQ	1
COQ2	2
PLAC8	9
COPS4	3
LIN54	6
THAP9	2
SEC31A	3
ENOPH1	4
HNRNPDL	2
HNRNPD	3
LOC106846486	2
BRDT	5
EPHX4	2
LOC106846498	8
KIAA1107	2
LOC106846441	3
GLMN	2
RPAP2	1
GFI1	2
EVI5	9
RPL5	3
FAM69A	8
MTF2	6
TMED5	1
CCDC18	1

RNPC3	6
LOC106846500	1
RFX5	5
PI4KB	2
ZNF687	2
PSMD4	5
PIP5K1A	1
VPS72	3
SCNM1	5
LYSMD1	1
GABPB2	6
MLLT11	7
CDC42SE1	4
BNIPL	5
PRUNE	2
FAM63A	1
ANXA9	1
CERS2	1
SETDB1	2
ARNT	4
HORMAD1	6
GOLPH3L	1
ENSA	4
MCL1	5
TARS2	4
RPRD2	4
PRPF3	6
MRPS21	5
CIART	4
ANP32E	2
PLEKHO1	7
VPS45	6
LOC106846563	6
OTUD7B	6
MTMR11	1
SF3B4	3
BOLA1	5
LOC106846575	1
LOC106846577	1
LOC106846576	5
LOC106846581	5
LOC106846590	6
LOC106846586	1
LOC106846589	4

LOC106846592	2
LOC106846587	6
LOC106846593	9
ACP5	2
ELOF1	5
ECSIT	10
ZNF653	5
PRKCSH	5
CCDC151	1
SWSAP1	2
PLPPR2	4
CCDC159	7
TMEM205	7
RAB3D	2
TSPAN16	7
DOCK6	2
SPC24	5
SMARCA4	3
LOC106846631	2
YIPF2	10
CARM1	7
LOC106846617	8
TMED1	5
DNM2	5
QTRT1	2
ILF3	4
SLC44A2	6
AP1M2	5
CDKN2D	5
KRI1	6
ATG4D	5
KEAP1	5
PDE4A	1
CDC37	2
LOC106846673	4
LOC106846667	1
LOC106846664	2
LOC106846648	6
LOC106846651	2
ZNF671	4
LOC106846652	4
LOC106846680	1
ZNF606	6
LOC106846672	5

LOC106846679	1
ZNF329	2
LOC106846660	6
ZNF544	5
ZNF8	1
A1BG	2
RPS5	2
ZNF584	3
ZNF132	6
LOC106846656	1
SLC27A5	7
ZBTB45	1
TRIM28	5
CHMP2A	3
UBE2M	3
MZF1	1
LOC106846661	1
HPSE2	2
CNNM1	1
GOT1	6
LOC106846694	1
SLC25A28	3
LOC106846696	2
CUTC	5
ABCC2	1
DNMBP	4
ERLIN1	5
CHUK	4
CWF19L1	2
BLOC1S2	3
TSNAX	2
EGLN1	2
SPRTN	2
EXOC8	6
GNPAT	10
LOC106846718	2
FAM89A	6
ARV1	3
LOC106846724	2
LOC106846736	1
ALOX5	6
8-Mar	7
LOC106846727	8
ZFAND4	1

FAM21C	2
LOC106846741	4
DCAF12L2	4
SMNDC1	6
MXI1	3
ADD3	3
HIF1AN	6
NDUFB8	5
SCD	2
ERMARD	4
TCTE3	5
PHF10	5
LOC106846762	4
WDR27	1
LOC106846769	1
DIMT1	6
KIF2A	3
SMIM15	2
NDUFAF2	5
PTPRJ	2
SMYD2	2
PTPN14	4
CENPF	5
KCNK2	2
PLA2G4A	6
NT5DC3	2
HSP90B1	5
LOC106846787	2
TDG	2
HCFC2	5
NFYB	2
TXNRD1	2
EID3	5
CHST11	5
SLC41A2	1
LOC106846823	2
KIAA1033	4
APPL2	1
LOC106846802	9
NUAK1	2
CKAP4	5
LOC106846806	8
TCP11L2	1
POLR3B	2

RFX4	5
ALX1	2
LRRIQ1	5
SLC6A15	5
LOC106846815	1
LOC106846834	1
LOC106846833	8
TASP1	6
LOC106846841	7
SMYD3	2
KIF26B	2
LOC106846851	6
LOC106846846	1
LOC106846853	8
TPM4	2
RAB8A	5
CIB3	9
FAM32A	3
AP1M1	5
KLF2	2
CALR3	5
LOC106846861	5
CHERP	2
MED26	5
SMIM7	2
TMEM38A	4
SIN3B	4
HAUS8	2
USE1	10
OCEL1	2
NR2F6	2
BABAM1	3
ABHD8	5
MRPL34	7
DDA1	7
GTPBP3	6
BST2	2
MVB12A	2
SLC27A1	2
PGLS	2
LOC106846910	4
LOC106846907	7
ATRX	2
LOC106846922	2

PGK1	2
TAF9B	2
LOC106846930	1
LOC106846934	5
FGFR2	4
WDR11	4
PKP2	6
YARS2	6
DNM1L	2
FGD4	1
BICD1	4
KIAA1551	2
AMN1	5
MGST1	2
HPS1	4
TMEM8B	1
FAM221B	8
HINT2	9
SPAG8	5
NPR2	2
RGP1	5
MSMP	2
GBA2	8
CREB3	5
TLN1	4
TPM2	7
CA9	10
ARHGEF39	1
CCDC107	2
TESK1	1
RUSC2	5
LOC106846989	6
UNC13B	9
FAM214B	7
STOML2	2
PIGO	1
FANCG	5
VCP	2
LOC106846999	5
DNAJB5	1
PHF24	6
LOC106847009	1
ASF1A	2
CEP85L	4

PLN	1
SLC35F1	1
NUS1	2
LOC106847087	1
GOPC	5
VGLL2	1
FAM162B	2
KPNA5	1
ZUFSP	4
RWDD1	5
DSE	1
LOC106847034	1
TSPYL1	6
TSPYL4	1
LOC106847039	1
NT5DC1	2
HDAC2	2
LAMA4	6
FAM229B	10
TUBE1	2
FYN	2
LOC106847049	1
REV3L	6
KIAA1919	7
RPF2	4
GTF3C6	5
AMD1	2
SLC22A16	5
DDO	1
SMARCA1	2
OCRL	2
ZDHHC9	6
AIFM1	2
SLC2A11	1
MIF	2
LOC106847146	8
LOC106847117	2
LOC106847118	5
CABIN1	1
SNRPD3	2
GUCD1	2
UPB1	1
ADORA2A	1
SPECC1L	7

BCR	2
RSPH14	1
GNAZ	2
LOC106847137	1
LOC106847147	1
DROSHA	2
LOC106847154	4
PDZD2	4
LOC106847156	1
GOLPH3	9
MPV17L	4
PDXDC1	7
NTAN1	5
RRN3	5
LOC106847164	5
BFAR	6
PARN	1
LOC106847170	1
MKL2	7
ERCC4	2
PIAS1	2
CALML4	1
CLN6	3
LOC106847179	1
LOC106847182	7
LOC106847186	1
EPB41L3	4
GMPR	2
ATXN1	2
RBM24	2
FAM8A1	1
NUP153	7
UBXN2A	2
ATAD2B	4
KLHL29	4
TDRD15	6
RGS17	2
MTRF1L	2
LOC106847239	6
FBXO5	2
VIP	1
LOC106847220	7
YPEL5	9
LBH	2

LCLAT1	6
UBE2D3	2
LOC106847251	1
CISD2	2
SLC9B1	5
LOC106847257	2
BDH2	2
CENPE	5
TXLNB	1
HECA	2
ABRACL	2
QRFPR	2
PRDM5	2
LOC106847275	7
MAD2L1	2
LOC106847269	1
OSTF1	2
NMRK1	2
CARNMT1	3
RASEF	2
LOC106847301	5
PEX5	6
C1R	9
C1S	4
LPCAT3	5
EMG1	3
PHB2	2
LOC106847315	6
ENO2	2
LRRC23	1
SPSB2	1
TPI1	7
USP5	3
CDCA3	5
LOC106847330	6
MLF2	6
COPS7A	3
PIANP	2
LOC106847295	1
ZNF384	4
ING4	5
ACRBP	5
LPAR5	1
CHD4	2

NOP2	4
IFFO1	6
GAPDH	2
NCAPD2	6
MRPL51	5
VAMP1	2
TAPBPL	6
MMP16	2
LOC106847349	1
LOC106847350	1
LOC106847351	1
CELF2	9
USP6NL	5
LOC106847359	5
SRI	4
ADAM22	4
DBF4	5
SPOPL	2
LOC106847388	6
ZNF772	1
LOC106847392	4
LOC106847389	2
ZNF304	1
LOC106847413	1
LOC106847398	4
LOC106847400	6
AURKC	3
PEG3	4
LOC106847380	7
ZNF835	5
LOC106847406	5
LOC106847405	6
ZNF582	2
LYRM2	1
LOC106847417	1
MDN1	6
CASP8AP2	2
BACH2	4
MAP3K7	4
SHCBP1L	5
DHX9	6
NPL	2
RGS8	9
RGS16	9

RGSL1	1
CSTF2T	6
LOC106847487	2
FAM173B	5
CCT5	5
CMBL	2
6-Mar	2
ROPN1L	5
ANKRD33B	1
DAP	2
CTNND2	2
RRM2	1
KLF11	2
GRHL1	4
TAF1B	5
YWHAQ	3
ADAM17	1
IAH1	10
CPSF3	3
ITGB1BP1	3
ASAP2	8
MBOAT2	2
KIDINS220	4
ID2	2
LOC106847496	1
RNF144A	2
CMPK2	2
SOX11	2
DCDC2C	7
ALLC	5
RPS7	2
RNASEH1	2
ADI1	2
TRAPPC12	5
TSSC1	3
NAALADL2	1
SPATA16	5
ECT2	5
FNDC3B	1
PLD1	1
TNIK	4
EIF5A2	9
RPL22L1	2
LOC106847545	1

OSBPL11	6
SNX4	4
LOC106847515	1
ZNF148	4
SLC12A8	1
MUC13	7
LOC106847541	2
UMPS	2
LOC106847516	1
LOC106847551	5
CCDC14	6
VWA8	2
DGKH	6
AKAP11	6
INCENP	4
FTH1	2
BEST1	5
RAB3IL1	7
FADS2	6
FADS1	2
FEN1	2
TMEM258	5
PPP1R32	5
SDHAF2	3
CPSF7	4
TMEM216	6
TMEM138	5
LOC106847573	6
TKFC	2
DDB1	2
SIPA1L1	2
LOC106847601	4
ENO1	2
CA6	7
ZNF518B	2
HS3ST1	6
TP53RK	5
EYA2	7
ZMYND8	4
NCOA3	6
SULF2	7
LOC106847650	4
PRKAB2	2
CHD1L	2

BCL9	4
ACP6	4
GPR89A	4
RNF115	4
LOC106847642	7
POLR3C	5
PIAS3	6
NUDT17	5
PEX11B	5
RBM8A	2
LIX1L	2
TXNIP	2
RASSF2	2
PRNP	2
ADRA1D	1
SMOX	2
LOC106847687	7
LOC106847691	1
RNF24	8
PANK2	5
AP5S1	6
CDC25B	2
SPEF1	1
LOC106847683	5
GFRA4	1
ATRN	4
PEBP4	5
EGR3	8
BIN3	5
CCAR2	4
SORBS3	9
PPP3CC	5
SLC39A14	2
PIWIL2	2
POLR3D	2
REEP4	3
NUDT18	7
XPO7	2
DOK2	5
EXD2	8
ERH	2
SLC39A9	6
SUSD6	7
SRSF5	4

SLC8A3	5
LOC106847737	3
SYNJ2BP	5
CHST15	8
CPXM2	2
BUB3	3
ACADSB	2
CFAP57	1
EBNA1BP2	6
FAM183A	5
LOC106847777	5
ZNF691	5
SVBP	2
LOC106847828	7
LOC106847795	2
P3H1	2
CLDN19	2
YBX1	5
PPIH	2
CCDC30	1
PPCS	7
ZMYND12	5
RIMKLA	7
FOXJ3	4
GUCA2A	7
LOC106847831	7
SCMH1	6
SLFNL1	7
CTPS1	2
CITED4	2
NFYC	3
DIRAS3	2
LOC106847775	1
WLS	4
DEPDC1	2
LRRC7	1
LRRC40	4
SRSF11	3
LOC106847784	1
ANKRD13C	1
CTH	2
PTGER3	6
ZRANB2	5
RGS22	8

FBXO43	5
POLR2K	2
SPAG1	5
RNF19A	7
ANKRD46	9
SNX31	1
PABPC1	5
YWHAZ	9
ZNF706	10
ERICH1	6
TDRP	4
FBXO25	6
ZNF596	2
LOC106847859	2
LONRF1	7
PRKD1	2
LOC106847863	5
MROH2B	8
PLCXD3	1
OXCT1	4
LOC106847871	4
FBXO4	5
NQO2	6
RIPK1	2
BPHL	2
LOC106847879	2
TUBB2B	2
PSMG4	2
FAM50B	5
EIF5B	3
TXNDC9	5
TIE1	2
LOC106847918	1
LOC106847923	5
CDC20	5
ELOVL1	2
MED8	2
SZT2	6
PTPRF	4
KDM4A	2
IPO13	6
DPH2	5
ATP6V0B	2
B4GALT2	2

CCDC24	1
KLF17	1
DMAP1	3
LOC106847919	6
LOC106847977	7
LYPD4	5
DMRTC2	5
RPS19	2
RABAC1	9
ATP1A3	1
ZNF574	5
POU2F2	2
DEDD2	2
ZNF526	8
GSK3A	6
ERF	7
CIC	4
PAFAH1B3	5
PRR19	5
TMEM145	5
LOC106847944	6
LIPE	7
CHST7	2
RP2	1
RGN	2
NDUFB11	2
RBM10	2
UBA1	4
CDK16	7
USP11	2
LOC106848000	3
ARAF	2
TIMP1	2
ELK1	8
UXT	4
DMD	2
LOC106848001	2
LOC106848012	1
NRIP1	4
LOC106848024	6
HSPA13	9
RBM11	6
LOC106848027	5
ROBO2	4

LOC106848015	1
LOC106848008	1
LOC106848032	2
LOC106848040	6
ZBTB18	2
LOC106848035	7
ADSS	6
DEPTOR	1
DSCC1	2
TAF2	4
NOV	2
MAL2	2
LANCL1	5
KANSL1L	3
RPE	2
MAP2	2
LOC106848073	2
SSH1	6
SVOP	1
USP30	1
ALKBH2	1
UNG	2
ACACB	1
KCTD10	4
UBE3B	8
MMAB	2
MVK	5
GLTP	2
TCHP	4
GIT2	4
ANKRD13A	5
LOC106848065	2
TCF7L2	2
NRAP	2
CASP7	2
DCLRE1A	2
NHLRC2	2
CCDC186	1
LOC106848095	1
TDRD1	4
TMEM65	2
FAM91A1	4
ANXA13	7
WDYHV1	2

LOC106848109	5
LOC106848112	9
PLEKHA2	6
TM2D2	9
ZFX	2
EIF2S3	2
LOC106848142	5
APOO	2
SAT1	9
ACOT9	2
PRDX4	4
LOC106848181	5
ZNF280B	5
PRAME	4
LOC106848161	9
LOC106848172	5
LOC106848171	2
LOC106848164	5
LOC106848167	1
LOC106848182	1
CUL4A	2
LAMP1	3
GRTP1	1
DCUN1D2	6
TMCO3	2
TFDP1	2
GAS6	2
LOC106848205	7
RASA3	6
LOC106848203	8
CDC16	4
UPF3A	6
CHAMP1	2
SHCBP1	5
VPS35	2
ORC6	2
MYLK3	1
LOC106848232	3
GPT2	4
DNAJA2	2
NETO2	2
ITFG1	2
TMEM126B	2
TMEM126A	2

CREBZF	6
CCDC89	5
SYTL2	1
LOC106848227	1
CCDC83	5
PICALM	7
LOC106848223	1
EED	2
LOC106848256	2
CCDC81	5
ME3	9
PRSS23	9
LOC106848254	8
CTSC	2
LOC106848239	2
TOP3B	6
MAPK1	6
YPEL1	5
PPIL2	9
LOC106848294	7
SDF2L1	5
CCDC116	1
YDJC	4
UBE2L3	5
LOC106848279	5
HIC2	2
TMEM191C	7
PI4KA	4
SERPIND1	8
SNAP29	5
CRKL	6
THAP7	2
LOC106848269	7
LOC106848297	1
USP25	7
PKDCC	2
EML4	2
DLG1	6
NCBP2	6
SENP5	2
PAK2	2
PIGX	4
CEP19	5
FBXO45	6

WDR53	5
SMCO1	6
RNF168	6
UBXN7	6
TCTEX1D2	5
PCYT1A	8
CPNE8	2
KIF21A	2
LOC106848335	1
PRKDC	2
CEBPD	2
SPIDR	4
PSMB4	3
POGZ	2
LOC106848382	8
TUFT1	2
SNX27	5
CELF3	1
RIIAD1	5
MRPL9	5
OAZ3	7
TDRKH	6
THEM4	5
LOC106848377	2
S100A10	2
S100A11	2
TCHH	2
PTCHD3	5
LOC106848386	1
YME1L1	3
MASTL	4
ACBD5	2
ABI1	2
APBB1IP	2
MYO3A	4
TEX101	1
LYPD3	2
PHLDB3	2
ZNF575	1
XRCC1	2
IRGQ	4
ZNF576	1
ZNF428	2
IRGC	7

SMG9	5
ZNF283	6
ZNF404	2
CLPTM1	9
RELB	2
CLASRP	4
ZNF296	2
GEMIN7	6
TRAPPC6A	2
EXOC3L2	2
SH3RF1	2
NEK1	3
CLCN3	1
LOC106848446	3
MFAP3L	8
LOC106848467	9
PDSS2	6
SEC63	2
EDEM1	6
ARL8B	5
ITPR1	1
SUMF1	2
LOC106848471	2
CRBN	2
TRNT1	5
COG6	2
LOC106848480	1
FOXO1	2
MRPS31	2
NDUFA6	5
NAGA	5
WBP2NL	7
LOC106848515	1
CENPM	4
SREBF2	4
CCDC134	5
LOC106848520	7
SNU13	5
XRCC6	4
DESI1	10
LOC106848504	2
POLDIP3	2
RRP7A	4
LOC106848517	2

TCF20	6
LOC106848498	7
LOC106848485	7
LOC106848497	7
LOC106848501	9
RIMS1	4
OGFRL1	2
ARHGAP19	1
LCOR	2
SNAPC1	4
HIF1A	2
PRKCH	1
TMEM30B	5
SLC38A6	4
TRMT5	5
MND1	6
TRIM2	2
ARFIP1	1
TIGD4	5
FBXW7	6
LOC106848595	1
LOC106848596	1
LOC106848563	7
SDR39U1	5
NFATC4	1
CIDEB	1
NOP9	6
DHRS1	6
RABGGTA	2
TINF2	5
GMPR2	6
NEDD8	5
MDP1	2
CHMP4A	2
TSSK4	5
TM9SF1	5
IPO4	5
REC8	1
IRF9	2
RNF31	2
PSME2	2
EMC9	10
PSME1	2
DCAF11	7

PCK2	2
LOC106848600	2
CDC73	4
PRG4	1
TPR	3
LOC106848629	6
PDC	5
COL12A1	2
LOC106848636	3
TMEM30A	1
FILIP1	6
CADPS	8
LOC106848682	1
PTPRG	2
FHIT	2
LOC106848675	1
FAM3D	1
FAM107A	2
KCTD6	2
PDHB	3
PXK	2
RPP14	4
ABHD6	1
DNASE1L3	5
LOC106848684	5
FLNB	9
SLMAP	3
DENND6A	2
ARF4	3
PDE12	5
LOC106848644	2
DNAH12	5
APPL1	2
IL17RD	7
ARHGEF3	1
FAM208A	4
CCDC66	5
WNT5A	2
DENND5B	4
FAM60A	2
CAPRIN2	2
IPO8	4
SFMBT1	2
LOC106821737	2

NEK4	5
SPCS1	5
GLT8D1	5
GNL3	2
PBRM1	6
NT5DC2	2
STAB1	5
NISCH	2
PHF7	10
BAP1	5
DNAH1	1
GLYCTK	9
WDR82	2
PPM1M	9
TWF2	2
ALAS1	3
POC1A	3
RPL29	9
ACY1	9
ABHD14A	1
LOC106821764	1
LOC106821777	2
SHISA5	2
ATRIP	5
CCDC51	2
SPINK8	7
NME6	2
ZNF589	1
CDC25A	1
LOC106821775	7
RRAGB	2
KIAA0922	6
LOC106821796	1
LOC106821797	5
ZNF496	2
RNF187	3
LOC106821823	1
LOC106821822	1
LOC106821831	6
TRIM17	1
TRIM11	2
OBSCN	2
IBA57	1
GUK1	2

MRPL55	9
LOC106821808	2
ARF1	3
PRSS38	4
JMJD4	2
LOC106821882	4
LOC106821887	2
LOC106821876	2
LOC106821883	7
LOC106821915	1
LOC106821903	6
AKAP17A	6
CD99	9
SIMC1	5
KIAA1191	2
ARL10	2
NOP16	3
HIGD2A	5
CLTB	2
FAF2	3
RNF44	7
CDHR2	9
TSPAN17	2
HK3	1
UIMC1	5
SPATA22	5
LOC106821968	7
CLUH	2
PAFAH1B1	10
METTL16	6
SGSM2	7
TSR1	7
SRR	1
SMG6	1
HIC1	1
RPA1	2
SMYD4	4
LOC106822043	6
PRPF8	2
RILP	10
SLC43A2	2
PITPNA	5
INPP5K	8
MYO1C	2

CRK	6
YWHAE	3
RPH3AL	2
LOC106822051	5
FAM101B	2
VPS53	2
FAM57A	7
GEMIN4	1
LOC106821995	7
GLOD4	4
RNMTL1	5
NXN	2
TIMM22	2
ABR	8
GOSR1	5
CPD	1
BLMH	3
NSRP1	4
EFCAB5	5
SSH2	5
ANKRD13B	1
GIT1	1
ABHD15	1
TAOK1	6
DAPK1	1
ZCCHC6	6
ISCA1	7
LOC106822049	7
GOLM1	2
NAA35	2
AGTPBP1	5
NTRK2	1
LOC106822023	5
LOC106822024	5
LOC106822022	1
IMPA1	2
ZFAND1	2
SNX16	2
DCP1A	6
CACNA1D	1
CHDH	1
ACTR8	2
LOC106822067	9
SLC3A2	4

CHRM1	1
SLC22A6	8
HRASLS5	10
SMPD4	5
LOC106822092	5
MED15	7
KLHL22	2
LOC106822095	6
MRPL40	2
LOC106822102	2
UFD1L	3
CDC45	1
CLDN5	2
LOC106822103	6
LOC106822104	2
SEPT5	9
TBX1	3
GNB1L	1
TXNRD2	5
ARVCF	2
AKAP4	7
CCNB3	6
OSBPL10	7
GPD1L	7
CMTM8	2
CMTM7	4
CMTM6	2
DYNC1LI1	5
LOC106822136	4
LOC106822128	5
SMC1A	4
RIBC1	5
HSD17B10	2
HUWE1	2
ZFP91	5
FADD	2
LOC106822169	2
FAS	2
ACTA2	2
STAMBPL1	4
LIPJ	1
LOC106822259	1
GNAO1	1
AMFR	7

NUDT21	5
OGFOD1	3
BBS2	3
MT3	2
LOC106822242	2
LOC106822243	2
LOC106822245	2
LOC106822247	2
LOC106822244	9
NUP93	4
HERPUD1	7
CPNE2	1
FAM192A	7
RSPRY1	1
ARL2BP	5
PLLP	2
CIAPIN1	3
COQ9	2
POLR2C	2
DOK4	9
CCDC102A	2
ADGRG1	1
DRC7	5
KATNB1	5
KIFC3	1
TEPP	7
USB1	2
CFAP20	3
CSNK2A2	4
CCDC113	5
PRSS54	5
SETD6	6
CNOT1	3
GOT2	2
FNIP1	4
RAPGEF6	6
SLX4IP	4
JAG1	4
LOC106822269	1
BTBD3	2
KLF3	2
FAM114A1	9
WDR19	1
LOC106822280	6

UPRT	4
ABCB7	2
RLIM	2
LOC106822351	1
LOC106822347	5
SIGLECL1	5
LOC106822354	5
IGLON5	4
ETFB	2
LOC106822321	2
LOC106822332	1
LOC106822325	1
KIAA0319	1
ALDH5A1	7
MRS2	2
DCDC2	5
ZNF711	2
SATL1	9
ZNF658	2
PDCD6IP	4
CLASP2	8
UBP1	2
LOC106822377	1
LOC106822391	4
LOC106822388	1
LMO4	2
HS2ST1	6
LOC106822383	4
SH3GLB1	5
LOC106822381	6
LOC106822392	1
LOC106822393	2
LOC106822399	5
MIS18A	4
URB1	2
LOC106822406	5
SYNJ1	5
PAXBP1	4
LOC106822407	5
ABCD2	5
LOC106822500	4
SLC2A13	4
LRRK2	4
CNTN1	1

RFX3	1
GLIS3	6
LOC106822485	4
LOC106822494	2
ABI2	8
LOC106822478	2
TERF2	9
NIP7	6
COG8	1
PDF	5
VPS4A	5
SNTB2	2
UTP4	2
CHTF8	2
TANGO6	6
LOC106822457	5
CDH3	2
ZFP90	1
PRMT7	6
SLC7A6OS	6
SLC7A6	6
PLA2G15	4
NFATC3	2
DUS2	1
LOC106822518	9
LOC106822432	4
PSMB10	2
PSKH1	1
NRN1L	9
EDC4	3
NUTF2	2
THAP11	2
CENPT	5
TSNAXIP1	5
RANBP10	6
GFOD2	5
LOC106822512	5
ENKD1	5
PARD6A	8
ACD	5
CTCF	2
LOC106822430	1
FAM65A	4
LOC106822431	4

ATP6V0D1	2
ZDHHC1	1
LRRC36	5
KCTD19	5
FHOD1	9
LOC106822493	7
TMEM208	6
E2F4	4
EXOC3L1	9
KIAA0895L	7
B3GNT9	2
LOC106822444	2
LOC106822424	1
LOC106822526	7
LOC106822527	1
LOC106822525	5
EFTUD1	5
LOC106822529	1
SLC43A1	2
TIMM10	2
SERPING1	2
YPEL4	1
CLP1	1
LOC106822563	2
ZDHHC5	5
MED19	3
TMX2	5
LOC106822561	2
CTNND1	2
CTSB	2
FDFT1	5
NEIL2	2
MAST2	2
PIK3R3	6
LOC106822596	7
POMGNT1	1
LURAP1	1
LOC106822597	1
RAD54L	5
LRRC41	5
LOC106822600	2
NSUN4	7
FAAH	6
ATPAF1	3

TEX38	5
EFCAB14	3
MED14	2
LOC106822612	2
MPC1L	5
ATP6AP2	2
BCOR	4
CHM	2
ZNF331	4
LOC106822660	2
LOC106822654	1
LOC106822634	5
LOC106822652	4
LOC106822653	6
LOC106822636	3
LOC106822659	5
LOC106822668	4
PPP2R1A	2
NOX4	5
LOC106822680	4
ESR1	8
LOC106822684	8
ARMT1	5
RMND1	2
ZBTB2	2
LOC106822708	7
FKBP15	2
SLC31A1	2
CDC26	5
PRPF4	3
WDR31	8
BSPRY	6
HDHD3	4
ALAD	10
POLE3	9
LOC106822720	5
RGS3	4
LOC106822725	2
SHANK2	1
LOC106822729	7
DHCR7	4
NADSYN1	2
MYO6	6
SENP6	2

ERI3	1
RNF220	3
TMEM53	7
LOC106822739	5
LOC106822744	5
KIF2C	7
RPS8	2
PTCH2	2
EIF2B3	8
HECTD3	1
UROD	8
ZSWIM5	2
CACNA1B	6
EHMT1	6
ARRDC1	2
ZMYND19	2
DPH7	3
MRPL41	10
PNPLA7	6
LOC106822773	1
NSMF	4
EXD3	7
NELFB	2
LOC106822769	8
FAM166A	7
TUBB4B	5
RNF208	4
NDOR1	4
TMEM203	5
TPRN	2
SSNA1	5
ANAPC2	6
TMEM210	1
LRRC26	7
LOC106822817	7
LOC106822857	4
LOC106822903	2
LOC106822905	3
TGIF1	2
ZBTB14	2
LOC106822957	5
LOC106822861	1
LAMA3	6
TTC39C	2

CABYR	10
OSBPL1A	5
IMPACT	2
ZNF521	6
SS18	2
PSMA8	5
TAF4B	2
MRPS24	3
LOC106822888	2
UBE2D4	5
DBNL	3
PGAM2	10
POLM	1
POLD2	7
YKT6	4
CAMK2B	2
NUDCD3	3
DDX56	1
TMED4	5
OGDH	6
ZMIZ2	8
PPIA	3
LOC106822946	5
PURB	3
MYO1G	2
LOC106822929	2
CCM2	2
NACAD	2
TBRG4	2
RAMP3	9
ADCY1	6
LOC106822880	1
TNS3	8
HUS1	3
SUN3	8
LOC106822955	5
UPP1	2
ZPBP	5
LOC106822969	1
FIGNL1	4
GRB10	2
COBL	8
HNRNPA0	2
KLHL3	1

SMAD5	2
FBXL21	2
CXCL14	2
LOC106822981	7
LOC106822985	9
CATSPER3	1
PCBD2	2
TXNDC15	3
LOC106823008	3
DDX46	4
CAMLG	5
SEC24A	4
RFT1	6
PRKCD	7
TKT	2
ICE1	2
MED10	2
NSUN2	2
SRD5A1	2
PAPD7	2
ADCY2	2
LOC106823002	5
FASTKD3	2
MTRR	2
LOC106823016	2
SEMA5A	4
NGFRAP1	2
BEX4	2
LOC106823038	2
LOC106823039	2
WBP5	2
LOC106823036	2
TCEAL4	2
TCEAL3	2
NEK11	1
ASTE1	1
ATP2C1	4
RMI1	5
HNRNPK	3
LOC106823066	2
KIF27	1
LOC106823063	1
GKAP1	5
UBQLN1	2

CYLC1	7
LOC106823067	6
ZNF177	2
DIAPH2	1
RPA4	1
GYPC	2
LOC106823105	2
LOC106823104	7
ARMCX2	2
ARMCX3	2
ARMCX1	2
HNRNPH2	3
RPL36A	2
TIMM8A	2
TAF7L	2
LOC106823103	2
TRIT1	6
CAP1	9
PPT1	2
RLF	3
TMCO2	8
ZMPSTE24	5
COL9A2	2
SMAP2	2
ZFP69	2
EXO5	6
LOC106823129	1
ZNF684	1
LOC106823132	1
RIMS3	8
SLC17A5	6
CD109	2
LOC106823156	5
ZNF165	2
ZKSCAN8	7
LOC106823154	6
ZSCAN9	2
ZKSCAN4	6
NKAPL	5
LOC106823149	1
LOC106823143	4
LOC106823158	6
ZSCAN23	1
LOC106823175	4

PCCB	2
MSL2	4
PPP2R3A	2
LOC106823211	1
EPHB1	7
CEP63	5
ANAPC13	2
RYK	2
SLCO2A1	1
RAB6B	2
SRPRB	2
LOC106823197	2
TOPBP1	1
SLC23A2	2
TMEM230	5
PCNA	5
CDS2	2
LOC106823172	1
LOC106823169	8
LOC106823209	8
GPCPD1	6
LOC106823194	2
MCM8	1
TRMT6	2
CRLS1	5
FERMT1	2
BMP2	2
TMX4	7
PLCB1	2
PLCB4	4
PAK7	7
SAMD12	4
NFE2L3	2
HNRNPA2B1	2
CBX3	2
SNX10	2
SKAP2	6
LOC106823223	5
LOC106823238	1
EIF2S2	5
RALY	3
CHMP4B	5
PXMP4	4
NECAB3	1

E2F1	2
ACTL10	5
CBFA2T2	4
LOC106823229	7
SNTA1	7
CDK5RAP1	3
LOC106823241	1
PRSS42	1
PTH1R	2
CCDC12	4
NBEAL2	4
LOC106823257	5
SETD2	6
KIF9	10
KLHL18	6
PTPN23	6
SCAP	6
ELP6	6
CSPG5	1
SMARCC1	2
CHMP2B	2
CNKSR3	2
LOC106823283	6
PJA1	2
EFNB1	2
LOC106823296	1
TAF4	5
LSM14B	9
STX7	2
CTGF	2
LOC106823309	1
KCNH2	6
NOS3	1
ATG9B	1
ABCB8	2
CDK5	2
SLC4A2	2
FASTK	2
TMUB1	5
AGAP3	2
ASB10	1
IQCA1L	1
LOC106823315	7
ABCF2	5

CHPF2	5
SMARCD3	2
NUB1	5
WDR86	2
CRYGN	1
PRKAG2	2
GALNTL5	7
GALNT11	6
KMT2C	6
CCT8L2	7
XRCC2	5
ACTR3B	6
DPP6	8
PAXIP1	6
HTR5A	1
INSIG1	2
EN2	2
CNPY1	2
RBM33	6
RNF32	5
LMBR1	7
NOM1	2
UBE3C	4
DNAJB6	5
PTPRN2	4
ATP5A1	2
HAUS1	2
LOC106823359	6
RNF165	7
ST8SIA5	2
PIAS2	5
LOC106823394	7
DENND1B	7
ZBTB41	4
ASPM	3
IGSF11	7
LOC106823403	7
B4GALT4	2
TMEM39A	6
IMPA2	1
TUBB6	2
AFG3L2	2
PRELID3A	2
SPIRE1	5

LOC106823412	5
LOC106823413	5
GNAL	7
CHMP1B	4
MPPE1	5
CDH18	1
CFAP43	1
SFR1	3
SLK	4
OBFC1	5
SH3PXD2A	2
NEURL1	7
PDCD11	4
USMG5	3
LOC106823417	1
HYDIN	1
VAC14	2
SF3B3	2
COG4	5
ST3GAL2	1
LOC106823438	5
AARS	2
EXOSC6	5
LOC106823481	6
LOC106823480	1
LOC106823492	1
LOC106823501	6
BZW1	9
CLK1	4
LOC106823491	6
PPIL3	6
NIF3L1	2
ORC2	3
FAM126B	6
NDUFB3	3
CASP10	1
ALS2CR12	5
TRAK2	6
STRADB	6
ALS2CR11	5
NIFK	2
TSN	2
MAPRE2	1
ZNF397	7

ZNF24	6
ZNF396	6
INO80C	2
GALNT1	2
LOC106823528	1
LOC106823556	5
RPRD1A	6
SLC39A6	2
ELP2	2
FHOD3	2
TPGS2	5
KIAA1328	1
HMGN3	3
PHIP	4
IRAK1BP1	5
CCNY	9
LHX9	5
NEK7	2
PTPRC	2
LOC106823562	1
NR5A2	9
ZNF281	6
KIF14	5
DDX59	6
LOC106823567	2
CAMSAP2	2
KIF21B	7
CACNA1S	8
TMEM9	2
IGFN1	1
MOSPD1	2
LOC106823574	8
FAM122B	6
HPRT1	2
PHF6	2
CCDC160	5
LOC106823581	5
LOC106823583	1
LOC106823588	1
TNKS1BP1	4
SSRP1	2
CHST10	2
TFCP2L1	2
CLASP1	2

LOC106823637	1
LOC106823638	1
TMEM177	5
LOC106823642	1
CFAP221	5
LOC106823682	1
ZNF317	2
LOC106823687	2
LOC106823688	1
UHMK1	2
UAP1	5
DDR2	6
HSD17B7	6
GPR156	2
LRRC58	2
FSTL1	2
NDUFB4	5
RABL3	1
GTF2E1	1
STXBP5L	1
POLQ	5
GOLGB1	6
IQCB1	3
EAF2	2
CD86	1
CSTA	2
CCDC58	2
FAM162A	3
WDR5B	1
KPNA1	4
PARP9	2
HSPBAP1	3
DIRC2	2
PDIA5	2
SEC22A	5
ADCY5	2
HACD2	5
LOC106823713	8
MYLK	4
TIMMDC1	6
ADPRH	4
PCDHB2	1
LOC106823776	6
HARS2	4

HARS	6
DND1	2
IK	3
NDUFA2	2
CD14	7
SLC35A4	1
APBB3	1
SRA1	6
EIF4EBP3	4
LOC106823762	4
HBEGF	4
PFDN1	1
LOC106823788	6
ETFDH	4
PPID	2
FNIP2	6
LOC106823785	1
RAPGEF2	6
SNRPB2	5
FAM193A	8
RNF4	2
MXD4	8
POLN	6
HAUS3	3
LOC106823799	9
NELFA	2
WHSC1	5
YIPF6	5
ACAD9	3
KIAA1257	5
LOC106823818	2
LOC106823816	8
ARHGAP17	2
TNRC6A	2
RBBP6	5
PRKCB	2
HTRA2	5
AUP1	6
TLX2	1
PCGF1	5
LOC106823843	2
MOGS	6
WBP1	4
RTKN	4

WDR54	5
LOC106823857	5
DCTN1	2
MTHFD2	5
LOC106823840	1
LOC106823841	1
STAMBP	4
DGUOK	2
TET3	2
BOLA3	5
MOB1A	2
XPNPEP1	4
LOC106823878	7
OXSM	5
NGLY1	8
TOP2B	1
LOC106823885	1
LOC106823886	8
NR1D2	6
RPL15	2
NKIRAS1	5
UBE2E1	2
LOC106823889	2
ZNF385D	2
EXOC2	5
IRF4	2
DUSP22	1
DPP8	6
PARP16	2
CLPX	9
PDCD7	2
UBAP1L	2
KBTBD13	7
RASL12	9
SLC51B	2
SPG21	2
PLEKHO2	6
PIF1	1
RBPMS2	2
OAZ2	5
ZNF609	6
PLOD2	2
LOC106823930	2
NIPA1	2

NIPA2	5
CYFIP1	4
TUBGCP5	6
LOC106823931	1
LOC106823932	1
LOC106823933	8
LOC106823949	3
RANBP2	6
LIMS1	2
GCC2	2
LOC106823940	8
SOWAHC	4
SEPT10	5
SH3RF3	1
LOC106823955	5
CXADR	4
BTG3	2
LOC106823954	1
SYTL4	2
TSPAN6	9
PCDH19	2
LOC106823978	5
ZNF667	4
ZNF583	8
LOC106823987	1
LARP1	2
FAXDC2	1
LOC106824004	5
CNOT8	3
GEMIN5	2
MRPL22	5
LOC106824031	9
LOC106824060	1
TTLL7	5
PRKACB	6
SAMD13	10
LOC106824054	7
LOC106824012	7
RPF1	5
GNG5	5
SPATA1	1
CTBS	5
SSX2IP	9
LPAR3	2

WDR63	5
SYDE2	2
LOC106824037	5
BCL10	2
CYR61	2
ZNHIT6	2
COL24A1	6
AP3B1	2
LOC106824039	7
TBCA	3
WDR41	6
AGGF1	2
F2R	4
IQGAP2	1
POC5	3
POLK	6
COL4A3BP	2
HMGCR	6
ANKRD31	4
GCNT4	4
FAM169A	2
NSA2	2
HEXB	1
ENC1	2
LOC106824064	8
SUMO1	3
NOP58	4
BMPR2	2
FAM117B	2
ICA1L	5
WDR12	3
NBEAL1	2
LOC106824078	7
LOC106824075	5
FRMD3	4
LOC106824087	7
LOC106824081	1
LOC106824086	7
LOC106824088	8
MED6	6
MAP3K9	2
PCNX	2
TSSC4	2
CD81	2

ASCL2	2
TH	2
IGF2	2
MRPL23	2
LSP1	2
CTSD	2
SCHIP1	2
LOC106824112	2
PDK3	2
POLA1	2
ARX	2
IRAK3	1
HELB	2
GRIP1	1
BRD7	3
ADCY7	6
PAPD5	8
HEATR3	2
CNEP1R1	3
LOC106824132	1
LOC106824131	8
LOC106824135	9
LANCL2	5
EGFR	2
RCN3	9
NOSIP	5
PRRG2	6
IRF3	7
BCL2L12	5
PRMT1	2
CPT1C	7
TSKS	1
AP2A1	4
MED25	4
PTOV1	2
NUP62	3
ATF5	1
VRK3	8
ZNF473	5
IZUMO2	1
POLD1	2
ATP6V1G1	3
LOC106824205	2
LOC106824207	7

TNFSF8	1
PAPPA	6
ASTN2	6
TRIM32	6
CDK5RAP2	5
MEGF9	6
FBXW2	2
PSMD5	2
LOC106824211	1
PHF19	2
CNTRL	5
RAB14	2
GSN	2
STOM	4
LOC106824202	5
LOC106824200	1
LOC106824199	1
LOC106824194	1
DAB2IP	2
TTLL11	8
NHLRC3	4
RUNX1T1	2
LOC106824239	2
CLDND1	5
CPOX	2
ST3GAL6	2
DCBLD2	4
LOC106824275	1
NCOA4	7
LOC106824267	5
PARG	4
OGDHL	2
SLC18A3	9
TSGA13	7
WDR60	5
LOC106824290	1
ESYT2	4
NCAPG2	3
LOC106824284	1
CDCA2	5
KCTD9	4
DOCK5	4
LOC106824296	5
LOC106824316	2

RARRES2	2
LRRC61	3
ZBED6CL	2
ATP6V0E2	2
ZNF777	8
ZNF783	2
ZNF212	1
ZNF398	4
LRRC72	7
ANKMY2	2
BZW2	2
TSPAN13	2
AGR3	1
AHR	2
SNX13	4
LOC106824346	5
PRPS1L1	5
HDAC9	8
TWIST1	2
TWISTNB	2
LOC106824332	1
LOC106824379	5
SP4	2
CDCA7L	2
RAPGEF5	6
LOC106824333	7
TOMM7	2
FAM126A	5
KLHL7	4
NUPL2	5
GPNMB	2
MALSU1	3
TRA2A	1
CCDC126	5
FAM221A	1
GNS	4
RASSF3	2
TBK1	2
XPOT	3
LOC106824385	5
LOC106824387	1
SRGAP1	4
ZNF831	1
PRELID3B	5

ATP5E	2
TUBB1	1
LOC106824409	6
LOC106824408	4
LOC106824410	1
LOC106824413	1
LOC106824411	1
LOC106824396	1
LOC106824405	4
LOC106824403	6
LOC106824407	2
LOC106824414	6
LOC106824416	5
RUBCN	4
FYTTD1	3
LRCH3	6
IQCG	5
RPL35A	2
HTRA1	2
PLEKHA1	6
BTBD16	8
TACC2	2
NSMCE4A	5
LOC106824439	1
LOC106824454	5
PFKL	2
DNMT3L	2
LOC106824444	2
LOC106824443	2
TRAPPC10	1
AGPAT3	7
LOC106824448	8
RRP1	4
LOC106824464	5
HNRNPU	2
LOC106824460	4
LOC106824463	5
DESI2	8
LOC106824462	5
SERPINB8	2
LOC106824474	7
LOC106824490	4
LOC106824485	2
LOC106824483	9

LOC106824484	2
LOC106824489	5
LOC106824494	6
LOC106824495	8
LOC106824499	3
LOC106824496	4
ERAL1	2
FLOT2	7
PHF12	6
LOC106824546	4
TRERF1	2
MRPS10	3
GUCA1A	7
LOC106824520	1
CCND3	2
MED20	6
BYSL	2
USP49	8
TOMM6	2
FRS3	2
MDFI	2
LOC106824529	1
NCR2	1
TREML2	7
LOC106824540	1
NFYA	5
OARD1	3
APOBEC2	4
UNC5CL	6
LOC106824524	8
MOCS1	1
KIF6	1
SAYSD1	5
DNAH8	5
GLO1	2
LOC106824584	5
BTBD9	9
ZFAND3	7
CCDC167	7
CMTR1	2
RNF8	6
TBC1D22B	6
TMEM217	5
PIM1	7

MTCH1	2
LOC106824553	4
PPIL1	2
CPNE5	6
RAB44	7
LOC106824595	7
SRSF3	3
STK38	6
KCTD20	2
PXT1	2
ETV7	1
BRPF3	6
MAPK13	3
MAPK14	6
SLC26A8	5
SRPK1	5
LHFPL5	1
CLPS	7
ARMC12	1
FKBP5	2
LOC106824618	6
GNL3L	2
LOC106824623	5
ERCC8	1
ELOVL7	2
DEPDC1B	2
PHGDH	2
HMGCS2	9
ADAM30	5
NOTCH2	2
LOC106824638	8
SEC22B	2
AMMECR1	2
HDX	4
ASH2L	5
LSM1	2
BAG4	1
DDHD2	2
PLPP5	1
WHSC1L1	6
LETM2	1
FGFR1	2
LOC106824675	8
ODF2L	5

NMI	2
RIF1	2
NEB	1
ARL5A	2
STAM2	3
FMNL2	1
PRPF40A	3
ARL6IP6	5
RPRM	2
RPL37	2
PRKAA1	7
TTC33	6
DAB2	2
FYB	1
RICTOR	2
OSMR	2
LIFR	2
EGFLAM	6
WDR70	6
NUP155	8
LOC106824791	4
LOC106824755	4
NIPBL	4
NADK2	1
SKP2	2
LMBRD2	6
LOC106824768	1
SPEF2	5
AGXT2	7
DNAJC21	5
LOC106824758	8
BRIX1	2
RAD1	2
TTC23L	1
RAI14	2
AMACR	2
TARS	2
IZUMO3	5
ELAVL2	2
CDKN2B	5
LOC106824796	3
LOC106824712	1
LOC106824742	1
NDRG1	2

TG	7
LOC106824815	1
PHF20L1	5
LOC106824828	1
LRRC6	5
KCNQ3	2
EFR3A	4
ASAP1	1
FAM49B	2
LOC106824805	8
ZBTB33	2
LAMP2	2
CUL4B	9
C1GALT1C1	6
PSD3	1
SH2D4A	2
CT83	7
ARHGEF9	4
ZMAT1	2
LOC106824891	4
KIAA1211L	6
TSGA10	5
LIPT1	5
MRPL30	2
MITD1	2
LRRIQ3	1
LOC106824901	1
ERICH3	1
CRYZ	4
TYW3	2
TMEM47	2
DCDC5	1
MPPED2	2
ARL14EP	2
KCNA4	4
KIF18A	5
LIN7C	2
CCDC34	10
ANO3	1
FSIP2	5
NCOA6	4
TP53INP2	1
PIGU	6
MAP1LC3A	7

DYNLRB1	2
ITCH	2
LOC106824939	4
LOC106824940	2
ASIP	1
SCAF8	2
LOC106824948	2
AP2A2	4
CHID1	2
TSPAN4	2
POLR2L	10
CD151	2
PNPLA2	5
RPLP2	2
SLC25A22	9
PDDC1	2
TALDO1	2
TMEM80	2
DEAF1	2
SCT	7
IRF7	5
PHRF1	6
RASSF7	2
LMNTD2	1
LRRC56	5
HRAS	2
LOC106824986	2
EAPP	3
CCDC124	10
KCNN1	1
ARRDC2	2
MAST3	6
PIK3R2	2
IFI30	9
LOC106825019	2
KIAA1683	8
JUND	2
LSM4	2
PGPEP1	1
ISYNA1	5
ELL	2
FKBP8	9
KXD1	2
UBA52	7

LOC106825004	2
CRLF1	4
TMEM59L	2
KLHL26	4
LOC106825024	2
LOC106825028	9
DDX39B	2
ATP6V1G2	1
NFKBIL1	1
LTA	1
LTB	9
LST1	7
PRRC2A	3
BAG6	2
APOM	5
GPANK1	5
CSNK2B	5
ABHD16A	5
LY6G6C	7
DDAH2	9
CLIC1	2
VARS	7
LSM2	3
HSPA1L	7
GNG12	2
GADD45A	2
SERBP1	3
LOC106825070	1
RND3	2
MMADHC	5
KIF5C	6
EPC2	2
MBD5	4
ORC4	3
ACVR2A	4
LOC106825093	1
ZEB2	2
BCL3	2
ZNF180	2
ZNF112	4
ZNF235	2
ZNF227	4
LOC106825107	4
ZNF45	3

GADD45G	2
LOC106825123	2
LOC106825121	4
OFD1	6
FAM111B	2
FAM111A	2
LOC106825145	5
KLHL13	2
LOC106825152	1
LOC106825156	1
PSMC2	3
RELN	2
ORC5	6
KMT2E	2
SRPK2	8
PUS7	2
LOC106825223	6
RINT1	2
LOC106825222	5
ATXN7L1	1
SYPL1	5
NAMPT	2
PIK3CG	1
PRKAR2B	4
HBP1	2
COG5	6
DUS4L	2
BCAP29	5
CBLL1	2
DLD	4
LAMB1	2
PNPLA8	2
NME8	5
STARD3NL	2
AMPH	2
LOC106825229	4
LOC106825262	7
USPL1	6
HMGB1	2
LOC106825252	1
LOC106825283	1
LOC106825281	1
ZNF350	4
ZNF615	2

LOC106825278	1
ZNF432	1
LOC106825276	1
LOC106825286	5
LOC106825268	1
LOC106825287	1
LOC106825288	1
LOC106825269	1
MUC5B	2
TOLLIP	8
BRSK2	4
MOB2	2
CSTB	2
PDXK	2
RRP1B	5
HSF2BP	5
SIK1	6
LOC106825309	8
LOC106825325	2
LOC106825328	2
PPP4R4	1
LOC106825359	10
LOC106825356	2
LOC106825358	1
LOC106825355	9
DDX24	2
OTUB2	7
LOC106825340	7
ASB2	5
FAM181A	5
UNC79	4
BTBD7	2
UBR7	10
LOC106825386	5
TMEM251	2
LOC106825392	4
CHGA	2
LOC106825346	1
GOLGA5	4
LGMN	2
CPSF2	4
NDUFB1	5
ATXN3	5
TRIP11	6

FBLN5	2
CATSPERB	1
PPP4R3A	2
CCDC88C	4
RPS6KA5	4
CALM1	5
NRDE2	6
PSMC1	3
TDP1	5
EFCAB11	4
LOC106825336	6
LOC106825411	1
FANK1	5
DHX32	5
BCCIP	2
UROS	6
LOC106825344	5
MMP21	1
EDRF1	4
LOC106825370	1
LOC106825343	1
TEX36	7
CTBP2	4
ZRANB1	6
PIGG	6
PDE6B	8
LOC106825438	6
PCGF3	4
CPLX1	7
GAK	7
LOC106825428	1
TMEM175	5
IDUA	2
FGFRL1	2
RNF212	5
LOC106825421	1
PARD6G	2
ADNP2	1
RBFA	2
LOC106825444	2
TXNL4A	5
PQLC1	7
KCNG2	5
LOC106825450	7

BORCS8	1
RFXANK	1
NR2C2AP	4
SUGP1	6
MAU2	4
GATAD2A	4
TSSK6	7
NDUFA13	2
YJEFN3	2
LOC106825459	1
LPAR2	2
GMIP	6
ATP13A1	4
FAT4	2
LOC106825493	5
LOC106825491	5
RPP21	2
GNL1	5
PRR3	2
ABCF1	9
PPP1R10	6
MRPS18B	6
ATAT1	1
LOC106825484	2
DHX16	2
PPP1R18	1
NRM	1
MDC1	5
TUBB	2
FLOT1	2
IER3	2
DDR1	2
LOC106825501	6
GTF2H4	5
VARS2	5
USP22	2
DHRS7B	6
TMEM11	2
LOC106825521	8
NATD1	5
MAP2K3	2
ZFP62	4
MGAT1	6
LOC106825526	5

PLEKHA5	2
AEBP2	2
PYROXD1	2
GOLT1B	4
LDHB	2
CMAS	2
ST8SIA1	6
C2CD5	1
ETNK1	1
SOX5	1
BCAT1	2
CASC1	5
LYRM5	5
KRAS	2
OCA2	1
SPATS2L	5
SGOL2	3
LOC106825581	2
LOC106825601	2
LOC106825600	4
LOC106825604	4
LOC106825605	2
LOC106825595	2
FLNA	2
TKTL1	2
TEX28	7
IRAK1	4
NAA10	2
L1CAM	2
SSR4	2
IDH3G	2
RRAGD	2
ANKRD6	4
SCML1	6
MKI67	5
HMGXB4	6
TOM1	5
LOC106825683	8
HMOX1	2
MCM5	2
RASD2	2
MB	1
RBFOX2	6
GCNT1	2

VPS13A	6
GNAQ	2
MINK1	2
CHRNE	7
LOC106825695	7
SLC25A11	9
RNF167	3
PFN1	2
ENO3	9
SPAG7	3
CAMTA2	6
INCA1	2
ZFP3	1
RABEP1	2
NUP88	3
RPAIN	5
C1QBP	2
DHX33	1
DERL2	5
MIS12	4
LOC106825746	5
FAM64A	1
KIAA0753	1
TXNDC17	3
MED31	4
FBXO39	7
TEKT1	5
SMTNL2	2
MYBBP1A	2
UBE2G1	2
ANKFY1	4
LOC106825674	1
ZZEF1	2
ATP2A3	2
NCBP3	4
ITGAE	5
GSG2	5
P2RX5	1
EMC6	5
TAX1BP3	2
CTNS	8
TRPV1	1
LOC106825661	2
LOC106825756	1

LOC106825776	1
LOC106825763	1
LOC106825782	1
LOC106825771	1
LOC106825765	7
HTATSF1	2
ARHGEF6	9
RBMX	4
ESF1	2
NDUFAF5	5
SEL1L2	8
RUFY2	1
HNRNPH3	2
LOC106825803	1
TRIM41	1
GNB2L1	2
EFCAB2	5
LOC106825816	7
TRAPPC2	6
RAB9A	2
EGFL6	2
ANKEF1	7
MKKS	2
GRK4	5
NOP14	3
MFSD10	3
ADD1	2
SH3BP2	2
TNIP2	2
METTL4	6
NDC80	5
SMCHD1	2
LPIN2	2
GHITM	10
LOC106825899	1
CCSER2	9
LOC106825902	1
CPEB4	4
LOC106825851	6
CRIM1	2
FEZ2	4
STRN	2
HEATR5B	6
GPATCH11	5

EIF2AK2	2
CEBPZ	4
NDUFAF7	1
PRKD3	2
QPCT	2
LOC106825852	1
CDC42EP3	2
RMDN2	1
ATL2	5
HNRNPLL	3
SRSF7	3
LOC106825873	8
GEMIN6	7
DHX57	6
MORN2	5
ARHGEF33	5
SOS1	2
CDKL4	1
MAP4K3	7
LOC106825846	2
TMEM178A	2
THUMPD2	4
SLC8A1	2
DCLK1	2
SOHLH2	6
MANBA	6
LOC106825955	5
PCDH9	2
AMDHD2	2
PDPK1	9
KCTD5	2
PRSS21	7
LOC106825960	9
LOC106825961	7
TCEB2	9
SRRM2	4
FLYWCH2	5
PKMYT1	5
LOC106826076	1
CLDN6	2
HCFC1R1	3
THOC6	2
CCDC64B	1
IL32	7

ZNF213	4
ZNF200	6
ZNF263	6
TIGD7	6
ZNF75A	1
ZNF174	2
ZNF597	4
NAA60	4
LOC106826082	1
CLUAP1	1
SLX4	5
TRAP1	2
CREBBP	4
ADCY9	2
TFAP4	9
GLIS2	7
PAM16	3
LOC106825968	5
DNAJA3	3
NMRAL1	5
HMOX2	5
CDIP1	7
LOC106826037	1
UBALD1	2
MGRN1	2
NUDT16L1	3
LOC106826067	5
SEPT12	5
SMIM22	10
ROGDI	5
GLYR1	4
UBN1	3
SEC14L5	7
NAGPA	2
ALG1	2
RBFOX1	1
LOC106826014	1
TXNDC12	7
RAB3B	4
NRDC	7
OSBPL9	5
EPS15	2
TTC39A	1
RNF11	7

LOC106825974	5
CDKN2C	2
FAF1	1
KCNQ5	7
NPR3	5
LOC106826096	1
CCNA1	5
SPG20	4
CCDC169	1
RAB21	5
TBC1D15	5
LOC106826112	7
LOC106826120	5
CARS	3
NAP1L4	9
PHLDA2	2
CDKN1C	9
GPBP1L1	4
NASP	3
AKR1A1	2
PRDX1	2
MMACHC	5
LOC106826138	3
TESK2	5
TOE1	5
MUTYH	6
HPDL	6
LOC106826142	1
BICC1	2
PHYHIPL	5
CCDC6	2
ANK3	6
CDK1	3
RHOBTB1	7
LOC106826154	5
ARID5B	6
RTKN2	1
ADO	5
EGR2	2
NRBF2	2
JMJD1C	3
REEP3	5
LOC106826183	7
LOC106826209	5

PACS2	4
TEX22	7
LOC106826217	1
MTA1	5
CRIP2	1
CRIP1	9
LOC106826221	2
SLC2A5	1
GPR157	2
NEFL	2
NEFM	5
RPL14	2
ZNF621	5
LOC106826235	2
LOC106826238	5
LOC106826239	5
PRMT2	2
DIP2A	2
LOC106826241	4
TMTC2	6
METTL25	2
CCDC59	3
LIN7A	5
KIF2B	7
UTP18	2
MBTD1	5
LOC106826470	2
LOC106826468	2
SPAG9	1
TOB1	2
LUC7L3	2
ANKRD40	7
SPATA20	8
MYCBPAP	5
RSAD1	2
ACSF2	2
LRRC59	5
EME1	1
MRPL27	2
XYLT2	6
COL1A1	2
SGCA	8
LOC106826323	8
PDK2	7

DLX4	6
KAT7	3
FAM117A	2
SLC35B1	3
SPOP	6
PHB	2
ZNF652	6
PHOSPHO1	7
IGF2BP1	4
SNF8	2
ATP5G1	2
CALCOCO2	8
TTLL6	1
LOC106826348	2
HOXB6	2
LOC106826386	2
HOXB3	4
HOXB2	2
SKAP1	2
SNX11	2
CBX1	2
NFE2L1	9
CDK5RAP3	5
PNPO	5
SP2	6
SCRN2	1
LRRC46	5
MRPL10	5
OSBPL7	6
KPNB1	2
LOC106826545	5
NPEPPS	3
MRPL45	3
SOCS7	7
ARHGAP23	2
LOC106826548	8
MLLT6	4
CISD3	9
PCGF2	2
PSMB3	2
PIP4K2B	6
CWC25	6
LOC106826272	1
RPL23	2

LASP1	1
ARL5C	9
RPL19	2
FBXL20	6
MED1	3
CDK12	6
STARD3	2
PGAP3	6
MIEN1	2
GRB7	1
IKZF3	4
ZPBP2	5
ORMDL3	8
PSMD3	3
MED24	4
THRA	2
NR1D1	1
LOC106826575	8
MSL1	1
CASC3	2
WIPF2	6
CDC6	4
TOP2A	5
SMARCE1	3
KRT222	1
KRT25	2
KRT28	2
KRT10	5
LOC106826304	1
KRT19	9
KRT14	2
EIF1	9
HAP1	2
JUP	2
P3H4	2
NT5C3B	5
KLHL10	8
KLHL11	2
ACLY	2
TTC25	5
CNP	4
DNAJC7	4
NKIRAS2	4
KAT2A	2

HSPB9	10
RAB5C	2
STAT5B	2
STAT3	2
PTRF	9
NAGLU	1
COASY	3
MLX	2
PSMC3IP	5
FAM134C	7
TUBG1	2
LOC106826279	2
EZH1	2
RAMP2	2
VPS25	6
WNK4	2
LOC106826429	5
CNTD1	1
BECN1	2
PSME3	7
LOC106826355	7
LOC106826365	2
PTGES3L	1
RUNDC1	1
RPL27	2
VAT1	4
BRCA1	1
NBR1	7
TMEM106A	1
LOC106826571	7
HOXD9	2
HOXD8	5
HOXD4	2
LOC106826595	8
MTX2	3
HNRNPA3	3
NFE2L2	2
LOC106826600	8
AGPS	2
TTC30B	5
LOC106826627	1
RBM45	5
PRKRA	2
FKBP7	5

PLEKHA3	7
TTN	4
CCDC141	1
SESTD1	2
ZNF385B	2
CWC22	2
UBE2E3	2
CERKL	1
SSFA2	2
LOC106826614	6
PDE1A	1
DNAJC10	1
RPL10	2
ATP6AP1	2
GDI1	2
FAM50A	2
LAGE3	3
UBL4A	2
BGN	9
FAM58A	2
DUSP9	2
BCAP31	2
LOC106826673	2
ZKSCAN2	2
AQP8	9
LCMT1	2
IKZF5	6
PSTK	4
LOC106826676	5
LOC106826684	6
FAM24B	5
CUZD1	5
LOC106826680	7
RNGTT	1
PYGB	2
ABHD12	5
GINS1	4
NINL	2
NANP	5
LOC106826695	1
LOC106826700	9
LOC106826701	5
LOC106826704	4
LOC106826705	1

BMPR1B	4
UNC5C	6
PDHA2	5
LOC106826713	1
BORCS5	3
DUSP16	2
CREBL2	2
DDX47	2
GPRC5A	7
HEBP1	2
FAM234B	2
GSG1	7
LOC106826727	1
EMP1	4
LOC106826751	5
GRIN2B	6
LOC106826746	1
ATF7IP	4
PLBD1	4
LOC106826752	5
WBP11	5
LOC106826736	8
MGP	9
ARHGDIB	2
RERG	2
EPS8	2
STRAP	5
DERA	2
PLS3	2
PSMC6	2
STYX	5
GNPNAT1	5
FERMT2	2
DDHD1	6
LOC106826767	1
DGCR2	6
TSSK1B	5
TSSK2	8
DGCR14	3
SLC25A1	2
UFM1	4
SPARC	2
SLC36A1	1
SLC36A3	7

LOC106826818	2
WDR74	2
LOC106826822	8
STX5	8
NXF1	4
TMEM223	10
TMEM179B	5
POLR2G	3
ZBTB3	1
TTC9C	5
HNRNPUL2	6
BSCL2	5
GNG3	5
UBXN1	3
METTL12	2
LOC106826837	5
B3GAT3	2
EML3	6
MTA2	2
TUT1	5
EEF1G	7
ASRGL1	5
RECK	1
GLIPR2	2
CLTA	2
GNE	4
RNF38	7
GPX3	2
TNIP1	2
CCDC69	6
GM2A	2
LOC106826864	6
HSDL2	7
PTBP3	2
UGCG	4
LOC106826916	5
LOC106826879	4
GNG10	2
DNAJC25	4
PTGR1	2
ZNF483	4
KIAA0368	6
LPAR1	6
MUSK	1

LOC106826910	8
LOC106826905	2
AKAP2	4
LOC106826918	5
PTPN3	2
EPB41L4B	4
FRRS1L	2
TMEM245	2
CTNNAL1	2
FAM206A	3
IKBKAP	4
ACTL7A	8
ACTL7B	1
KLF4	6
RAD23B	2
ZNF462	2
LOC106826887	2
TMEM38B	5
FKTN	4
FSD1L	5
SLC44A1	2
LOC106826877	2
LOC106826919	5
LOC106826942	1
BEST2	1
ASNA1	5
LOC106826927	3
LOC106826930	1
FBXW9	5
LOC106826929	8
DHPS	5
WDR83	4
WDR83OS	2
MAN2B1	4
LOC106826940	8
LOC106826939	1
LOC106826934	6
LOC106826932	1
LOC106826931	1
LOC106826937	2
SPACA1	5
LOC106826945	4
SLC10A6	7
LOC106826948	7

LOC106826950	1
CDV3	7
LOC106826952	2
LOC106826953	2
LOC106826955	2
LOC106826951	2
CCDC22	2
GPKOW	2
WDR45	9
CCDC120	2
GRIPAP1	7
KCND1	8
OTUD5	4
PIM2	6
PQBP1	2
TIMM17B	2
ERAS	4
HDAC6	6
KTN1	3
ATG14	2
FBXO34	1
OSR2	2
LOC106827002	2
LOC106827012	2
BHLHB9	2
GPRASP2	2
GPRASP1	2
LOC106827032	2
TMEM14A	2
TRAM2	2
EFHC1	5
MCM3	2
TFAP2B	5
LOC106827019	1
PGK2	5
CRISP2	10
LOC106827044	2
CENPQ	3
MUT	2
LOC106827023	5
CD2AP	6
LOC106827065	1
ZNF37A	4
LOC106827061	4

LOC106827063	2
LOC106827066	5
VAMP7	2
LOC106827082	1
LOC106827083	2
EEF1A1	2
MTO1	6
LOC106827080	8
MB21D1	2
DDX43	2
CC2D2A	8
FBXL5	3
FAM200B	1
CD38	1
LOC106827095	8
CDKL5	1
SNX6	2
CFL2	2
BAZ1A	6
LOC106827100	1
SRP54	6
LOC106827103	5
LOC106827108	6
GDPD2	1
LOC106827110	2
PROX1	6
LOC106827187	1
RPS6KC1	6
ANGEL2	2
VASH2	1
FLVCR1	1
SPATA45	5
TATDN3	6
NSL1	7
BATF3	2
FAM71A	7
ATF3	2
NENF	9
TMEM206	2
PPP2R5A	8
DTL	5
INTS7	2
LPGAT1	5
NEK2	5

SLC30A1	6
LOC106827196	7
RCOR3	7
SYT14	2
DIEXF	2
IRF6	2
LOC106827161	2
TRAF3IP3	4
G0S2	9
ZNF346	1
NSD1	2
RAB24	2
PRELID1	2
LMAN2	3
PFN3	1
PRR7	4
DBN1	4
PDLIM7	2
DDX41	2
FAM193B	2
LOC106827181	2
B4GALT7	8
N4BP3	9
RMND5B	5
NHP2	4
HNRNPAB	4
PHYKPL	5
CLK4	1
LOC106827164	2
LOC106827206	2
LOC106827205	2
LOC106827207	8
LOC106827209	1
MTMR12	4
ZFR	6
SUB1	3
LOC106827217	2
LOC106827218	6
SALL3	2
LOC106827221	4
REV1	2
LOC106827223	2
SERPINB6	3
LOC106827242	1

LOC106827249	5
LOC106827250	5
IGF1	1
NUP37	2
CCDC53	5
DRAM1	1
GNPTAB	2
SYCP3	5
CHPT1	10
LOC106827285	5
MYBPC1	2
ARL1	5
LOC106827259	4
UTP20	4
GAS2L3	6
SCYL2	5
DEPDC4	1
ACTR6	3
UHRF1BP1L	6
LOC106827274	1
APAF1	4
IKBIP	2
SLC25A3	2
TMPO	4
LOC106827255	1
NEDD1	5
CFAP54	1
LOC106827315	2
RBAK	4
LOC106827324	1
RNF216	2
FSCN1	1
ACTB	2
IL13RA2	5
LOC106827334	5
LOC106827339	6
LOC106827341	7
PHF8	6
LRIF1	5
LAMTOR5	2
RBM15	6
KCNC4	2
LOC106827421	1
UBL4B	7

STRIP1	2
AHCYL1	2
EPS8L3	9
GSTM3	5
LOC106827366	2
LOC106827367	2
AMPD2	2
GNAI3	2
AMIGO1	9
LOC106827378	4
PSMA5	3
SORT1	1
SARS	3
KIAA1324	5
LOC106827369	5
TMEM167B	4
TAF13	2
WDR47	4
CLCC1	5
GPSM2	2
AKNAD1	7
PRPF38B	3
LOC106827380	1
HENMT1	2
FAM102B	2
LOC106827425	1
LOC106827404	2
VAV3	6
NTNG1	2
PRMT6	4
ENTPD6	2
LOC106827372	1
SLC39A10	2
DNAH7	5
STK17B	5
FTSJ1	2
EBP	4
TBC1D25	2
RBM3	2
WDR13	2
ITGAV	2
ZC3H15	4
KDM6A	2
MAGEE2	1

PBDC1	2
LOC106827456	1
SLTM	5
RNF111	6
CCNB2	5
RBM41	2
NUP62CL	2
PIH1D3	6
LOC106827464	10
AKR1E2	5
LOC106827466	2
LOC106827471	2
LOC106827473	2
LOC106827474	2
LOC106827475	4
JRKL	1
CCDC82	1
MAML2	1
MTMR2	1
CEP57	1
FAM76B	1
SESN3	1
ENDOD1	6
LOC106827509	5
CWC15	2
AMOTL1	5
PIWIL4	2
LOC106827525	5
ANKRD49	5
MRE11A	6
MED17	6
LOC106827493	1
TAF1D	5
CEP295	1
LOC106827516	5
CCDC67	5
LOC106827488	5
SLC36A4	1
LOC106827486	2
FAT3	1
CHORDC1	2
NAALAD2	2
TANGO2	2
DGCR8	6

TRMT2A	7
RANBP1	5
CCDC188	7
RTN4R	2
LOC106827532	2
DGCR6L	2
LOC106827544	4
LOC106827543	6
LOC106827547	5
LOC106827560	1
ARC	2
GML	2
LOC106827565	2
CFAP126	5
SDHC	2
NDUFS2	3
B4GALT3	2
PPOX	4
USP21	6
UFC1	5
DEDD	5
NIT1	3
PFDN2	3
KLHDC9	5
USF1	2
TSTD1	7
TDRD3	3
LOC106827613	1
MELK	5
LOC106827616	7
LOC106827617	7
ZNF184	4
ZNF391	1
LOC106827621	1
POM121L2	5
PRSS16	5
CHMP7	9
R3HCC1	5
ENTPD4	2
LOC106827636	7
LOC106827646	9
LOC106827644	2
LOC106827643	9
LOC106827642	6

PDZD11	2
IGBP1	6
NCKAP1	2
FSCB	8
LOC106827735	8
LOC106827737	1
LOC106827724	7
KLHL28	6
FAM179B	6
PRPF39	4
FKBP3	9
FANCM	2
MIS18BP1	3
RPL10L	1
MDGA2	1
LOC106827699	5
LOC106827697	7
BATF	4
FOS	2
LOC106827730	5
TMED10	2
NEK9	2
ZC2HC1C	5
ACYP1	5
MLH3	2
EIF2B2	3
DLST	4
YLPM1	6
FCF1	2
AREL1	4
ISCA2	5
NPC2	2
LIN52	1
ALDH6A1	6
BBOF1	5
ENTPD5	1
COQ6	5
FAM161B	6
ZNF410	2
PTGR2	2
PNMA1	6
DNAL1	5
LOC106827717	8
NUMB	4

PSEN1	5
LOC106827685	8
RBM25	3
ZFYVE1	7
DCAF4	5
RGS6	1
CEP135	5
LOC106827748	2
CEBPG	5
LRP3	2
WDR88	5
LOC106827752	4
TSPYL2	2
LOC106827762	1
PDCD2	3
TBP	3
PSMB1	2
LOC106827777	2
PIGH	5
PLEK2	2
EIF2S1	3
ATP6V1D	5
MPP5	4
LOC106827808	9
