# Supplementary material for: Single-Cell RNA Sequencing Reveals Dynamic Transcriptional Landscape of Testicular Maturation in Dezhou Donkeys
Source: Animals (Basel). 2026 May 26;16(11):1621. doi: 10.3390/ani16111621 (PMC13255784; doi:10.3390/ani16111621)
Supplement: Supplementary file 1 [file animals-16-01621-s001.zip › Table s3.pdf]

	Gene_Clusters
BANP	1
SLC7A5	1
MAP1LC3B	1
FBXO31	1
MTHFSD	1
LOC106829176	1
EMC8	1
LOC106829317	1
GINS2	1
GSE1	2
USP10	2
KLHL36	1
COTL1	2
TLDC1	1
WFDC1	1
ADAD2	1
TAF1C	2
DNAAF1	1
MBTPS1	2
OSGIN1	1
MLYCD	1
HSBP1	1
PLCG2	2
GCSH	1
LOC106833328	1
CMC2	1
DYNLRB2	1
WWOX	1
NUDT7	2
TERF2IP	2
GABARAPL2	1
TMEM170A	1
CFDP1	2
BCAR1	2
LDHD	1
ZNRF1	1
GLG1	2
NDUFC1	1
SETD7	1
MGST2	2
MAML3	2
SCOC	1
CLGN	1

ELMOD2	1
TBC1D9	2
RNF150	2
ZNF330	2
IL15	2
LOC106841444	2
LOC106841137	1
USP53	1
SYNPO2	2
METTL14	1
LOC106842379	2
CAMK2D	1
LARP7	1
ZC3H7A	2
RSL1D1	1
GSPT1	1
SNX29	1
CPPED1	1
HDGFL1	1
SOX4	2
ID4	1
ZFPM2	2
DCAF13	1
SLC25A32	1
FZD6	2
ATP6V1C1	1
AZIN1	2
KLF10	2
ODF1	1
NCALD	1
PLCZ1	1
CAPZA3	1
LOC106824265	2
GMPS	1
LOC106825562	2
LOC106826008	2
PTDSS2	1
RNH1	1
ZKSCAN1	2
ZSCAN21	2
LOC106826667	2
MCM7	2
TAF6	2
LAMTOR4	2

ATP5G2	2
CALCOCO1	2
CBX5	2
HNRNPA1	2
GTSF1	1
PPP1R1A	1
CTNNB1	2
ULK4	1
DLG5	2
POLR3A	2
RPS24	2
ZMIZ1	2
PPIF	1
ZCCHC24	2
ANXA11	2
PLAC9	2
LOC106828660	2
SKIL	2
CLDN11	1
RRBP1	2
DSTN	1
ARHGEF4	2
LOC106828832	2
PLEKHB2	2
LOC106828861	2
KLHL8	2
SFT2D3	1
CCHCR1	1
MDFIC	1
LOC106829424	1
CAV2	2
CAV1	2
ASZ1	1
CTTNBP2	2
LSM8	1
MORF4L2	1
DPY30	1
LOC106829580	2
SNX5	2
BANF2	1
LOC106829728	2
LOC106829729	2
FAM3C	2
TM9SF2	2

CLYBL	1
GGACT	1
TMTC4	2
LOC106830030	1
TPP2	2
CCDC168	1
LOC106830082	2
PRPF4B	2
FAM217A	1
LOC106830266	1
ECI2	1
LOC106830318	2
LOC106830311	2
LOC106830298	2
LOC106830306	2
MAGED1	2
GSPT2	1
LOC106830356	2
ZNF75D	2
TMEM37	2
DBI	2
KCNQ1	2
FAM181B	2
PRCP	2
DDIAS	2
RAB30	2
LOC106830607	2
PCF11	2
LOC106830624	2
ANKRD42	1
CCDC90B	1
TXNDC16	1
ERO1A	2
MAP1S	2
INSL3	2
RPL18A	2
COLGALT1	2
LOC106830945	1
LOC106830988	2
GIMAP4	2
LOC106831082	2
LOC106831104	2
LOC106831048	2
LOC106831126	2

ATXN7L3B	2
GLIPR1	1
KRR1	1
ZDHHC17	2
CSRP2	2
PAWR	2
LOC106831515	1
PPP1R12A	2
LOC106831656	2
LOC106831673	1
ANXA3	1
LOC106831806	2
CD200	2
LOC106831880	2
LOC106831871	2
CH25H	2
LIPA	2
IFIT3	2
TRIM36	1
LOC106832184	1
YTHDC2	2
MCC	2
REEP5	2
SRP19	2
APC	2
LOC106832316	2
NREP	2
CAMK4	1
MAN2A1	2
PJA2	2
FER	1
LOC106832491	1
RB1CC1	2
PCMTD1	2
ERGIC2	1
TMTC1	2
PLRG1	1
RBM46	1
MAP9	2
GUCY1A3	1
GUCY1B3	1
PDGFC	2
UBAC2	1
GPR18	1

DEGS1	2
LOC106833202	2
ARL6IP1	2
SMG1	2
NEDD9	2
SMIM13	1
ELOVL2	1
PAK1IP1	1
GSTO1	2
ITPRIP	1
CFAP58	1
MCM4	2
UBE2V2	1
LOC106833614	1
RPS6	2
DENND4C	2
HAUS6	2
RRAGA	2
SH3GL2	1
DIAPH3	1
LOC106834153	1
APEX2	2
NSDHL	1
PNMA3	2
LOC106834348	2
PIN1	1
UBL5	1
FBXL12	2
NDUFA4	1
LOC106834443	2
LOC106834576	1
LOC106834569	2
ACAA2	1
MYO5B	1
CFAP53	1
MBD1	1
MRO	1
ME2	1
SMAD4	2
MEX3C	2
MBD2	2
STARD6	1
LOC106834825	1
TCF4	2

TXNL1	1
FECH	1
LOC106835049	1
PURA	2
CXXC5	2
UBE2D2	1
OTUD1	2
KIAA1217	2
ARHGAP21	2
PRTFDC1	2
ENKUR	1
ANKRD26	2
SPAG17	1
WDR3	2
GDAP2	2
FSHR	2
STON1	2
FOXN2	2
HSPH1	2
FRY	2
BRCA2	2
N4BP2L1	2
N4BP2L2	2
PDS5B	2
STARD13	2
RFC3	1
CDK17	2
LOC106835979	1
LOC106835990	2
LUZP2	1
LOC106836067	1
LOC106836085	1
ANXA1	2
ALDH1A1	2
ZFAND5	2
LOC106836044	1
LOC106836148	1
ABHD17B	1
TMEM2	2
LOC106836190	1
LOC106836272	2
LOC106836261	2
LOC106836340	2
LOC106836363	2

LOC106836378	2
LOC106836370	2
LOC106836456	2
ACADM	1
RABGGTB	2
ASB17	1
USP33	2
FAM73A	1
NEXN	2
FUBP1	2
DNAJB4	1
IFI44L	2
IFI44	2
LOC106836908	1
MSN	2
LOC106836966	2
LOC106836973	1
LOC106836989	1
LOC106836962	2
LOC106837012	2
LOC106836995	1
DDX26B	2
LOC106837097	2
PPIB	2
SNX22	2
SNX1	1
HERC1	2
RAB8B	1
RPS27L	2
LACTB	1
LOC106837398	2
TPM1	2
TLN2	1
VPS13C	2
LOC106837466	2
RORA	2
ICE2	2
ANXA2	2
GTF2A2	1
LOC106837673	2
LOC106837682	2
LOC106837710	2
ELOVL3	2
NFKB2	1

FBXL15	1
CUEDC2	2
MFSD13A	2
SUFU	2
TRIM8	2
ARL3	1
WBP1L	2
LOC106838161	2
AS3MT	2
NT5C2	2
LYSMD2	2
DMXL2	2
AP4E1	2
LOC106838383	1
SPPL2A	2
TRPM7	2
USP50	1
USP8	1
GABPB1	2
DTWD1	2
GALK2	1
COPS2	1
SECISBP2L	2
SHC4	1
EID1	2
FBN1	2
DUT	1
MYEF2	2
SEMA6D	2
RAB43	2
CNBP	2
APOE	2
LOC106838862	2
BCAM	2
ATE1	1
RAD21	2
UTP23	1
EIF3H	1
TRPS1	1
PAK3	2
LONRF3	2
MAGED2	2
LOC106839298	2
CBFB	1

FAM96B	2
RRAD	2
PDP2	1
CA7	2
NAE1	1
DYNC1LI2	1
CMTM4	1
CMTM3	1
CMTM2	1
CKLF	1
FSCN3	1
ARF5	2
ZNF800	1
LOC106839717	1
SF3B6	2
PFN4	1
FAM228A	1
TMEM35	2
LOC106840164	1
RABIF	1
SYT2	1
PPP1R12B	2
KIZ	1
RALGAPA2	2
NAA20	1
RIN2	2
SCP2D1	1
DTD1	1
LOC106840414	2
POLR3F	1
DZANK1	1
ZNF133	2
CSRP2BP	2
TOM1L2	2
SREBF1	2
PEMT	1
MED9	1
COPS3	1
FLCN	2
PLD6	1
MPRIP	2
LOC106840842	1
LOC106840878	2
HADHA	1

RAB10	2
POMC	1
EFR3B	1
DNAJC27	1
PTRHD1	1
NCOA1	2
ITSN2	2
EGLN3	1
NPAS3	2
ARHGAP5	2
DTD2	1
HEATR5A	2
HECTD1	2
AP4S1	1
STRN3	1
COCH	2
G2E3	1
LOC106841503	2
LOC106841507	2
GABRA3	2
LOC106841657	2
LOC106841685	2
EPHA1	2
TCAF1	2
LOC106842077	1
TPK1	1
CNTNAP2	2
CUL1	1
LOC106842183	1
LOC106842173	1
PDIA4	2
ZNF425	2
MFSD14B	2
PLCXD1	2
GTPBP6	1
SLC25A6	1
IDS	2
ACVR1	2
PKP4	2
TANC1	2
WDSUB1	2
BAZ2B	2
7-Mar	2
LOC106842977	2

RBMS1	2
PSMD14	1
DPP4	1
LOC106843171	1
IFIH1	2
GCA	1
FIGN	2
GRB14	2
TTC21B	2
SCN1A	2
STK39	1
CERS6	1
NOSTRIN	1
ABCB11	2
BBS5	1
PPIG	2
CCDC173	1
PHOSPHO2	1
KLHL23	1
METTL5	1
UBR3	2
TMEM5	1
LOC106843789	2
BET1L	1
RIC8A	1
PSMD13	1
IFITM5	2
CROT	2
LOC106844060	2
RERE	2
ERRFI1	2
PARK7	1
TNFRSF9	2
UTS2	2
VAMP3	1
CAMTA1	2
LOC106844420	1
DNAJC11	1
THAP3	1
KLHL21	2
ESPN	2
ACOT7	1
RPL22	2
CHD5	1

LOC106844671	1
DFFB	1
CEP104	1
LRRC47	2
WRAP73	1
TPRG1L	1
ACTRT2	1
FAM213B	1
LOC106844873	1
LOC106844921	1
LOC106844946	2
LOC106844959	1
LOC106844970	2
LOC106844993	2
LOC106845003	2
TNRC6C	2
TMC6	2
SYNGR2	1
TK1	1
BIRC5	1
SOCS3	2
USP36	2
TIMP2	2
LGALS3BP	2
CANT1	1
ENGASE	1
CBX4	2
GAA	2
EIF4A3	2
SGSH	2
SLC26A11	2
RNF213	2
RPTOR	2
CHMP6	1
LOC106845759	1
SLC38A10	2
ACTG1	2
OXLD1	1
ARL16	1
HGS	1
MRPL12	1
FAM195B	1
P4HB	2
ARHGDIA	2

ANAPC11	1
PCYT2	1
PYCR1	2
NOTUM	2
ASPSCR1	1
STRA13	1
RAC3	1
LOC106846098	1
RFNG	1
GPS1	1
LOC106846150	2
CSNK1D	1
LOC106846237	2
OGFOD3	1
NARF	1
FOXK2	1
RAB40B	1
FN3KRP	1
B3GNTL1	2
METRNL	2
LOC106846442	1
LOC106846562	2
CCNG2	1
CCNI	2
CCDC158	1
SCARB2	2
ART3	1
CXCL11	2
CXCL10	2
SDAD1	1
NAAA	2
USO1	2
LOC106846949	2
LOC106847010	1
LOC106846994	2
ADAMTS1	2
N6AMT1	2
RWDD2B	1
USP16	1
CCT8	1
TIAM1	2
LOC106847304	1
SOD1	1
LOC106847492	1

LOC106847602	1
LOC106847667	2
LOC106847653	2
LOC106847686	1
CHD1	2
ERAP1	2
CAST	2
ELL2	2
RHOBTB3	1
SPATA9	1
RFESD	1
ARSK	2
TTC37	2
FAM81B	1
SLF1	2
LOC106848244	2
LOC106848292	1
LOC106848366	1
LOC106848622	2
MSL3	2
SKA2	1
TRIM37	1
LOC106821906	2
MTMR4	2
MKS1	1
DYNLL2	1
SRSF1	2
VEZF1	2
MSI2	2
LOC106822238	1
LOC106822285	2
LOC106822294	1
NEU1	1
EHMT2	1
LOC106822359	1
LOC106822373	2
SLC6A2	2
LPCAT2	2
MMP2	2
FTO	2
AKTIP	1
CHD9	2
CYLD	2
LOC106823033	2

STK35	1
SNRPB	1
NOP56	2
IDH3B	1
MAEL	1
ILDR2	2
LOC106823148	1
TADA1	2
POGK	2
C4BPB	2
PFKFB2	2
LOC106823339	1
DYRK3	1
RASSF5	2
SRGAP2	2
SLC41A1	2
NUCKS1	2
LEMD1	1
NUAK2	1
DSTYK	2
RBBP5	2
LRRN2	1
MDM4	1
LOC106823833	2
ETNK2	2
SOX13	2
ZC3H11A	2
ATP2B4	2
PRELP	2
BTG2	2
LOC106824201	1
LOC106824206	1
LOC106824251	1
WRN	2
TEX15	1
PPP2CB	1
GTF2E2	1
DCTN6	1
LEPROTL1	2
DUSP4	1
TNKS	1
ERI1	1
LOC106824344	2
LOC106824724	2

LOC106824728	1
LOC106824771	2
LOC106824761	2
LOC106824780	1
MRPS30	1
NNT	2
PAIP1	2
HMGCS1	2
LOC106825087	2
NPEPL1	1
RAB22A	1
LOC106825186	1
ANKRD60	1
PMEPA1	2
RAE1	1
BMP7	2
FAM209B	1
RTFDC1	1
CSTF1	1
AURKA	1
FAM210B	1
PFDN4	1
ZNF217	2
TSHZ2	2
NFATC2	1
ADNP	2
BCAS4	1
PARD6B	2
UBE2V1	1
SPATA2	1
LOC106825788	2
ZNFX1	1
DDX27	1
FBXO11	2
MSH6	2
MSH2	1
CALM2	2
LOC106826027	1
TTC7A	1
MCFD2	1
SOCS5	1
CRIPT	2
PIGF	1
ATP6V1E2	1

TMEM247	1
EPAS1	1
CAMKMT	1
PREPL	2
DYNC2LI1	1
PLEKHH2	2
ZFP36L2	2
LOC106826494	2
LOC106826590	2
LOC106826609	2
YWHAH	2
PRR14L	2
PISD	2
PATZ1	2
PIK3IP1	2
LOC106826967	1
LOC106826625	1
MORC2	1
LOC106826998	1
TCN2	2
PES1	1
CCDC157	1
TBC1D10A	1
GATSL3	1
LIF	2
MTMR3	2
ASCC2	1
LOC106827325	1
NF2	1
NIPSNAP1	1
NEFH	2
AP1B1	1
GAS2L1	1
EWSR1	2
EMID1	2
KREMEN1	2
ZNRF3	2
XBP1	2
CCDC117	2
HSCB	1
TTC28	2
PITPNB	1
LOC106827813	1
LOC106827816	1

TLE1	2
LOC106827823	1
CEP78	1
LOC106827825	1
LOC106827827	1
LOC106827833	2
IRS1	2
LOC106827855	1
MFF	1
AGFG1	1
DAW1	1
DNER	2
TRIP12	1
FBXO36	1
SP110	2
SPATA3	1
ARMC9	2
LOC106827898	2
LOC106827903	2
TMF1	1
UBA3	1
ARL6IP5	2
FRMD4B	2
FOXP1	2
EIF4E3	2
GPR27	2
RYBP	1
LOC106827948	2
LOC106827952	2
LOC106827951	2
ZNF32	2
LOC106827958	1
UBR4	2
ALDH4A1	1
ARHGEF10L	2
PADI2	2
SDHB	1
ATP13A2	2
CROCC	2
NECAP2	1
SPATA21	1
SZRD1	1
FBXO42	2
EPHA2	2

LOC106827995	1
SPEN	2
FHAD1	1
PRDM2	2
PDPN	2
LOC106828045	1
TMEM117	2
TWF1	1
PUS7L	2
BMP2K	2
PAQR3	1
GK2	1
LOC106828066	1
RASGEF1B	2
LOC106828078	1
LOC106828083	1
LOC106828084	1
DR1	1
FNBP1L	2
BCAR3	2
DNTTIP2	2
GCLM	1
ARHGAP29	2
ABCD3	2
F3	2
SLC44A3	2
CNN3	2
TMEM56	1
PTBP2	2
LOC106828123	2
LOC106828128	1
LOC106828132	1
LOC106828133	1
PARD3B	2
INO80D	2
EEF1B2	2
GPR1	1
FASTKD2	1
KLF7	1
CREB1	2
METTL21A	1
CCNYL1	1
IDH1	2
PIKFYVE	2

FRG1	1
ASAH1	2
PCM1	2
LOC106828206	1
MTUS1	1
SLC7A2	2
ZDHHC2	2
MICU3	2
DEF8	2
TUBB3	1
TCF25	1
VPS9D1	2
CDK10	1
CHMP1A	1
RPL13	2
ANKRD11	2
TRAPPC2L	1
MVD	2
LOC106828257	2
DUSP11	1
TPRKB	1
ALMS1	1
EGR4	2
CCT7	1
PRADC1	1
RAB11FIP5	1
SPR	1
EXOC6B	2
LOC106828299	2
DYSF	2
ZNF638	2
PAIP2B	1
NAGK	1
TEX261	1
FAM136A	1
PCYOX1	2
TIA1	2
PCBP1	2
GMCL1	1
ANXA4	1
AAK1	2
NFU1	1
GFPT1	1
ANTXR1	2

APLF	1
CNRIP1	1
PNO1	1
C1D	1
ETAA1	2
ACTR2	2
SERTAD2	2
AFTPH	1
LGALSL	1
PELI1	1
VPS54	2
EHBP1	1
B3GNT2	2
VBP1	1
CMC4	1
FUNDC2	1
MPP1	2
DKC1	2
KIRREL	2
ARHGEF11	2
LRRC71	1
HDGF	1
MRPL24	1
ISG20L2	1
GPATCH4	1
APOA1BP	2
MEF2D	2
TSACC	1
CCT3	1
LOC106828470	2
LMNA	2
LAMTOR2	2
UBQLN4	2
SSR2	2
SYT11	2
LOC106828483	2
MSTO1	1
DAP3	1
ASH1L	2
RUSC1	2
FDPS	2
PKLR	2
CLK2	2
SCAMP3	2

GBA	2
MTX1	1
KRTCAP2	2
DPM3	2
EFNA1	2
EFNA4	1
FLAD1	1
SHC1	2
PYGO2	1
PBXIP1	2
PMVK	2
ADAR	2
UBE2Q1	2
TDRD10	2
IL6R	2
LOC106828536	1
LOC106828537	2
MICU2	1
ZDHHC20	2
TCEAL8	2
MRPL57	2
SKA3	1
SAP18	1
N6AMT2	1
LOC106828567	1
IFT88	1
CRYL1	1
LOC106828544	1
TRIM33	1
OLFML3	2
HIPK1	2
LRIG2	2
SLC16A1	2
LOC106828585	2
MOV10	2
CAPZA1	1
DDX20	1
RAP1A	1
ATP5F1	1
PIFO	1
CEPT1	2
DRAM2	2
LOC106828613	2
RARRES3	2

LOC106828623	2
RBBP8	2
RIOK3	1
LOC106828637	1
NPC1	2
ANKRD29	2
CEP192	2
RNMT	1
NAPG	1
VAPA	1
RAB31	1
PPP4R1	1
RALBP1	2
TWSG1	2
ANKRD12	2
NDUFV2	2
BASP1	1
MYO10	1
FAM134B	1
ZNF622	2
11-Mar	1
FBXL7	2
COL22A1	2
KLK15	1
NUFIP2	2
UBR2	2
GLTSCR1L	2
CNPY3	2
PEX6	2
MEA1	1
KLHDC3	1
RRP36	1
MRPL2	1
KLC4	2
SRF	2
CUL9	2
DNPH1	2
TJAP1	2
LRRC73	1
YIPF3	2
MAD2L1BP	1
RSPH9	1
MRPS18A	1
VEGFA	2

MRPL14	1
SLC29A1	2
HSP90AB1	2
SLC35B2	2
CDC5L	1
ENPP4	2
RCAN2	2
TDRD6	1
LOC106828811	1
SDPR	2
LOC106828791	1
MYO1B	2
STAT4	1
STAT1	2
GLS	2
LOC106828798	1
NAB1	2
LOC106828802	1
INPP1	1
HIBCH	2
LOC106828809	1
LOC106828829	1
KIAA0430	2
MYH11	2
FOPNL	1
ABCC1	2
LOC106828839	1
SLF2	2
SEMA4G	2
MRPL43	1
LZTS2	2
BTRC	1
DPCD	1
FBXW4	2
NPM3	2
MGEA5	2
KCNIP2	1
LOC106828860	1
LDB1	2
PPRC1	2
NOLC1	2
LOC106828867	2
LOC106828825	2
CXCR3	2

ACRC	1
OGT	2
TAF1	2
NONO	2
ZMYM3	2
MED12	2
LOC106828881	1
ADAM18	1
LOC106828943	2
SFRP1	2
GOLGA7	1
GPAT4	1
KAT6A	2
LOC106828953	1
AP3M2	1
POLB	1
VDAC3	1
SLC20A2	2
SMIM19	2
THAP1	1
HOOK3	2
POMK	2
HGSNAT	2
INTS10	2
FAM160A2	1
LOC106828974	1
LOC106829009	1
PHC2	1
ZNF362	2
TRIM62	2
AZIN2	1
AK2	1
RNF19B	1
TMEM54	1
YARS	1
KIAA1522	2
BSDC1	1
TSSK3	1
FAM229A	1
MARCKSL1	2
LCK	2
EIF3I	1
IQCC	1
KHDRBS1	2

PTP4A2	2
TINAGL1	2
LOC106829072	2
FABP3	2
ZCCHC17	1
SNRNP40	1
PUM1	2
LAPTM5	2
LOC106829021	1
KDM8	2
NSMCE1	1
IL4R	2
GTF3C1	2
LAT	2
SPNS1	2
NFATC2IP	1
CD19	1
RABEP2	2
ATXN2L	1
LOC106829110	1
ZHX1	2
TBC1D31	1
DERL1	1
ZHX2	2
PHF14	2
LOC106829135	1
THSD7A	2
TMEM106B	2
LOC106829126	1
CEP162	1
NT5E	2
SNX14	2
SYNCRIP	2
LOC106829134	2
LOC106829137	1
LOC106829139	2
USP38	2
GAB1	2
SMARCA5	2
ANAPC10	1
ABCE1	1
SMAD1	1
ZNF827	2
LSM6	2

TTC29	1
ARHGAP10	2
NR3C2	2
LOC106829186	2
KIAA0355	2
GPI	1
PDCD2L	1
UBA2	1
WTIP	2
LOC106829208	2
LOC106829286	1
LOC106829214	1
LOC106829212	1
ZNF30	2
LGI4	2
FXYD1	2
FXYD5	2
LOC106829228	1
LSR	2
USF2	2
GAPDHS	1
LOC106829289	1
LOC106829293	1
LOC106829247	2
KMT2B	2
U2AF1L4	1
PSENEN	1
LIN37	1
HSPB6	1
HCST	2
TYROBP	2
SDHAF1	1
CLIP3	2
LOC106829269	1
WDR62	1
TBCB	1
CAPNS1	1
LOC106829276	2
ZNF146	2
DNAJC19	2
FXR1	1
CCDC39	1
TTC14	2
USP13	2

NDUFB5	2
MRPL47	1
ACTL6A	2
GNB4	2
PIK3CA	2
ZMAT3	2
TBL1XR1	2
LOC106829327	2
VGLL4	2
TAMM41	1
TSEN2	1
MKRN2	1
RAF1	2
TMEM40	2
CAND2	2
RPL32	2
MBD4	2
RBSN	1
MRPS25	1
NR2C2	2
LOC106829360	2
CCDC174	1
SLC6A6	2
LSM3	1
XPC	2
TMEM43	2
HDAC11	1
NUP210	2
LOC106829337	1
SYCE1	1
UBAP2	2
LOC106829395	1
ST6GAL2	1
CPA6	1
ARFGEF1	2
CSPP1	1
COPS5	1
PPP1R42	1
LOC106829407	1
SGK3	1
VCPIP1	1
MYBL1	1
LOC106829419	1
PLSCR4	2

GYG1	1
HPS3	1
CP	2
TM4SF1	2
TM4SF4	1
WWTR1	2
LOC106829449	2
TSC22D2	2
SERP1	1
EIF2A	1
LOC106829426	2
SIAH2	1
LEMD3	2
LLPH	1
TMBIM4	1
ZBTB20	2
GRAMD1C	1
SPICE1	1
CFAP44	1
GTPBP8	1
CCDC80	2
SLC35A5	1
ATG3	2
KIAA1210	1
SNCA	1
SMARCAD1	2
LOC106829537	2
MARK1	2
RAB3GAP2	2
IARS2	2
MIA3	2
AIDA	1
BROX	1
SUSD4	2
CCDC185	1
CAPN2	1
TP53BP2	2
FKBP9	2
NT5C3A	1
RP9	2
BBS9	2
DPY19L1	2
DPY19L2	1
HERPUD2	1

ATP6V1H	1
TCEA1	1
LYPLA1	1
LOC106829594	2
LOC106829589	2
NCAPG	1
DCAF16	1
FAM184B	1
MED28	1
LAP3	2
TAPT1	1
ST8SIA4	2
ARHGEF10	2
PACRGL	1
ADGRA3	2
LOC106829631	1
DNAJC15	1
ENOX1	1
LACC1	2
TSC22D1	1
GPALPP1	2
TPT1	2
SLC25A30	1
SPERT	1
ZC3H13	2
LCP1	2
SUCLA2	1
NUDT15	1
MED4	1
ITM2B	2
RB1	2
RCBTB2	2
CDADC1	1
CAB39L	1
SETDB2	2
TRIO	2
FAM105A	2
ANKH	2
LOC106829722	1
FAXC	1
LOC106829738	2
PNISR	2
LOC106829744	2
MPP6	1

STK31	1
WASF1	1
FIG4	1
SMPD2	1
PPIL6	1
LOC106829760	2
CEP57L1	1
SESN1	1
FOXO3	2
LACE1	1
SNX3	1
OSTM1	1
CFI	2
PLA2G12A	1
CCDC109B	1
OSTC	1
RPL34	2
TBCK	1
NPNT	2
LOC106829829	2
CPNE3	2
RMDN1	1
WWP1	1
CA2	1
LRRCC1	1
AASS	2
RNF133	1
NDUFA5	1
WASL	2
LOC106829838	1
SPAM1	1
LOC106829873	1
GPR37	2
JADE1	1
PGRMC2	2
LARP1B	1
PLK4	1
HSPA4L	1
THOC2	2
XIAP	2
STAG2	2
SH2D1A	2
PKNOX1	1
NDUFV3	1

WDR4	1
RSPH1	1
ABCG1	2
ZBTB21	2
BACE2	2
PCP4	2
LCA5L	1
WRB	1
HMGN1	1
FANCL	2
PAPOLG	2
REL	2
PUS10	2
LOC106829971	1
PSPC1	2
LOC106829976	2
PARP4	2
CENPJ	1
CREB3L2	2
PTN	2
MTPN	1
LOC106829994	1
NUP205	2
CNOT4	1
LOC106829988	1
CALD1	2
BPGM	1
AKR1B1	1
LOC106830013	1
PDLIM5	2
IFIT5	2
PANK1	1
LOC106830028	1
KIF20B	1
ANKRD1	2
PCGF5	1
PPP1R3C	2
TNKS2	2
ADAM10	2
ALDH1A2	1
MYZAP	2
CGNL1	2
TCF12	2
CD59	1

LOC106830059	1
HIPK3	2
DCTD	2
WWC2	2
CDKN2AIP	2
ING2	2
RWDD4	2
TRAPPC11	1
STOX2	2
IRF2	2
CASP3	1
CENPU	1
ACSL1	2
SLC25A4	1
CFAP97	1
LOC106830094	1
SNX25	1
ANKRD37	1
UFSP2	1
RTTN	2
LOC106830213	1
UBQLNL	1
LOC106830220	2
LOC106830223	2
ABCA5	2
SOX9	2
SLC39A11	1
SSTR2	2
COG1	2
FAM104A	1
LOC106830246	1
SDK2	1
RPL38	2
KIF19	2
GPRC5C	2
SLC9A3R1	2
GRIN2C	1
FDXR	2
FADS6	2
HID1	2
ATP5H	1
HN1	1
SUMO2	2
NUP85	2

GGA3	2
MRPS7	1
MIF4GD	1
TMEM94	2
CASKIN2	2
RECQL5	2
H3F3B	2
WBP2	1
TRIM47	2
TRIM65	2
MRPL38	2
ACOX1	2
TEN1	1
EXOC7	1
RNF157	2
PRPSAP1	2
SPHK1	2
UBE2O	2
ST6GALNAC2	1
LOC106830339	1
MXRA7	2
JMJD6	2
METTL23	2
SRSF2	2
MFSD11	1
LOC106830346	2
SEC14L1	1
SEPT9	2
LOC106830424	2
NUDCD1	1
ENY2	2
EBAG9	1
SYBU	1
LOC106830444	1
LOC106830427	2
MARC1	1
LOC106830453	2
TIFA	2
SMC2	1
ABCA1	2
ZMYM1	2
SFPQ	2
ZMYM4	2
KIAA0319L	2

NCDN	2
PSMB2	1
LOC106830530	2
AGO3	2
TEKT2	1
TRAPPC3	1
MAP7D1	2
THRAP3	2
SH3D21	1
EVA1B	2
STK40	1
LSM10	1
OSCP1	1
MRPS15	1
ZC3H12A	2
GNL2	1
YRDC	1
SF3A3	1
RRAGC	1
MYCBP	1
RHBDL2	1
AKIRIN1	1
NDUFS5	1
MACF1	2
OXCT2	1
PPIE	1
LETM1	1
TMEM129	1
UVSSA	1
MAEA	2
SPON2	2
MICU1	1
DNAJB12	1
ANAPC16	1
PSAP	2
LOC106830630	2
LOC106830632	2
UNC5B	2
PCBD1	1
SGPL1	2
PRF1	2
EIF4EBP2	2
PPA1	1
LOC106830662	1

TMEM89	1
IP6K2	1
PRKAR2A	1
ARIH2OS	1
ARIH2	1
P4HTM	2
WDR6	2
DALRD3	1
NDUFAF3	1
IMPDH2	2
QARS	1
USP19	2
LAMB2	2
KLHDC8B	2
LOC106830758	1
LOC106830685	1
GPX1	1
RHOA	2
TCTA	1
DAG1	2
BSN	2
RNF123	2
GMPPB	1
IP6K1	2
FAM212A	1
MON1A	1
RBM5	2
GNAI2	2
SEMA3B	2
IFRD2	1
LOC106830729	2
TUSC2	1
RASSF1	2
ZMYND10	1
TMEM115	2
CISH	2
DOCK3	2
RBM15B	2
VPRBP	2
RAD54L2	2
TEX264	2
LOC106830750	1
IQCF3	1
LOC106830658	1

LOC106830763	1
LOC106830764	1
LOC106830751	1
PARP3	2
RC3H2	2
ZBTB6	1
RABGAP1	2
STRBP	1
LHX2	1
PSMB7	1
NR5A1	2
RPL35	2
ARPC5L	1
GOLGA1	1
PPP6C	1
RABEPK	1
HSPA5	2
GAPVD1	2
MAPKAP1	1
PBX3	2
DNAJB9	1
THAP5	1
ELMO1	2
DNAJC17	2
GCHFR	1
LOC106830883	1
BAHD1	1
DDX3X	2
DHX38	2
IST1	2
ZNF821	1
ATXN1L	1
PHLPP2	2
LOC106830916	2
MKLN1	2
PODXL	2
CHCHD3	1
LOC106830923	1
TTLL5	1
TGFB3	2
IFT43	1
ANGEL1	1
LRRC74A	1
IRF2BPL	2

POMT2	1
GSTZ1	1
TMED8	2
SAMD15	1
AZI2	1
ZCWPW2	1
RBMS3	2
TGFBR2	2
STT3B	1
CFB	2
NELFE	1
DXO	2
LOC106831006	2
ATF6B	2
FKBPL	1
EGFL8	2
AGPAT1	2
RNF5	2
HIBADH	2
JAZF1	1
CREB5	2
FKBP14	2
ZNRF2	1
NOD1	2
GGCT	1
GARS	2
LSM5	2
KBTBD2	2
TSTD3	1
CCNC	2
ASCC3	2
LOC106831099	1
LOC106831100	2
HSPA6	2
DUSP12	1
OLFML2B	2
TMCO1	2
ALDH9A1	1
MGST3	2
PBX1	2
NUF2	1
LOC106831138	1
LOC106831142	2
LOC106831143	2

LOC106831147	1
PPIP5K2	2
PAM	2
VEZT	2
NR2C1	2
NDUFA12	1
TMCC3	1
CEP83	1
PLXNC1	2
SOCS2	1
MRPL42	1
UBE2N	1
NUDT4	2
BTG1	2
DCN	2
CCER1	1
LPL	2
ATP6V1B2	2
LOC106831209	1
ZBTB7C	2
SMAD2	2
IER3IP1	1
ATP5C1	1
SFMBT2	1
PRKCQ	1
PSMA2	1
LOC106831256	1
ULK3	2
SCAMP2	2
MPI	1
FAM219B	2
LOC106831275	1
SCAMP5	2
PPCDC	1
LOC106831279	2
COMMD4	1
SIN3A	2
SNUPN	1
IMP3	1
LINGO1	2
HMG20A	2
PEAK1	2
TSPAN3	2
PSTPIP1	2

RCN2	2
SCAPER	2
CCDC179	1
SVIP	1
ZNF25	2
BMS1	1
HNRNPF	1
BMPR1A	2
ADIRF	2
GLUD1	2
FAM35A	2
GDF10	1
PTPN20	1
LRRC18	1
VSTM4	1
FAM170B	1
LOC106831391	1
ERCC6	2
DYRK1A	2
DSCR3	2
TTC3	2
PIGP	1
DOPEY2	1
LOC106831423	1
SETD4	2
TMEM18	1
ACP1	1
SH3YL1	1
FAM92A1	1
RBM12B	2
KIAA1429	2
DPY19L4	2
TP53INP1	2
NDUFAF6	1
PLEKHF2	1
AP3S1	1
ATG12	1
FEM1C	1
LTBR	2
CD9	2
NDUFA9	1
AKAP3	1
RAD51AP1	1
CCND2	2

PARP11	1
LOC106831509	2
ITFG2	1
FKBP4	1
SLC6A13	1
KDM5A	2
CCDC77	1
WNK1	2
RAD52	2
LOC106831444	2
EFNB2	2
ARGLU1	2
FAM155A	1
LOC106831541	1
PTPN18	1
CCDC115	1
TGFBR3	2
HFM1	1
ZNF644	2
LOC106831586	1
ZNF326	2
LRRC8D	2
LRRC8C	2
LRRC8B	1
LOC106831577	2
LOC106831580	2
CCBL2	1
PKN2	2
TRIM21	2
RRM1	2
STIM1	2
PGAP2	1
ART5	1
RNF121	1
NUMA1	2
IL18BP	2
LOC106831652	1
LAMTOR1	1
ANAPC15	1
LOC106831658	2
INPPL1	2
CLPB	1
LOC106831662	2
STARD10	1

ATG16L2	2
P2RY2	2
FAM168A	2
PLEKHB1	2
MRPL48	2
LOC106831678	1
C2CD3	1
PPME1	1
LIPT2	1
FBXO8	1
LOC106831737	1
SAP30	1
HMGB2	2
KAT2B	2
RAB5A	1
EFHB	1
SATB1	2
TBC1D5	2
DAZL	2
DPH3	1
LOC106831784	1
LOC106831787	1
CYLC2	1
PPP3R2	1
ZNF189	1
MRPL50	1
PLPPR1	1
LOC106831797	2
TEX10	1
STX17	2
NR4A3	2
CDH2	2
DSC3	2
DSC2	2
DSC1	2
ADGRB3	2
LMBRD1	2
LOC106831846	1
SBDS	1
TMEM248	2
RABGEF1	2
TPST1	2
CRCP	1
ASL	2

GUSB	2
NUPR2	1
CHCHD2	1
CCT6A	2
PSPH	1
GBAS	2
MRPS17	1
SEPT14	1
LOC106831868	2
TGFB1I1	1
PYCARD	2
FUS	2
BCKDK	1
VKORC1	2
ZNF646	1
STX4	2
HSD3B7	2
SETD1A	2
ORAI3	1
LOC106831839	2
BCL7C	2
RNF40	1
PHKG2	1
SRCAP	2
PRR14	2
ZNF689	2
LOC106831915	1
ITGAL	2
SEPHS2	2
ZNF771	1
HSD17B11	2
NUDT9	1
SPARCL1	2
SPP1	2
PKD2	2
PPM1K	2
HERC5	1
PYURF	1
TIGD2	2
NR2F2	2
CHD2	2
FAM174B	1
SLCO3A1	2
AKAP13	1

MRPL46	1
MRPS11	1
ISG20	2
MFGE8	2
ABHD2	1
FANCI	2
KIF7	2
PEX11A	1
MESP1	2
ANPEP	2
LOC106832031	1
ARPIN	2
IDH2	1
LOC106832036	1
SEMA4B	2
CIB1	1
LOC106832040	1
VPS33B	1
PRC1	1
HDDC3	2
FES	2
BLM	1
CRTC3	2
LOC106832054	2
WDR73	1
NMB	2
SEC11A	2
ZNF592	2
ALPK3	2
PDE8A	2
RPS17	2
CPEB1	2
WHAMM	2
HOMER2	2
FAM103A1	1
LOC106831990	1
BTBD1	1
LOC106831991	1
SH3GL3	1
ABHD17C	1
MESDC1	2
EEF1E1	1
BLOC1S5	1
LOC106832087	2

TXNDC5	2
SNRNP48	2
DSP	2
CAGE1	1
SSR1	2
RREB1	2
LOC106832121	1
MED7	1
THG1L	1
CLINT1	2
UBLCP1	1
TTC1	1
PWWP2A	2
FABP6	1
CCNJL	1
PTTG1	1
CCNG1	1
NUDCD2	1
MAT2B	2
COPS8	1
COL6A3	2
RAB17	1
LRRFIP1	2
UBE2F	1
ILKAP	2
TRAF3IP1	1
ASB1	1
NDUFA10	1
MYEOV2	1
GPC1	2
RNPEPL1	2
CAPN10	1
KIF1A	1
PPP1R7	1
HDLBP	1
STEAP2	2
CFAP69	1
GTPBP10	1
CLDN12	2
AKAP9	2
LOC106832249	2
LRRD1	1
ANKIB1	2
LOC106832253	1

GATAD1	2
PEX1	2
RBM48	1
LOC106832256	1
FAM133B	2
SAMD9	2
SAMD9L	2
CTNNA2	1
ASB3	2
ERLEC1	1
GPR75	2
PSME4	1
ACYP2	2
LOC106832289	1
SPTBN1	2
RTN4	2
RPS27A	2
CCDC88A	1
CFAP36	1
PPP4R3B	2
EFEMP1	2
CCDC85A	2
LOC106832347	2
NFIC	2
FZR1	1
LOC106832357	1
HMG20B	2
APBA3	2
DAPK3	2
EEF2	2
PIAS4	1
MPND	1
CHAF1A	1
UBXN6	1
MYDGF	1
PLIN3	1
ARRDC5	1
ZNRF4	1
SAFB2	1
SAFB	2
LOC106832396	1
LOC106832398	2
LOC106832399	2
URI1	1

CCNE1	1
TNNI1	1
IPO9	2
SHISA4	2
LMOD1	2
TIMM17A	1
RNPEP	2
GPR37L1	2
UBE2T	1
PCLO	2
RNF6	1
NUP58	1
MTMR6	1
SPATA13	2
MIPEP	1
SACS	2
GPR63	2
FHL5	1
UFL1	1
MANEA	1
ACSL3	1
FARSB	1
EPHA4	2
JAKMIP1	1
TRIM35	2
CLU	2
CCDC25	1
PBK	1
ELP3	1
LOC106832584	2
ZNF395	2
EXTL3	2
EIF4A2	2
RFC4	1
BCL6	2
LPP	1
CLDN1	2
CCDC50	2
MB21D2	2
HRASLS	1
ATP13A4	2
OPA1	1
HES1	2
CPN2	2

ATP13A3	2
VRK1	2
PAPOLA	2
AK7	1
GSKIP	1
ATG2B	2
SYNE3	1
CLMN	1
DICER1	2
PSIP1	1
TTC39B	1
ZDHHC21	2
NFIB	2
MPDZ	2
CIB4	1
LOC106832678	1
DRC1	1
HADHB	2
MRPS9	1
TGFBRAP1	2
FHL2	1
LOC106832696	2
UXS1	2
TAB3	2
XRCC5	1
MREG	1
LOC106832724	1
NDUFA11	1
ACSBG2	1
CLPP	2
GTF2F1	1
KHSRP	2
C3	2
SORL1	2
LOC106832769	2
HSPA8	2
TMEM225	1
RUNX1	1
RCAN1	1
KCNE1	2
LOC106832850	1
SMIM11A	2
MRPS6	1
SLC5A3	2

ATP5O	1
ITSN1	2
CRYZL1	1
DONSON	1
SON	2
DNAJC28	1
TMEM50B	1
IFNGR2	1
IFNAR1	2
IL10RB	2
KCNT2	1
LOC106832852	2
LOC106832862	2
LOC106832864	1
LOC106832865	2
TIMM21	1
KDSR	2
BCL2	2
ZCCHC2	1
KIAA1468	2
WASF3	2
RPL21	2
RASL11A	2
GTF3A	1
MTIF3	1
POLR1D	2
LOC106832904	1
PAN3	2
POMP	1
UBL3	2
ZNF697	2
HSD3B2	2
IKZF4	2
RPS26	2
ZC3H10	2
ESYT1	2
MYL6B	2
MYL6	2
SMARCC2	2
CS	2
STAT2	2
TIMELESS	2
RBMS2	2
BAZ2A	2

PTGES3	2
NACA	1
PRIM1	1
TAC3	2
LRP1	2
SHMT2	1
NDUFA4L2	2
R3HDM2	2
MARS	1
DDIT3	2
MBD6	2
DCTN2	1
DTX3	2
B4GALNT1	1
OS9	2
TSPAN31	2
TSFM	1
CTDSP2	1
LOC106833028	1
LRIG3	2
TNFAIP8	2
PRR16	2
FBXO33	2
PNN	2
TRAPPC6B	1
GEMIN2	1
LOC106833074	1
COPB1	2
SPON1	2
FAR1	1
BTBD10	1
RASSF10	2
TEAD1	2
PARVA	2
DKK3	2
USP47	2
GALNT18	1
ZBED5	2
EIF4G2	2
CTR9	1
RNF141	1
ADM	2
ZNF143	2
TMEM41B	2

DENND5A	2
NRIP3	2
LOC106833113	1
AKIP1	1
ST5	2
RPL27A	2
STK33	1
EIF3F	2
MBP	2
ZNF236	2
LOC106833149	2
ZNF516	1
TSHZ1	2
ZADH2	2
KTI12	1
ZFYVE9	2
CC2D1B	2
ZCCHC11	2
GPX7	2
FAM159A	2
LOC106833177	1
ECHDC2	1
LOC106833187	2
LRP8	2
LOC106833192	1
LRRC42	2
TMEM59	2
MRPL37	1
MROH7	1
DHCR24	2
LOC106833228	2
ARHGAP20	1
FDX1	1
RDX	2
UBE3A	2
SNRPN	1
SNURF	1
LOC106833253	1
SALL2	2
TOX4	1
RAB2B	1
CHD8	2
SUPT16H	2
RPGRIP1	1

HNRNPC	2
ZNF219	2
ARHGEF40	2
TPPP2	1
NDRG2	2
RNASE1	2
LOC106833279	2
RNASE12	2
LOC106833283	1
LOC106833287	1
TMEM55B	1
APEX1	1
OSGEP	1
TTC5	1
LOC106833444	2
GNAI1	1
SEMA3C	2
LOC106833456	1
HOOK1	1
JUN	2
MYSM1	2
LOC106833467	1
OMA1	2
LOC106833474	2
PRKAA2	2
PLPP3	2
ALDH3B2	1
NUDT8	1
NDUFV1	1
LOC106833486	1
CDK2AP2	1
TMEM134	1
RPS6KB2	1
CLCF1	2
RHOD	2
RBM4B	1
LOC106833517	1
CCS	2
CTSF	1
ZDHHC24	1
DPP3	1
MRPL11	1
B4GAT1	2
BRMS1	1

YIF1A	1
KLC2	1
CST6	2
BANF1	2
EIF1AD	1
SART1	2
TSGA10IP	1
LOC106833551	2
FOSL1	2
CCDC85B	1
FIBP	1
CTSW	2
EFEMP2	2
MUS81	1
LOC106833561	1
AP5B1	1
RNASEH2C	1
SIPA1	2
PCNXL3	2
FAM89B	1
SSSCA1	1
LTBP3	2
LOC106833580	2
DPF2	2
CDC42EP2	2
POLA2	2
LOC106833596	1
SYVN1	2
FAU	2
ZNHIT2	1
TM7SF2	2
VPS51	1
TMEM262	1
ZFPL1	1
NAALADL1	2
SAC3D1	1
SNX15	1
ARL2	1
BATF2	2
EHD1	1
SF1	1
PYGM	1
PRDX5	1
TRMT112	2

TEX40	1
GPR137	1
BAD	1
PPP1R14B	1
FKBP2	2
VEGFB	2
DNAJC4	1
NUDT22	1
FERMT3	2
STIP1	2
OTUB1	1
LOC106833641	2
NAA40	2
MARK2	2
LOC106833648	2
RTN3	2
ATL3	2
NSFL1C	1
LOC106833691	1
PSMF1	1
SLC52A3	2
LOC106833752	2
CSNK2A1	1
TBC1D20	1
RBCK1	2
ZCCHC3	2
DEFB119	1
LOC106833706	1
DEFB123	1
REM1	1
ID1	2
BCL2L1	1
FOXS1	2
DUSP15	1
TTLL9	1
PDRG1	1
PLAGL2	1
ASXL1	2
COMMD7	2
MAPRE1	1
SUN5	1
NET1	2
ASB13	1
FAM208B	1

GDI2	1
FBXO18	2
RBM17	1
BLZF1	1
CCDC181	1
KIFAP3	1
PRRX1	2
PRRC2C	2
VAMP4	1
LOC106833843	2
LOC106833889	2
LOC106833850	1
GSTK1	2
SPATA6	1
LOC106833860	2
CMPK1	1
GLUL	1
IER5	2
STX6	2
KIAA1614	2
XPR1	2
ACBD6	1
CEP350	1
TOR1AIP1	2
LOC106833921	2
LOC106833923	2
AXDND1	1
SOAT1	2
ABL2	1
TOR3A	1
FAM20B	1
RALGPS2	2
LOC106833935	1
TEX35	1
LINC00083	1
RASAL2	1
LOC106833942	2
LOC106833949	1
LOC106833953	2
TNN	2
MRPS14	1
CACYBP	1
DSG2	2
B4GALT6	2

TRAPPC8	2
RNF138	1
CCDC178	1
AGA	1
DPYSL2	2
BNIP3L	2
PPP2R2A	2
ST3GAL4	1
FOXRED1	1
SRPRA	2
FAM118B	1
CDON	2
DDX25	1
HYLS1	1
LOC106834033	1
TJP1	2
NSMCE3	2
APBA2	2
MCEE	1
MPHOSPH10	1
FAN1	2
MTMR10	2
MAGEL2	2
NDN	2
ANXA5	2
EXOSC9	1
BBS7	1
TRPC3	2
KIAA1109	2
ADAD1	1
LOC106834080	1
SPATA5	1
LOC106834085	2
IWS1	2
MAP3K2	2
ERCC3	2
BIN1	2
LOC106834143	1
YBX3	1
SMIM10L1	1
LOC106834115	1
ETV6	2
BCL2L14	1
LRP6	2

LOC106834152	1
THOP1	2
SGTA	1
SLC39A3	1
GADD45B	2
LMNB2	1
TIMM13	1
LSM7	2
JSRP1	2
AMH	2
PLEKHJ1	1
DOT1L	2
IZUMO4	1
MOB3A	1
MKNK2	2
LOC106834170	1
REXO1	1
ATP8B3	1
TCF3	2
LOC106834176	1
LOC106834177	2
MBD3	2
REEP6	1
LOC106834182	1
RPS15	2
DAZAP1	1
GAMT	1
NDUFS7	1
LOC106834187	1
LOC106834225	1
LOC106834190	2
MIDN	1
ATP5D	1
STK11	1
GPX4	1
POLR2E	1
ABCA7	2
WDR18	2
CFD	2
PTBP1	2
RNF126	1
BSG	1
CDC34	1
TPGS1	1

ODF3L2	1
ACAA1	1
PLCD1	1
GOLGA4	1
LRRFIP2	1
EPM2AIP1	2
TRANK1	2
FAM135A	2
SMAP1	1
SPATA7	1
PTPN21	1
ZC3H14	1
FOXN3	2
GGH	2
YTHDF3	2
MTFR1	1
PDE7A	1
DNAJC5B	1
LOC106834325	1
PKIG	1
SERINC3	2
FITM2	2
OSER1	1
IFT52	1
SRSF6	2
CHD6	2
EMILIN3	2
ZHX3	1
PLCG1	2
TOP1	2
CCDC150	1
SF3B1	2
COQ10B	2
HSPD1	2
LOC106834369	2
BOLL	1
LOC106834376	1
TYW5	1
TJP2	2
PTAR1	2
SMC5	1
PLXDC2	2
ARL5B	2
MRC1	1

HACD1	1
VIM	2
TRDMT1	2
LOC106834439	1
DNAJB14	2
LAMTOR3	1
TRMT10A	1
ALDH1L1	1
TXNRD3	1
CHCHD6	1
PLXNA1	2
TPRA1	2
MCM2	2
PODXL2	2
ABTB1	2
RUVBL1	1
EEFSEC	1
RPN1	2
TRA2B	2
SENP2	1
LOC106834503	1
CLCN2	2
EIF4G1	2
PSMD2	1
ABCF3	1
DVL3	1
ABCC5	2
YEATS2	2
KLHL24	2
MCF2L2	2
MCCC1	2
DCUN1D1	1
TULP1	1
FANCE	2
TCP11	1
ANKS1A	1
TAF11	1
UHRF1BP1	2
SNRPC	1
LOC106834573	1
NUDT3	2
LOC106834578	1
GRM4	2
LOC106834583	1

ITPR3	2
LOC106834585	1
LOC106834587	1
ZBTB9	1
CUTA	2
DAXX	1
TAPBP	2
RGL2	2
PFDN6	1
WDR46	1
RPS18	2
VPS52	2
RING1	2
HSD17B8	2
SLC39A7	2
RXRB	2
LOC106834638	2
BRD2	2
LOC106834611	2
PSMB9	2
TAP1	2
PSMB8	2
TAP2	2
LOC106834619	2
MGMT	2
LOC106834644	1
GLRX3	1
JADE2	2
CDKN2AIPNL	2
UBE2B	1
CDKL3	1
PPP2CA	2
SKP1	1
TCF7	2
VDAC1	1
PDCD10	1
SERPINI1	2
ACTRT3	1
MYNN	2
PHC3	2
RYR2	2
MTR	2
ACTN2	1
HEATR1	2

LGALS8	1
ERO1B	1
LYST	2
B3GALNT2	2
TBCE	1
ARID4B	2
RBM34	1
TOMM20	2
IRF2BP2	2
TARBP1	2
LOC106834708	1
PDK1	1
ITGA6	2
HAT1	1
SLC25A12	2
DCAF17	1
GORASP2	1
LOC106834731	1
OLA1	1
SP3	2
PDGFA	2
LOC106834749	2
PRKAR1B	1
SUN1	2
GET4	1
ADAP1	1
LOC106834763	2
GPR146	2
ZFAND2A	2
INTS1	2
MAFK	2
TMEM184A	2
PSMG3	2
FTSJ2	1
NUDT1	1
SNX8	1
LFNG	2
IQCE	1
BRAT1	2
SEL1L	2
GTF2A1	1
CEP128	1
SLIRP	1
ISM2	2

AHSA1	1
PUM3	1
VLDLR	2
SMARCA2	2
LOC106834845	1
FAM122A	1
FAM200A	1
ZKSCAN5	2
ZNF789	2
TRIM4	2
RASSF8	1
BHLHE41	2
SSPN	2
ITPR2	2
ASUN	1
TM7SF3	2
MED21	1
STK38L	1
SMCO2	1
PPFIBP1	2
KLHL42	2
CCDC91	1
ATOX1	1
G3BP1	2
SAP30L	1
CTDP1	1
ATP9B	1
DYNLT1	1
TMEM181	2
TULP4	1
GTF2H5	1
SNX9	2
ZDHHC14	2
TMEM242	1
LOC106834939	1
ARID1B	2
LOC106834949	2
LOC106834950	1
FATE1	1
PRRG3	2
VMA21	2
CD99L2	2
MTM1	1
LOC106834963	2

LOC106834964	2
LOC106834965	1
POP4	1
LOC106834971	1
BLOC1S6	1
SLC30A4	1
LOC106834987	1
GATM	1
SHF	1
DUOXA2	1
SORD	1
LOC106835000	1
TRIM69	2
B2M	2
SPG11	2
CASC4	2
WDR76	1
MFAP1	1
HYPK	2
SERF2	1
PDIA3	2
MAP1A	2
TP53BP1	2
LOC106835030	2
LOC106835086	1
MYL9	2
LOC106835046	1
SAMHD1	2
RPN2	2
SRC	2
BLCAP	1
NNAT	2
CTNNBL1	1
LOC106835073	1
ADIG	1
DHX35	2
CABS1	1
JCHAIN	1
RUFY3	1
GRSF1	2
MOB1B	1
LOC106835105	1
ANKRD17	2
ALB	1

MRPL19	1
EVA1A	1
POLE4	2
HK2	1
NOP10	1
EMC4	1
EMC7	1
AVEN	1
SCG5	2
AQR	2
DPH6	2
TMCO5A	1
SPRED1	2
THBS1	2
EIF2AK4	2
SRP14	1
KNSTRN	1
IVD	1
METTL7A	1
SLC11A2	2
CSRNP2	2
TFCP2	2
BIN2	1
ACVR1B	2
NR4A1	2
KRT7	2
KRT78	2
KRT8	2
LOC106835271	2
KRT18	2
EIF4B	2
SPRYD3	1
MFSD5	1
PFDN5	1
LOC106835285	1
AAAS	1
SP1	1
AMHR2	2
PRR13	1
PCBP2	2
MAP3K12	2
TARBP2	2
ATF7	2
CITED1	1

RPS4X	2
PIN4	1
RGAG4	2
RARRES1	1
GFM1	2
LOC106835335	2
LOC106835338	2
LOC106835340	2
SSR3	2
TIPARP	2
CEP112	1
AMZ2	1
WIPI1	1
LOC106835366	2
LAMA2	2
ARHGAP18	2
L3MBTL3	2
MED23	2
MAK16	1
LOC106835419	1
MTERF3	1
PTDSS1	2
SDC2	2
MTDH	2
RPL30	2
HRSP12	1
NIPAL2	1
LOC106835441	2
TRIM27	2
ZNF311	2
LOC106835457	2
SDCCAG8	1
CEP170	1
TUSC3	1
PLEKHA7	2
RPS13	2
PIK3C2A	2
NUCB2	2
NCR3LG1	2
SAAL1	1
LOC106835497	1
PHKA2	1
SH3KBP1	2
EIF1AX	2

RPS6KA3	2
MBTPS2	2
SMS	2
TANC2	2
ACE	1
DCAF7	1
MAP3K3	2
LIMD2	1
STRADA	1
CCDC47	2
DDX42	2
FTSJ3	1
PSMC5	1
LOC106835542	2
TEX2	1
DDX5	2
CEP95	1
SMURF2	2
KPNA2	1
LOC106835556	2
BPTF	2
PITPNC1	1
LOC106835559	2
HELZ	2
LOC106835635	2
RHOB	2
PUM2	2
LAPTM4A	2
TTC32	1
LOC106835586	1
RDH14	1
KCNS3	1
LOC106835647	1
MYCN	2
DDX1	2
TRIB2	2
LOC106835604	1
MTMR14	1
BRPF1	2
OGG1	1
TADA3	1
LOC106835611	1
JAGN1	1
EMC3	1

FANCD2OS	1
BRK1	1
VHL	2
IRAK2	2
SEC13	1
FEM1B	1
SPESP1	1
GLCE	2
PAQR5	1
KIF23	1
RPLP1	2
UACA	2
LRRC49	1
NRAS	1
SYCP1	1
VANGL1	2
ATP1A1	2
CD58	1
IGSF3	2
CD2	2
PTGFRN	2
TTF2	2
TRIM45	1
MAN1A2	2
FAM46C	1
LOC106835713	2
LOC106835803	2
LOC106835804	1
CFAP45	1
TAGLN2	1
IGSF8	2
ATP1A2	2
DCAF8	1
PEX19	1
COPA	2
NCSTN	2
SLAMF1	2
CD48	2
LOC106835778	1
AKIRIN2	1
RARS2	1
LOC106835845	1
ZNF292	2
CCDC127	1

SDHA	2
PDCD6	1
EXOC3	1
CEP72	1
TPPP	1
BRD9	2
SLC12A7	2
CLPTM1L	1
NDUFS6	1
CWC27	2
SREK1IP1	1
RNF180	2
IPO11	1
LOC106835936	2
SIAE	1
SPA17	1
MSANTD2	2
CCDC15	1
SLC37A2	1
TMEM218	1
FEZ1	1
EI24	2
ACRV1	1
LOC106835965	1
LOC106835968	2
LOC106835969	2
LOC106836129	1
LOC106836124	2
LOC106836089	2
LOC106836144	1
LOC106836135	2
LOC106836126	2
LOC106836130	2
LOC106836112	2
ZNF569	1
LOC106836054	1
SPINT2	1
LOC106836060	2
CATSPERG	1
PSMD8	1
GGN	1
LOC106836066	1
EIF3K	2
LGALS4	1

HNRNPL	2
MRPS12	1
SNRNP25	1
RHBDF1	2
HBA1	2
HBQ1	2
LUC7L	1
FAM234A	2
MRPL28	1
TMEM8A	1
NME4	1
RAB11FIP3	2
FAM195A	1
RHOT2	2
STUB1	1
METRN	2
FAM173A	2
NARFL	1
MSLN	2
LMF1	1
SOX8	2
LOC106836194	1
CACNA1H	2
LOC106836202	1
CLCN7	2
TELO2	2
SPSB3	1
NUBP2	1
HAGH	1
MSRB1	1
NDUFB10	1
RNF151	1
TBL3	1
GFER	1
SLC9A3R2	1
TSC2	2
PKD1	2
LOC106836284	2
TRAF7	2
PGP	1
E4F1	2
ECI1	1
RNPS1	2
ABCA3	2

LOC106836250	1
LOC106836255	2
LOC106836256	1
PCMTD2	2
LKAAEAR1	1
TCEA2	1
LOC106836294	2
ZNF512B	2
UCKL1	1
TPD52L2	1
ABHD16B	1
ZBTB46	2
SLC2A4RG	2
LIME1	2
ZGPAT	1
ARFRP1	1
TNFRSF6B	2
GMEB2	2
LOC106836313	1
PPDPF	1
ARFGAP1	1
GID8	1
TCFL5	1
OGFR	2
RPS21	2
LAMA5	2
ADRM1	1
MTG2	2
PSMA7	2
LOC106836345	1
PHACTR3	2
SYCP2	1
COL27A1	1
ZNF618	2
MCMBP	1
BAG3	2
TIAL1	2
RGS10	2
PRDX3	2
SFXN4	2
FAM45A	1
EIF3A	2
CACUL1	1
FAM204A	1

RAB11FIP2	2
PDZD8	2
SHTN1	2
LOC106836384	1
LOC106836429	1
LOC106836430	2
ARL4D	2
DHX8	2
DUSP3	1
LOC106836437	1
LSM12	1
HDAC5	2
ATXN7L3	1
UBTF	2
RUNDC3A	1
SLC25A39	1
GRN	2
GPATCH8	2
FZD2	2
MEIOC	1
GJC1	2
CCDC103	1
DCAKD	1
NMT1	1
HEXIM1	2
MAP3K14	2
LOC106836484	1
GOSR2	1
WNT3	1
NSF	1
LOC106836492	1
KANSL1	2
CDC27	1
MYL4	1
KITLG	2
TMTC3	2
CEP290	2
LOC106836518	1
LOC106836529	1
ECHDC3	1
UPF2	2
LOC106836526	2
MGAT5	2
CCNT2	2

RAB3GAP1	2
R3HDM1	1
UBXN4	1
MCM6	2
DARS	2
LOC106836548	1
CXCR4	2
MYLIP	2
JARID2	2
CD83	2
RNF182	2
MCUR1	1
RANBP9	1
NOL7	1
PHACTR1	1
MME	1
DHX36	1
RAP2B	1
MBNL1	2
TTC23	1
SYNM	2
IGF1R	2
ARRDC4	2
EPC1	2
LOC106836615	1
ITGB1	2
PARD3	2
CUL2	1
CREM	1
MYO9A	1
SENP8	1
HEXA	2
LOC106836642	1
LOC106836643	1
LOC106836644	2
ADPGK	2
NEO1	2
NPTN	2
CD276	2
TBC1D21	1
STOML1	2
DHRS3	1
VPS13D	2
MIIP	1

PLOD1	2
CLCN6	2
AGTRAP	1
MAD2L2	1
LOC106836673	1
MTOR	1
EXOSC10	1
SRM	2
TARDBP	2
LOC106836685	1
NMNAT1	1
LZIC	1
CTNNBIP1	1
CLSTN1	2
PIK3CD	2
SLC25A33	1
TGFB2	2
SPATA17	1
GPATCH2	1
KCTD3	2
RGS1	2
RGS2	1
UCHL5	1
TROVE2	1
GLRX2	1
TXNDC11	2
SNN	1
LITAF	1
LOC106836782	1
LOC106836783	1
PRM3	1
TNP2	1
SOCS1	2
DEXI	1
CIITA	2
ATF7IP2	1
LOC106836781	2
USP7	1
LOC106836785	1
CARHSP1	1
PMM2	1
TMEM186	1
ABAT	1
METTL22	1

IFRD1	2
LOC106836802	1
TMEM168	2
PRPH	1
TUBA1C	1
LOC106836806	2
LOC106836811	2
LMBR1L	1
DHH	2
RHEBL1	2
KMT2D	2
PRKAG1	2
FKBP11	1
CCDC65	1
RND1	2
LOC106836827	1
KANSL2	2
LOC106836907	1
LOC106836831	1
CCDC184	1
PFKM	1
SENP1	1
TMEM106C	1
HDAC7	2
SLC48A1	2
PCED1B	2
LOC106836897	2
RPL3	2
LOC106836910	1
TAB1	2
RALYL	2
EXOC1	1
PDCL2	1
CLOCK	1
TMEM165	2
SRD5A3	1
PIK3C3	2
CDC123	1
LOC106836936	1
OPTN	2
TAF7	2
LOC106836957	2
DIAPH1	2
FCHSD1	2

ARAP3	2
KIAA0141	2
RNF14	1
NDFIP1	2
NR3C1	2
LAMC1	2
SMG7	2
ARPC5	2
LOC106837001	2
EDEM3	2
FAM129A	2
RNF2	2
TRMT1L	2
SWT1	2
IVNS1ABP	2
DPYSL5	1
MAPRE3	1
AGBL5	1
OST4	2
EMILIN1	2
KHK	1
PREB	1
PRR30	1
ATRAID	2
TRIM54	1
GTF3C2	2
EIF2B4	2
SNX17	1
PPM1G	1
NRBP1	2
FNDC4	2
ZNF512	2
GPN1	1
SLC4A1AP	1
MRPL33	2
RBKS	1
FOSL2	2
PPP1CB	2
SPDYA	1
CLIP4	1
RHBDD2	2
HIP1	1
LOC106837126	1
NSUN5	2

TRIM50	2
FKBP6	1
BAZ1B	2
BCL7B	1
TBL2	1
MLXIPL	2
DNAJC30	1
WBSCR22	1
CLDN4	1
WBSCR27	2
WBSCR28	1
LIMK1	2
DNAJC2	1
ARMC10	2
CCDC146	1
FGL2	2
PTPN12	2
RSBN1L	2
TMOD3	1
MAPK6	1
MYO5C	2
MYO5A	1
ARPP19	1
FAM214A	2
CHCHD10	2
MMP11	2
LOC106837167	2
TCF21	2
TBPL1	1
SGK1	2
HBS1L	1
BCLAF1	2
MAP7	1
MAP3K5	2
PEX7	1
IFNGR1	2
MTHFD1L	1
LOC106837209	2
PCMT1	1
LATS1	2
KATNA1	1
GINM1	1
PPIL4	1
TAB2	2

SASH1	2
STXBP5	2
RAB32	1
SHPRH	2
EPM2A	2
STX11	2
SF3B5	1
PLAGL1	2
LTV1	1
PHACTR2	1
PEX3	1
ADAT2	2
AIG1	2
LOC106837250	1
SNRPF	2
NTN4	2
USP44	1
METAP2	1
PELI2	2
TMEM260	2
EXOC5	1
AP5M1	1
ACSS2	1
GSS	1
TRPC4AP	2
EIF6	1
LOC106837276	1
CEP250	2
ERGIC3	2
LOC106837283	2
ROMO1	2
RBM39	2
PHF20	2
SCAND1	2
EPB41L1	2
AAR2	1
BNIP1	1
CREBRF	2
ATP6V0E1	1
RPL26L1	2
ERGIC1	2
DUSP1	2
LOC106837354	1
UBTD2	1

EFCAB9	1
STK10	2
FBXW11	1
NPM1	2
RANBP17	1
KCNIP1	2
KCNMB1	2
LCP2	2
LOC106837367	1
SPDL1	1
PANK3	2
RARS	2
WWC1	2
TENM2	2
EPHA7	2
KLRK1	2
LOC106837403	2
GABARAPL1	2
CD69	2
LOC106837420	2
M6PR	1
PHC1	1
RIMKLB	1
LOC106837435	1
SLC2A3	1
FOXJ2	2
ZBTB10	2
ZNF704	2
FABP5	2
LOC106837465	1
NCKAP5	1
LOC106837477	1
TRAF6	2
COMMD9	1
FJX1	1
CD44	2
PDHX	1
APIP	2
CAT	2
CAPRIN1	1
TCP11L1	1
PTPRD	2
TMEM261	1
HPS5	2

GTF2H1	1
LDHA	1
LDHC	1
TSG101	2
LOC106837538	1
ACSL5	1
GPAM	2
SHOC2	1
BBIP1	1
PDCD4	2
LOC106837553	1
SMC3	2
DUSP5	2
LOC106837563	2
LOC106837564	2
MYH9	2
TXN2	1
EIF3D	2
IFT27	1
TEX33	1
TST	1
IL2RB	2
C1QTNF6	2
LOC106837588	1
CARD10	2
CDC42EP1	2
LGALS2	2
GGA1	1
SH3BP1	1
LOC106837599	1
LOC106837601	2
ANKRD54	1
MICALL1	1
POLR2F	2
LOC106837609	2
PICK1	1
MAFF	2
LOC106837614	2
TMEM184B	2
LOC106837615	2
KDELR3	2
DDX17	2
CBY1	1
TOMM22	1

JOSD1	2
DNAL4	1
KBTBD3	1
AASDHPPT	2
LOC106837671	1
IKZF2	2
ERBB4	2
ZNF703	2
PROSC	1
RAB11FIP1	1
LOC106837695	2
NEK3	2
ATP7B	1
CCDC70	1
INTS6	1
RNASEH2B	2
TRIM13	1
SPRYD7	1
EBPL	2
PALLD	2
LOC106837730	2
DDX60	2
CPE	2
MSMO1	2
KLHL2	1
TMA16	1
TKTL2	1
NAF1	2
SLC30A7	2
EXTL2	2
VCAM1	2
RTCA	1
LRRC39	2
SASS6	1
AGL	2
PLPPR4	2
SNX7	1
HMGN4	1
LOC106837800	2
LOC106837801	2
LOC106837809	2
LOC106837812	2
LOC106837815	2
LOC106837884	2

LOC106837830	2
LOC106837835	1
LOC106837833	2
LOC106837817	2
LOC106837827	2
LOC106837838	2
LOC106837819	2
LOC106837845	2
TRIM38	2
LRRC16A	2
LOC106837860	1
GMNN	2
LOC106837863	2
ACOT13	2
TDP2	1
NDUFA8	1
MORN5	1
LHX6	1
RBM18	1
CIR1	1
SCRN3	1
GPR155	1
WIPF1	2
CHN1	2
ATF2	2
ATP5G3	1
KIAA1715	1
LOC106837909	1
MAPK7	1
B9D1	1
EPN2	2
TOP3A	2
FLII	2
DRG2	1
GID4	1
ATPAF2	1
RAB23	2
BAG2	2
ZNF451	2
KIAA1586	2
DST	2
COL21A1	2
KIF5B	2
ARHGAP12	2

ZEB1	2
KIAA1462	2
MAP3K8	2
LOC106837978	1
COIL	1
SCPEP1	2
LOC106837981	1
AKAP1	1
CPEB2	1
BOD1L1	2
RAB28	1
CRTAP	2
GLB1	2
CNOT10	1
ARHGAP28	2
LAMA1	2
MTCL1	1
LOC106838029	2
BTF3	1
ANKRA2	1
ARHGEF28	2
TNPO1	2
LOC106838062	1
PTCD2	1
MAP1B	2
LOC106838044	2
BDP1	2
LOC106838049	1
LOC106838053	1
OCLN	2
RAD17	1
AK6	1
TAF9	1
LOC106838082	1
LOC106838137	2
SLC35G2	1
NCK1	1
MRAS	2
CEP70	1
FAIM	1
PIK3CB	2
MRPS22	1
RBP1	1
NMNAT3	1

SLC25A36	2
ZBTB38	2
RASA2	2
RNF7	1
ATP1B3	1
TFDP2	1
GK5	2
XRN1	2
U2SURP	2
LOC106838133	2
FAM133A	1
KIF13B	2
MSRA	1
LOC106838169	1
PINX1	1
TPP1	2
TAF10	1
ILK	2
RRP8	2
TIMM10B	1
APBB1	2
SMPD1	2
ABHD13	1
TNFSF13B	2
IRS2	2
COL4A1	2
COL4A2	2
RAB20	1
CARS2	1
ING1	1
ANKRD10	2
LOC106838217	1
LOC106838220	1
LIG1	1
EMP3	2
SYNGR4	1
KDELR1	2
GRWD1	1
CYTH2	1
SPACA4	1
DBP	2
CA11	1
PLEKHA4	2
PPP1R15A	2

TULP2	1
NUCB1	2
DHDH	1
FTL	2
RUVBL2	1
SNRNP70	2
LIN7B	1
DKKL1	1
PIH1D1	1
ALDH16A1	2
FLT3LG	2
RPL13A	2
RPS11	2
LOC106838298	2
DUSP21	1
FUNDC1	1
MAOA	2
TMEM263	2
MTERF2	2
TIMP3	2
LOC106838356	2
ZFAND6	2
FAH	2
ITM2A	2
LOC106838359	1
LOC106838369	1
BRWD3	2
PRSS46	1
RTP3	1
CCRL2	2
FYCO1	1
LZTFL1	1
SACM1L	2
LIMD1	2
ZDHHC3	1
TMEM42	1
KIF15	1
ZNF197	2
LOC106838413	1
LOC106838422	2
ZNF445	2
TCAIM	1
LOC106838428	1
SMARCAL1	1

RPL37A	2
IGFBP2	2
IGFBP5	2
TNP1	1
TNS1	2
ARPC2	2
AAMP	1
LOC106838446	1
TMBIM1	2
LOC106838442	1
USP37	2
RNF25	1
STK36	1
TTLL4	1
LOC106838458	2
PRKAG3	2
WNT6	2
SH3TC2	2
PCYOX1L	2
CSNK1A1	2
SLC26A2	1
TCOF1	2
CD74	2
RPS14	2
NDST1	2
SYNPO	2
RBM22	2
DCTN4	2
ZNF300	2
MRPS5	1
ZNF514	2
ANAPC1	2
ZC3H8	2
ZC3H6	2
TTL	2
CHCHD5	1
SLC20A1	2
CKAP2L	1
PSD4	1
PAX8	2
TEX37	1
THNSL2	2
CD8A	2
RMND5A	2

RNF103	2
CHMP3	1
KDM3A	2
MRPL35	1
IMMT	1
PTCD3	1
ST3GAL5	2
USP39	2
RNF181	1
VAMP5	2
VAMP8	2
MAT2A	2
SH2D6	2
CAPG	2
TCF7L1	2
KCMF1	1
TRABD2A	1
SUCLG1	1
LOC106838665	1
PIGC	1
SUCO	2
PRDX6	1
ANKRD45	1
LOC106838675	1
KLHL20	2
RC3H1	2
CALCRL	2
GULP1	2
COL3A1	2
SLC40A1	2
OSGEPL1	2
ORMDL1	1
PKIB	1
RNF217	2
TPD52L1	1
HDDC2	1
LOC106838738	1
RBMX2	2
TFAM	1
UBE2D1	1
CISD1	1
IPMK	2
FBXL17	1
EFNA5	2

JAK1	2
LOC106838756	2
LEPR	2
PDE4B	1
SGIP1	1
WDR78	1
MAP7D3	1
FHL1	1
LOC106838791	1
RPGR	1
LOC106838792	1
SPCS2	2
XRRA1	1
FAM177A1	1
PPP2R3C	1
PSMA6	1
NFKBIA	2
RALGAPA1	2
BRMS1L	1
LOC106838809	1
FAM46A	2
IBTK	2
TPBG	2
PGM3	1
RWDD2A	1
PRSS35	2
LOC106838849	2
ULK2	1
AKAP10	2
LOC106838855	1
ZSWIM7	1
LOC106838860	1
NCOR1	2
CENPV	2
UBB	2
ZNF287	1
IFT57	1
BBX	2
CCDC54	1
CBLB	2
ALCAM	2
NFKBIZ	2
RPL24	2
ZBTB11	2

PCNP	2
SENP7	2
TFG	1
TOMM70A	1
LOC106838896	1
FILIP1L	2
RNF146	1
ECHDC1	1
PON2	2
PPP1R9A	2
PEG10	2
SGCE	2
CASD1	2
COL1A2	2
BET1	1
TMEM243	2
DMTF1	2
KIAA1324L	2
LOC106838952	1
MOB3B	1
EQTN	1
IFT74	1
CAAP1	1
RIPK2	2
DECR1	1
NECAB1	2
LOC106838978	1
OTUD6B	1
LRRC69	1
LOC106838992	1
CGGBP1	1
GPRC5B	2
IQCK	1
KNOP1	1
LOC106839013	1
CCP110	1
GDE1	1
COQ7	1
LOC106839027	2
SPATA18	1
USP46	1
LOC106839055	2
LOC106839043	1
FIP1L1	2

LNX1	1
CHIC2	2
PDGFRA	2
PGLYRP2	2
AKAP8L	1
AKAP8	1
BRD4	2
ILVBL	2
SYDE1	2
CCDC105	1
GATB	1
RPS3A	2
SLC9A1	2
TMEM222	1
CD164L2	1
WASF2	2
AHDC1	2
IFI6	2
FAM76A	1
STX12	1
RPA2	1
EYA3	1
DNAJC8	1
ATPIF1	1
SESN2	2
RCC1	1
TRNAU1AP	1
RAB42	1
TAF12	1
GMEB1	2
SRSF4	2
MECR	1
PPP2R5E	1
SYNE2	2
LOC106839162	1
BLVRA	1
ZC2HC1A	1
PKIA	2
ZFHX4	2
TCEB1	1
STAU2	2
RDH10	2
RPL7	1
LOC106839193	1

TERF1	1
FAM13B	1
NME5	1
BRD8	1
GFRA3	2
FAM53C	2
KDM3B	2
EGR1	2
ETF1	1
CTNNA1	2
SIL1	1
MATR3	2
PAIP2	1
SPATA24	1
DNAJC18	1
MRPL44	1
SERPINE2	2
CUL3	1
DOCK10	2
POR	2
MDH2	1
HSPB1	1
YWHAG	2
DTX2	1
LOC106839281	2
LOC106839282	2
POLR2J	1
LRWD1	1
ALKBH4	1
ORAI2	1
PRKRIP1	1
CUX1	2
LOC106839293	2
IFT22	1
FIS1	2
ZNHIT1	2
PLOD3	2
AP1S1	1
TRIM56	2
LOC106839302	1
SRRT	1
TRIP6	2
SLC12A9	2
POP7	1

GIGYF1	2
GNB2	2
LRCH4	1
SAP25	2
AGFG2	1
TSC22D4	2
MEPCE	2
RNF139	1
TATDN1	1
NDUFB9	1
MTSS1	2
SQLE	2
NSMCE2	1
TRIB1	2
FAM84B	2
PDCL3	1
RPL31	2
TBC1D8	2
CNOT11	1
RNF149	2
CREG2	2
MAP4K4	2
IL1R1	2
MFSD9	1
CDS1	1
WDFY3	2
DCP1B	1
CECR5	1
ATP6V1E1	1
BCL2L13	1
MICAL3	2
TUBA8	1
USP18	2
TARSL2	1
TM2D3	1
PCSK6	2
SNRPA1	1
CHSY1	2
LRRK1	2
LINS1	1
MEF2A	2
LRRC28	1
NID2	1
LOC106839488	1

GNG2	2
FRMD6	2
MAP4K5	2
SOS2	1
VCPKMT	2
ARF6	2
NEMF	2
RBM28	2
FAM71F2	1
CALU	2
CCDC136	1
ATP6V1F	1
TSPAN33	1
SMO	2
AHCYL2	1
UBE2H	2
ZC3HC1	1
TMEM209	2
SSMEM1	1
CPA5	1
CEP41	1
MEST	2
C1GALT1	2
MIOS	2
RPA3	2
GLCCI1	1
ICA1	1
BTBD6	1
NUDT14	1
LOC106839592	1
CEP170B	1
SIVA1	2
ADSSL1	1
INF2	2
LOC106839602	1
KIF26A	2
LOC106839608	1
PPP1R13B	1
ZFYVE21	2
XRCC3	2
KLC1	2
APOPT1	1
BAG5	1
LOC106839624	2

TNFAIP2	1
CDC42BPB	2
TRAF3	2
RCOR1	1
TECPR2	1
CINP	1
ZNF839	2
MOK	1
WDR20	1
HSP90AA1	2
LOC106839641	1
PPP2R5C	1
LOC106839652	2
DLK1	2
SLC25A29	2
YY1	2
EVL	1
HHIPL1	2
CCNK	1
SETD3	1
LOC106839702	1
PCNXL2	2
LOC106839676	2
KCNK1	2
ACSL4	2
KCNE5	1
NXT2	2
COL4A5	2
PSMD10	2
VSIG1	1
LOC106839719	2
MID2	2
TSC22D3	2
MORC4	1
RNF128	2
LOC106839736	2
MED30	2
SYK	2
AUH	1
NFIL3	2
SPTLC1	2
NFIA	2
TM2D1	1
DOCK7	2

LOC106839777	1
FBXW8	1
TESC	2
FBXO21	1
RFC5	1
VSIG10	2
PEBP1	1
SUDS3	1
LOC106839782	2
LOC106839784	1
MGAT2	2
EMC1	2
MRTO4	1
LOC106839786	1
CAPZB	1
MINOS1	1
PLA2G2C	1
LARS	1
RBM27	1
TCERG1	2
PPP2R2B	1
DPYSL3	2
RGCC	2
NAA16	2
MTRF1	1
KBTBD7	2
WBP4	2
ELF1	2
SUGT1	1
LECT1	2
STIM2	1
VTA1	1
TEX29	1
PCID2	2
FAM98A	1
LTBP1	2
TTC27	1
BIRC6	2
YIPF4	1
SLC30A6	2
PARP8	1
EMB	1
NUDC	1
NR0B2	1

GPN2	1
PIGV	1
ARID1A	2
DHDDS	1
ZNF683	1
CD52	2
UBXN11	1
SH3BGRL3	1
SLC30A2	1
STMN1	1
PAQR7	1
MTFR1L	1
SEPN1	2
MAN1C1	2
LDLRAP1	2
TMEM50A	2
LOC106839952	1
SYF2	1
CLIC4	2
SRRM1	2
RCAN3	2
NIPAL3	2
STPG1	1
SRSF10	1
PNRC2	2
FUCA1	2
HMGCL	1
LYPLA2	1
TCEB3	1
RPL11	2
ID3	2
TCEA3	2
HNRNPR	2
KDM1A	1
LOC106840059	1
ZBTB40	2
LOC106840006	1
LOC106840061	1
HSPG2	2
USP48	2
ECE1	2
EIF4G3	2
HP1BP3	2
KIF17	1

DDOST	2
FAM43B	2
MUL1	2
CAMK2N1	2
SEPP1	2
AFAP1	1
GRPEL1	1
TADA2B	2
CCDC96	1
TBC1D14	2
KIAA0232	2
LOC106840080	2
LOC106840082	2
LOC106840083	1
OXR1	1
EIF3E	2
EMC2	1
ATP11C	2
LOC106840109	1
LOC106840123	2
ZBTB25	2
HSPA2	1
COL14A1	2
MRPL13	1
SNTB1	2
CDK7	1
MRPS36	1
CENPH	1
CCNB1	1
SLC30A5	2
PIK3R1	2
FAM171B	2
CACHD1	2
UBE2U	1
ROR1	2
PGM1	2
ITGB3BP	1
ATG4C	1
KHDRBS3	1
LOC106840173	2
LOC106840184	2
SHROOM2	2
CLCN4	2
COLCA2	2

LOC106840202	1
SIK2	2
ALG9	2
LOC106840208	1
CRYAB	2
HSPB2	2
DIXDC1	2
PIH1D2	1
LOC106840216	1
TIMM8B	1
SDHD	1
BCO2	1
PTS	1
NCAM1	2
LOC106840339	1
USP28	2
LOC106840238	1
RBM7	2
REXO2	2
CADM1	2
ZPR1	1
APOA1	2
TAGLN	2
CEP164	1
FXYD6	1
JAML	2
CD3E	2
CD3D	2
CD3G	2
UBE4A	2
ATP5L	1
KMT2A	2
TTC36	2
ARCN1	1
PHLDB1	2
TREH	1
DDX6	2
BCL9L	2
CCDC84	2
RPS25	2
TRAPPC4	1
SLC37A4	1
HYOU1	2
DPAGT1	2

NLRX1	2
MCAM	2
RNF26	1
USP2	1
FAM213A	2
DYDC2	1
DYDC1	1
FAT1	2
LOC106840368	2
TLR3	2
SORBS2	2
PDLIM3	2
CCDC110	1
CAPN5	2
ACER3	1
EMSY	2
PRKRIR	2
DGAT2	1
SERPINH1	2
GDPD5	2
RPS3	2
TPBGL	1
SLCO2B1	2
DHX15	2
CCDC149	1
PI4K2B	1
ANAPC4	1
SEL1L3	2
SMIM20	1
RBPJ	2
RPP38	1
MEIG1	1
SUV39H2	1
HSPA14	1
LOC106840454	2
FRMD4A	2
BEND7	1
SEPHS1	1
LOC106840463	1
LOC106840478	1
LOC106840479	2
NRK	2
FAM199X	1
SLC25A53	1

TMSB15B	1
ATP5J2	1
BUD31	1
PDAP1	1
ARPC1B	1
ARPC1A	1
LOC106840503	1
LMTK2	2
CCZ1	1
PMS2	2
AIMP2	1
EIF2AK1	2
USP42	1
FAM220A	1
LOC106840515	1
RAC1	2
DAGLB	2
KDELR2	1
ZDHHC4	1
LOC106840531	2
SLC4A3	2
INHA	2
OBSL1	2
CHPF	2
DES	2
DNPEP	1
STK16	1
GLB1L	1
ANKZF1	2
ATG9A	1
ZFAND2B	2
FAM134A	1
RPS12	2
SLC18B1	1
HACD3	1
DENND4A	2
TIPIN	1
RPL4	2
LOC106840613	1
ZMYND11	2
DIP2C	2
LARP4B	2
GTPBP4	1
LOC106840621	2

WDR37	2
PFKP	1
PITRM1	2
KLF6	2
THTPA	1
LOC106840636	2
SLC22A17	2
LOC106840641	2
LOC106840645	1
HOMEZ	1
RNF212B	2
ACIN1	2
LOC106840648	1
PSMB5	1
AJUBA	2
PRMT5	1
RBM23	2
REM2	2
LRP10	2
MRPL52	2
ABHD4	1
DAD1	1
LOC106840669	2
ERBB2IP	2
SREK1	2
TMED3	1
CTSH	2
LOC106840816	1
LOC106840815	1
TBC1D2B	2
CIB2	2
IDH3A	2
ACSBG1	1
DNAJA4	1
WDR61	1
CRABP1	1
PSMA4	1
UBE2Q2	1
FBXO22	1
LOC106840843	1
MYADM	2
NDUFA3	2
PRPF31	1
MBOAT7	2

TSEN34	2
RPS9	2
CDC42EP5	2
LENG9	2
LOC106840889	1
TTYH1	2
EPS8L1	1
PPP1R12C	1
HSPBP1	1
KMT5C	2
TMEM190	1
RPL28	2
ISOC2	1
ZNF628	1
NAT14	2
ZNF865	2
LOC106840950	2
EPN1	1
LOC106840980	2
GTF2I	2
GTF2IRD1	2
RFC2	1
EIF4H	1
LOC106841001	2
ZCCHC10	2
LOC106841006	1
LOC106841008	1
SOWAHA	2
SEPT8	2
LOC106841013	2
KIF3A	2
RAD50	2
IRF1	2
LOC106841020	2
PDLIM4	2
P4HA2	2
ACSL6	1
NMD3	1
PPM1L	1
YAE1D1	1
CDK13	2
SUGCT	1
LRRC27	1
INPP5A	2

UTF1	2
LOC106841076	1
ZNF511	1
CALY	2
FUOM	1
ECHS1	1
PAOX	1
MTG1	2
CDC14B	1
HABP4	1
PTCH1	2
LOC106841110	2
LOC106841118	2
LOC106841113	1
LOC106841124	1
APPBP2	2
PPM1D	1
BCAS3	1
LOC106841129	1
TBX2	2
MED13	2
RNFT1	1
RPS6KB1	1
TUBD1	1
VMP1	2
CLTC	2
LHFPL2	2
ARSB	2
BHMT	1
JMY	2
HOMER1	2
PAPD4	2
CMYA5	2
SERINC5	2
SPZ1	1
ZFYVE16	2
FAM151B	1
MSH3	1
ACOT12	1
RPS23	2
LOC106841203	1
RASA1	2
CCNH	2
TMEM161B	2

MEF2C	2
CETN3	2
LYSMD3	1
ARRDC3	2
LOC106841223	1
NR2F1	2
SLAIN2	1
NFXL1	2
CORIN	1
COMMD8	1
LOC106841238	1
GNPDA2	1
SLC30A9	2
TMEM33	2
LIMCH1	2
UCHL1	2
APBB2	2
NSUN7	1
LOC106841282	1
RBM47	2
PDS5A	2
UBE2K	1
SMIM14	1
UGDH	2
LIAS	1
RPL9	2
LOC106841231	2
LOC106841299	1
RAB5B	1
PYM1	1
DNAJC14	1
ORMDL2	1
SARNP	1
CD63	2
ITGA7	2
SIGLEC15	1
UBR1	2
TTBK2	1
STARD9	2
HAUS2	1
LRRC57	1
SNAP23	1
ZNF106	2
TMEM87A	2

EHD4	2
MGA	2
TYRO3	2
RPAP1	2
ITPKA	2
RTF1	2
NDUFAF1	1
NUSAP1	1
OIP5	1
LOC106841361	1
INO80	2
UBAP1	1
NUDT2	1
LOC106841397	1
ENHO	2
RPP25L	1
DCTN3	1
GALT	1
LOC106841407	2
AGPAT5	1
LOC106841426	1
LOC106841425	2
LOC106841432	2
CCT4	1
FAM161A	1
LOC106841438	1
XPO1	2
USP34	2
AHSA2	1
LOC106841431	1
ZMYM5	2
LOC106841427	1
LOC106841446	1
ZMYM2	2
NFX1	2
CHMP5	1
BAG1	1
DNAJA1	2
NDUFB6	1
LOC106841470	1
TOPORS	2
DDX58	2
LOC106841479	2
LONP2	1

SIAH1	2
N4BP1	2
LOC106841478	1
ZNF423	1
PGRMC1	2
UBE2A	1
SEPT6	1
SOWAHD	1
RPL39	2
UPF3B	1
FDX1L	1
ICAM1	2
DNMT1	1
LOC106841527	2
POLR2D	1
AMMECR1L	2
SAP130	1
UGGT1	2
HS6ST1	2
POU2F1	2
CD247	2
CREG1	1
RCSD1	1
MPZL1	2
MPC2	1
DCAF6	1
GPR161	2
TIPRL	1
SFT2D2	2
XCL1	2
ATP1B1	2
LOC106841567	1
AP1S2	2
ZRSR2	2
MOSPD2	2
FANCB	2
GEMIN8	1
DEK	2
TPMT	2
KIF13A	2
FAM175B	1
ZNF169	1
LOC106841626	2
CDK20	2

SPIN1	2
NXNL2	2
CKS2	1
SECISBP2	1
SEMA4D	2
LOC106841639	2
MRPL15	2
LOC106841638	1
TMEM68	2
RPS20	2
CHCHD7	1
LOC106841659	1
PENK	1
IMPAD1	1
UBXN2B	2
SDCBP	1
NSMAF	2
TOX	2
AASDH	2
PPAT	2
PAICS	2
SRP72	2
ARL9	1
HOPX	1
SPINK2	1
REST	2
POLR2B	1
IGFBP7	2
TFIP11	1
SRRD	1
ASPHD2	1
KIAA1671	2
LOC106841732	1
LOC106841731	2
LOC106841735	1
LOC106841736	1
POLR3E	1
EEF2K	2
LOC106841746	1
LOC106841750	1
ANKS4B	2
ZP2	2
ARHGAP36	1
LMCD1	2

RAD18	1
SRGAP3	2
THUMPD3	1
SETD5	2
ACSS1	2
APMAP	2
CST7	2
LOC106841791	2
LOC106841779	1
LOC106841792	1
CST8	1
LOC106841793	2
LOC106841794	1
NAPB	2
GZF1	1
NXT1	1
UBA5	1
ACKR4	2
DNAJC13	2
NUDT16	1
DDX18	1
INHBB	2
RALB	1
TMEM185B	2
EPB41L5	2
PTPN4	2
SLC15A4	2
GLT1D1	1
FZD10	2
PIWIL1	1
RAN	1
LY6E	1
LOC106841942	2
GLI4	1
TOP1MT	1
ZC3H3	1
GSDMD	2
NAPRT	1
EEF1D	1
TSTA3	1
SCRIB	2
PUF60	2
NRBP2	2
PLEC	2

PARP10	2
GRINA	1
SPATC1	1
OPLAH	1
EXOSC4	1
LOC106841927	1
SHARPIN	1
MAF1	2
MROH1	1
DGAT1	1
TMEM249	1
SLC52A2	2
CPSF1	2
CYHR1	1
LRRC14	1
LRRC24	2
ZNF251	2
COMMD5	1
ZNF7	2
LOC106841950	2
RPL8	1
CYTIP	2
GPD2	2
NR4A2	2
CENPW	1
TRMT11	1
HINT3	1
NCOA7	2
LOC106841973	1
RGS12	2
LRPAP1	1
EZR	1
TAGAP	1
LOC106842013	1
SOD2	1
WTAP	2
ACAT2	1
TCP1	1
MRPL18	1
PNLDC1	1
IGF2R	2
SLC22A3	2
MAP3K4	2
AGPAT4	2

PACRG	1
QKI	2
LOC106842058	2
MPC1	2
RPS6KA2	2
RNASET2	2
FGFR1OP	1
MLLT4	2
CXXC4	2
TET2	2
PPA2	1
INTS12	2
TM9SF3	2
ZNF518A	2
ENTPD1	1
ALDH18A1	2
GPC3	2
GPC4	2
HS6ST2	2
MBNL3	2
NFAT5	2
NQO1	2
NOB1	2
WWP2	1
PSMD7	1
ZFHX3	2
ARL13B	1
PROS1	2
MACROD2	1
FLRT3	1
APLP2	2
ZBTB44	2
RABL2A	1
THAP2	1
ZFC3H1	2
TSPAN8	1
CNOT2	1
LOC106842194	2
RAB3IP	1
CCT2	1
FRS2	2
YEATS4	2
MED13L	2
SDSL	1

PLBD2	1
IQCD	1
DDX54	2
RASAL1	2
DTX1	2
LOC106842212	2
RPL6	2
NAA25	1
ALDH2	1
ACAD10	2
ATXN2	2
CCDC63	1
PPP1CC	1
VPS29	1
GPN3	1
ARPC3	1
ATP2A2	2
IFT81	1
P2RX4	2
CAMKK2	1
ANAPC5	1
ORAI1	1
MORN3	1
TMEM120B	1
PSMD9	1
WDR66	1
MLXIP	2
DIABLO	1
CLIP1	2
ZCCHC8	2
RSRC2	2
CCDC62	1
VPS37B	2
OGFOD2	1
MPHOSPH9	1
LOC106842327	1
CDK2AP1	1
SBNO1	1
RILPL2	1
TMED2	2
DDX55	2
EIF2B1	1
GTF2H3	1
CCDC92	1

NCOR2	2
LOC106842364	1
LOC106842351	1
PHF3	2
PTP4A1	1
SC5D	2
ARHGEF12	2
ANXA7	2
CFAP70	1
MRPS16	1
DNAJC9	1
FAM149B1	1
P4HA1	2
LOC106842371	2
CHML	2
FH	2
FMN2	2
MIEF1	1
ATF4	2
RPS19BP1	1
FAM83F	1
TNRC6B	2
SGSM3	2
MKL1	2
ST13	2
DNAJB7	1
RBX1	2
EP300	2
L3MBTL2	1
RANGAP1	1
SLC35F5	2
ACTR3	2
JAM2	2
ATP5J	1
GABPA	1
APP	2
HIVEP1	2
EDN1	2
NEDD4L	2
ZNF532	2
SEC11C	1
LMAN1	2
CCBE1	1
PMAIP1	2

BAMBI	2
LOC106842506	1
ARMC4	1
RAB18	2
MAP4	2
CIAO1	1
SNRNP200	2
ITPRIPL1	1
ARID5A	2
KANSL3	2
LMAN2L	1
CNNM4	2
CNNM3	2
LOC106842542	1
LOC106842544	1
TMEM131	2
VWA3B	1
LOC106842554	1
LOC106842593	1
LOC106842594	1
LOC106842607	1
PRSS37	1
SSBP1	1
MRPS33	1
BRAF	2
ADCK2	2
DENND2A	1
MKRN1	1
KDM7A	2
PARP12	2
TBXAS1	2
CLEC2L	2
LOC106842633	2
LOC106842634	2
UBN2	2
KIAA1549	2
SVOPL	1
TRIM24	1
THOC7	2
PSMD6	1
ADAMTS9	2
MAGI1	2
SLC25A26	1
LRIG1	2

ZBBX	1
LOC106842667	1
USP15	1
PGM2	2
RELL1	2
ARAP2	2
CNTLN	1
LOC106842686	2
ISLR	2
STRA6	2
LOC106842692	2
UBL7	1
ARID3B	2
CLK3	1
EDC3	1
ASNS	1
SDHAF3	1
LOC106842713	1
DYNC1I1	1
CD34	2
LOC106842723	2
LOC106842724	1
SLC46A2	2
SNX30	1
INIP	1
KIAA1958	2
PIK3R4	2
SH3BP5	1
METTL6	1
EAF1	1
LOC106842761	1
HACL1	1
GJA1	1
SERINC1	2
LOC106842773	1
CDC42SE2	1
LYRM7	1
ISOC1	2
SLC12A2	2
LOC106842806	1
PRRC1	2
TEX43	1
PHAX	2
ALDH7A1	1

CSNK1G3	2
PPIC	2
ZNF474	1
FTMT	1
WRNIP1	2
SERPINB1	1
SERPINB9	2
ACVR2B	1
EXOG	2
WDR48	1
GORASP1	1
CSRNP1	2
SLC25A38	1
RPSA	2
EIF1B	2
ENTPD3	2
LOC106842882	2
NDUFC2	1
KCTD14	1
RSF1	2
CLNS1A	1
PAK1	2
CSTF2	1
ADGRL2	2
CXCL12	2
ZNF22	2
SEC61B	1
COL15A1	2
CORO2A	1
NANS	2
ANP32B	2
HEMGN	1
TRMO	1
XPA	1
NCBP1	1
TSTD2	1
TMOD1	2
TDRD7	1
BCKDHB	1
UBE2G2	1
SUMO3	2
PTTG1IP	2
FAM207A	1
POFUT2	2

COL18A1	2
COL6A1	2
SPATC1L	1
LSS	2
MCM3AP	2
LOC106842955	1
YBEY	1
LOC106842990	2
YWHAB	2
TOMM34	1
STK4	1
PI3	2
LOC106843015	1
SDC4	2
SYS1	2
TP53TG5	1
PIGT	2
WFDC2	2
LOC106843025	1
DNTTIP1	1
UBE2C	1
SNX21	1
ACOT8	1
CTSA	2
CD40	2
SLC35C2	2
TMSB4X	2
GALNT2	2
SLAIN1	1
MYCBP2	2
FBXL3	2
CLN5	1
KCTD12	2
LMO7	2
UCHL3	1
COMMD6	1
TBC1D4	2
LOC106843095	2
KLF5	1
DIS3	1
LOC106843110	2
TMEM123	2
DYNLT3	1
THOC3	2

SFXN1	2
LOC106843143	1
TMEM237	2
ALS2	2
ANO10	1
HIGD1A	1
LOC106843176	2
ZBTB47	2
NKTR	2
SS18L2	1
VIPR1	2
LYZL4	1
CCK	1
TRAK1	1
MRPL39	1
APOD	1
PPP1R2	1
ACAP2	1
CENPC	1
YTHDC1	2
LOC106843234	2
CDYL	1
RPP40	1
LYRM4	1
FARS2	1
LOC106843241	2
NRN1	2
MED29	1
ZFP36	2
RPS16	2
SUPT5H	1
LOC106843267	1
DYRK1B	1
FBL	2
PSMC4	1
LOC106843290	1
PLD3	2
SERTAD1	2
ITPKC	2
SNRPA	1
RAB4B	1
EGLN2	1
LOC106843317	2
LOC106843319	1

WFS1	2
ACTN1	2
ZFP36L1	2
LOC106843327	1
MOCS2	1
NDUFS4	1
ARL15	2
SNX18	2
GZMK	2
GZMA	2
GPX8	2
DHX29	2
SKIV2L2	1
LOC106843363	1
DDX4	1
IL6ST	2
MAP3K1	2
SETD9	1
GPBP1	2
PLK2	2
NUDT19	1
ANKRD27	2
PDCD5	1
LOC106843343	1
DPY19L3	2
ZNF507	2
MIB1	2
LOC106843403	1
ROCK1	2
LOC106843411	1
RAB27A	1
LOC106843422	1
PIGB	2
CCPG1	1
DYX1C1	1
PYGO1	2
NEDD4	2
MNS1	1
ZNF280D	2
ANO6	2
LOC106843440	2
ARID2	1
SCAF11	2
SLC38A1	2

SLC38A2	2
LOC106843438	2
LOC106843523	2
TRIM26	2
PPP1R11	1
ZNRD1	2
ZFP57	1
CHCHD1	1
ZSWIM8	2
NDST2	1
VCL	2
AP3M1	1
ADK	1
KAT6B	2
VDAC2	1
COMTD1	1
SPCS3	1
ASB5	1
SPATA4	1
GPM6A	2
CPSF6	2
SLC35E3	2
RAP1B	1
IFNG	2
DYRK2	2
YIPF5	2
SH3RF2	1
SPATA6L	1
CDC37L1	2
AK3	2
INSL6	1
LOC106843622	1
RIC1	2
LOC106843614	1
ATM	2
ACAT1	1
CUL5	2
ELMOD1	2
CWF19L2	1
RAB2A	1
CHD7	2
ASPH	2
NKX3-1	1
SLC25A37	1

PRDM1	2
ATG5	1
AIM1	2
RTN4IP1	1
QRSL1	1
RAB4A	1
CCSAP	2
NUP133	1
URB2	2
LOC106843714	2
ZFP2	2
ZNF879	2
RUFY1	1
HNRNPH1	2
CANX	2
MAML1	2
SQSTM1	2
LOC106843725	2
RNF130	1
GFPT2	2
CNOT6	2
LOC106843749	1
GPHN	2
FAM71D	1
KLC3	1
ERCC2	2
PPP1R13L	2
CD3EAP	2
ERCC1	2
FOSB	2
VASP	2
DMWD	1
RSPH6A	1
IRF2BP1	2
10-Mar	1
MRC2	2
TLK2	1
WDR44	2
NTM	2
AXL	2
HNRNPUL1	2
TMEM91	2
BCKDHA	1
LOC106843824	2

LOC106843830	2
LCA5	2
LOC106843847	2
DGKE	2
TMEM100	2
MMD	1
HLF	2
SFSWAP	2
ULK1	2
EP400	2
NOC4L	2
PXMP2	1
ANKLE2	2
GOLGA3	1
ZNF605	2
ZNF84	1
ZNF891	2
ZNF10	2
ZNF268	2
ANHX	2
LOC106843887	1
TOMM5	1
FRMPD1	1
EXOSC3	1
SLC25A51	1
ZNF214	1
RBMXL2	1
LOC106843960	1
HSD17B12	2
EXT2	2
LOC106843951	1
LOC106843983	1
CRY2	2
LOC106843985	1
PEX16	1
MDK	2
AMBRA1	2
ATG13	2
ZNF408	2
CKAP5	1
PACSIN3	2
ACP2	1
MADD	2
MYBPC3	2

SLC39A13	1
PSMC3	1
RAPSN	2
CELF1	2
PTPMT1	1
KBTBD4	1
NDUFS3	1
MTCH2	1
FNBP4	2
NUP160	2
PLD2	2
PSMB6	1
CXCL16	2
MED11	1
PELP1	2
ARRB2	2
ACADVL	2
GABARAP	1
CTDNEP1	1
YBX2	1
EIF5A	1
GPS2	1
LOC106844188	1
ACAP1	1
PLSCR3	2
TMEM256	1
SPEM1	1
LOC106844195	1
TMEM102	1
FGF11	2
CHRNB1	2
LOC106844202	2
POLR2A	2
LOC106844213	2
SENP3	1
EIF4A1	2
SAT2	1
TP53	2
TMEM88	2
NAA38	1
CHD3	2
TRAPPC1	1
PER1	2
VAMP2	2

TMEM107	1
BORCS6	1
ORAOV1	1
CCND1	2
MRPL21	1
CPT1A	2
MTL5	1
PPP6R3	2
KMT5B	2
CHKA	1
NDUFS8	1
ZNF333	2
NDUFB7	1
GIPC1	1
DNAJB1	2
DDX39A	1
ADGRE5	2
ASF1B	1
LOC106844282	1
LOC106844328	1
MRI1	1
CCDC130	1
IER2	2
STX10	2
TRMT1	1
NFIX	2
RAD23A	1
CALR	2
FARSA	1
GCDH	1
DNASE2	2
RNASEH2A	1
JUNB	2
TMEM109	1
LOC106844359	1
LOC106844360	1
MS4A13	1
MS4A5	1
PATL1	2
OSBP	2
PBLD	2
SIRT1	2
DNAJC12	2
AAED1	2

LOC106844422	2
CTSV	2
LOC106844430	1
ITPA	1
DDRGK1	2
MRPS26	1
PTPRA	2
VPS16	1
PCED1A	2
LOC106844435	1
LOC106844437	1
CPXM1	2
EBF4	2
CCNL1	2
PTX3	2
LOC106844467	2
MLF1	1
HNRNPM	2
2-Mar	1
ANGPTL4	2
RPS28	2
NDUFA7	1
CD320	2
CERS4	2
CCL25	2
ELAVL1	1
TIMM44	1
CTXN1	1
MAP2K7	2
TRAPPC5	1
STXBP2	1
CAMSAP3	2
PNPLA6	1
MCOLN1	2
ZNF358	2
ARHGEF18	2
INSR	2
LOC106844528	2
PSMG1	1
ETS2	2
FAM222B	1
RPL23A	2
RAB34	2
PROCA1	1

SUPT6H	2
SDF2	2
KIAA0100	2
LOC106844578	2
ALDOC	2
PIGS	2
SLC46A1	1
TMEM199	1
TNFAIP1	2
TMEM97	2
NLK	2
WSB1	2
NF1	2
EVI2B	2
RAB11FIP4	1
COPRS	1
UTP6	1
SUZ12	1
TEFM	1
ZNF207	2
PSMD11	2
TMEM98	2
SPACA3	1
CCT6B	1
ZNF830	2
LIG3	1
RFFL	1
FNDC8	1
PEX12	1
AP2B1	1
LOC106844656	1
MMP28	2
CCL5	2
LOC106844668	2
LOC106844672	2
DDX52	1
DUSP14	1
AATF	1
GGNBP2	1
ZNHIT3	1
PVRL3	1
CD96	2
PHLDB2	2
DCUN1D4	1

TMEM69	2
IPP	2
CNIH4	2
DNAH14	1
ENAH	2
SRP9	1
EPHX1	2
SDE2	1
H3F3A	2
LOC106844775	1
VTI1B	1
CCDC60	2
TMEM233	1
PRKAB1	2
CIT	2
RAB35	1
GCN1	2
RPLP0	2
PXN	2
PLA2G1B	2
LOC106844790	2
TRIAP1	1
GATC	2
SRSF9	2
DYNLL1	1
COQ5	1
POP5	1
MLEC	1
LOC106844806	2
LOC106844743	1
SART3	2
ISCU	1
LOC106844826	1
NCL	2
LOC106844828	1
LOC106844831	1
PTMA	2
PDE6D	2
COPS7B	1
NPPC	2
EFHD1	1
GIGYF2	1
ATG16L1	2
USP40	2

LOC106844856	1
DNAJB3	1
SH3BP4	2
BRI3BP	1
UBC	2
SCARB1	2
LOC106844881	1
CHURC1	1
MAX	2
FUT8	2
LOC106844902	2
STK25	1
BOK	2
THAP4	1
COG2	1
LACTB2	1
TRAM1	1
NCOA2	2
SUPT20H	1
ALG5	1
SMAD9	2
SOCS6	2
RRP12	2
PGAM1	1
EXOSC1	1
ZDHHC16	1
MMS19	2
UBTD1	1
LOC106844961	1
MORN4	1
PI4K2A	2
MARVELD1	2
ZFYVE27	2
CRTAC1	2
KPNA4	1
TRIM59	1
SMC4	1
IFT80	1
SPATA19	1
JAM3	2
NCAPD3	1
VPS26B	1
ACAD8	1
THYN1	2

CCAR1	2
STOX1	2
DDX21	2
SRGN	2
VPS26A	1
SUPV3L1	1
HK1	1
TSPAN15	1
LOC106845024	2
AIFM2	1
AFAP1L2	2
ABLIM1	1
DIP2B	2
FAM186A	1
LIMA1	1
CERS5	2
LOC106845049	1
GPD1	1
SMARCD1	2
AQP5	2
FAIM2	1
BCDIN3D	2
NCKAP5L	2
TMBIM6	1
LOC106845064	1
MCRS1	2
SPATS2	1
NDFIP2	1
RBM26	2
FRA10AC1	1
SLC35G1	2
USP14	2
CETN1	1
ENOSF1	1
ALG13	2
NKAP	2
NDUFA1	2
SEPW1	2
GLTSCR2	2
ZNF541	1
NAPA	1
C5AR2	2
BBC3	1
SAE1	1

ZC3H4	2
TMEM160	2
ARHGAP35	2
AP2S1	1
STRN4	2
SLC1A5	2
PRKD2	1
CALM3	1
LOC106845175	2
LOC106845176	1
ETV1	1
ARL4A	1
EPHA5	2
TNFRSF14	2
LOC106845204	1
PEX10	2
RER1	1
SKI	2
FAAP20	2
LOC106845219	2
GNB1	2
NADK	1
LOC106845222	2
CDK11B	1
MMP23B	2
LOC106845191	2
SSU72	1
VWA1	2
CCNL2	2
AURKAIP1	1
MXRA8	2
CPSF3L	1
UBE2J2	1
FAM132A	2
AGRN	2
ISG15	2
NOC2L	2
QSER1	2
WT1	2
RCN1	2
ELP4	1
NAV2	2
SPTY2D1	2
LOC106845288	1

MRPS2	2
LOC106845330	1
LOC106845332	2
WDR5	1
BRD3	2
SLC2A6	2
CACFD1	1
STKLD1	1
SURF4	2
SURF2	1
RPL7A	2
SURF6	1
LOC106845296	2
GBGT1	1
LOC106845370	1
GTF3C4	2
SETX	1
MED27	1
RAPGEF1	2
UCK1	1
POMT1	1
PRRC2B	2
FAM78A	1
NUP214	2
AIF1L	2
ABL1	2
ASS1	2
NCS1	2
GPR107	2
USP20	1
TOR1A	2
PTGES	1
PRRX2	1
ASB6	1
NTMT1	1
LOC106845407	1
IER5L	2
CRAT	1
FAM73B	2
SH3GLB2	2
DOLK	1
PHYHD1	2
LRRC8A	2
CCBL1	1

ENDOG	1
ZDHHC12	1
SET	2
WDR34	1
SPTAN1	2
GLE1	1
ODF2	1
CERCAM	2
URM1	1
COQ4	1
TRUB2	1
SWI5	1
GOLGA2	1
LOC106845449	2
FAM102A	2
DPM2	1
ST6GALNAC4	2
AK1	1
ENG	2
CDK9	2
STXBP1	2
RPL12	2
SLC2A8	1
RALGPS1	1
ZBTB34	2
ZBTB43	2
FAM71E1	1
EMC10	1
CLEC11A	1
LOC106845491	1
KLK1	2
KLK4	1
DPP7	2
MAN1B1	2
ENTPD2	2
LOC106845525	1
LCNL1	2
PTGDS	2
C8G	1
FBXW5	1
EDF1	1
PHPT1	1
RABL6	2
CCDC183	1

TMEM141	1
CAMSAP1	1
UBAC1	1
NACC2	2
LOC106845548	1
GPSM1	2
DNLZ	2
SNAPC4	2
SDCCAG3	1
PMPCA	1
NOTCH1	2
AGPAT2	1
FAM69B	1
LRRC1	1
ELOVL5	2
GSTA4	1
UBE2J1	1
PM20D2	2
SRSF12	1
PNRC1	2
KIF22	1
MAZ	2
MVP	2
LOC106845617	1
TAOK2	2
HIRIP3	1
LOC106845626	1
FAM57B	1
ALDOA	2
PPP4C	1
YPEL3	2
MAPK3	1
CORO1A	2
BOLA2B	1
SLX1A	1
LOC106845634	2
SGF29	1
NUPR1	2
CLN3	2
CAND1	1
ATP2B1	2
DUSP6	2
HMGB4	1
ZMYM6NB	1

ZMYM6	2
CCDC93	1
LOC106845679	1
INSIG2	2
LOC106845687	2
ETS1	2
ARHGAP32	2
MINA	1
CRYBG3	1
ARL6	1
RANGRF	1
SLC25A35	1
ODF4	1
RPL26	2
NDEL1	1
MYH10	2
CCDC42	1
STX8	1
GAS7	2
ELAC2	2
LOC106845780	1
LOC106845779	1
PMP22	2
TEKT3	1
CDRT4	1
LOC106845785	1
LOC106845805	2
LOC106845808	1
PLK1	1
DCTN5	1
RBBP7	2
SYAP1	1
LOC106845814	1
CTPS2	2
PRICKLE1	2
PPHLN1	1
ZCRB1	1
GXYLT1	2
DAAM1	2
DACT1	2
KIAA0586	1
TIMM9	1
ARID4A	2
PSMA3	1

ACTR10	1
SLC35B3	1
CEP126	1
LOC106845873	1
YAP1	2
BIRC3	2
LOC106845877	2
WDR1	1
SLC2A9	1
TMEM128	1
LYAR	1
STX18	1
PRKX	2
MXRA5	2
SGMS1	1
MINPP1	2
ATAD1	2
PTEN	1
ZC3H7B	2
TOB2	2
PHF5A	1
ACO2	2
POLR3H	1
A4GALT	2
ARFGAP3	1
PACSIN2	1
TTLL1	1
BIK	2
TSPO	2
SCUBE1	2
EFCAB6	1
SULT4A1	1
SAMM50	1
LDOC1L	2
PRR5	2
ARHGAP8	2
LOC106845961	1
NUP50	1
KIAA0930	1
FAM118A	2
SMC1B	1
RIBC2	1
PPARA	2
CERK	2

TBC1D22A	1
FAM19A5	1
BRD1	2
ZBED4	2
CRELD2	1
PIM3	2
TTLL8	1
MOV10L1	1
TRABD	2
TUBGCP6	2
HDAC10	2
PLXNB2	2
DENND6B	1
PPP6R2	1
LMF2	2
NCAPH2	1
LOC106846016	1
TYMP	2
ODF3B	1
CPT1B	1
CHKB	2
MAPK8IP2	1
ARSA	1
ACR	1
INHBA	2
ROCK2	2
PDIA6	2
ATP6V1C2	1
ODC1	1
PERP	1
ARFGEF3	2
NHSL1	1
CCDC28A	1
REPS1	1
MNAT1	1
PPM1A	1
DHRS7	1
JKAMP	1
NOL8	2
ZNF484	2
HELLS	2
TBC1D12	2
NOC3L	1
LOC106846085	2

LGALS3	2
MAPK1IP1L	2
WDHD1	2
SAMD4A	1
LOC106846116	2
LOC106846118	2
CGRRF1	1
CDKN3	1
PALB2	1
NDUFAB1	1
EARS2	2
GGA2	1
SCNN1B	2
USP31	2
LOC106846161	1
DCUN1D3	2
LYRM1	1
AFF2	1
FMR1NB	1
FMR1	2
LOC106846175	2
WNK2	1
NINJ1	1
CARD19	1
SUSD3	1
FGD3	1
CENPP	1
OGN	2
GPR180	1
CLDN10	1
DZIP1	2
MBNL2	2
IPO5	1
FARP1	2
DOCK9	2
HAX1	1
UBAP2L	2
LOC106846241	1
TPM3	2
RPS27	2
JTB	2
SLC39A1	2
CRTC2	2
INTS3	2

NPR1	2
SNAPIN	1
CHTOP	2
S100A1	1
S100A13	2
S100A16	2
S100A6	2
LELP1	1
LOC106846215	1
CHRAC1	2
AGO2	2
SLC45A4	2
PIP4K2A	2
LOC106846327	1
SPAG6	1
COMMD3	1
DNAJC1	1
MLLT10	2
DSEL	2
LOC106846330	2
MRPL1	1
CNOT6L	2
TNRC18	2
WIPI2	1
FOXK1	1
RAP1GDS1	1
EIF4E	1
METAP1	1
ADH5	2
SCCPDH	1
AHCTF1	2
CDC42BPA	2
ADCK3	2
PSEN2	2
PARP1	1
PARM1	2
RCHY1	1
THAP6	1
TMX3	2
CRTC1	2
COMP	1
UPF1	1
COPE	1
DDX49	1

SUGP2	1
MRPS18C	1
COQ2	2
PLAC8	2
COPS4	1
SEC31A	2
SCD5	2
TMEM150C	2
ENOPH1	1
HNRNPDL	2
HNRNPD	2
BRDT	1
LOC106846441	1
RPAP2	1
RPL5	2
FAM69A	1
MTF2	2
TMED5	2
CCDC18	1
RNPC3	2
ZNF687	2
PSMD4	1
SCNM1	1
GABPB2	2
MLLT11	1
CDC42SE1	2
BNIPL	1
PRUNE	2
FAM63A	2
CERS2	2
SETDB1	2
ARNT	2
GOLPH3L	1
ENSA	1
MCL1	2
TARS2	2
RPRD2	2
PRPF3	2
MRPS21	1
CIART	2
APH1A	2
ANP32E	1
PLEKHO1	1
VPS45	2

OTUD7B	2
BOLA1	1
LOC106846569	2
LOC106846572	2
LOC106846574	2
LOC106846577	2
LOC106846576	1
LOC106846581	1
LOC106846586	2
LOC106846589	2
LOC106846587	2
LOC106846593	1
ACP5	2
ELOF1	1
CNN1	2
ECSIT	1
PRKCSH	2
TMEM205	1
TSPAN16	1
DOCK6	2
SPC24	1
LDLR	2
LOC106846631	1
YIPF2	1
CARM1	1
TMED1	1
ILF3	2
SLC44A2	2
CDKN2D	1
ATG4D	1
CDC37	1
LOC106846673	2
LOC106846648	2
LOC106846651	1
ZNF671	2
LOC106846680	1
ZNF606	2
ZNF544	1
A1BG	1
ZNF584	1
ZNF132	2
SLC27A5	1
TRIM28	1
CHMP2A	1

UBE2M	1
MZF1	2
LOC106846661	2
CNNM1	2
GOT1	1
SLC25A28	2
DNMBP	1
CHUK	1
CWF19L1	2
BLOC1S2	1
TSNAX	1
EGLN1	2
EXOC8	2
GNPAT	1
LOC106846718	2
FAM89A	1
8-Mar	1
ZFAND4	1
FAM21C	2
DCAF12L2	1
SMNDC1	2
ADD3	1
NDUFB8	1
SCD	2
TCTE3	1
PHF10	1
DIMT1	1
KIF2A	1
LOC106846776	2
NDUFAF2	1
PTPN14	2
CENPF	1
NT5DC3	2
HSP90B1	2
LOC106846787	1
TDG	2
HCFC2	1
NFYB	1
EID3	1
LOC106846823	1
ALDH1L2	1
KIAA1033	1
LOC106846802	1
NUAK1	2

CKAP4	2
LOC106846806	1
RFX4	1
LRRIQ1	1
LOC106846815	2
SPTLC3	2
LOC106846851	2
LOC106846853	1
TPM4	2
RAB8A	1
CIB3	1
FAM32A	1
AP1M1	1
KLF2	2
CALR3	1
CHERP	2
SLC35E1	2
MED26	1
SMIM7	2
TMEM38A	2
SIN3B	2
HAUS8	1
MYO9B	2
USE1	1
NR2F6	1
USHBP1	2
ABHD8	1
MRPL34	2
DDA1	1
ANO8	2
BST2	2
SLC27A1	2
PGLS	1
GLB1L3	2
LOC106846910	2
ATRX	2
LOC106846922	2
PGK1	1
LOC106846934	1
FGFR2	2
PLPP4	2
PKP2	2
YARS2	1
FGD4	2

BICD1	2
KIAA1551	2
AMN1	1
METTL20	2
MGST1	2
FAM221B	1
HINT2	2
NPR2	2
RGP1	1
GBA2	1
TLN1	2
CA9	1
CCDC107	2
TESK1	1
FAM214B	1
FANCG	1
VCP	2
PHF24	1
CEP85L	1
PLN	1
NUS1	2
GOPC	1
DCBLD1	1
FAM162B	1
TRAPPC3L	1
FAM26F	2
DSE	2
TSPYL1	2
TSPYL4	2
NT5DC1	1
HDAC2	2
FAM229B	1
FYN	1
LOC106847054	2
REV3L	2
KIAA1919	1
RPF2	2
AMD1	2
CDK19	2
SMARCA1	2
OCRL	2
ZDHHC9	2
UTP14A	2
ELF4	2

AIFM1	2
MIF	1
LOC106847146	1
LOC106847117	1
LOC106847118	1
GGT1	2
SNRPD3	2
GUCD1	2
SPECC1L	1
BCR	2
LOC106847147	1
DROSHA	2
PDZD2	2
LOC106847156	2
GOLPH3	2
PDXDC1	1
NTAN1	1
RRN3	1
LOC106847164	1
BFAR	2
PARN	1
MKL2	1
PIAS1	2
ADGRL3	2
EPB41L3	1
ATXN1	2
FAM8A1	1
NUP153	1
ATAD2B	2
MTRF1L	1
LOC106847211	2
YPEL5	1
EHD3	2
LOC106847249	2
LOC106847250	2
UBE2D3	1
LOC106847251	1
SLC9B1	1
BDH2	1
CENPE	1
CITED2	2
HECA	2
PRDM5	2
MAD2L1	1

LOC106847269	1
OSTF1	1
CARNMT1	1
CD163L1	1
LOC106847301	1
CLSTN3	2
RBP5	2
C1R	2
C1S	2
LPCAT3	2
EMG1	1
PHB2	1
LOC106847315	2
ENO2	2
SPSB2	1
TPI1	1
GPR162	1
LOC106847330	2
MLF2	2
ZNF384	2
ING4	1
ACRBP	1
CHD4	2
GAPDH	1
MRPL51	1
VAMP1	2
TAPBPL	2
USP6NL	1
LOC106847359	1
SRI	2
DBF4	1
AURKC	1
PEG3	2
ZNF835	1
LOC106847405	1
LYRM2	1
MDN1	2
CASP8AP2	2
MAP3K7	2
SHCBP1L	1
DHX9	2
RGS16	2
RNASEL	2
PRKG1	2

CSTF2T	2
LOC106847487	1
FAM173B	1
CCT5	1
CMBL	2
6-Mar	2
DAP	1
LOC106847471	2
TAF1B	1
YWHAQ	1
IAH1	1
CPSF3	1
ITGB1BP1	1
ASAP2	1
MBOAT2	2
KIDINS220	2
ID2	2
RNF144A	2
ALLC	1
RPS7	2
TRAPPC12	1
TSSC1	1
NAALADL2	2
SPATA16	1
ECT2	1
TNFSF10	2
FNDC3B	2
PLD1	2
TNIK	1
RPL22L1	2
ZNF148	2
HEG1	2
ITGB5	2
UMPS	1
LOC106847508	2
LOC106847551	1
VWA8	2
AKAP11	2
FTH1	2
RAB3IL1	1
FADS2	2
FADS1	2
FEN1	1
MYRF	2

SDHAF2	1
CPSF7	2
TMEM216	1
DDB1	2
SIPA1L1	2
LOC106847601	2
ENO1	2
TP53RK	1
ZMYND8	2
LOC106847650	2
PRKAB2	2
BCL9	2
GPR89A	2
CD160	2
RNF115	2
POLR3C	1
PIAS3	2
RBM8A	2
TXNIP	2
RASSF2	2
PRND	1
PRNP	2
SMOX	2
LOC106847691	1
MAVS	1
AP5S1	1
LOC106847683	1
ATRN	2
LOC106847693	2
PEBP4	1
EGR3	2
CCAR2	2
PDLIM2	2
SORBS3	2
PPP3CC	1
SLC39A14	2
PIWIL2	1
POLR3D	1
REEP4	1
HR	2
NUDT18	1
XPO7	2
DOK2	1
ERH	2

SLC39A9	2
SRSF5	2
LOC106847737	1
SYNJ2BP	1
CPXM2	2
BUB3	1
ACADSB	1
EBNA1BP2	1
FAM183A	1
LOC106847777	1
SLC2A1	2
SVBP	2
LOC106847828	1
LOC106847795	1
P3H1	2
YBX1	1
PPIH	2
PPCS	1
ZMYND12	1
FOXJ3	2
SCMH1	2
CTPS1	2
CITED4	2
NFYC	2
DIRAS3	2
WLS	2
LRRC40	2
SRSF11	2
CTH	1
RGS22	1
POLR2K	2
SPAG1	1
RNF19A	2
PABPC1	1
YWHAZ	2
ZNF706	1
ZNF596	2
LONRF1	1
PRKD1	2
C7	2
MROH2B	1
OXCT1	2
FBXO4	1
NQO2	2

BPHL	2
LOC106847879	2
PSMG4	2
SLC22A23	2
FAM50B	1
EIF5B	2
TXNDC9	1
CDC20	1
ELOVL1	1
SZT2	2
PTPRF	2
KDM4A	2
ATP6V0B	2
DMAP1	1
RPS19	2
ARHGEF1	2
RABAC1	1
ZNF574	1
DEDD2	2
ZNF526	1
CIC	2
PAFAH1B3	1
TMEM145	1
MEGF8	2
CNFN	2
LOC106847944	1
LIPE	1
RP2	2
NDUFB11	2
RBM10	2
UBA1	2
CDK16	1
USP11	2
LOC106848000	2
ARAF	2
TIMP1	2
DMD	2
NRIP1	2
HSPA13	2
LOC106848027	1
ROBO2	2
ROBO1	2
GBE1	2
ZBTB18	2

DEPTOR	2
TAF2	2
ENPP2	2
NOV	2
MAL2	2
TNFRSF11B	2
LANCL1	1
RPE	2
MAP2	2
LOC106848073	2
ALKBH2	1
UBE3B	1
MVK	1
GIT2	1
ANKRD13A	1
LOC106848065	1
TCF7L2	2
CASP7	2
DCLRE1A	2
NHLRC2	2
CCDC186	2
TDRD1	1
ANXA13	1
FBXO32	2
WDYHV1	1
LOC106848109	1
LOC106848112	2
PLEKHA2	2
HTRA4	1
TM2D2	1
ZFX	2
EIF2S3	2
KLHL15	2
SAT1	2
ACOT9	2
PRDX4	1
ZNF280B	2
PRAME	1
LOC106848164	1
CUL4A	1
LAMP1	1
TMCO3	2
GAS6	2
LOC106848205	1

UPF3A	1
VPS35	2
ORC6	1
LOC106848232	1
ITFG1	2
TMEM126B	1
CREBZF	2
CCDC89	1
CCDC83	1
PICALM	1
LOC106848256	2
CCDC81	1
ME3	1
PRSS23	2
LOC106848239	2
TOP3B	2
PPM1F	2
YPEL1	1
PPIL2	1
LOC106848294	1
SDF2L1	1
YDJC	1
UBE2L3	1
LOC106848279	1
HIC2	2
TMEM191C	1
PI4KA	2
SNAP29	1
CRKL	2
USP25	1
PKDCC	1
EML4	2
DLG1	2
MFI2	2
NCBP2	1
SENP5	2
PIGX	2
CEP19	1
WDR53	1
TCTEX1D2	1
PCYT1A	1
CPNE8	2
KIF21A	2
PRKDC	2

CEBPD	2
SPIDR	2
PSMB4	1
POGZ	2
TUFT1	2
SNX27	1
RIIAD1	1
MRPL9	1
OAZ3	1
THEM4	1
S100A10	1
S100A11	2
YME1L1	2
ACBD5	1
ABI1	2
APBB1IP	2
PHLDB3	1
IRGQ	2
ZNF576	1
IRGC	1
SMG9	1
ZNF283	2
RELB	2
NEK1	1
CLCN3	2
MFAP3L	1
SCML4	1
SEC63	2
EDEM1	2
ARL8B	1
BHLHE40	2
ITPR1	2
SUMF1	2
CRBN	1
TRNT1	1
COG6	2
FOXO1	2
NDUFA6	1
FAM109B	1
NAGA	2
WBP2NL	1
SREBF2	2
SNU13	1
XRCC6	1

DESI1	1
LOC106848504	1
POLDIP3	2
LOC106848498	1
OGFRL1	2
LCOR	2
HIF1A	2
TRMT5	1
TRIM2	2
FBXW7	2
SDR39U1	1
KHNYN	2
NYNRIN	2
DHRS1	1
TINF2	1
NEDD8	1
MDP1	1
TSSK4	1
TM9SF1	2
IPO4	1
REC8	1
IRF9	2
RNF31	2
PSME2	2
EMC9	1
PSME1	2
DCAF11	1
LOC106848600	1
TPR	2
LOC106848629	1
COL12A1	2
LOC106848636	1
PTPRG	2
FHIT	1
FAM107A	2
KCTD6	2
PDHB	1
PXK	2
RPP14	2
ABHD6	2
DNASE1L3	2
FLNB	2
SLMAP	2
DENND6A	2

ARF4	2
LOC106848644	2
APPL1	2
IL17RD	1
ARHGEF3	2
FAM208A	2
CCDC66	1
WNT5A	2
FAM60A	1
CAPRIN2	2
IPO8	2
LOC106821737	2
NEK4	1
SPCS1	1
GLT8D1	1
GNL3	1
PBRM1	2
SMIM4	2
STAB1	1
NISCH	2
PHF7	1
BAP1	1
PPM1M	1
ALAS1	2
POC1A	1
RPL29	1
ABHD14A	1
SHISA5	2
NME6	1
RRAGB	2
TLR2	2
KIAA0922	1
ZNF496	2
RNF187	1
LOC106821823	1
LOC106821822	2
TRIM17	1
IBA57	1
MRPL55	1
LOC106821808	1
ARF1	1
CD99	1
SIMC1	2
ARL10	2

NOP16	1
HIGD2A	1
CLTB	1
FAF2	2
RNF44	1
TSPAN17	1
UIMC1	1
PAFAH1B1	1
METTL16	1
SGSM2	1
TSR1	1
SRR	1
RPA1	2
LOC106822043	1
PRPF8	2
RILP	1
SLC43A2	2
PITPNA	1
INPP5K	1
MYO1C	2
YWHAE	2
RPH3AL	2
LOC106822051	1
FAM101B	2
VPS53	1
FAM57A	1
GEMIN4	1
LOC106821995	1
GLOD4	1
RNMTL1	1
NXN	2
ABR	1
GOSR1	1
CPD	2
BLMH	1
NSRP1	2
ABHD15	1
TAOK1	2
ZCCHC6	2
ISCA1	1
GOLM1	2
NAA35	2
AGTPBP1	1
LOC106822024	1

IMPA1	1
SNX16	1
DCP1A	2
ACTR8	2
LOC106822067	1
SLC3A2	2
HRASLS5	1
SMPD4	1
LOC106822092	1
KLHL22	1
MRPL40	1
LOC106822102	1
UFD1L	1
CLDN5	2
SEPT5	1
TBX1	1
GNB1L	1
TXNRD2	1
ARVCF	2
AKAP4	1
OSBPL10	1
GPD1L	1
CMTM7	1
CMTM6	2
DYNC1LI1	1
SMC1A	2
RIBC1	1
HSD17B10	2
HUWE1	2
ZFP91	1
FADD	1
FAS	2
ACTA2	2
STAMBPL1	2
NUDT21	1
OGFOD1	1
MT3	2
LOC106822242	2
LOC106822243	2
LOC106822244	2
NUP93	2
HERPUD1	1
CPNE2	1
FAM192A	1

RSPRY1	2
ARL2BP	1
CIAPIN1	1
COQ9	1
POLR2C	1
CCDC102A	2
ADGRG1	2
ADGRG3	2
DRC7	1
KATNB1	1
KIFC3	2
TEPP	2
ZNF319	2
CFAP20	1
CCDC113	1
CNOT1	1
GOT2	2
FNIP1	2
RAPGEF6	2
KLF3	2
FAM114A1	1
KLHL5	1
LOC106822280	2
ABCB7	2
RLIM	2
LOC106822351	2
LOC106822349	2
LOC106822321	2
MRS2	2
DCDC2	1
ZNF711	2
APOOL	1
ZNF658	2
PDCD6IP	2
CLASP2	2
UBP1	2
LOC106822388	1
LMO4	2
LOC106822383	2
SH3GLB1	1
CLCA4	2
LOC106822399	1
URB1	2
LOC106822406	1

PAXBP1	2
ABCD2	1
LRRK2	1
LOC106822485	2
LOC106822494	2
NIP7	1
PDF	1
VPS4A	1
SNTB2	2
CDH3	2
PRMT7	2
SLC7A6OS	1
PLA2G15	1
NFATC3	2
SLC12A4	2
LCAT	2
PSMB10	2
PSKH1	1
NRN1L	1
EDC4	1
NUTF2	2
RANBP10	2
GFOD2	1
LOC106822512	1
ENKD1	1
PARD6A	1
CTCF	2
LOC106822430	2
FAM65A	2
LOC106822431	2
ATP6V0D1	1
ZDHHC1	2
FHOD1	2
TMEM208	1
KIAA0895L	1
LOC106822444	1
LOC106822527	1
LOC106822525	1
TIMM10	1
UBE2L6	2
SERPING1	2
ZDHHC5	1
MED19	1
TMX2	1

LOC106822561	1
CTNND1	2
CTSB	2
FDFT1	1
GATA4	2
MAST2	2
LOC106822596	1
POMGNT1	2
LRRC41	2
LOC106822600	1
NSUN4	1
MKNK1	2
ATPAF1	1
TEX38	1
EFCAB14	1
MPC1L	1
ATP6AP2	2
CHM	2
ZNF331	2
LOC106822652	1
LOC106822653	1
LOC106822636	1
PPP2R1A	2
LOC106822669	2
FOLH1B	2
LOC106822680	2
ARMT1	1
ZBTB2	2
FKBP15	2
SLC31A1	2
PRPF4	1
WDR31	1
ALAD	1
POLE3	1
LOC106822720	1
RGS3	2
LOC106822729	1
DHCR7	2
MYO6	2
SENP6	2
RNF220	2
TMEM53	1
LOC106822744	1
KIF2C	1

RPS8	2
PTCH2	2
EIF2B3	1
UROD	1
ZSWIM5	2
EHMT1	2
ARRDC1	2
ZMYND19	1
DPH7	1
MRPL41	1
PNPLA7	2
NELFB	2
LOC106822769	1
FAM166A	1
TUBB4B	1
RNF208	2
LOC106822785	1
NDOR1	2
TMEM203	1
SSNA1	1
TMEM210	1
LRRC26	1
LOC106822857	1
LOC106822903	2
TGIF1	2
LOC106822912	2
ZBTB14	2
TTC39C	1
CABYR	1
IMPACT	2
SS18	2
PSMA8	1
CHST9	2
MRPS24	1
LOC106822888	2
UBE2D4	1
PGAM2	1
AEBP1	2
POLD2	1
YKT6	1
CAMK2B	2
TMED4	1
ZMIZ2	1
PPIA	2

LOC106822946	1
PURB	2
LOC106822929	2
CCM2	1
TBRG4	2
RAMP3	1
LOC106822880	1
TNS3	2
HUS1	1
UPP1	1
ZPBP	1
HNRNPA0	2
KLHL3	2
SMAD5	2
CXCL14	2
LOC106822985	2
PCBD2	1
TXNDC15	1
LOC106823008	2
DDX46	2
CAMLG	1
SEC24A	2
RFT1	2
PRKCD	1
MED10	1
NSUN2	1
PAPD7	2
LOC106823002	1
LOC106823016	2
SEMA5A	2
LOC106823046	2
NGFRAP1	2
BEX4	2
LOC106823039	2
TCEAL7	2
WBP5	2
TCEAL4	2
HNRNPK	2
LOC106823066	2
KIF27	1
LOC106823063	1
GKAP1	1
UBQLN1	2
CYLC1	1

LOC106823104	1
ARMCX2	2
ARMCX3	2
HNRNPH2	1
GLA	2
RPL36A	2
TIMM8A	1
TAF7L	1
TRIT1	2
CAP1	1
PPT1	2
RLF	2
TMCO2	1
SMAP2	1
CD109	2
ZKSCAN8	1
NKAPL	1
LOC106823149	2
LOC106823175	2
MSL2	2
PPP2R3A	2
EPHB1	2
CEP63	1
ANAPC13	1
AMOTL2	2
RYK	2
LOC106823197	2
TF	2
SLC23A2	2
TMEM230	1
PCNA	1
CDS2	2
GPCPD1	2
LOC106823194	2
TRMT6	2
CRLS1	1
LRRN4	2
BMP2	2
TMX4	1
PLCB4	2
LOC106823215	2
HNRNPA2B1	2
CBX3	2
SNX10	1

LOC106823223	1
EIF2S2	1
CHMP4B	1
ACTL10	1
CBFA2T2	2
SNTA1	1
PRSS42	1
SETD2	2
KIF9	1
KLHL18	2
PTPN23	2
SCAP	2
ELP6	2
SMARCC1	2
CHMP2B	2
CNKSR3	1
LOC106823283	2
PJA1	1
EFNB1	2
LSM14B	1
STX7	1
CTGF	2
ENPP1	2
KCNH2	2
ABCB8	2
CDK5	1
SLC4A2	2
FASTK	2
AGAP3	2
ABCF2	1
CHPF2	1
SMARCD3	2
NUB1	1
WDR86	1
CRYGN	1
PRKAG2	2
LOC106823316	2
GALNTL5	1
KMT2C	2
XRCC2	1
ACTR3B	2
INSIG1	2
RBM33	2
RNF32	1

LMBR1	1
NOM1	2
UBE3C	2
DNAJB6	1
ATP5A1	2
HAUS1	1
ST8SIA5	2
PIAS2	1
LOC106823394	1
DENND1B	1
ASPM	1
IGSF11	1
B4GALT4	2
ARHGAP31	2
TMEM39A	2
CIDEA	2
AFG3L2	2
PRELID3A	2
SPIRE1	1
LOC106823413	1
GNAL	1
MPPE1	1
SLK	2
OBFC1	1
SH3PXD2A	2
NEURL1	1
USMG5	1
VAC14	2
MTSS1L	2
SF3B3	2
COG4	1
LOC106823438	1
PDPR	2
BZW1	1
CLK1	2
PPIL3	1
ORC2	1
FAM126B	2
NDUFB3	1
CFLAR	2
TRAK2	2
STRADB	2
ALS2CR11	1
NIFK	1

TSN	2
DTNA	2
MAPRE2	1
ZNF397	1
ZNF24	2
GALNT1	2
LOC106823556	1
RPRD1A	1
SLC39A6	2
ELP2	1
FHOD3	2
TPGS2	1
HMGN3	1
PHIP	2
IRAK1BP1	1
NEK7	1
PTPRC	2
LOC106823537	2
ZNF281	2
CAMSAP2	2
TMEM9	2
LOC106823574	1
HPRT1	2
PHF6	1
CCDC160	1
LOC106823583	2
TNKS1BP1	2
SSRP1	2
CHST10	2
CLASP1	2
CFAP221	1
ZNF317	2
LOC106823688	1
UHMK1	2
UAP1	1
HSD17B7	2
LRRC58	2
FSTL1	2
NDUFB4	1
GTF2E1	1
GOLGB1	2
IQCB1	1
CCDC58	1
FAM162A	1

PARP9	2
DTX3L	2
PARP15	2
PARP14	2
DIRC2	2
PDIA5	2
SEC22A	1
HACD2	2
MYLK	2
LOC106823701	2
TIMMDC1	1
LOC106823776	2
HARS2	2
HARS	1
DND1	2
IK	1
NDUFA2	1
TMCO6	2
SLC35A4	1
LOC106823762	2
HBEGF	2
PFDN1	1
LOC106823788	2
PPID	1
RAPGEF2	2
SNRPB2	1
FAM193A	1
RNF4	2
LOC106823799	2
WHSC1	1
ACAD9	1
ARHGAP17	2
TNRC6A	2
RBBP6	2
PRKCB	2
DOK1	2
AUP1	2
LOC106823843	2
MOGS	2
WDR54	1
LOC106823857	1
DCTN1	2
SLC4A5	2
DGUOK	1

BOLA3	1
MOB1A	1
XPNPEP1	1
NGLY1	1
TOP2B	2
NR1D2	2
RPL15	2
NKIRAS1	1
UBE2E1	1
ZNF385D	2
DUSP22	1
PARP16	2
CLPX	1
RASL12	2
SLC51B	1
MTFMT	1
PLEKHO2	2
RBPMS2	2
OAZ2	1
ZNF609	2
PLOD2	2
LOC106823930	2
NIPA2	1
CYFIP1	2
TUBGCP5	2
LOC106823932	2
LOC106823933	1
LOC106823949	1
RANBP2	2
LIMS1	2
GCC2	2
SULT1C4	1
SOWAHC	2
SEPT10	1
CXADR	2
BTG3	2
TSPAN6	1
LOC106823978	1
LARP1	2
CNOT8	1
MRPL22	1
TTLL7	1
PRKACB	2
RPF1	1

GNG5	2
SPATA1	1
SSX2IP	1
LPAR3	1
SYDE2	2
LOC106824037	1
DDAH1	2
CYR61	2
AP3B1	2
WDR41	1
CRHBP	1
F2R	2
IQGAP2	1
POC5	1
POLK	2
HMGCR	2
ANKRD31	1
FAM169A	2
ENC1	2
SUMO1	1
NOP58	2
BMPR2	2
FAM117B	2
ICA1L	1
WDR12	1
CARF	2
NBEAL1	2
LOC106824075	1
FRMD3	2
LOC106824087	1
LOC106824088	1
TTC9	1
PCNX	2
TSSC4	1
CD81	2
IGF2	2
MRPL23	2
LSP1	2
CTSD	2
LOC106824112	1
PCYT1B	2
HELB	2
GRIP1	2
BRD7	2

PAPD5	1
HEATR3	2
CNEP1R1	1
LOC106824131	1
LANCL2	1
EGFR	2
FCGRT	2
NOSIP	1
IRF3	1
BCL2L12	1
PRMT1	2
ADM5	2
TSKS	1
MED25	2
PTOV1	2
NUP62	1
VRK3	1
POLD1	2
ATP6V1G1	1
LOC106824205	2
PAPPA	2
CDK5RAP2	1
CNTRL	1
RAB14	2
GSN	2
STOM	1
DAB2IP	2
NHLRC3	2
PROSER1	2
RUNX1T1	2
LOC106824239	1
CLDND1	1
CPOX	2
ST3GAL6	2
DCBLD2	2
NCOA4	1
LOC106824267	1
PARG	2
OGDHL	2
TSGA13	1
WDR60	1
LOC106824290	1
ESYT2	2
NCAPG2	1

CDCA2	1
KCTD9	2
LOC106824296	1
RARRES2	2
LRRC61	1
ATP6V0E2	1
ZNF398	2
LRRC72	1
ANKMY2	2
AHR	2
SNX13	2
LOC106824346	1
TWIST1	2
TWISTNB	1
ITGB8	2
SP4	2
CDCA7L	2
RAPGEF5	1
LOC106824333	1
TOMM7	1
FAM126A	1
KLHL7	1
NUPL2	1
MALSU1	2
TRA2A	2
GNS	2
RASSF3	2
SRGAP1	2
PRELID3B	1
ATP5E	2
LOC106824410	1
LOC106824403	2
LOC106824414	2
LOC106824416	1
TFRC	2
RUBCN	2
FYTTD1	2
LRCH3	2
IQCG	1
RPL35A	2
HTRA1	2
PLEKHA1	2
TACC2	2
NSMCE4A	1

LOC106824443	1
TRAPPC10	1
AGPAT3	1
RRP1	2
HNRNPU	2
LOC106824460	1
LOC106824463	1
DESI2	1
LOC106824499	1
LOC106824496	1
ERAL1	2
FLOT2	1
MYO18A	1
LOC106824546	2
MRPS10	1
LOC106824520	1
BYSL	1
TOMM6	1
NFYA	1
OARD1	1
DAAM2	1
SAYSD1	1
GLO1	2
LOC106824584	1
BTBD9	1
ZFAND3	1
CCDC167	1
RNF8	2
PIM1	1
PPIL1	1
CPNE5	2
SRSF3	2
BRPF3	2
MAPK13	1
MAPK14	2
SRPK1	1
ARMC12	1
FKBP5	1
TSR2	1
GNL3L	2
LOC106824623	1
PHGDH	2
HMGCS2	1
NOTCH2	2

RGAG1	2
TMEM164	2
HDX	1
ASH2L	1
LSM1	1
BAG4	1
DDHD2	2
WHSC1L1	2
FGFR1	2
ODF2L	1
NMI	2
RIF1	2
ARL5A	2
STAM2	1
FMNL2	2
PRPF40A	2
RPL37	2
PRKAA1	1
TTC33	1
DAB2	2
FYB	2
RICTOR	2
OSMR	2
LIFR	2
NUP155	1
LOC106824755	2
NIPBL	2
NADK2	1
SPEF2	1
DNAJC21	1
AMACR	2
IZUMO3	1
ELAVL2	2
LOC106824742	1
NDRG1	2
PHF20L1	1
EFR3A	2
ASAP1	1
MYC	2
ZBTB33	2
LAMP2	2
CUL4B	1
CSGALNACT1	1
ZMAT1	2

TSGA10	1
LIPT1	1
MRPL30	2
ERICH3	1
CRYZ	1
TYW3	1
TMEM47	2
ARL14EP	1
KCNA4	1
LIN7C	2
LGR4	2
CCDC34	1
FSIP2	1
NCOA6	2
TP53INP2	2
PIGU	2
MAP1LC3A	1
DYNLRB1	1
LOC106824940	2
SCAF8	2
LOC106824948	2
AP2A2	2
TSPAN4	2
POLR2L	1
CD151	2
PNPLA2	1
RPLP2	2
SLC25A22	1
PDDC1	1
TALDO1	1
TMEM80	2
DEAF1	1
IRF7	2
PHRF1	2
RASSF7	2
LRRC56	1
HRAS	1
LOC106824986	2
EAPP	1
CCDC124	1
PIK3R2	1
IFI30	1
MPV17L2	1
KIAA1683	1

JUND	2
LSM4	1
ISYNA1	1
ELL	1
FKBP8	1
KXD1	1
UBA52	1
TMEM59L	2
LOC106825024	2
LOC106825028	2
DDX39B	2
LTB	2
PRRC2A	2
BAG6	2
GPANK1	1
CSNK2B	1
ABHD16A	2
LY6G6D	2
DDAH2	2
CLIC1	2
VARS	1
LSM2	1
HSPA1L	1
LOC106825057	2
GNG12	1
GADD45A	2
LOC106825070	1
RND3	2
MMADHC	1
KIF5C	2
EPC2	2
ORC4	1
ZEB2	2
BCL3	2
PVR	2
ZNF227	2
GADD45G	2
LOC106825123	2
LOC106825121	2
OFD1	2
LOC106825131	2
LOC106825137	2
FAM111B	2
FAM111A	2

PSMC2	1
KMT2E	2
SRPK2	1
PUS7	2
LOC106825223	2
RINT1	2
LOC106825222	1
SYPL1	1
NAMPT	2
PRKAR2B	2
HBP1	2
COG5	2
DUS4L	1
BCAP29	1
DLD	1
LAMB1	2
NME8	1
EPDR1	2
STARD3NL	2
LOC106825199	2
AMPH	1
HMGB1	2
LOC106825253	1
ZNF350	1
LOC106825278	1
LOC106825269	2
TOLLIP	1
MOB2	1
CSTB	2
RRP1B	1
HSF2BP	1
LOC106825325	1
LOC106825359	1
LOC106825356	2
DDX24	1
OTUB2	1
LOC106825340	1
BTBD7	2
UBR7	1
LOC106825386	1
TMEM251	1
CHGA	2
GOLGA5	1
LGMN	2

RIN3	1
NDUFB1	1
ATXN3	1
TRIP11	2
PPP4R3A	2
RPS6KA5	2
CALM1	1
PSMC1	1
KCNK13	2
TDP1	1
EFCAB11	2
FANK1	1
DHX32	1
BCCIP	1
UROS	1
LOC106825344	1
TEX36	1
CTBP2	2
ZRANB1	2
PIGG	2
LOC106825438	1
GAK	1
TMEM175	1
IDUA	2
FGFRL1	2
RNF212	1
TXNL4A	1
LOC106825450	1
RFXANK	1
NR2C2AP	1
MAU2	2
GATAD2A	1
TSSK6	1
NDUFA13	2
LPAR2	2
GMIP	1
ATP13A1	2
LOC106825489	2
LOC106825494	2
LOC106825493	1
RPP21	2
GNL1	1
ABCF1	1
PPP1R10	2

MRPS18B	1
LOC106825484	1
MDC1	2
FLOT1	2
IER3	2
DDR1	2
LOC106825501	2
GTF2H4	1
USP22	2
NATD1	1
MAP2K3	2
BTNL9	2
ZFP62	2
MGAT1	2
PLEKHA5	2
AEBP2	2
SLCO1A2	1
PYROXD1	1
LDHB	1
ABCC9	2
CMAS	1
ETNK1	2
SOX5	1
LYRM5	1
KRAS	2
SPATS2L	1
SGOL2	1
LOC106825581	2
LOC106825601	2
LOC106825600	2
LOC106825604	2
FLNA	2
TKTL1	2
SSR4	1
IDH3G	2
RRAGD	2
ANKRD6	2
MKI67	1
PTPRE	1
TOM1	1
HMOX1	2
MCM5	2
APOL6	2
RBFOX2	1

GNA14	2
GNAQ	2
MINK1	2
LOC106825695	1
SLC25A11	1
RNF167	2
ZNF594	2
NUP88	1
DHX33	2
DERL2	1
KIAA0753	1
TXNDC17	1
XAF1	2
FBXO39	1
TEKT1	1
MYBBP1A	2
UBE2G1	1
ANKFY1	2
ATP2A3	2
NCBP3	1
ITGAE	1
EMC6	1
TAX1BP3	2
SHPK	2
LOC106825755	2
LOC106825782	1
HTATSF1	1
RBMX	2
ESF1	2
NDUFAF5	1
HNRNPH3	2
TRIM41	2
GNB2L1	2
EFCAB2	1
TRAPPC2	1
RAB9A	2
MKKS	1
ADD1	2
NDC80	1
SMCHD1	2
LPIN2	2
GHITM	1
CPEB4	2
CRIM1	2

FEZ2	1
HEATR5B	1
GPATCH11	1
EIF2AK2	2
CEBPZ	2
PRKD3	2
CDC42EP3	1
ATL2	1
GALM	1
SRSF7	2
LOC106825873	1
GEMIN6	1
DHX57	2
MORN2	1
ARHGEF33	1
SOS1	2
MAP4K3	1
LOC106825846	2
THUMPD2	2
ZC4H2	2
MANBA	1
AMDHD2	1
KCTD5	1
PRSS21	1
TCEB2	1
SRRM2	2
FLYWCH2	1
TNFRSF12A	2
HCFC1R1	2
THOC6	2
IL32	2
ZNF205	2
ZNF263	2
NAA60	1
LOC106826082	1
CLUAP1	1
SLX4	1
CREBBP	2
ADCY9	2
GLIS2	1
PAM16	1
VASN	2
DNAJA3	1
NMRAL1	1

HMOX2	1
UBALD1	2
MGRN1	2
NUDT16L1	1
ANKS3	2
LOC106826067	1
SEPT12	1
SMIM22	1
ROGDI	1
GLYR1	2
UBN1	1
NRDC	1
OSBPL9	2
EPS15	2
RNF11	1
LOC106825974	1
NPR3	2
CCNA1	1
SPG20	2
RAB21	1
TBC1D15	1
LOC106826120	2
CARS	1
NAP1L4	1
CDKN1C	2
LOC106826125	2
GPBP1L1	2
NASP	1
AKR1A1	1
PRDX1	1
LOC106826138	1
TESK2	1
TOE1	1
LOC106826152	2
BICC1	2
PHYHIPL	1
ANK3	2
RHOBTB1	1
ARID5B	2
ADO	1
EGR2	2
JMJD1C	2
REEP3	1
LOC106826209	1

SUCLG2	1
PACS2	1
TEX22	1
MTA1	1
CRIP2	1
CRIP1	2
H6PD	2
RPL14	2
LOC106826235	2
LOC106826238	1
LOC106826239	1
PRMT2	2
DIP2A	2
CCDC59	1
LIN7A	1
KIF2B	1
UTP18	1
LOC106826470	1
LOC106826468	1
TOB1	2
WFIKKN2	2
LUC7L3	2
ABCC3	2
SPATA20	1
RSAD1	1
ACSF2	2
LRRC59	1
MRPL27	1
XYLT2	2
COL1A1	2
LOC106826323	1
PDK2	1
KAT7	1
SLC35B1	1
SPOP	2
ZNF652	2
PHOSPHO1	1
SNF8	1
CALCOCO2	1
SKAP1	2
NFE2L1	2
CDK5RAP3	1
SCRN2	2
LRRC46	1

MRPL10	1
KPNB1	2
NPEPPS	2
SOCS7	1
LOC106826548	1
MLLT6	2
CISD3	1
PCGF2	2
PSMB3	1
PIP4K2B	2
LOC106826272	1
RPL23	2
RPL19	2
FBXL20	2
MED1	2
CDK12	2
PPP1R1B	2
TCAP	2
PNMT	1
ERBB2	2
MIEN1	1
ZPBP2	1
ORMDL3	1
THRA	2
CASC3	2
WIPF2	1
TOP2A	1
SMARCE1	1
KRT10	1
KRT19	2
JUP	2
P3H4	2
NT5C3B	1
KLHL10	1
KLHL11	2
ACLY	2
TTC25	1
NKIRAS2	1
DHX58	2
KAT2A	2
HSPB9	1
RAB5C	1
GHDC	2
STAT5B	2

STAT3	2
PTRF	2
NAGLU	2
COASY	1
FAM134C	2
TUBG1	1
LOC106826279	2
PLEKHH3	2
EZH1	2
LOC106826429	1
BECN1	1
LOC106826365	1
RPL27	2
IFI35	2
RND2	1
NBR1	1
TMEM106A	2
LOC106826571	1
HOXD8	1
MTX2	1
HNRNPA3	2
NFE2L2	2
AGPS	1
TTC30B	1
PRKRA	2
FKBP7	1
PLEKHA3	1
SESTD1	2
CWC22	2
UBE2E3	1
SSFA2	2
PDE1A	1
DNAJC10	1
LOC106826643	1
RPL10	2
ATP6AP1	2
GDI1	2
LAGE3	1
BGN	2
FAM58A	1
LOC106826665	2
BCAP31	2
LOC106826673	1
LCMT1	1

IKZF5	1
LOC106826676	1
FAM24B	1
LOC106826680	1
RNGTT	1
PYGB	2
ABHD12	1
GINS1	2
NINL	2
NANP	1
LOC106826701	1
LOC106826704	2
PDHA2	1
MANSC1	2
BORCS5	1
DUSP16	2
CDKN1B	1
GPRC5A	1
HEBP1	2
GSG1	1
EMP1	2
LOC106826751	1
ATF7IP	2
PLBD1	2
LOC106826752	1
WBP11	1
LOC106826736	1
MGP	2
ARHGDIB	2
EPS8	2
STRAP	1
DERA	1
PLS3	2
PSMC6	1
GNPNAT1	1
FERMT2	2
DDHD1	1
DGCR2	2
TSSK1B	1
TSSK2	1
SLC25A1	2
UFM1	1
SPARC	2
LOC106826818	2

WDR74	1
LOC106826822	1
NXF1	2
TMEM223	1
TMEM179B	1
POLR2G	1
TTC9C	1
BSCL2	1
GNG3	1
UBXN1	1
METTL12	2
LOC106826837	1
B3GAT3	2
EML3	2
MTA2	2
EEF1G	1
ASRGL1	1
RECK	2
GLIPR2	2
CLTA	2
RNF38	1
GPX3	2
TNIP1	1
ANXA6	2
CCDC69	1
GM2A	2
LOC106826864	2
HSDL2	1
PTBP3	2
UGCG	2
DNAJC25	1
PTGR1	1
KIAA0368	2
LOC106826910	1
LOC106826911	2
LOC106826905	2
AKAP2	2
EPB41L4B	2
FRRS1L	2
TMEM245	2
CTNNAL1	2
FAM206A	1
IKBKAP	2
ACTL7A	1

ACTL7B	1
KLF4	2
RAD23B	1
ZNF462	2
LOC106826887	2
TMEM38B	1
FKTN	2
FSD1L	1
SLC44A1	2
ASNA1	1
FBXW9	1
LOC106826929	1
DHPS	1
WDR83OS	1
MAN2B1	2
LOC106826940	1
LOC106826937	2
SPACA1	1
LOC106826945	2
SLC10A6	1
LOC106826948	1
LOC106826950	1
CDV3	1
LOC106826952	2
LOC106826953	2
LOC106826955	2
LOC106826951	2
KCND1	1
PQBP1	2
KTN1	2
ATG14	1
FBXO34	1
OSR2	2
LOC106827012	1
GPRASP1	2
LOC106827032	1
TRAM2	2
MCM3	2
PGK2	1
CRISP2	1
CENPQ	1
CD2AP	2
ZNF37A	2
LOC106827066	1

LOC106827072	1
EEF1A1	2
CC2D2A	1
CD38	2
CDKL5	1
CFL2	2
BAZ1A	1
SRP54	1
LOC106827103	1
ANGEL2	2
FLVCR1	2
SPATA45	1
ATF3	2
NENF	1
TMEM206	1
PPP2R5A	1
LPGAT1	1
NEK2	1
SLC30A1	2
LOC106827196	1
TRAF5	2
RCOR3	1
SERTAD4	2
LOC106827184	2
DIEXF	1
LOC106827161	2
TRAF3IP3	2
G0S2	2
ZNF346	2
NSD1	2
RAB24	1
PRELID1	1
LMAN2	2
PFN3	1
PRR7	2
PDLIM7	2
FAM193B	2
LOC106827181	2
B4GALT7	1
RMND5B	1
NHP2	1
HNRNPAB	2
PHYKPL	2
CLK4	2

LOC106827164	2
LOC106827206	1
MTMR12	1
ZFR	2
SUB1	1
LOC106827221	2
REV1	2
LOC106827223	2
SERPINB6	1
LOC106827250	1
IGF1	1
NUP37	2
CCDC53	1
GNPTAB	2
CHPT1	1
ARL1	1
LOC106827259	2
GAS2L3	2
SLC17A8	2
SCYL2	1
APAF1	2
IKBIP	2
SLC25A3	2
TMPO	2
NEDD1	1
LOC106827315	2
LOC106827316	2
RBAK	2
ACTB	1
IL13RA2	1
LRCH2	2
LOC106827334	1
PHF8	2
FAM120C	2
LRIF1	1
CD53	2
LAMTOR5	2
UBL4B	1
AHCYL1	2
CSF1	2
EPS8L3	1
GSTM3	1
LOC106827366	1
LOC106827367	2

AMPD2	2
PSMA5	1
PSRC1	1
SARS	1
LOC106827369	1
TMEM167B	1
TAF13	1
WDR47	1
GPSM2	1
PRPF38B	2
LOC106827380	1
LOC106827404	2
ENTPD6	1
SLC39A10	2
STK17B	1
EBP	2
RBM3	2
WDR13	2
ITGAV	2
ZC3H15	1
KDM6A	2
SLTM	2
CCNB2	1
RBM41	2
NUP62CL	1
LOC106827464	1
AKR1E2	1
LOC106827466	1
LOC106827473	2
LOC106827474	2
LOC106827475	2
MAML2	2
CEP57	1
SESN3	1
LOC106827509	1
CWC15	2
AMOTL1	1
LOC106827525	1
ANKRD49	1
MED17	2
LOC106827493	1
TAF1D	2
LOC106827516	1
CCDC67	1

SLC36A4	2
CHORDC1	2
NAALAD2	2
TANGO2	1
TRMT2A	1
RANBP1	1
RTN4R	1
DGCR6L	1
LOC106827540	2
TOM1L1	1
ARC	2
CFAP126	1
PCP4L1	2
NDUFS2	2
DEDD	1
NIT1	1
PFDN2	2
KLHDC9	1
USF1	2
TSTD1	2
F11R	2
TDRD3	2
PCDH20	2
ZNF184	2
ZNF391	2
R3HCC1	1
LOXL2	1
LOC106827636	1
LOC106827646	2
LOC106827644	2
LOC106827643	2
LOC106827642	2
PDZD11	1
NCKAP1	2
FSCB	1
LOC106827724	1
KLHL28	2
FAM179B	2
PRPF39	2
FKBP3	1
MIS18BP1	1
LOC106827699	1
LOC106827697	1
FOS	2

TMED10	2
NEK9	2
ZC2HC1C	1
ACYP1	1
MLH3	2
EIF2B2	1
YLPM1	2
ISCA2	1
NPC2	1
ALDH6A1	2
BBOF1	1
ENTPD5	1
COQ6	1
FAM161B	1
ZNF410	1
PTGR2	2
ELMSAN1	2
PNMA1	2
DNAL1	1
PSEN1	1
LOC106827685	1
RBM25	2
ZFYVE1	1
DCAF4	1
DPF3	2
RGS6	1
CEP135	1
CEBPG	1
LRP3	2
TSPYL2	1
PDCD2	1
TBP	1
LOC106827777	2
PLEK2	1
EIF2S1	1
ATP6V1D	1
LOC106827808	1
